# Organocatalytic Asymmetric Synthesis of Si-Stereogenic Siloxanols

**DOI:** 10.1021/acscatal.3c03932

**Published:** 2024-01-05

**Authors:** Jacob J. Dalton, Adilene Bernal Sánchez, Austin T. Kelly, James C. Fettinger, Annaliese K. Franz

**Affiliations:** Department of Chemistry, University of California Davis, One Shields Ave, Davis, California 95616, United States

**Keywords:** asymmetric catalysis, organocatalyst, silylation, desymmetrization, hydrogen bonding, Lewis base, stereogenic silicon

## Abstract

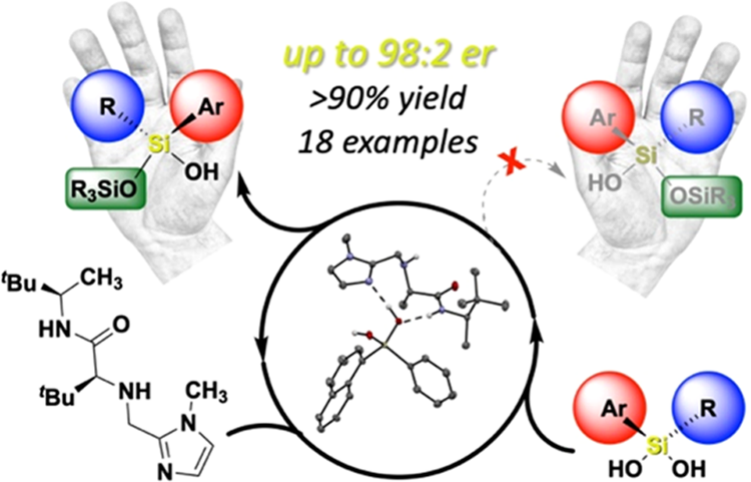

We report the organocatalytic synthesis of Si-stereogenic compounds via desymmetrization of a prochiral silanediol with a chiral imidazole-containing catalyst. This metal-free silylation method affords high yields with enantioselectivity up to 98:2 for various silanediol and silyl chloride substrate combinations (including secondary alkyl, vinyl, and H groups), accessing products with potential for further elaboration. NMR and X-ray studies reveal insight into the H-bonding interactions between the imidazole organocatalyst and the silanediol and the dual activating role of the Lewis basic imidazole to account for the high enantioselectivity.

Access to functionalized, chiral-at-silicon molecules in high enantiopurity is important to further expand the applications of organosilicon molecules in synthetic chemistry,^1−6^ material science,^7−9^ and medicinal chemistry.^10−12^ The synthesis of Si-stereogenic compounds has provided a unique challenge since their first early investigation in 1907.^13−15^ The catalytic asymmetric synthesis of enantioenriched Si-stereogenic silanes remains rare, particularly methods that produce organosilanes with handles for further synthetic transformation.^16,17^ While several examples for desymmetrization of prochiral disilanes have been reported,^18−28^ there are only two recent reports of asymmetric methods for desymmetrization of prochiral silanediols to access enantioenriched silanols:^29−31^ He and co-workers reported a Cu-catalyzed sigma-bond metathesis approach for SiO-H insertion (Figure 1a),^29^ and Zhu and Oestreich reported an imidodiphosphorimidate (IDPi)-catalyzed silylation with allylsilanes (Figure 1b).^30^ Both the Cu- and IDPi-catalyzed methods require a *tert*-butyl group for high levels of enantioenrichment. While several options exist for the enantioselective organocatalytic silylation of alcohols (Figure 1c),^32−36^ organocatalytic transformations of silanols remain rare, with only four recent related examples giving access to Si-stereogenic silyl ethers.^27,30,37,38^

**Figure 1:**
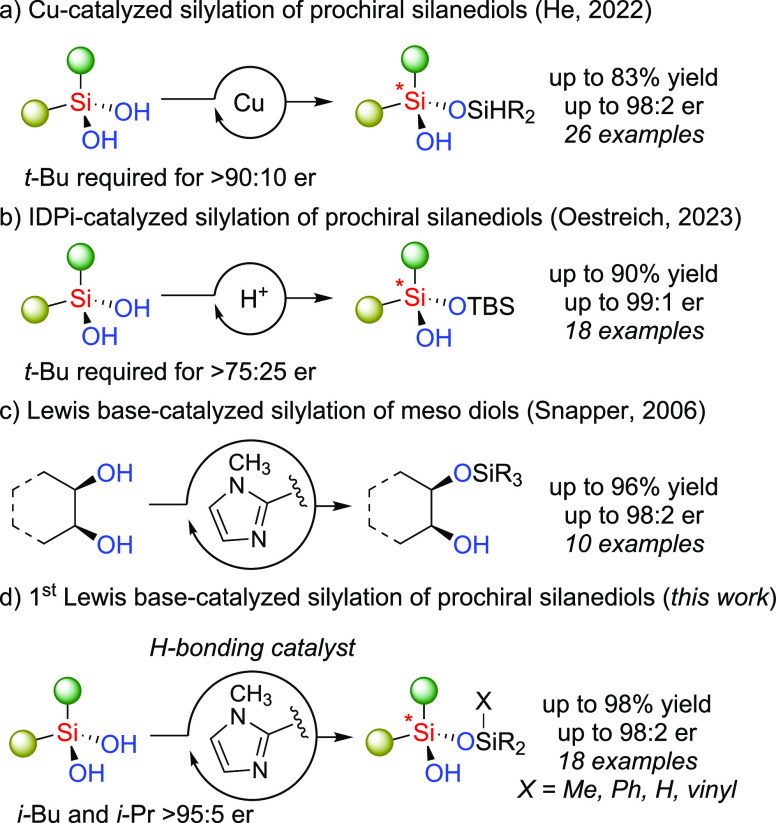
(a) Desymmetrization of prochiral silanediols via Cu-catalyzed SiO-H insertion; (b) desymmetrization of prochiral silanediols via Bronstead acid catalysis; (c) desymmetrization of meso-diols via Lewis base-catalyzed silylation; and (d) desymmetrization of prochiral silanediols via Lewis base-catalyzed silylation.

Here, we report a novel organocatalytic asymmetric synthesis of Si-stereogenic siloxanols upon the desymmetrization of prochiral silanediols (Figure 1d). Based on our expertise with silanediols,^39−41^ we posited that enantioenriched Si-stereogenic compounds could be accessed upon selective silylation of one enantiotopic hydroxy group. Due to the rarity of precedent for asymmetric silanediol transformations, we initially sought inspiration from the organocatalytic silylative desymmetrization of meso 1,2- and 1,3-diols such as a chiral imidazole-containing catalyst reported by Hoveyda and Snapper (Figure 1c).^32−35^

From our initial evaluation of a series of nonenzymatic organocatalysts (Figure 2), chiral imidazole **4a** was one of the only leads identified for the enantioselective desymmetrization of prochiral silanediols with readily available chlorosilanes.^32^ Our initial investigations considered the silylation of silanediols **1a** and **1b** with unoptimized conditions, e.g., stoichiometric quantities of **4a** (see SI, Table S1). Initial investigations with hydrido-chlorosilane **2a** led to challenges with product isolation under unoptimized conditions, so chlorosilane **2b** and silanediol **1b** were employed for optimization studies. Preliminary results produced siloxanol **3bb** in 65% yield with 80:20 er (Table 1, entry 1). Notably, no bis-silylation product (via infrared) was observed under these conditions. Imidazole catalyst **4a** also has the ability to be quantitatively recycled using a simple acid–base extraction sequence without loss of catalytic efficiency.^32^

**Figure 2:**
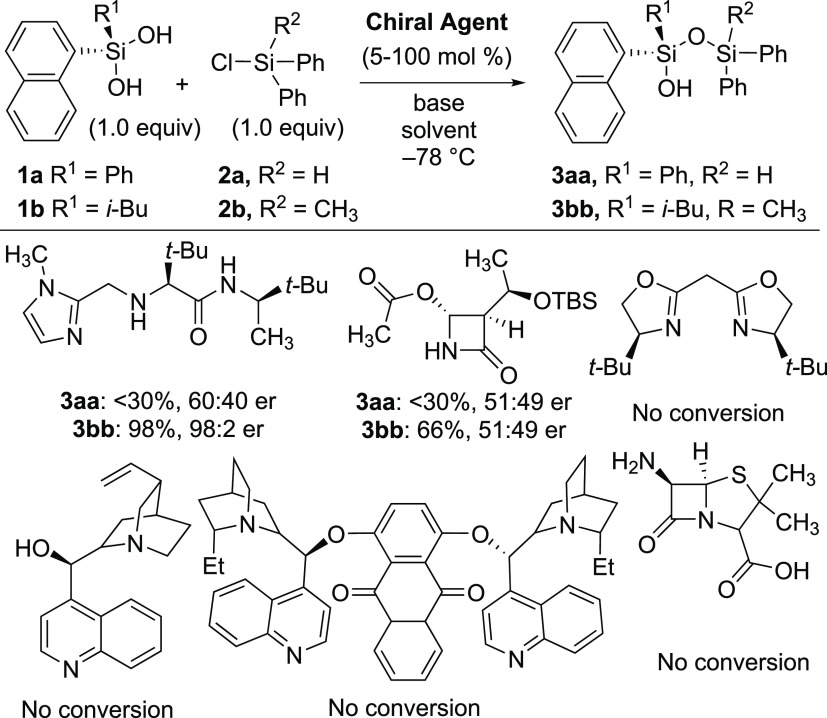
Preliminary screening of nonenzymatic catalysts for silylative desymmetrization (See the SI for full details).

**Table 1:**
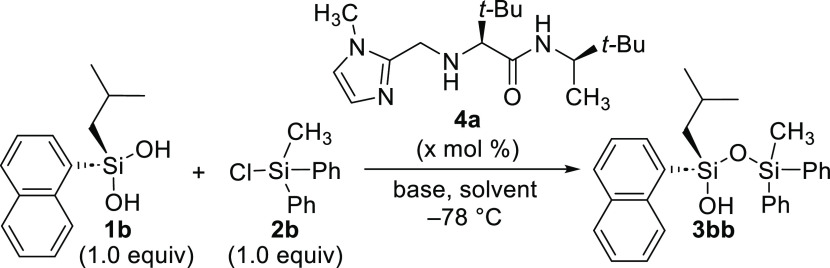
Optimization of Silanediol Desymmetrization Using **4a**

entry	solvent	4a^a^ (%)	time (h)	base	yield^b^ (%)	er^c^
1	THF	100	3	*i-*Pr_2_NEt	65	80:20
2	PhMe	100	3	*i-*Pr_2_NEt	93	95:5
3	PhMe	20	16	*i-*Pr_2_NEt	95	93:7
4	PhMe	20	16	Et_3_N	99	81:19
5	PhMe	20	16	DBU	41^d^	65:35
6	PhMe	20	16	Cs_2_CO_3_	7^e^	85:15
7	PhMe	20	16	Pinene^f^	42^g^	82:18
8	PhMe	20	16	Et_3_N:Pinene^f^	73	90:10
9	PhMe	20	16	*i-*Pr_2_NEt:Pinene^f^	91	99:1
10	PhMe	20	4	*i-*^*i*^Pr_2_NEt:Pinene^f^	73	95:5
11	PhCl	20	16	*i-*Pr_2_NEt:Pinene^f^	95	88:12
12^e^	CH_2_Cl_2_	20	16	*i-*Pr_2_NEt:Pinene^f^	90	73:17
13^e^	PhMe	5	16	*i-*Pr_2_NEt:Pinene^f^	98	98:2

a Catalyst quantitatively recycled after each optimization trial.

b Isolated yield after column chromatography.

c Determined using HPLC with Diacel CHIRALPAK AD-H column.

d Formation of double silylated (trisiloxane) product is observed under these conditions, which accounts for the lower yield.

e Low yield due to poor solubility of Cs_2_CO_3_ in PhMe; remaining mass recovered is **1b**.

f Pinene = ±α-Pinene, used in 1:1 ratio with base, if indicated.

g Remaining mass recovered is **1b**. The absolute configuration of **3bb** was determined to be (*S*) based on analogy to **3aa** and **3ga**, based on comparison to known compounds (See the SI)

We proceeded to optimize conditions for the organocatalytic desymmetrization using silanediol **1b** with chlorosilane **2b**, where the choice of solvent and control of exogenous acid proved to be key factors in achieving high yield and enantioselectivity (Table 1). Switching from THF to toluene improved yields of **3bb** to 93% and enantioselectivity to 95:5 (entry 1 vs 2). Catalyst loading at 20 mol % maintains high yield and enantioselectivity, albeit with a longer reaction time (16 h vs 3h, entry 3). Using Et_3_N in place of DIPEA also affords an excellent yield, albeit with reduced enantioselectivity (entry 4). Using DBU afforded **3bb** with low enantioselectivity (entry 5) and low yield due to the formation of trisiloxane **5** (isolated), which we had not observed under other silylation conditions. Using an inorganic base such as Cs_2_CO_3_ was ineffective for the transformation due to poor solubility, and significant unreacted starting material was observed (entry 6). When α-pinene was investigated as a scavenger to manage endogenous acid (entries 7–9),^42−46^ the best overall combination of yield and enantioselectivity was observed using a 1:1 of DIPEA/pinene (entry 9). Reducing the reaction time to 4 h led to a corresponding decreased yield, confirming that 8–16 h is generally required (entry 10). Comparing DCM and chlorobenzene solvents retains similar product yields but reduces the enantioselectivity (entries 12–13). The yield and enantioselectivity remain high using a 5 mol % catalyst loading of **4a** (entry 13), and these conditions were used as the optimized conditions. It is notable that the organocatalyst can be quantitively recycled after each reaction, a practice employed for reaction optimization and substrate scope experiments.

Due to the thermodynamic favorability of Si–O bond formation,^47−49^ we were aware that bis-silylation may also proceed to afford achiral trisiloxane **5** during the desymmetrization process. Since the formation of the achiral trisiloxane would reduce yields of the desired enantioenriched product, care was taken to identify any byproducts during the desymmetrization. Under optimized conditions, only a single silylation of the silanediol was observed, and siloxanol **3** appears inert to further siloxane formation (Scheme 1).

**Scheme 1:**
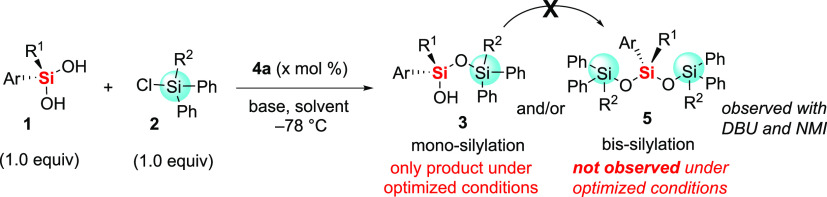
Bis-Silylation Not Observed during Desymmetrization

Exploring the scope of silanediols with diphenylmethylchlorosilane (**2b**) demonstrated that silanediols bearing mixed aryl-alkyl substituents generally offer the best combination of reactivity and stability (Table 2). Siloxanol **3bb** was synthesized on a gram scale in 98% yield with 98:2 er (Table 2). 4-Fluoro-1-naphthyl **1c** was silylated to afford **3cb** in 56% yield and 92:8 er, with the reduced yield attributed to product instability on silica gel. Using a 2-naphthyl (2-Np), o-tolyl, or phenyl instead of 1-naphthyl (1-Np) shows that the enantioselectivity is sensitive to the steric interactions of the aryl group while maintaining a generally consistent 86:14 er. With diarylsilanediol **1a**, the two aryl substituents reduce the ability of the catalyst to effectively discriminate between the two OH of the prochiral silanediol, affording **3ab** with only moderate enantioselectivity (83%, 70:30 er). Replacement of the branched *i-*butyl with linear *n*-butyl still proceeds with good enantioselectivity (83:17 er). Inclusion of electron-donating 4-MeOPh with the *n*-butyl group (**1i**) degrades the enantioenrichment to 77:23. Notably, the use of a *tert*-butyl-substituted silanediol, which has been previously reported,^29,30^ is not tolerated and does not proceed with selectivity: the silylation of **1j** is either not selective or unreactive, depending on chlorosilane pairing, which is attributed to steric bulk disrupting silanediol H-bonding to the organocatalyst (vide infra).

**Table 2:**
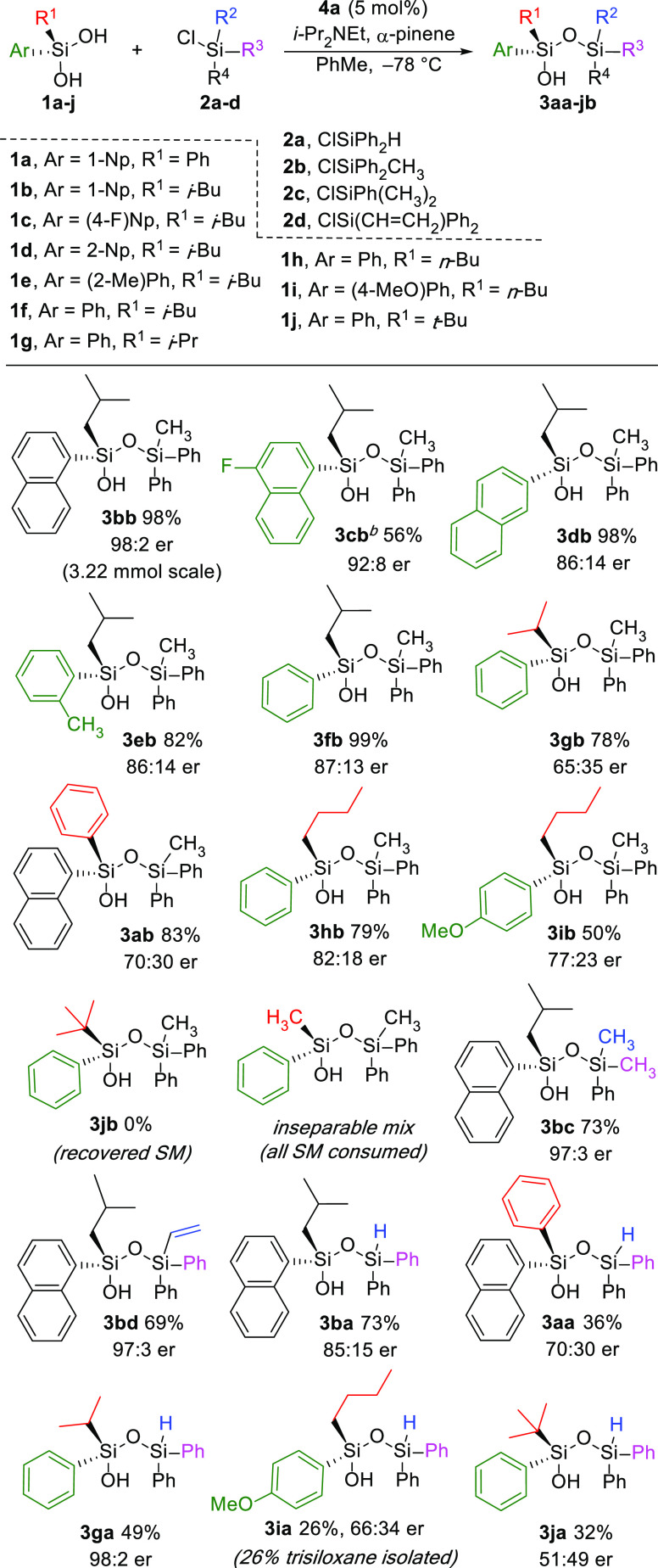
Substrate Scope^a^^,^^b^

a Isolated yields are after column chromatography; catalyst quantitatively recycled after synthesis of each substrate. Enantiomeric ratio determined using HPLC with a Diacel CHIRALPAK AD-H, AS-H, or OD-H column. **3bb** Performed on a gram scale; all other reactions conducted on a 0.15 mmol scale.

b High conversion observed; lower isolated yield attributed to the instability of **3cb** on silica gel.

Next, the scope of the chlorosilane was investigated to include vinyl and hydrido functional handles on silicon (Table 2). Silylation of **1b** using dimethylphenylchlorosilane **2c** maintains a high enantioselectivity (97:3 er) for the formation of siloxanol **3bc**. Pairing vinylchlorosilane **2d** with **1b** maintains high enantioselectivity (97:3 er) for the synthesis of vinylsiloxanol **3bd**. Now using optimized conditions, we also demonstrated the utility of hydridochlorsilane **2a** to isolate hydridosiloxanols under these conditions. Silyation of mixed aryl-alkyl silanediols again afforded the highest selectivity when using **2a**, where selective silylation with similar diaryl substituents is more challenging. Notably, when using **2a**, the desymmetrization of the isopropyl substrate **1g** produced **3ga** in excellent 98:2 enantioselectivity with the SiH functional handle. Desymmetrization of the *tert*-butyl silanediol **1j** proceeds with low yield and selectivity, which further highlights the opportunities for isobutyl and isopropyl in this methodology. When **2a** was used in conjunction with either **1a** or **1b**, selectivity was reduced (70:30 and 85:15, respectively). In the case of n-butyl, product **3ia**, a low selectivity and low yield are observed, with an equimolar quantity of the trisiloxane byproduct also isolated. Trialkylsilyl chlorides without the aryl group proved unreactive for the transformation while using Ph_3_SiCl gave poor yield and selectivity (see SI Figure S4).

Imidazole rings are known to be potent Lewis base catalysts for silylation of alcohols,^50−52^ and **4a** was designed to function as a chiral Lewis base activator of chlorosilanes during the desymmetrization of meso-diols.^32^ Therefore, it is reasonable to expect Lewis base activation of **2** by **4a** for the silylation of silanediol **1**. ^29^Si NMR spectra of the equimolar mixture of **2b** with either **4a** or NMI at room temperature in C_6_D_6_ showed a strong ^29^Si resonance appearing at −9.6 ppm accompanied by the disappearance of the **2b** resonance at 10.2 ppm (Figure 3). The observed shift in the ^29^Si resonance supports the activation of **2b** by **4a**, similar to previous literature reports of chlorosilane activation by N-containing heterocycles.^51−53^

**Figure 3:**
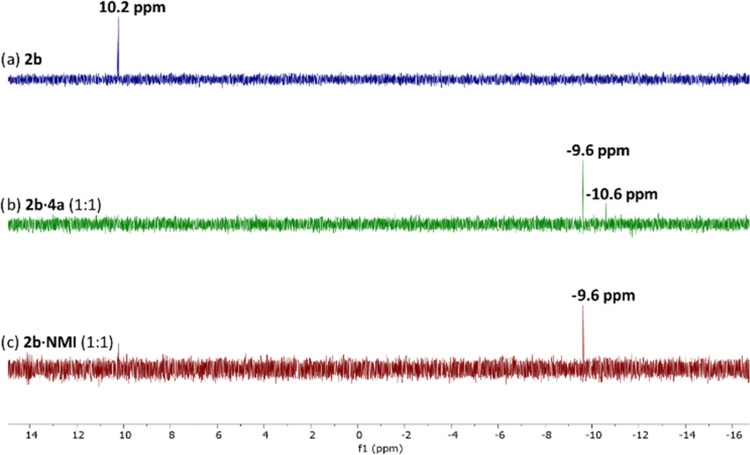
^29^Si NMR spectra for (a) **2b**, (b) **2b** with 1 equiv of **4a**, and (c) **2b** with 1 equiv of NMI. All NMR spectra were taken in C_6_D_6_ (0.075 M) at 23 °C.

The role of the organocatalyst as a Lewis base during silylation was further evaluated by synthesizing and testing analogues that lacked the imidazole component (Figure 4a). When (*R*)-2-(benzylamino)-*N*-((*R*)-3,3-dimethylbutan-2-yl)-3,3-dimethylbutanamide (**4b**) was evaluated for the desymmetrization of **1b** with **2b**, no reaction was observed, resulting in quantitative recovery of starting material. We next turned to evaluate (*R*)-2-(pyridyl)-*N*-((*R*)-3,3-dimethylbutan-2-yl)-3,3-dimethylbutanamide (**4c**) since heterocycles containing a pyridine have been shown to be active catalysts in silylation.^51,54^ Pyridine analogue **4c** also did not catalyze silylation of the silanediol, affording only recovered starting material. According to prior reports of **4a** for the silylation of meso-diols, including an achiral Lewis base cocatalyst was also observed to improve reaction times without negative impacts on enantioselectivity.^35^ When achiral Lewis bases such as *N*-methyl imidazole (NMI) and 5-(ethylthio)-1H-tetrazole (**6**) were investigated as cocatalysts, no rate acceleration was observed. The addition of NMI reduced yield and enantioselectivity, suggesting that NMI can act as a competitor to **4a** rather than as a cocatalyst (Figure 4b). The addition of **6** afforded a reduced yield, but no change in enantioselectivity was observed.^35^

**Figure 4:**
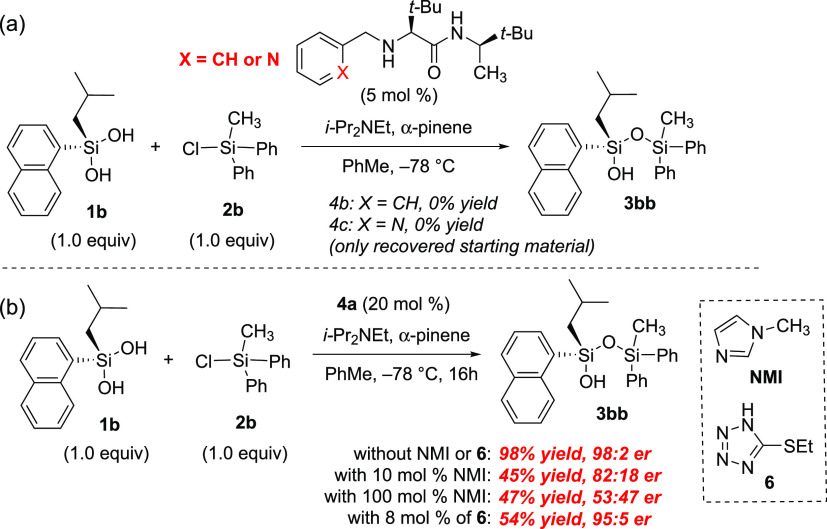
(a) Replacing the imidazole ring of organocatalyst **4a** with a phenyl or pyridyl group results in loss of catalyst activity. (b) Adding NMI as a cocatalyst; adding **6** as a cocatalyst.

Both ^1^H NMR and X-ray cocrystallization experiments highlight the H-bonding interactions between the silanediol and organocatalyst. In an enantiopure cocrystal of silanediol **1a** and organocatalyst **4a** (Figure 5a), **4a** adopts a dual binding mode with a single hydroxy group of **1a**, generating a chiral environment to promote enantioselective desymmetrization. This structure highlights the imidazole function as an H-bond acceptor for SiOH (O-NH 1.74 Å), while demonstrating the amide operating as an H-bond donor to the oxygen of the silanol (NH-O 2.14 Å). ^1^H NMR binding studies with **4a** also confirm the hydrogen-bonding interactions with the silanediol in solution, showing shifts for both the amide and imidazole signals (Figure 5b). Upon mixing **4a** and **1b** in deuterated benzene, the amide proton signal exhibits a downfield shift (Δ**δ**^NH^ = 0.37 ppm), and both protons of the imidazole shift upfield (Δ**δ**H^a^ = 0.55 ppm and Δ**δ**H^b^ = 0.42), consistent with a H-bonding interaction at both positions. The binding affinity (*K*_a_) was calculated according to the shift of imidazole peak H^b^ to be *K*_a_^Hb^ = 191 ± 25.^55−60^ An NMR binding study of **4a** with **1a** confirmed a lower binding affinity (*K*_a_ = 90 ± 3; see SI, Figure S3), which was expected since this substrate (**1a**) afforded reduced enantioselectivity in the desymmetrization reaction.

**Figure 5:**
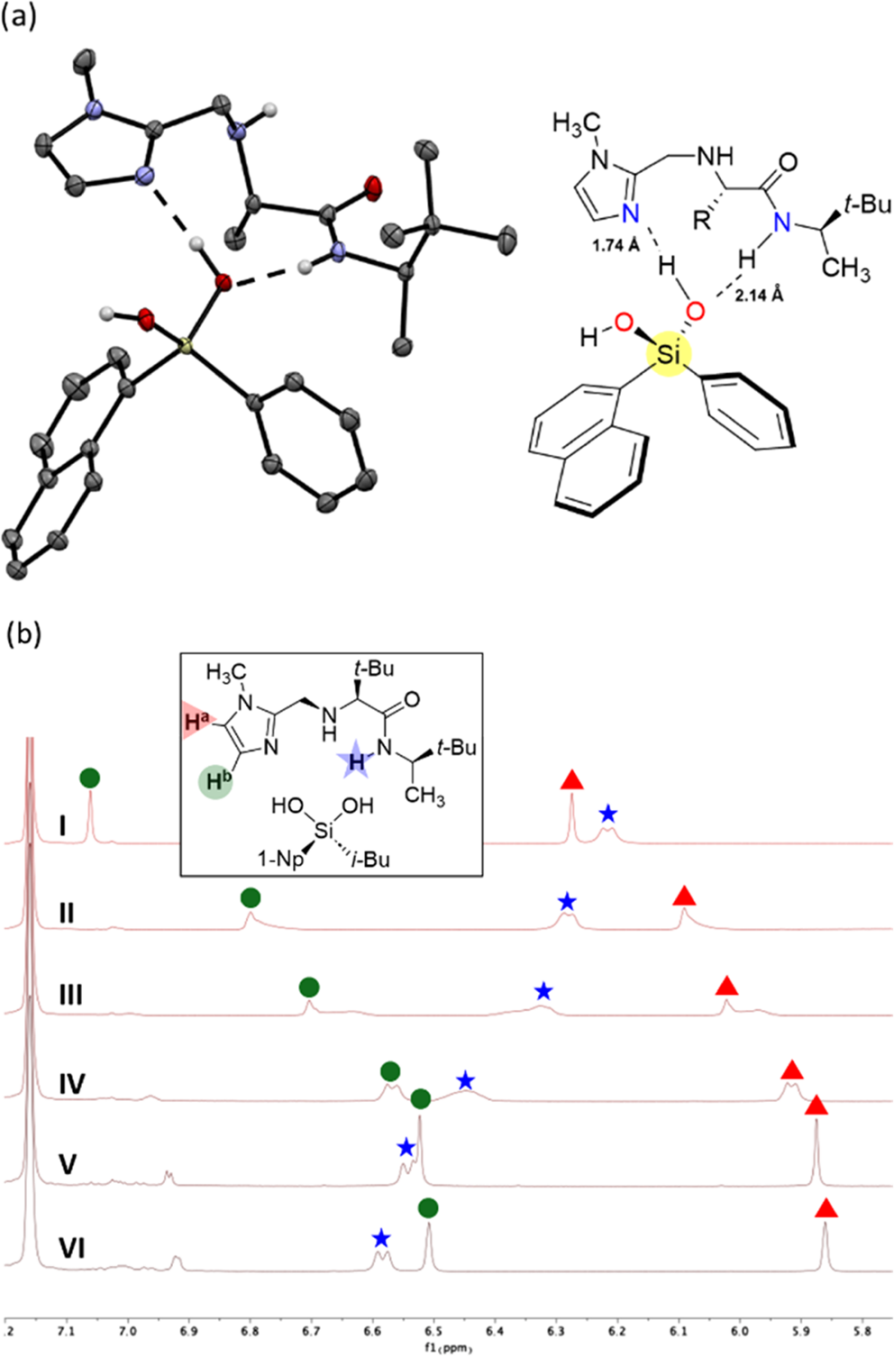
(a) X-ray crystal structure of **1a** bound with **4a** (R = *t*-Bu). Hydrogen atoms and one *t*-butyl group have been omitted for clarity. Hydrogen bond lengths are shown for N(imidazole)-HOSi 1.74 Å and N–H(amide)-OSi 2.14 Å. (b) ^1^H NMR binding data of silanediol **1b** with catalyst **4a**. I = 0 equiv **1b**, II = 1 equiv **1b**, III = 3 equiv **1b**, and IV = 5 equiv **1b**. V = 7 equiv **1b**. VI = 10 equiv **1b**. All ^1^H NMR spectra were collected at 0.02 M **4a** in C_6_D_6_ at room temperature.

To elucidate the interplay between catalyst and silanediol binding, additional binding studies were conducted with **1b**, **1e**, and **1j**, where it was determined that high *K*_a_ values correlate with high levels of enantioenrichment (Table 3). Comparing the binding of silanediol **1a** revealed that modest binding can produce moderate enantioenrichment, while silanediol **1b** forms the strongest association to **4a** and produces high enantioenrichment. Silanediols **1e** and **1j** produce signal shifts of the same magnitude, yet due to the rate of shifting, **1e** has a greater *K*_a_. To visualize this trend, product ee was graphed as a function of the silanediol binding affinity (*K*_a_) with **4a** (Figure 6). The graph shows a strong correlation between high *K*_a_ and enantioenrichment (*R*^2^ = 0.997). From this relationship, we conclude that the main factor dictating the enantioenrichment of siloxanol products is the ability of the parent silanediol to form a strong H-bonding complex with **4a**. Additional binding experiments between silanediol **1b** and benzyl analogue **4b** (see SI, Figure S8) also support that the imidazole is necessary for a strong H-bonding complex for desymmetrization. The performance of the varying substrates, alongside the results of the X-ray and NMR binding studies, overall supports that the selectivity of the process is dependent on the binding and steric interactions between the silanediol and **4a**, as well as the chlorosilane.

**Figure 6:**
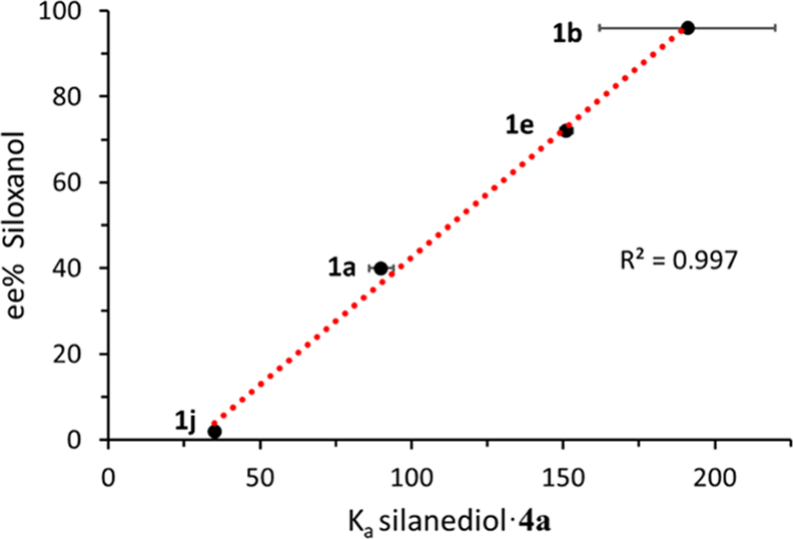
Preliminary correlation of ee% of siloxanol product to silanediol-catalyst binding affinity (*K*_a_).

**Table 3:** Binding Data (*K*_a_) of Silanediols with **4a**

silanediol	Δδ (ppm)^a^	K_a_ (M^–1^)^b^	ee
**1a**	–0.43	90 ± 3	40
**1b**	–0.41	191 ± 25	96
**1e**	–0.23	151 ± 2	72
**1j**	–0.23	35 ± 1	2

a Δδ (ppm) for silanediols **1a**, **1b**, **1e**, and **1j** calculated for shift of imidazole H_b_.

b Error was calculated as 95% confidence intervals based on the fit of the NMR titration data to the model.

Our experimental evidence suggests that a single molecule of **4a** fulfills the dual catalytic role for H-bonding molecular recognition of the prochiral silanediol and Lewis base activator of the chlorosilane. In this case, the ^1^H NMR and X-ray study findings provide evidence for the formation of the **4a**·**1** complex, while ^29^Si NMR reveals an interaction between **4a** and **2** that is similar to previous reports of the formation of pentacoordinate halosilane-Lewis base adducts.^52,61,62^ However, experiments with several Lewis base additives did not demonstrate cocatalysis and afforded decreased yield and/or selectivity, which supports that only one molecule of the organocatalyst is serving a dual catalytic role. This is in contrast to previous reports for meso-diols, where Lewis basic additives and computational studies support that two molecules of **4a** are engaged during desymmetrization.^35^ Our substrate scope provides insight into the asymmetric induction, where enantioselectivity is attributed to the orientation of the bulky groups of organocatalyst **4a** and the alkyl group of silanediol **1** to leave only one angle of approach for silyl chloride **2**. At least one aryl group on **2** is necessary for silylation of unbound OH to proceed. A full mechanistic study is underway to establish a more detailed mechanism for the Lewis base-catalyzed enantioselective silylation of prochiral silandiols in comparison to meso-diols.

In conclusion, this work reports the development of a metal-free synthesis of Si-stereogenic siloxanol compounds from prochiral silanediols with high yields and enantioselectivity while incorporating silane and vinyl functional handles. X-ray and NMR binding studies, and a comparison of catalyst analogues, support that this transformation is catalyzed via a two-point H-bonding interaction of the bifunctional imidazole catalyst with the silanediol. This H-bonding activation method shows scope effective with secondary alkyl groups (i.e., isobutyl and isopropyl), which indicates future potential for diastereoselective applications with chiral carbon and stereogenic silicon. New methods for efficient access to Si-stereogenic compounds containing various functional handles are expected to have important applications for materials, medicinal chemistry, and catalysis.

## Supporting Information Available

Full experimental and characterization data of all compounds, including NMR spectra, HPLC traces, and X-ray crystallography data (PDF)
