# Supplementary material for: Organocatalytic Asymmetric Synthesis of Si-Stereogenic Siloxanols
Source: ACS Catal. 2024 Jan 5;14(2):1005–12. doi: 10.1021/acscatal.3c03932 (PMC10804373; doi:10.1021/acscatal.3c03932)
Supplement: Supplementary file 1 — cs3c03932_si_001.pdf [file cs3c03932_si_001.pdf]

Supporting Information

Organocatalytic Asymmetric Synthesis of Si-Stereogenic Siloxanols

Jacob J. Dalton, Adilene Bernal Sánchez, Austin T. Kelly, James C. Fettinger and Annaliese K. Franz*

Department of Chemistry, University of California, Davis, One Shields Ave, Davis, CA 95616

**E-mail: akfranz@ucdavis.edu*

1. General information 3
1.1 General procedure for the organocatalyzed desymmetrization of silanediols for the synthesis of siloxanols 3aa-hl 4
1.2 General procedure for the synthesis of racemic siloxanols as HPLC standards 5
1.3 Determination of absolute stereochemistry 5
2. Chiral agent screen 6
3. Optimization for silylative desymmetrization of prochiral silanediols 8
4. Reaction scope 9
4.1 Silanediol scope 9
5. Binding and concentration studies using ^{1}H NMR spectroscopy 11
5.1 General procedure for binding and concentration studies[3–7] 11
5.2 Determination of association constant[5,8] 12
5.3 Summary of binding data using ^{1}H NMR spectroscopy 20
6. Lewis base-Chlorosilane ^{29}Si NMR Study 21
7. Product competition studies 22
7.1 General procedure for competition studies 22
8. Acid Scavenger Studies 23
9. Proposed catalytic cycle 25
10. Characterization of reported compounds 26
9.1 Characterization data for siloxanols (3aa-mb) 33
11. X-Ray crystallographic structure and information 62
11.1 X-Ray co-crystal analysis of 1a•4 63
12. References 74
12. Author contributions 75
13. NMR spectra........76

1. General information

All nuclear magnetic resonance (NMR) spectra were obtained on Bruker Nanobay AVIIIHD 400 (400 MHz for ^{1}H; 100 MHz for ^{13}C; 80 MHz for ^{29}Si) and/or 300 MHz Bruker Avance-NEO (300 MHz for ^{1}H) and/or Varian VNMRS 600 (600 MHz for ^{1}H; 150 MHz for ^{13}C; 119 MHz for ^{29}Si) at room temperature unless noted otherwise. Chemicals shifts are reported in parts per million (δ scale) and referenced according to the following standards: tetramethylsilane internal standard for ^{1}H signals in chloroform (δ 0.00), benzene residual solvent (δ 7.16) for ^{1}H signals in benzene, deuterated chloroform or benzene carbon resonances (middle peak is δ 77.1 or δ 128.1, respectively) for $^{13}C\{^{1}H\}$ signals, tetramethylsilane external standard in $CDCl_3$ for $^{29}Si\{^{1}H\}$ signals (δ 0.00). For some ^{29}Si NMR spectra, chromium(III) acetylacetonate was added at 0.01 M as a T1 relaxation agent with relaxation delays set to 5 seconds.[1] Coupling constants are reported in Hertz (Hz) and multiplicities are reported as follows: singlet (s), doublet (d), triplet (t), quartet (q), and multiplet (m). Compounds were analyzed for HRMS on a Thermo Fisher Orbitrap XL (Davis, CA) using electrospray in the positive ion mode and the negative ion mode at >60,000 resolution and using 5 kV spray voltage, with a curtain plate temperature of 275 °C and sheath gas setting of 15. These settings result in mass accuracies <5 ppm. Samples were analyzed via flow injection analysis by injecting 5 µL samples into a stream of 50% acetonitrile and 50% aqueous solution of 0.1% formic acid, flowing at 200 µL/minute. X-ray crystallography was completed using a Bruker APEX-II CCD Diffractometer. High performance liquid chromatography (HPLC) data were obtained on Shimadzu LC-20AB system with CHIRALPAK® AD-H column (4.6 x 250 mm, 5 µm), CHIRALPAK® OD-H column (4.6 x 250 mm, 5 µm) or CHIRALPAK® AS column (4.6 x 250 mm, 5 µm) and Shimadzu SPD-M20A photodiode array detector. Each HPLC sample was eluted at a constant flow rate with an isocratic 98:2 hexanes/isopropanol system, and 40 °C column oven temperature unless otherwise specified. UV/Vis spectra were recorded using Shimadzu SPD-M20A photodiode array detector. Melting points were obtained on a Stanford Research Systems EZ-Melt automated melting point apparatus, all reported melting points are averaged values of 3 trials. Reactions requiring cryogenic temperatures for greater than 3 h were placed in a Nestlab CB 80 Cryocool (cryocool) to maintain the reaction temperature.

Commercially available reagents were purchased and used without further purification unless otherwise indicated. All deuterated solvents were dried using 4Å molecular sieves prior to binding and dilution studies. All chlorosilanes were purchased from Gelest. Bromohalides were purchased and used as is from Spectrum, Alpha Aesar, or Combiblocks. α-Pinene was purchased from Acros and used without further purification. Boc-*tert*-leucine was purchased from Chem Impex, *N*-methyl imidazole was purchased from Alfa Aesar and distilled over CaH_2 before use. Reactions were analyzed by thin-layer chromatography (TLC) on EMD glass plates that were pre-coated with silica gel 60 F254, and the reactions were purified by column chromatography using Acros silica gel 60 Å (0.035-0.070 mm). The following abbreviations are used throughout: ethyl acetate (EtOAc), tetrahydrofuran (THF), toluene (PhMe), dichloromethane (DCM), lithium aluminum hydride (LAH), diisobutylaluminium hydride (DIBAL), 1,8-diazabicyclo[5.4.0]udec-7-ene (DBU), and isopropanol (IPA).

1.1 General procedure for the organocatalyzed desymmetrization of silanediols for the synthesis of siloxanols 3aa-hl

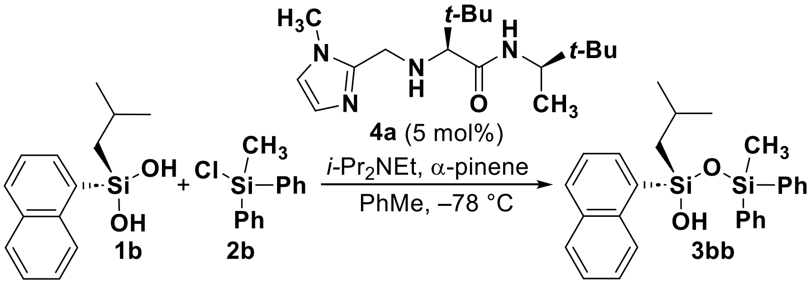

While a glove box was not used for these reactions, care should be taken to ensure the reaction vessel remains air and moisture free to prevent potential undesired condensation products. A 25-mL round bottom flask equipped with a stir bar was flame-dried under high vacuum. After the flask cooled to room temperature under vacuum, it was evacuated then purged three times with argon and fitted with a septa and argon balloon. Then desymmetrization agent **4a** was weighed out and added (5 mol %, 0.0075 mmol, 0.0023 g), followed by silanediol **1b** (1.00 equiv, 0.15 mmol, 0.0369 g), while maintaining a positive flow of argon into the flask. 2.00 mL of toluene was added. The flask was cooled to –78 °C, and iPr_2NEt (1.20 equiv, 0.18 mmol, 31.4 mL, freshly distilled over CaH_2 or from a sure/sealed™ bottle) and α-pinene (1.2 equiv, 0.18 mmol, 28.6 mL) were added via syringe. Finally, chlorosilane **2b** (1.00 equiv, 0.15 mmol, 31.5 mL, bottle fitted with a septa and argon balloon, stored under argon) was added and the reaction was stirred at –78 °C in an acetone dry ice bath for 15 min, then transferred as quickly as possible to a cryocool at –78 °C and left to react for an additional 15 h. Then the reaction mixture was quenched with 10% citric acid aqueous solution (2.0 mL) and extracted with Et_2O (5 mL 3 times), note that Et_2O was specifically used because other solvents generally gave poorer yields. The combined organics were washed with brine, dried over Na_2SO_4 and concentrated in vacuo. The product was directly purified using column chromatography on silica gel (9:1 hexanes/EtOAc), the siloxanol product was characterized after purification. TLC analysis of the crude reaction mixture was sufficient to confirm product formation and SM consumption, therefore crude NMR was not taken. HPLC analysis was performed after purification.

1.2 General procedure for the synthesis of racemic siloxanols as HPLC standards

The general procedure for the organocatalyzed desymmetrization was followed except the chiral desymmetrization agent **4a** was omitted and freshly distilled 1-methylimidazole (NMI) (1.00 equiv) was substituted in place of **4a**.When NMI was used in place of 4a yields were generally low and multiple undesired side products were formed, including the trisiloxane. These side products could be difficult or impossible to separate from the desired racemic product, therefore in some cases a 1:3 mixture of 4a/NMI was used which resulted in racemic or scalemic products in higher yield and without the undesired side products.

1.3 Determination of absolute stereochemistry

The S configuration for the siloxanol product is assigned to the chiral Si atom after HPLC traces compared to previously reported traces for compound **3aa** as well as compound **3ga.**[2]

2. Chiral agent screen

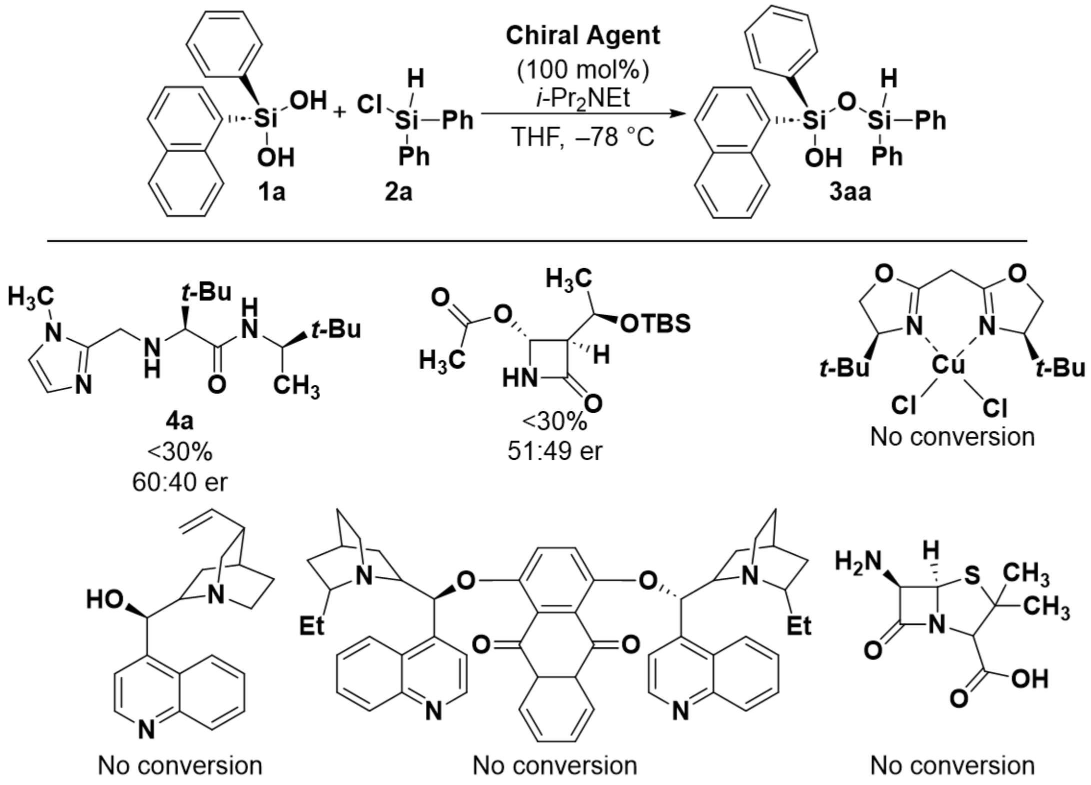

Figure S1. Preliminary screening of non-enzymatic chiral agents using 100 mol % chiral agent. Reactions were performed under argon at 0.1 mmol scale, 0.075 M concentration in THF, with 1.2 equivalents of *i*-Pr_2NEt used. Reactions were evaluated using TLC and ^1H NMR spectroscopy. No conversion indicates that no product was detected by TLC.

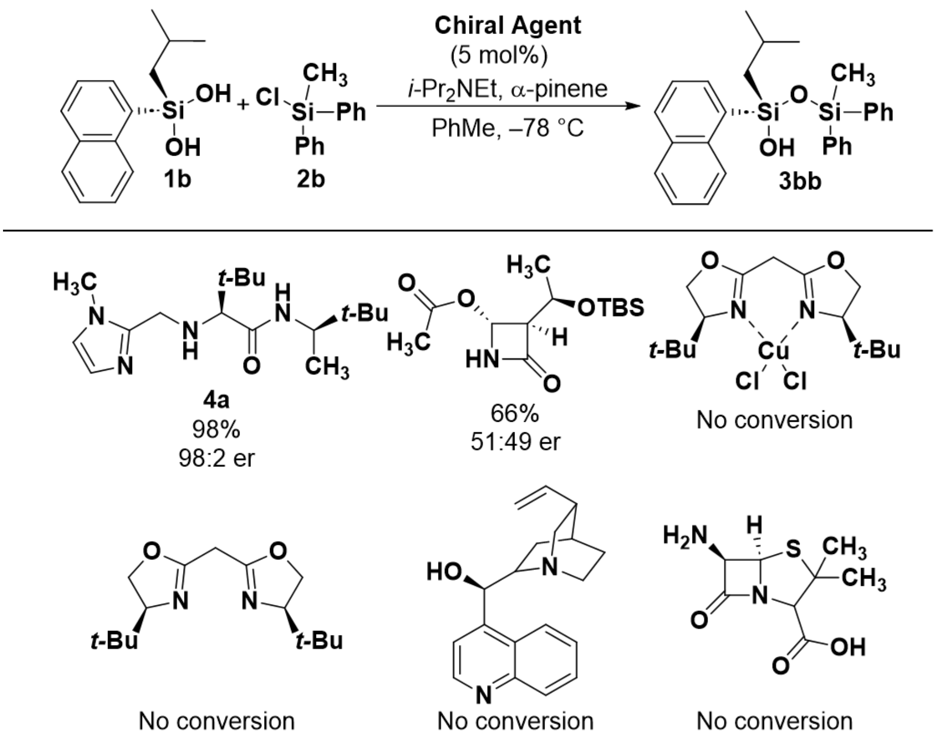

Figure S2. Repeat screening of non-enzymatic chiral agents at 5 mol % using optimized conditions, under argon at 0.15 mmol scale, 0.075 M concentration in PhMe with 1.2 equivalents of *i*-Pr_2NEt and 1.2 equivalents (+)-α-pinene No conversion indicates that no product was detected by ^{1}H NMR spectroscopy or TLC.

3. Optimization for silylative desymmetrization of prochiral silanediols

Table S1. Reaction Optimization

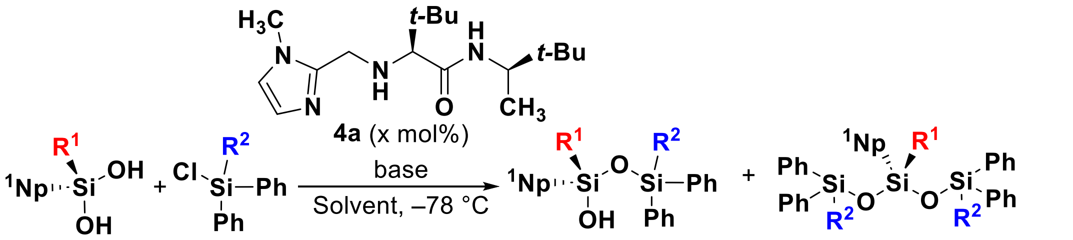

entry	compound	R[1]	R[2]	solvent	**4a**[a](mol%)	time (h)	base	yield[c] (%)	er[d]
1[b]	**3aa**	Ph	H	THF	100	0.17	^{i}Pr$_2$NEt	30[b]	68:32
2	**3aa**	Ph	H	THF	100	0.17	^{i}Pr$_2$NEt	nd	nd
3	**3ab**	Ph	CH_3	THF	100	3	^{i}Pr$_2$NEt	nd	nd
4	**3ba**	*i*-Bu	H	THF	100	0.17	^{i}Pr$_2$NEt	nd	nd
5	**3bb**	*i*-Bu	CH_3	THF	100	3	^{i}Pr$_2$NEt	65	80:20
6	**3bb**	*i*-Bu	CH_3	PhMe	100	3	^{i}Pr$_2$NEt	93	95:5
7	**3bb**	*i*-Bu	CH_3	PhMe	20	16	^{i}Pr$_2$NEt	95	93:7
8	**3bb**	*i*-Bu	CH_3	PhMe	20	16	Et_3N	99	81:19
9	**3bb**	*i*-Bu	CH_3	PhMe	20	16	DBU[e]	41	65:35
10	**3bb**	*i*-Bu	CH_3	PhMe	20	16	Cs_2CO_3[f]	7	92:8
11	**3bb**	*i*-Bu	CH_3	PhMe	20	16	Et_3N[g]	81	79:21
12	**3bb**	*i*-Bu	CH_3	PhMe	20	16	^{i}Pr$_2$NEt[g]	99	92:8
13	**3bb**	*i*-Bu	CH_3	PhMe	20	16	Proton Sponge	77	92:8
14	**3bb**	*i*-Bu	CH_3	PhMe	20	16	α-pinene[h]	42	82:18
15	**3bb**	*i*-Bu	CH_3	PhMe	20	16	^{i}Pr$_2$NEt:α-pinene	91	99:1
16	**3bb**	*i*-Bu	CH_3	PhCl	20	16	^{i}Pr$_2$NEt:α-pinene	95	88:12
17	**3bb**	*i*-Bu	CH_3	CH_2Cl_2	20	16	^{i}Pr$_2$NEt:α-pinene	90	73:17
18	**3bb**	*i*-Bu	CH_3	PhMe	20	4	^{i}Pr$_2$NEt:α-pinene	73	95:5
19	**3bb**	*i*-Bu	CH_3	PhMe	5	4	^{i}Pr$_2$NEt:α-pinene	57	94:6
20	**3bb**	*i*-Bu	CH_3	PhMe	5	16	^{i}Pr$_2$NEt:α-pinene	98	98:2
21[i]	**3bb**	*i*-Bu	CH_3	PhMe	100	16	^{i}Pr$_2$NEt:α-pinene	37[j]	n/a

Unless otherwise noted all reactions were performed on 0.15 mmol scale, at 0.075 M concentration, 1.0 equiv of chlorosilane, and 1.2 equiv of base. [a]Catalyst quantitatively recycled after each optimization trial. [b]Initial lead, yield estimated using TLC and NMR spectroscopy. [c]Isolated yield after column, nd; not determined; a complex reaction mixture was found with no desired product observed. [d]Determined using HPLC with Diacel CHIRALPAK® AD-H column. [e]Bis-silylated trisiloxane formed in 22% isolated yield under these conditions. [f]Low yield due to insolublility of $CsCO_3$ in PhMe, starting material recovered. [g]3.0 equivalents of base used. [h]Remaining mass recovered as unreacted starting material. [i]Reaction performed using 1-methylimidazole (NMI) in place of **4a** to compare reactivity and obtain racemic HPLC sample. [j]43% yield of Ph2MeSiOH. Significant quantities of inseparable siloxane products observed.

4. Reaction scope

4.1 Silanediol scope

Figure S3. Silanediol Scope

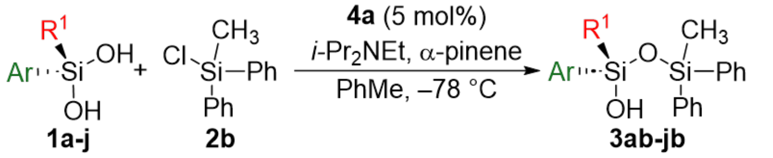

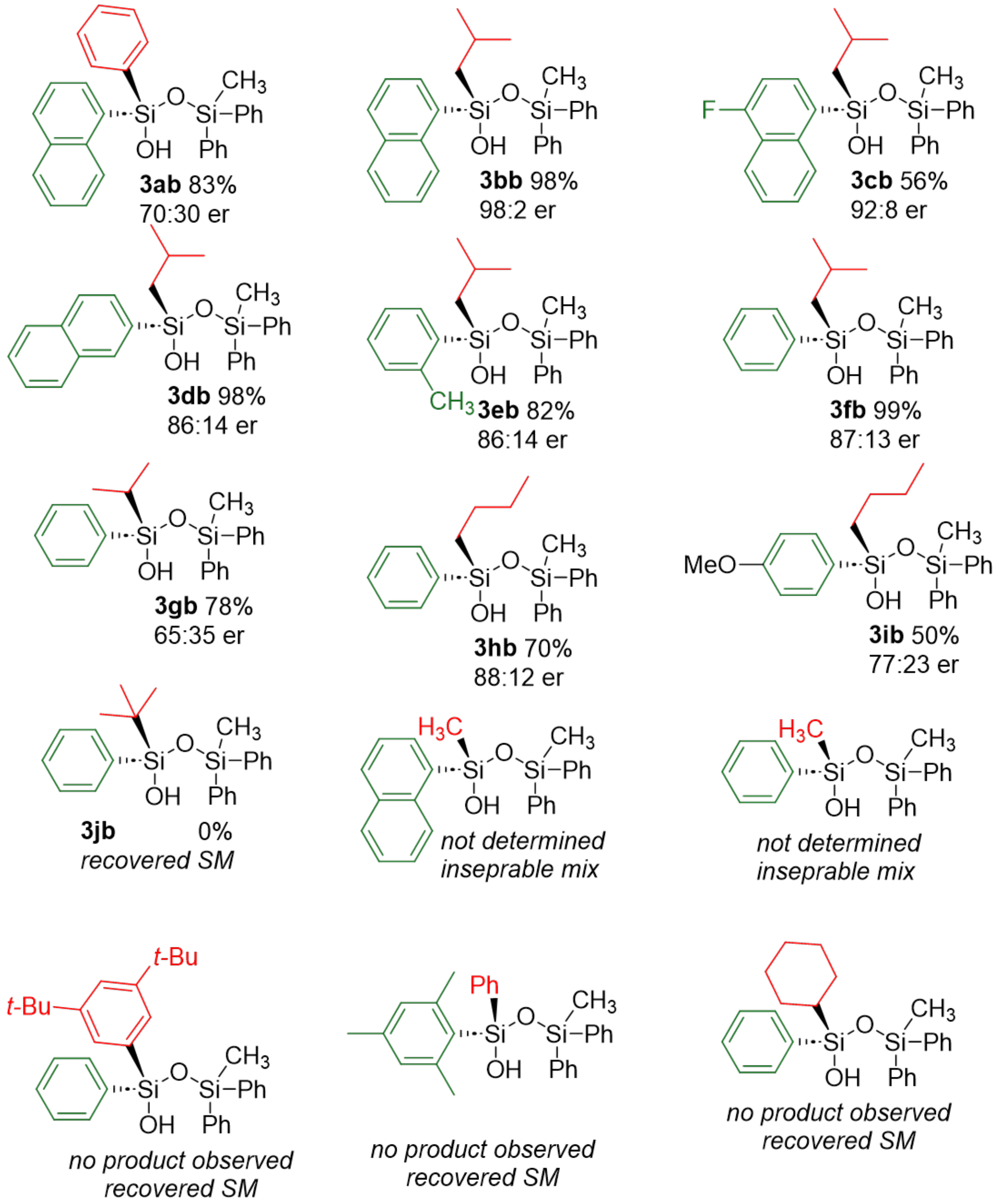

4.2 Chlorosilane scope

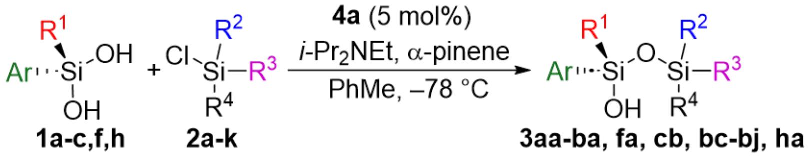

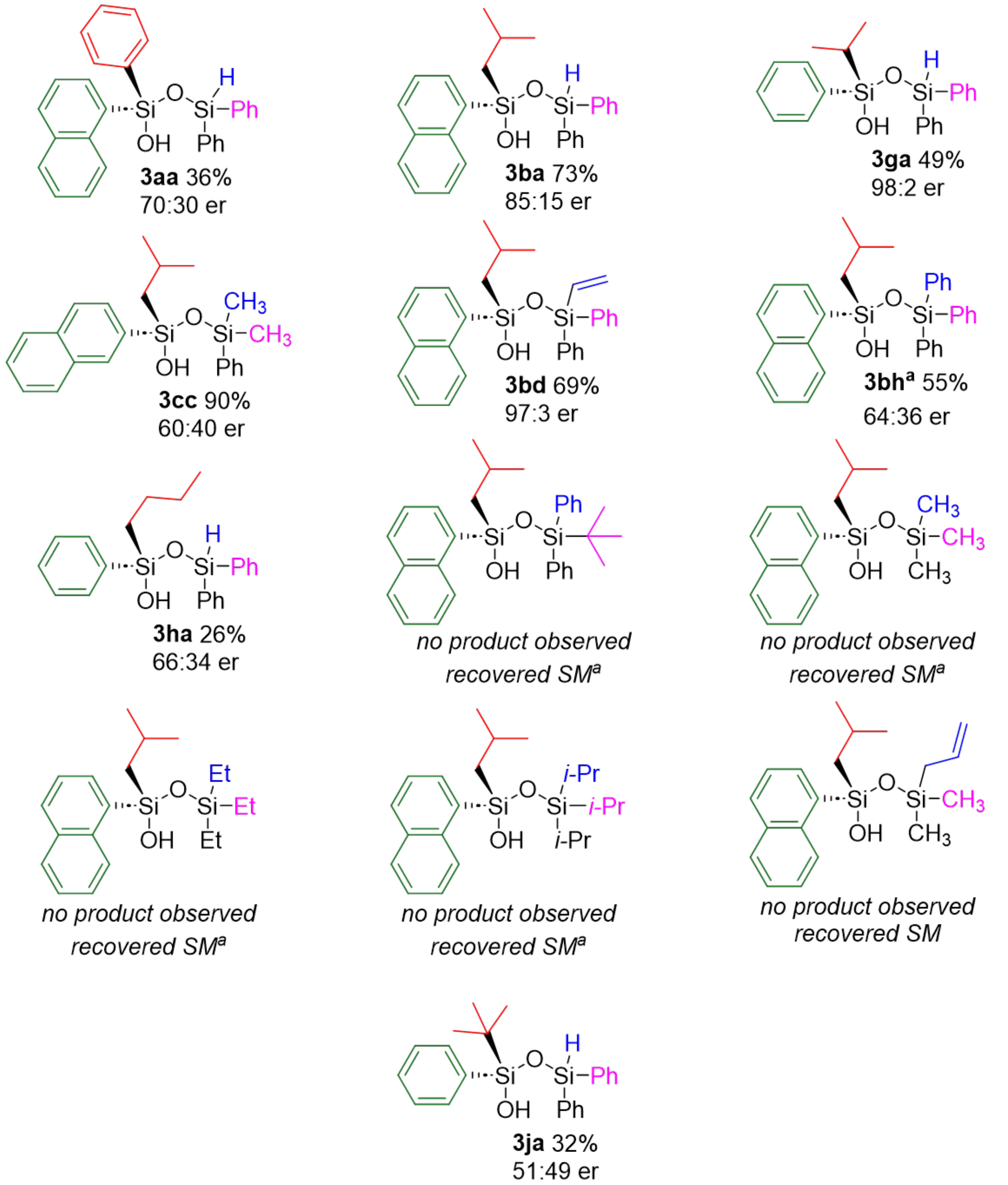

Figure S4. Chlorosilane Scope. [a]Performed with 100 mol % of **4a.**

5. Binding and concentration studies using ^{1}H NMR spectroscopy

Calculated association constants (K_a) for binding of silanediol **1b** were used to compare H-bonding affinities between catalyst structures **4a-c**. We investigated the effect of concentration on the affinity to bind with silanediol **1b**, to understand more about solution-phase H-bonding conformations. Association constants were also calculated with chlorosilane **2b** to probe potential interactions between catalyst **4a** and chlorosilane electrophiles. Association constants were also compared for organocatalyst **4a** and silanediols **1a, 1b**, **1e**, and **1j** to identify if there is a correlation between the enantioselectivity and binding constants using ^{1}H NMR spectroscopy.

5.1 General procedure for binding and concentration studies[3–7]

A stock solution was made by dissolving **4a** (0.08 mmol) in 4.0 mL of C_6D_6. The solution was split into two equal parts and labeled A and B. Vial A was left as is, containing a solution of **4a**. Vial B was charged with 10 equivalents (0.8 mmol) of the guest (silanediol **1a-b**,**1e**, **1j**, or chlorosilane **2b**). After the stock solutions were prepared, six oven dried NMR tubes were purged with argon and fitted with septa caps. The NMR tubes were then filled with different volumes of each stock solution to give the desired equivalents of guest (Table S2). The ^{1}H NMR spectrum of each solution was recorded after 16 scans at room temperature on Bruker Nanobay AVIIIHD 400 or Varian VNMRS 600

Table S2. Contents of NMR Tubes for Binding Studies

NMR tube number	equivalents guest	solution A volume (mL)	solution B volume (mL)
1	0	0.50	0.00
2	1	0.45	0.05
3	3	0.35	0.15
4	5	0.25	0.25
5	7	0.15	0.35
6	10	0	0.50

5.2 Determination of association constant[5,8]

Binding constants were calculated using Microsoft Excel Solver to fit a nonlinear curve with the model equation below.[4,6] K_a was the only variable optimized and no constraints were used. Error was calculated as 95% confidence intervals based on the fit of the NMR titration data to the model.

$$\Delta\delta_i = \delta_{obs,i} - \delta_{free} = \frac{C}{R_0}\Delta\delta \quad \text{(Eq. S1)}$$

$$C = \frac{(K_aR_0+1+K_aS_i)\pm\sqrt{(K_aR_0+1+K_aS_i)^2-4K_a^2R_0S_i}}{2K_a} \quad \text{(Eq. S2)}$$

$\Delta\delta = \delta_{complex} - \delta_{free}$; $\delta_{complex}$ = chemical shift (ppm) of the proton in the bound Lewis base complex, estimated from extrapolation of where the binding curve levels off, δ_{free} = chemical shift (ppm) of the C-H^{imid} proton when no guest is present; $\delta_{obs,\,i}$ = chemical shift of C-H^{imid} proton at *i* equivalents of guest. R_0 = initial concentration of the amide and S_i = concentration of guest at *i* equivalents in M, K_a = binding constant in M^{-1}.

A.

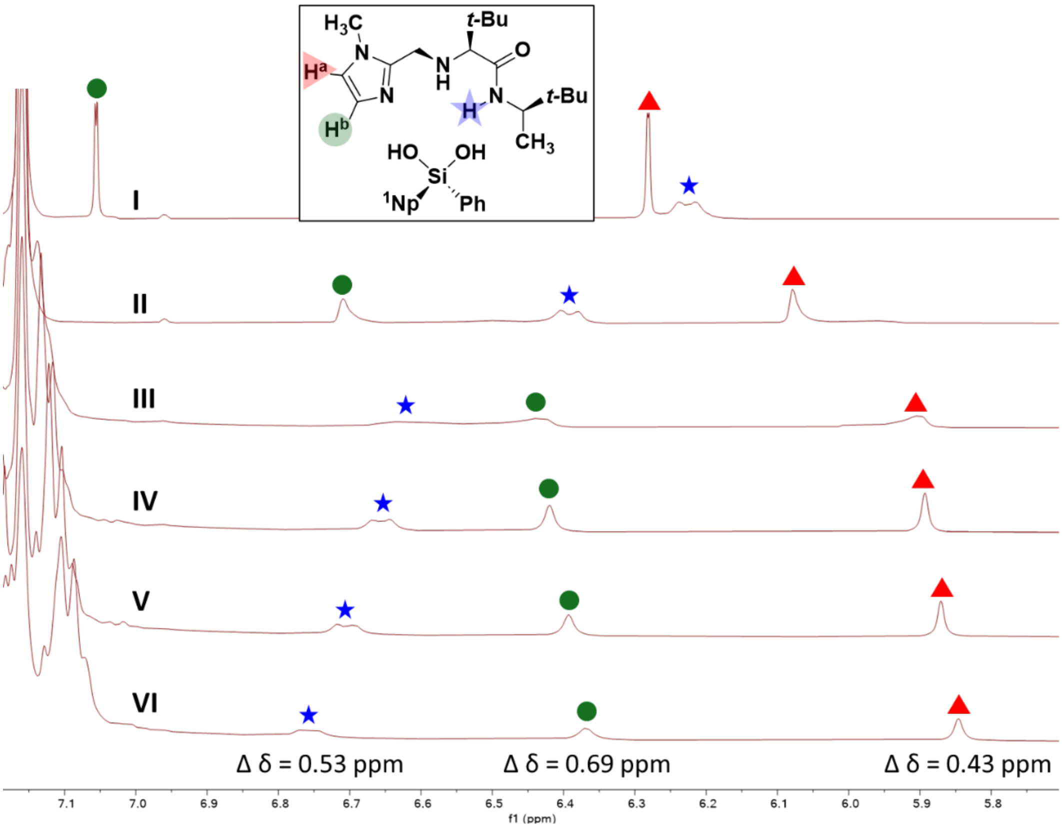

B.

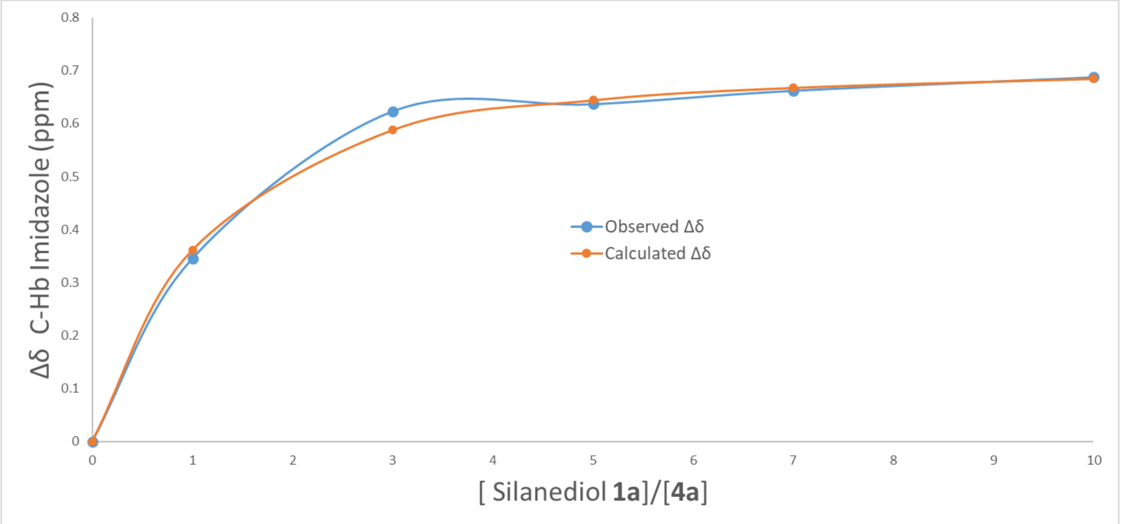

Figure S3. (A) ^{1}H NMR binding study of chiral imidazole **4a** (2 mM in benzene d-6) in the presence of silanesdiol **1a; I** = 0 equiv, **II** = 1 equiv, **III** = 3 equiv, **IV** = 5 equiv, **V** = 7 equiv, **VI** = 10 equiv**.** Δδ = 0.69 is observed for the C-H^{b} peak in the presence of 10 equivalents of **1**a. (B) binding constants were calculated using ^{1}H NMR titration data for the imidazole C-H^{b} peak; K_a = 90 ± 3.

A.

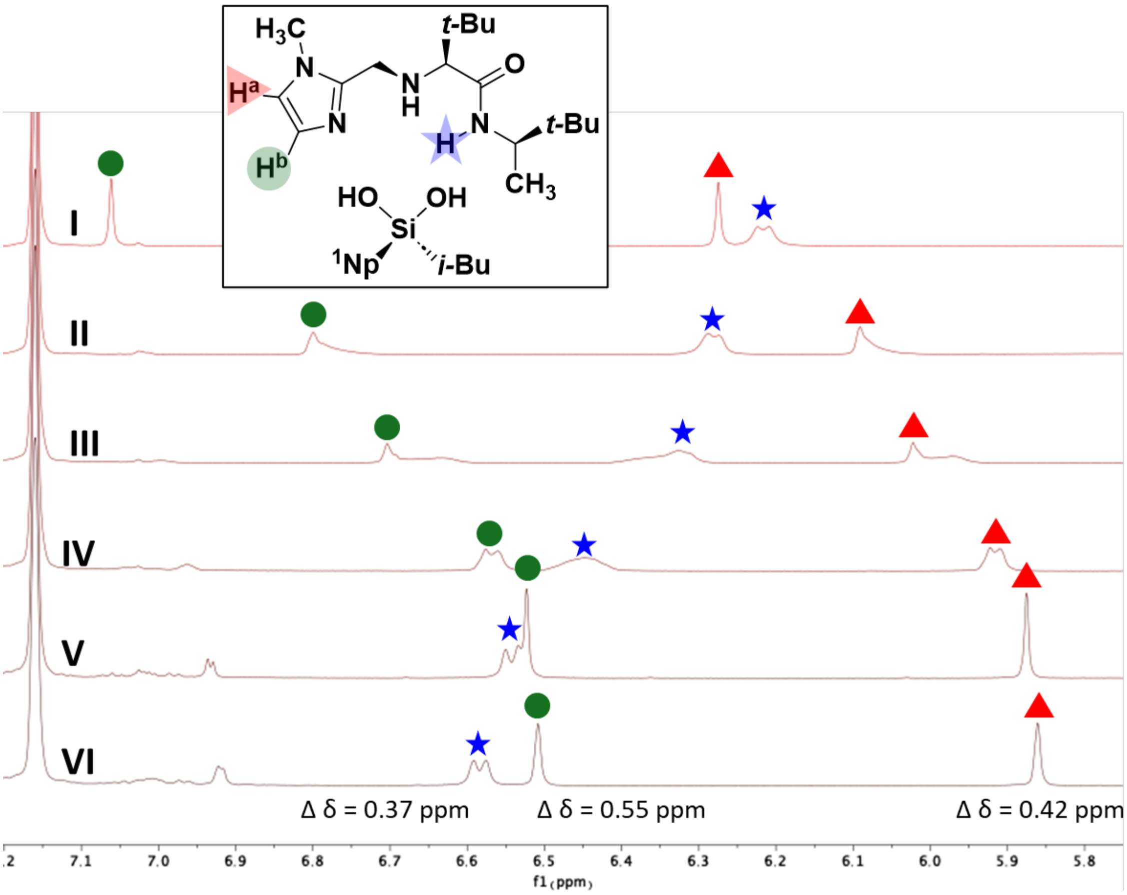

B.

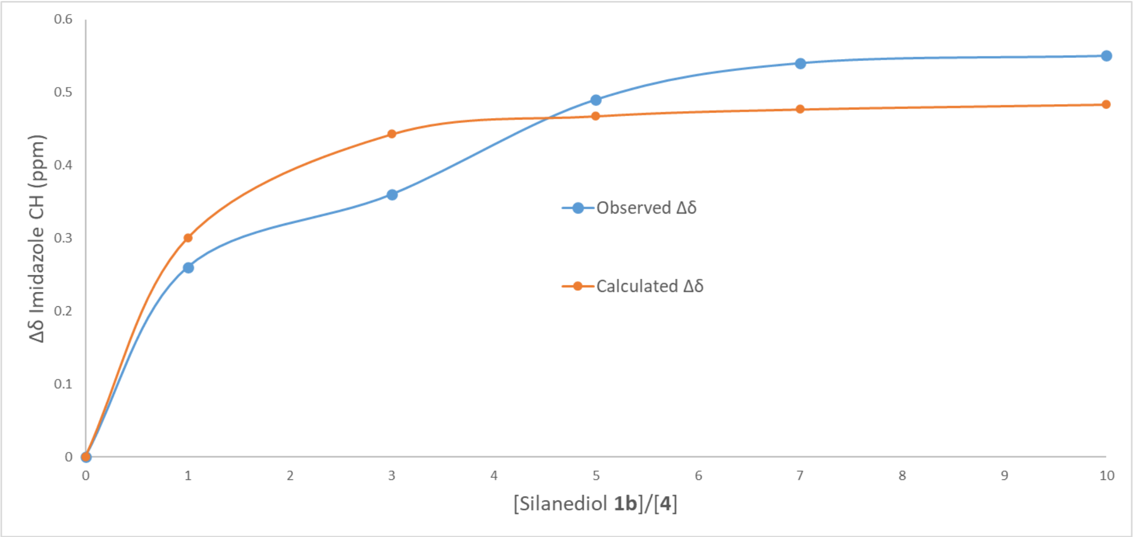

Figure S4. (A) ^{1}H NMR binding study of chiral imidazole **4a** (2 mM in C_6D_6) in the presence of silanediol **1b**; **I** = 0 equiv, **II** = 1 equiv, **III** = 3 equiv, **IV** = 5 equiv, **V** = 7 equiv, **VI** = 10 equiv**.** Δδ = 0.55 ppm is observed for the C-H^b peak in the presence of 10 equivalents of **1b**. (B) binding constants were calculated using ^{1}H NMR titration data for the C-H^b peak; K_a = 191 ± 25.

A.

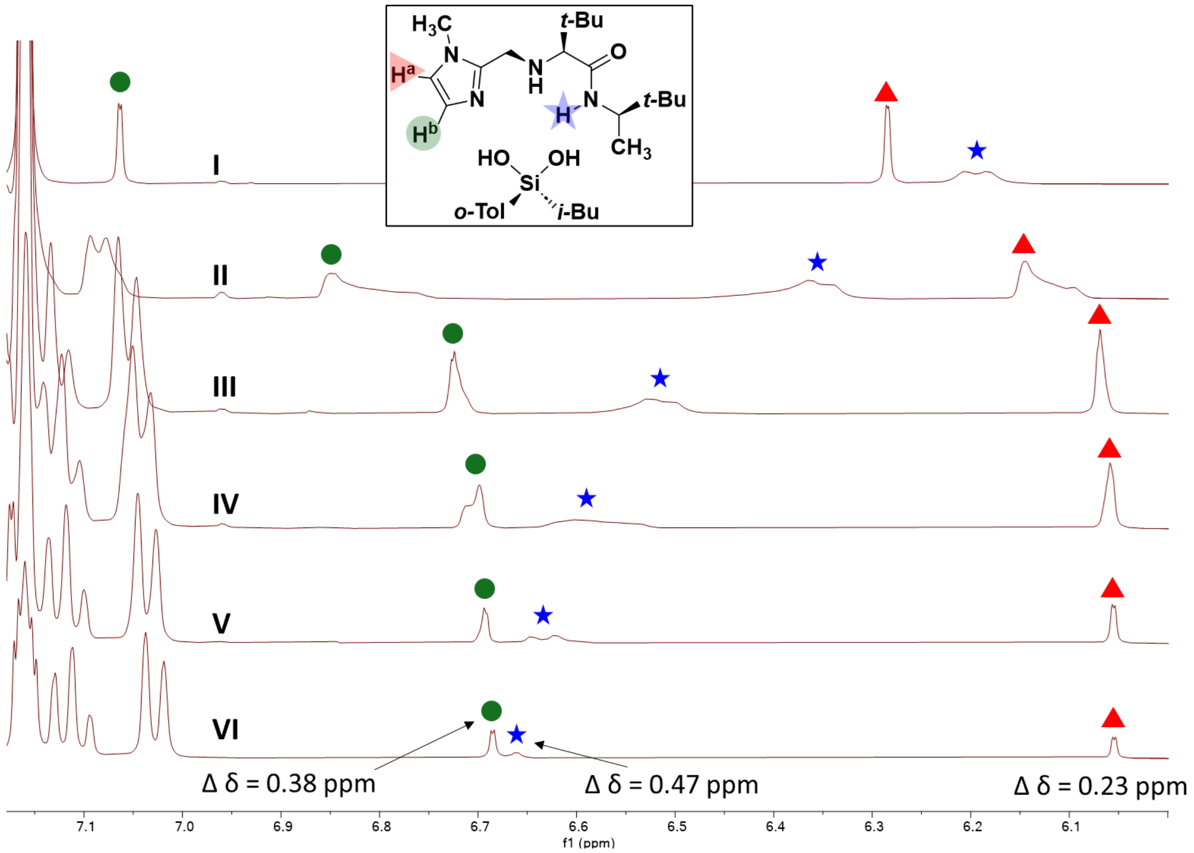

B.

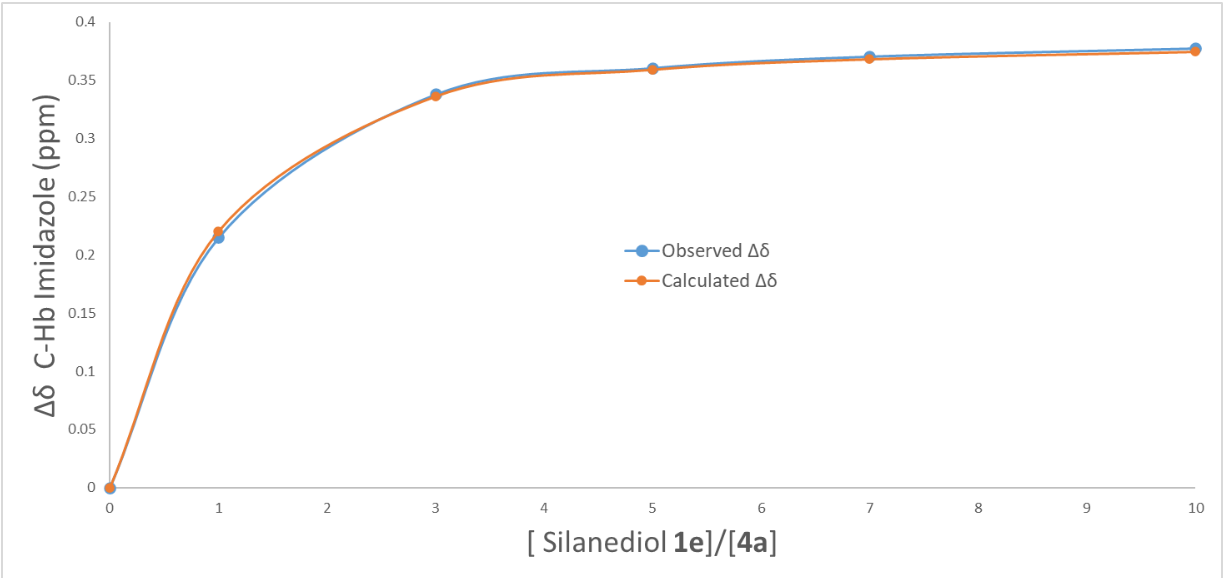

Figure S5. (A) ^{1}H NMR binding study of chiral imidazole **4a** (2 mM in benzene d-6) in the presence of silanediol **1e; I** = 0 equiv, **II** = 1 equiv, **III** = 3 equiv, **IV** = 5 equiv, **V** = 7 equiv, **VI** = 10 equiv**.** Δδ = 0.38 is observed for the C-H^b peak in the presence of 10 equivalents of **1**e. (B) binding constants were calculated using ^{1}H NMR titration data for the C-H^b peak; K_a = 151 ± 2.

A.

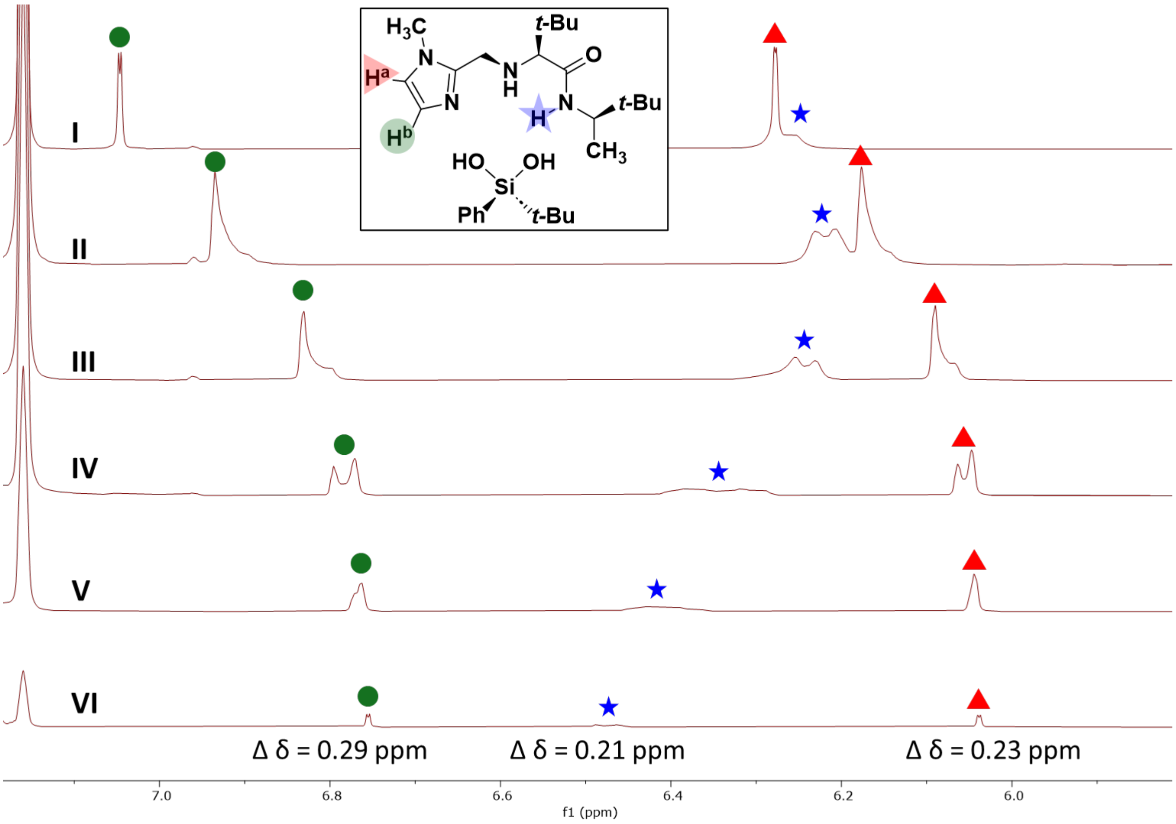

B.

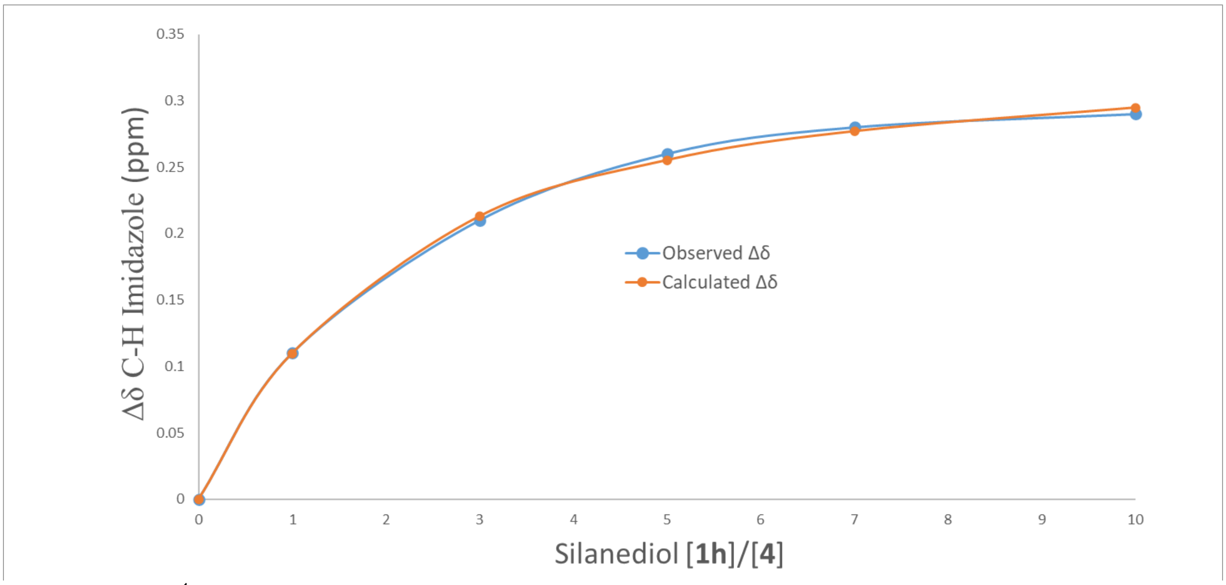

Figure S6. (A) ^{1}H NMR binding study of chiral imidazole **4a** (2 mM in benzene d-6) in the presence of silanesdiol **1j**; **I** = 0 equiv, **II** = 1 equiv, **III** = 3 equiv, **IV** = 5 equiv, **V** = 7 equiv, **VI** = 10 equiv**.** Δδ = 0.29 is observed for the C-H^b peak in the presence of 10 equivalents of **1j**. (B) binding constants were calculated using ^{1}H NMR titration data for the C-H^b peak; K_a = 35 ± 1.

A.

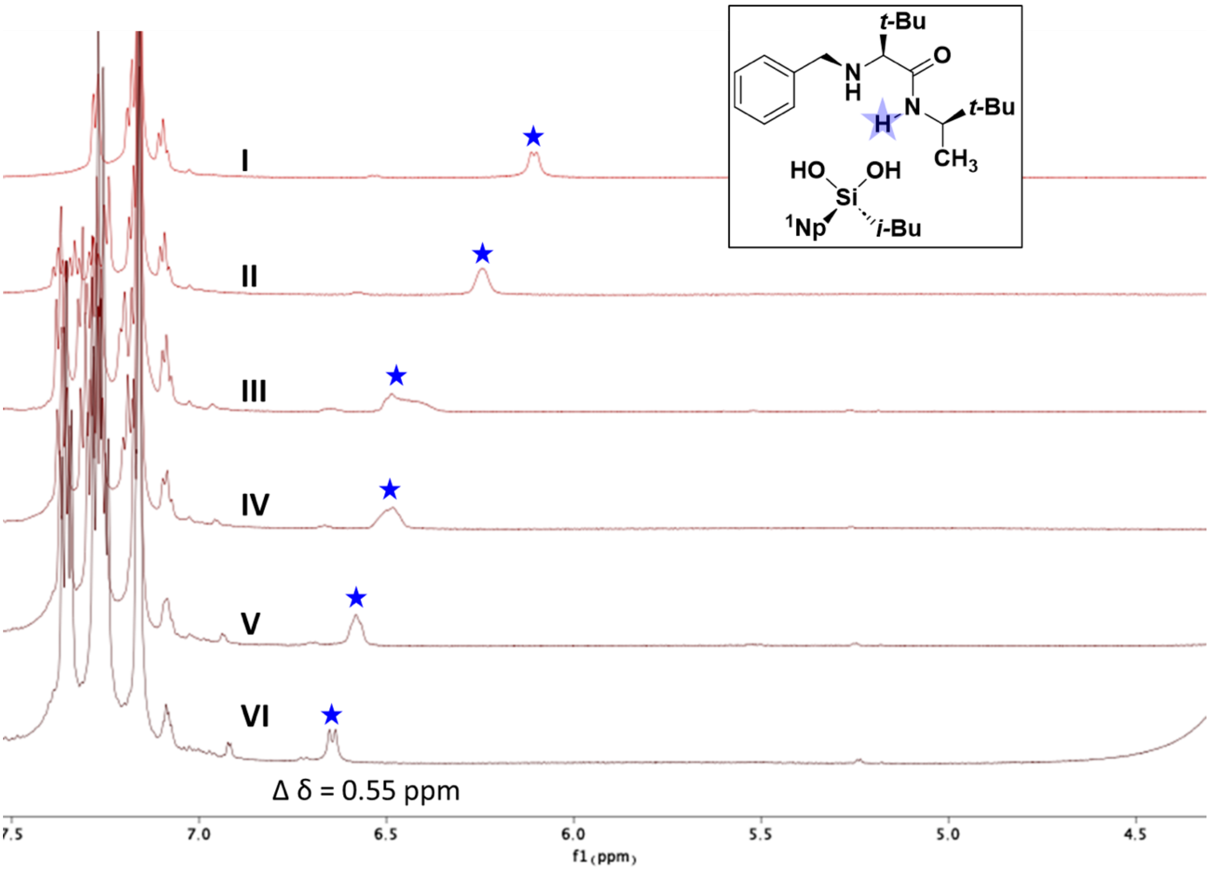

B.

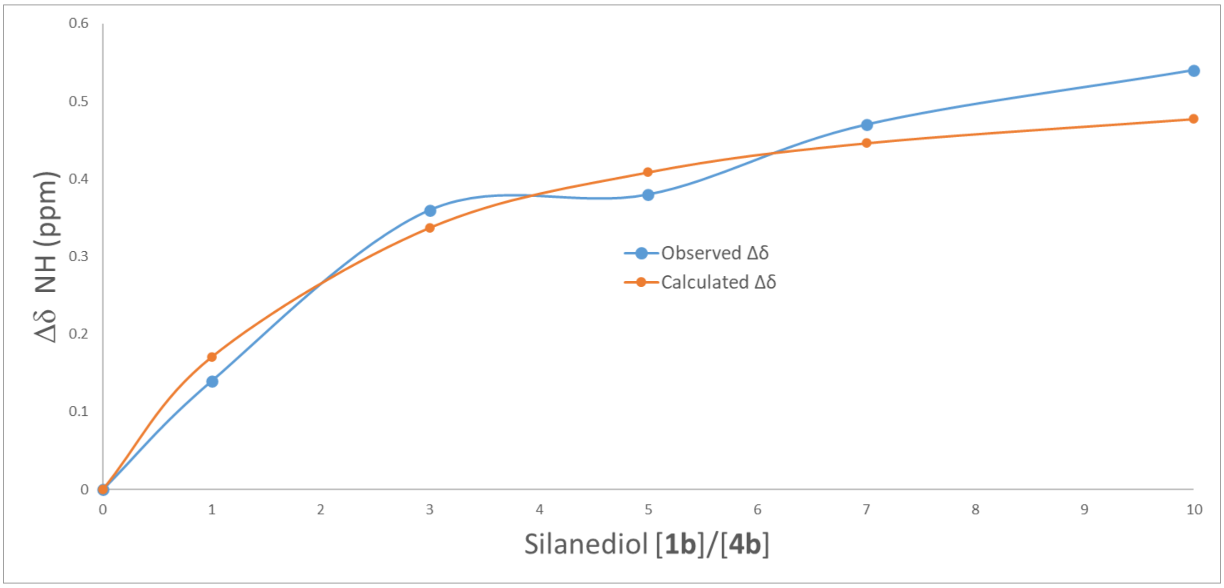

Figure S7. (A) ^{1}H NMR binding study of **4b** (benzyl derivative) (2 mM in C_6D_6in the presence of silanediol **1b**; **I** = 0 equiv, **II** = 1 equiv, **III** = 3 equiv, **IV** = 5 equiv, **V** = 7 equiv, **VI** = 10 equiv**.** Δδ = 0.55 is observed for the amide N-H peak in the presence of 10 equivalents of **1b**. (B) binding constants were calculated using ^{1}H NMR titration data for the amide N-H peak; K_a = 31 ± 4.

A.

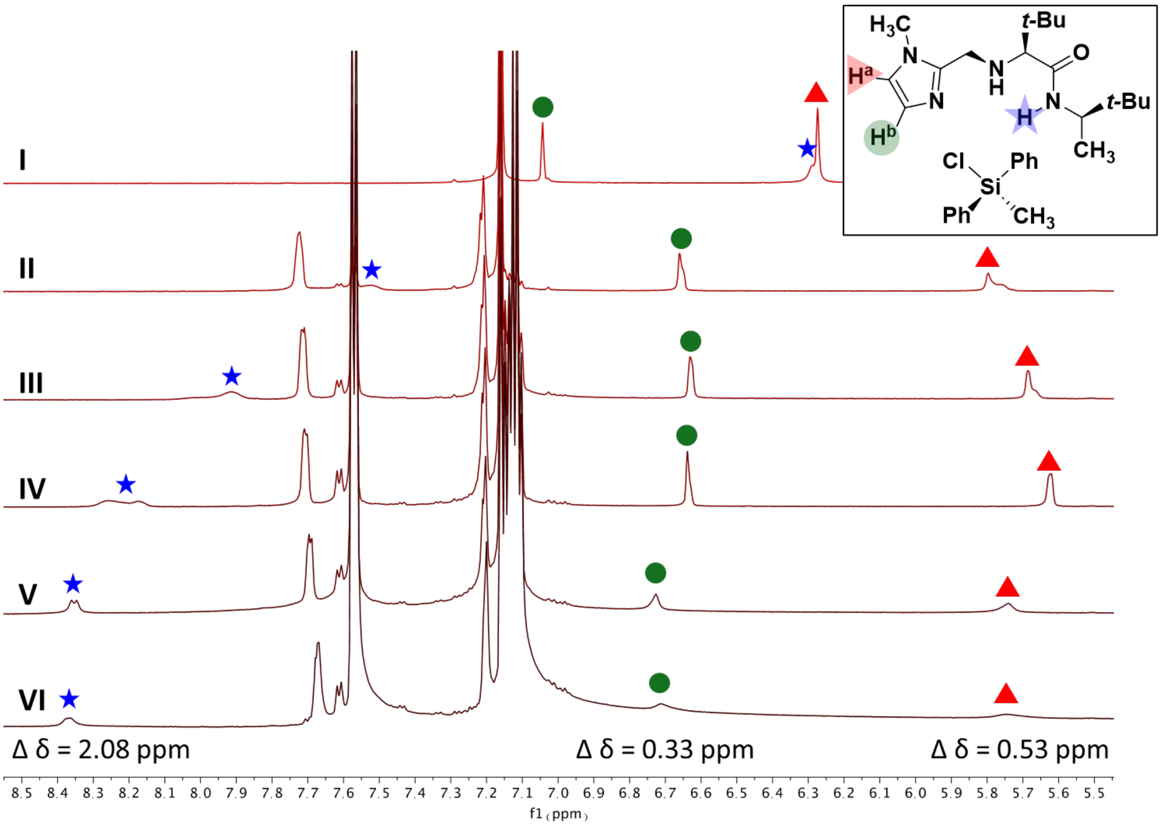

B.

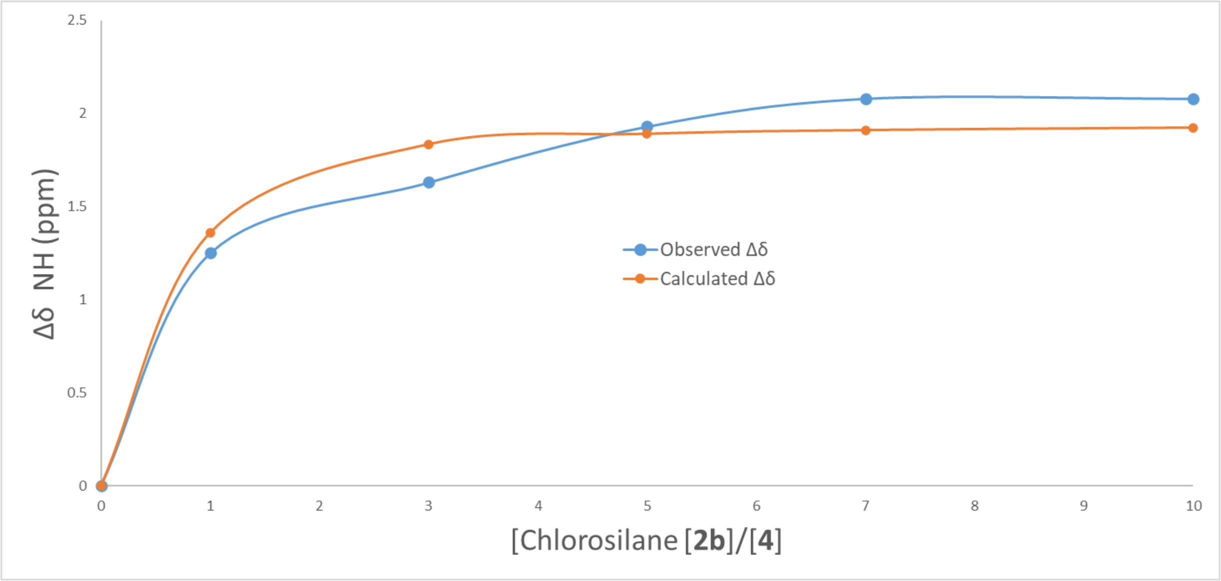

Figure S8. (A) ^{1}H NMR binding study of chiral imidazole **4a** (2 mM in benzene d-6) in the presence of chlorosilane **2b**; **I** = 0 equiv, **II** = 1 equiv, **III** = 3 equiv, **IV** = 5 equiv, **V** = 7 equiv, **VI** = 10 equiv**.** Δδ = 2.08 ppm is observed for the amide NH peak in the presence of 10 equivalents of **2b**. (B) binding constants were calculated using ^{1}H NMR titration data for the amide NH peak; K_a = 379 ± 35.

A.

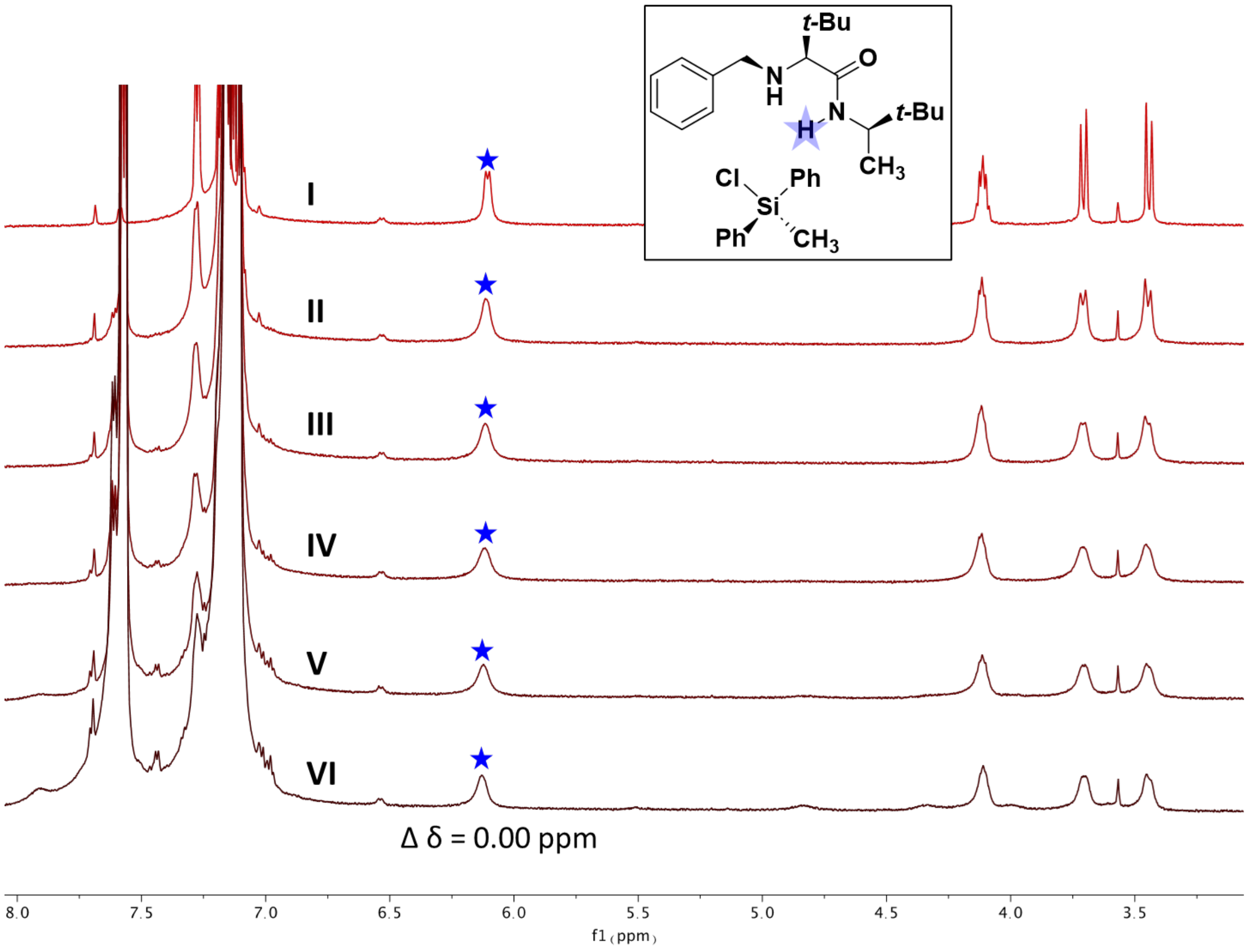

Figure S9. ^{1}H NMR binding study of chiral imidazole **4b** (2 mM in benzene d-6) in the presence of chlorosilane **2b**; No shift (Δδ = 0.00) for the amide NH peak is observed in the presence of 10 equivalents of **2b**.

5.3 Summary of binding data using ^{1}H NMR spectroscopy

Table S3. Summary of binding data with variations in host and guest.

Host	Guest	Δδ (ppm) NH (amide)	Δδ (ppm) H_b (imidazole)	Δδ (ppm) H_a (imidazole)	K_a (M^{-1})
**4a**	**1a**	0.53	0.69	0.43	90 ± 3[a]
**4a**	**1b**	0.37	−0.55	−0.42	191 ± 25[a]
**4a**	**1e**	0.38	−0.47	−0.23	151 ± 2[a]
**4a**	**1j**	0.29	−0.21	−0.23	35 ± 1[a]
**4b**	**1b**	0.55	n/a	n/a	31 ± 4[b]
**4a**	**2b**	2.09	−0.33	−0.53	379 ± 35[b]
**4b**	**2b**	0.00	n/a	n/a	n/a

[a]Ka calculated using Δδ for imidazole peak H_b [b]Ka calculated using Δδ for amide NH peak.

5.4 Preliminary correlation of NMR binding data with enantioselectivity

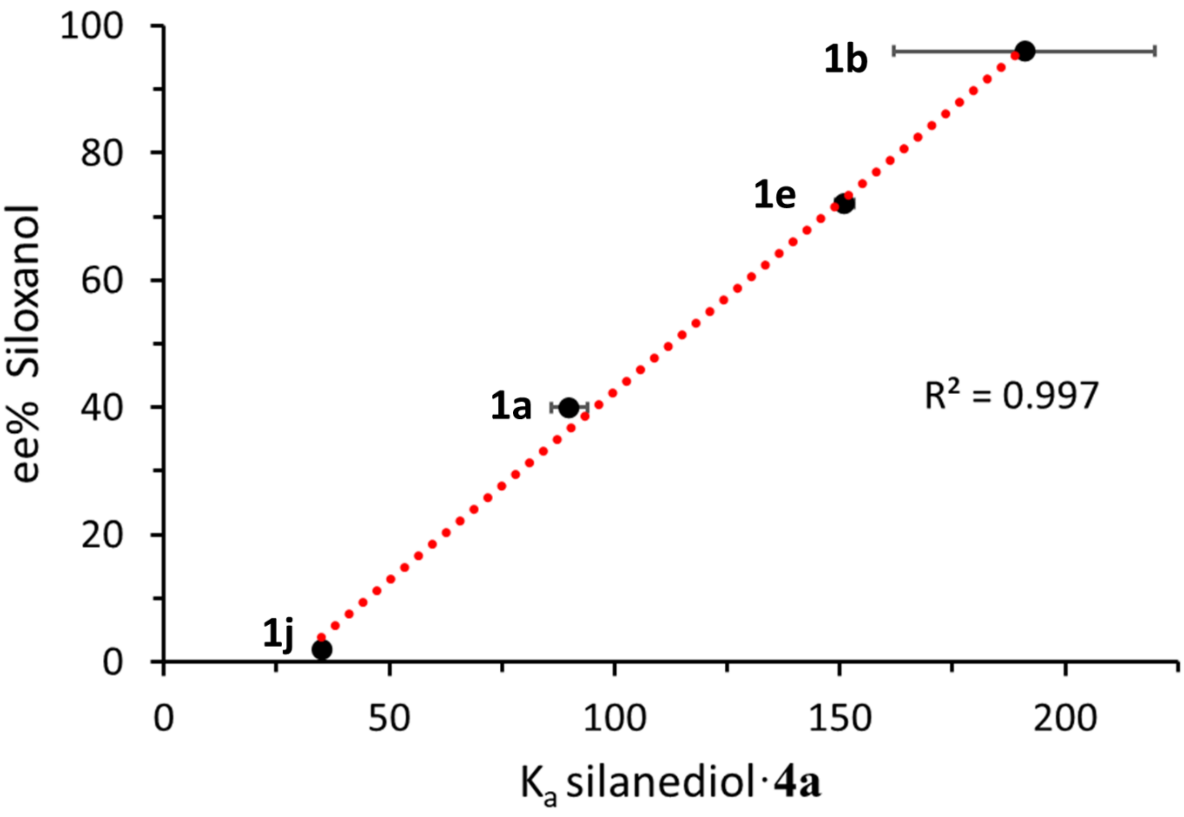

Figure S10. Product ee as a function of catalyst-substrate binding (K_a)

6.0 Lewis base-Chlorosilane ^{29}Si NMR Study

General procedure: Three separate stock solutions were made by dissolving **4a** (0.075 mmol) in 1.0 mL of C_6D_6, freshly distilled **2b** (0.15 mmol) in 2.0 mL of C_6D_6 and NMI (0.075 mmol, from sure seal bottle) in 1.0 mL of C_6D_6 After the stock solutions were prepared, three oven dried NMR tubes were purged with argon and fitted with septa caps. The NMR tubes were then labeled A-C. Tube A was filled with 0.5 mL of **2b** solution. Tube B was filled with 0.25 mL of **2b** solution and 0.25 mL **4b** solution. Tube C was filled with 0.25 mL **2b** solution and 0.25 mL NMI solution. The ^{29}Si DEPT NMR spectrum of each solution was recorded after 128 scans at room temperature on Bruker Nanobay AVIIIHD 400.

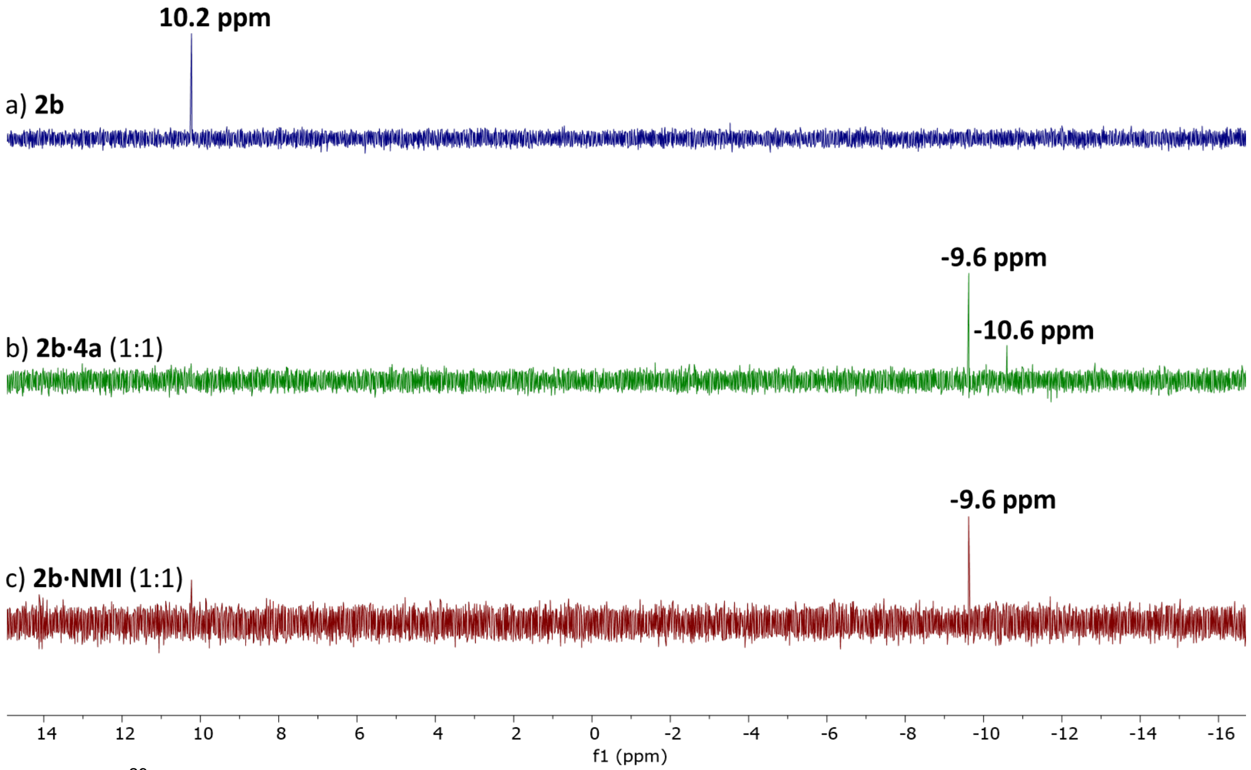

Figure S11 ^{29}Si DEPT NMR Spectra of a) **2b**, b) 1:1 mixture of **2b** and **4a**, c) 1:1 mixture of **2b** and NMI. All spectra taken in C_6D_6 at room temperature.

7. Product competition studies

7.1 General procedure for competition studies

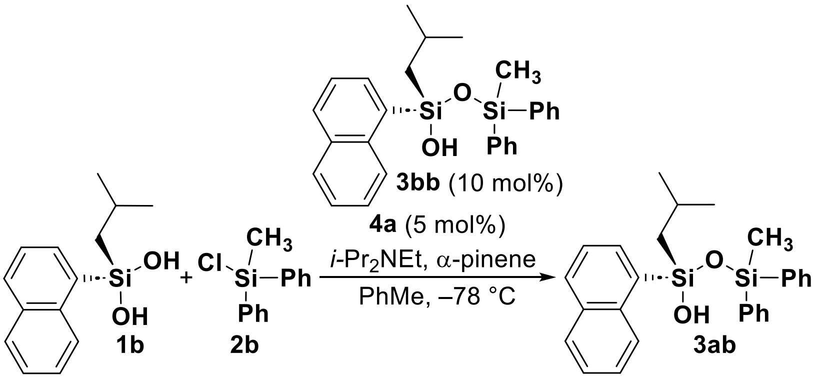

While a glove box was not used care should be taken to ensure the reaction vessel remains air and moisture free to prevent potential undesired condensation products. A 25-mL round bottom flask equipped with a stir bar was flame dried under high vacuum. After the flask cooled to room temperature under vacuum, it was evacuated then purged three times with argon and fitted with a septa and argon balloon. Then desymmetrization agent **4a** (5 mol %, 0.0075 mmol, 0.0023 g), and silanediol **1b** (1.00 equiv, 0.15 mmol, 0.0369 g), were added while maintaining a positive flow of argon into the flask. 2.000 mL of toluene was added. Siloxanol **3b** (10 mol %, 0.015 mmol, 0.1 mL of 0.15 M solution in toluene) was added via syringe. The flask was cooled to –78 °C, and iPr_2NEt (1.20 equiv, 0.18 mmol, 31.4 mL, from a sure sealed bottle) and α-pinene (1.2 equiv, 0.18 mmol, 28.6 mL) were added via syringe. Finally, chlorosilane **2b** (1.00 equiv, 0.15 mmol, 31.5 mL, bottle fitted with a septa and argon balloon, stored under argon) was added and the reaction was stirred at –78 °C in an acetone dry ice bath for 15 min, then transferred as quickly as possible to a cryocool at –78 °C and left to react for an additional 15 h. Then the reaction mixture was quenched with 10% citric acid aqueous solution (2.0 mL) and extracted with Et_2O (5 mL 3 times). Et_2O was specifically chosen, as use of other extraction solvents generally gave reduced yields. The combined organics were washed with brine, dried over Na_2SO_4 and concentrated in vacuo. The product was directly purified using column chromatography on silica gel (9:1 hexanes/EtOAc), the siloxanol product was characterized after purification. TLC analysis of the crude reaction mixture was sufficient to confirm product formation and SM consumption, therefore crude NMR was not taken. HPLC analysis was performed after purification.

Table S4. Comparison of enantiomeric ratio upon addition of product of varying enantiopurity.

entry	er with additive spike	er of final product
1	97:3	97:3
2	75:25	96:4
3	50:50	97:3

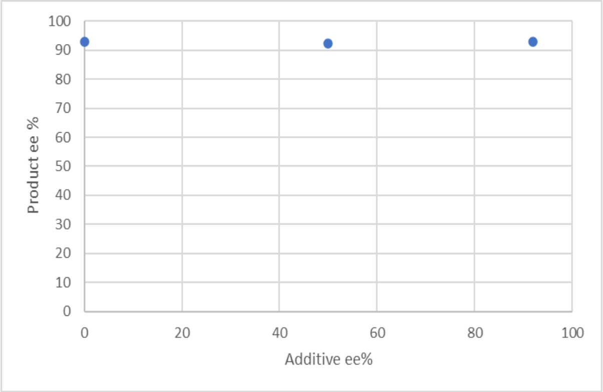

Figure S12. Graph of product ee% vs additive ee%. Additive ee% has no effect on the enantiopurity of the final product.

8. Acid Scavenger Studies

While a glove box was not used care should be taken to ensure the reaction vessel remains air and moisture free to prevent potential undesired condensation products. Three 25-mL round bottom flasks equipped with a stir bar were flame dried under high vacuum. After the flasks cooled to room temperature under vacuum, they were purged three times with argon and fitted with a septa and argon balloon. Then desymmetrization agent **4a** (5 mol %, 0.0075 mmol, 2.30 mg), and silanediol **1b** (1.00 equiv, 0.15 mmol, 36.9 mg), were added while maintaining a positive flow of argon into the flask. 2.00 mL of Toluene was added. The flasks were cooled to –78 °C, and iPr_2NEt (1.20 equiv, 0.18 mmol, 31.4 mL, from a sure sealed bottle) was added.

Entry 1. Chlorosilane **2b** was distilled into a bomb flask and stored under argon (figure S13). Then freshly distilled **2b** (1.00 equiv, 0.15 mmol, 31.5 mL) was added and the reaction was stirred at –78 °C in an acetone dry ice bath for 15 min, then transferred as quickly as possible to a cryocool at –78 °C and left to react for an additional 15 hours. Then the reaction mixture was quenched with 10% citric acid aqueous solution (2.0 mL) and extracted with Et_2O (5.0 mL 3 times). Et_2O was specifically chosen, as use of other extraction solvents generally gave reduced yields. The combined organics were washed with brine, dried over Na_2SO_4 and concentrated in vacuo. The product was purified using column chromatography (9:1 hexanes/EtOAc).

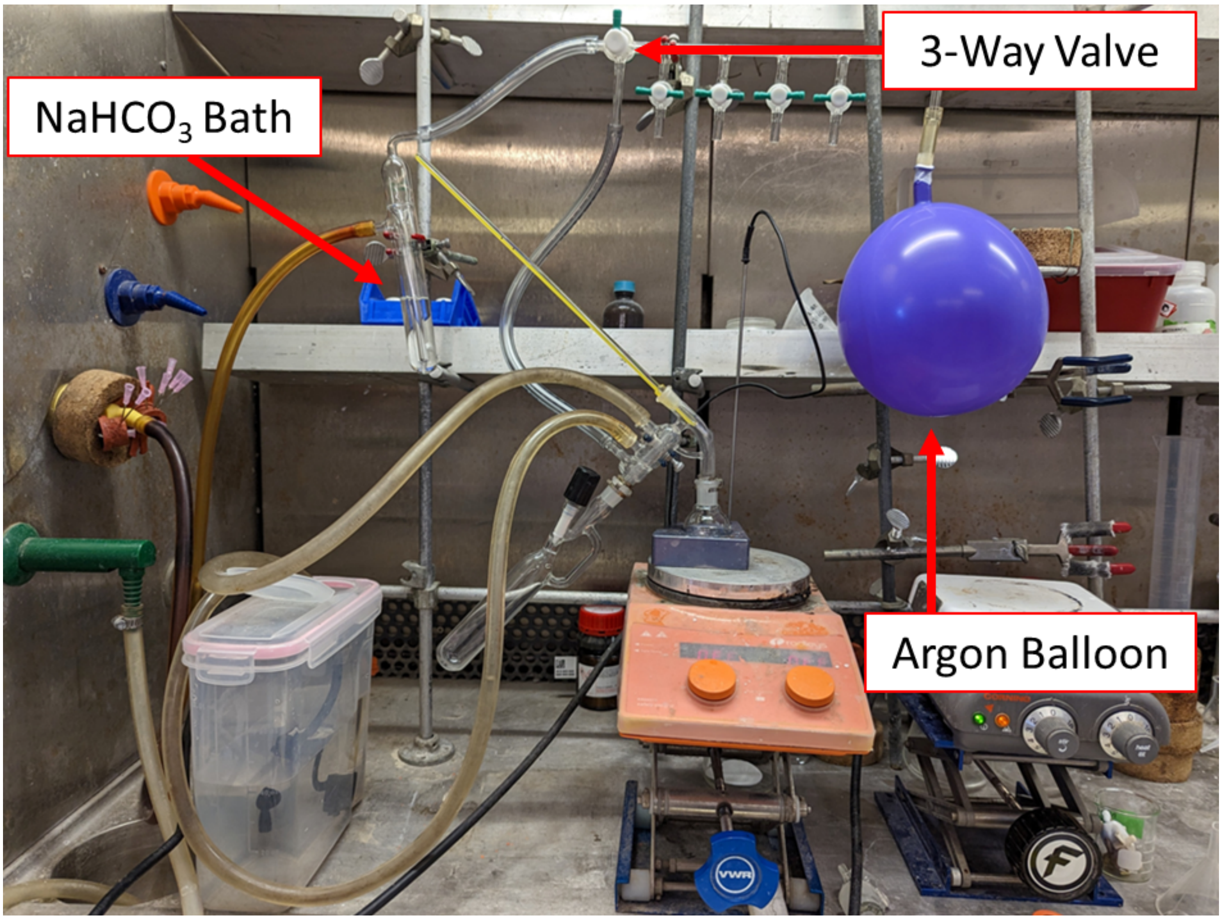

Figure S13 Distillation apparatus for the purification of Chlorosilanes

Entry 2: Terpinene (1.20 equiv, 0.18 mmol, 29.3 mL) was added via syringe. Finally, chlorosilane **2b** (1.00 equiv, 0.15 mmol, 31.5 mL, bottle fitted with a septa and argon balloon, stored under argon) was added and the reaction was stirred at –78 °C in an acetone dry ice bath for 15 min, then transferred as quickly as possible to a cryocool at –78 °C and left to react for an additional 15 hours. Then the reaction mixture was

quenched with 10% citric acid aqueous solution (2.0 mL) and extracted with Et_2O (5.0 mL 3 times). Et_2O was specifically chosen, as use of other extraction solvents generally gave reduced yields. The combined organics were washed with brine, dried over Na_2SO_4 and concentrated in vacuo. The product was purified using column chromatography (9:1 hexanes/EtOAc).

Entry 3: (+)-α-pinene (1.20 equiv, 0.18 mmol, 28.6 mL) was added via syringe. Finally, chlorosilane **2b** (1.00 equiv, 0.15 mmol, 31.5 mL, bottle fitted with a septa and argon balloon, stored under argon) was added and the reaction was stirred at –78 °C in an acetone dry ice bath for 15 min, then transferred as quickly as possible to a cryocool at –78 °C and left to react for an additional 15 hours. Then the reaction mixture was quenched with 10% citric acid aqueous solution (2.0 mL) and extracted with Et_2O (5 mL 3 times). The combined organics were washed with brine, dried over Na_2SO_4 and concentrated in vacuo. The product was purified using column chromatography (9:1 hexanes/EtOAc.)

Entry 4: (-)-α-pinene (1.20 equiv, 0.18 mmol, 28.6 mL) was added via syringe. Finally, chlorosilane **2b** (1.00 equiv, 0.15 mmol, 31.5 mL, bottle fitted with a septa and argon balloon, stored under argon) was added and the reaction was stirred at –78 °C in an acetone dry ice bath for 15 min, then transferred as quickly as possible to a cryocool at –78 °C and left to react for an additional 15 hours. Then the reaction mixture was quenched with 10% citric acid aqueous solution (2.0 mL) and extracted with Et_2O (5 mL 3 times). The combined organics were washed with brine, dried over Na_2SO_4 and concentrated in vacuo. The product was purified using column chromatography (9:1 hexanes/EtOAc.)

Enantiomeric ratios for all three entries were determined after purification by HPLC with Diacel CHIRALPAK ® AD-H column (1% IPA/hexanes) 1 mL/min, **3ab1 =** 6.6 min, **3ab2** 7.3 min.

Table S5. A comparison of yield and enantiomeric ration when using various methods of acid control during the desymmetrization process.

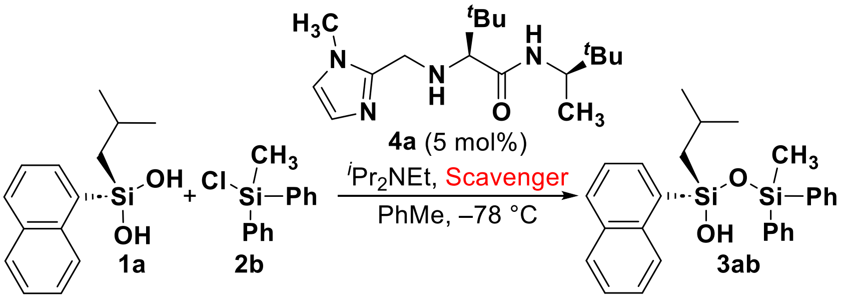

Entry	Acid Scavenger	Yield (%)	er
1	none	93	95:5
2	Turpinene	72	97:3
3	(+)-α-Pinene	98	98:2
4	(-)-α-Pinene	89	92:8

9. Proposed catalytic cycle

The catalytic cycle begins with the formation of the catalyst substrate complex (**A**) as supported by x-ray crystallography and ^{1}H NMR binding studies. Silanols form hydrogen bonding networks in solution, and X-ray crystallography confirms that in the case of **1b** the lowest energy conformation in the solid state is a cyclic trimer, therefore an equilibrium between the self-associated silanediol and complex **A** is likely in solution. After **A** has formed the chlorosilane is activated by **4a,** activation of 2 by free 4a results in the formation of an off cycle pentavalent silicon. For productive activation, ^{29}Si NMR, and co-catalyst experiments support the activation of **2** by the same molecule of **4b** that is bound to **1** (**B**). Kinetic analysis of the desymmetrization to confirm the catalyst order has been attempted using in situ infrared spectroscopy but we were unable to obtain reproducible results due to the lack of distinctive IR peaks to track, and due to unexpected changes in reaction rate between trials. After the **B** is formed the free silanol of **1** performs a nucleophilic attack (**C**) on the activated chlorosilane to produce **3** which is then released. Once **3** is formed it is inert to reaction conditions, trisiloxane products are not observed, and product addition studies found that the enrichment of **3** does not affect the stereochemical outcome of **1** as it is converted to **3**.

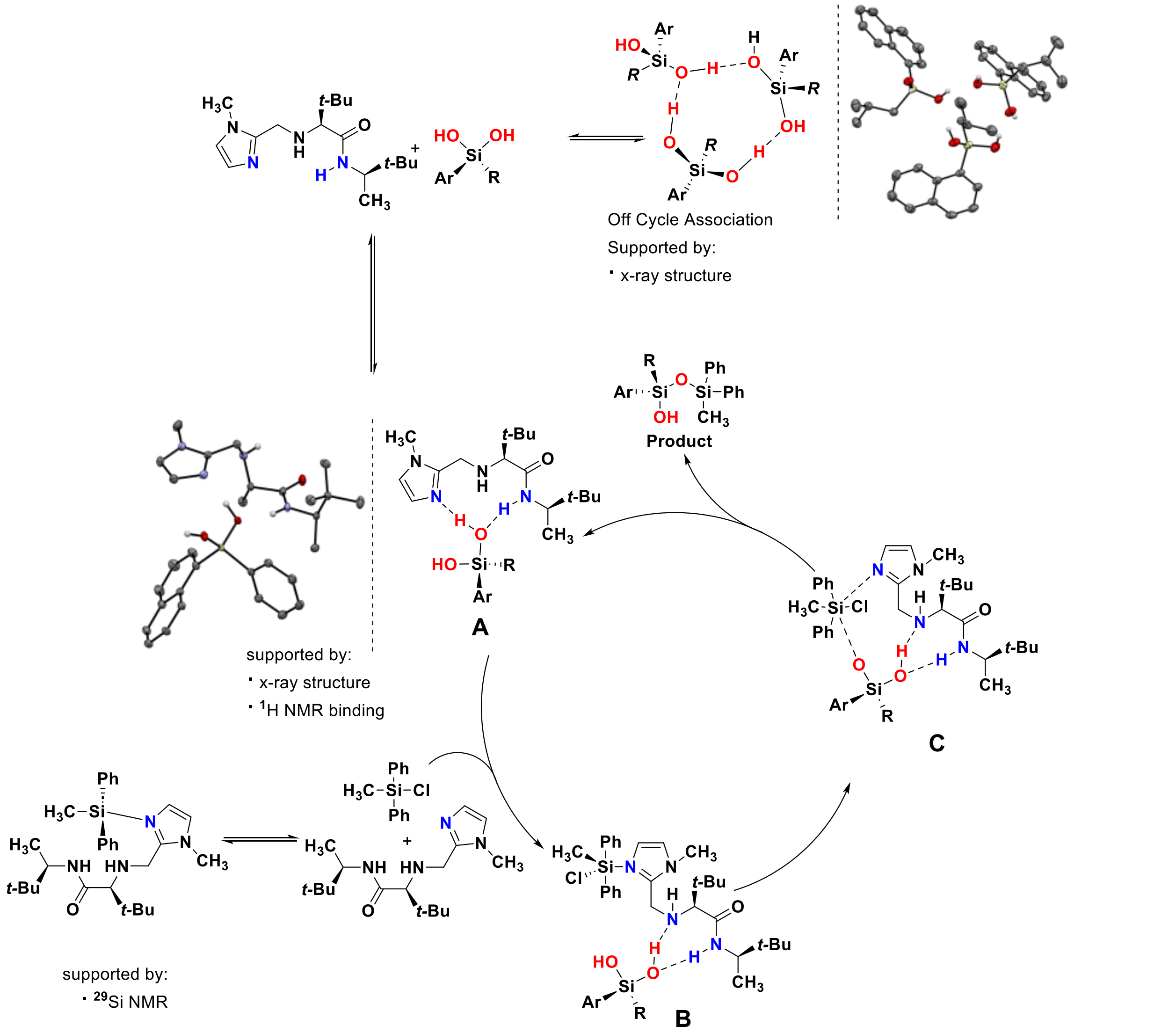

Figure S14. Lewis base activation pathways for silylative desymmetrization

10. Characterization of reported compounds

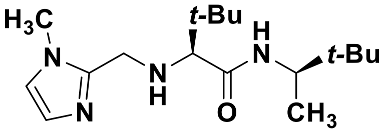

(*S*)-*N*-((*R*)-3,3-dimethylbutan-2-yl)-3,3-dimethyl-2-((1-methyl-1H-imidazol-2-yl)methylamino)butanamide (4a). Compound **4a** was synthesized according to literature precedent and found to match previously reported samples.[9]H NMR (600 MHz, $CDCl_3$) δ 6.94 (d, *J* = 1.0 Hz, 1H), 6.82 (d, *J* = 1.0 Hz, 1H), 6.56 (d, *J* = 9.6 Hz, 1H), 3.92 (dq, *J* = 9.8, 6.8 Hz, 1H), 3.80 (d, *J* = 14.0 Hz, 1H), 3.63 (s, 3H), 3.62 (dd obscured, 1H), 2.69 (s, 1H), 1.77 (s, br, 1H), 1.07 (d, *J* = 6.8 Hz, 3H), 0.97 (s, 9H), 0.93 (s, 9H).

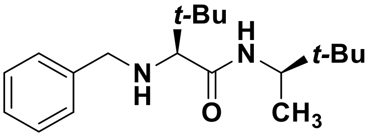

(*S*)-2-(benzylamino)-(*R*)-3,3-dimethylbutan-2-yl)-3,3-dimethylbutanamide (4b). Compound **4b** was synthesized by adapting the previously reported synthesis of **4a**.[9] A round bottom flask with stir bar was charged with *(S)*-2-amino-*N*-*((R)*-3,3-dimethylbutan-2-yl)-3,3-dimethylbutanamide (0.61 mmol, 131.7 mg, synthesized over 2 steps and carried forward without purification), and $MgSO_4$ (132.4 mg, 100 wt %), then CH_2Cl_2 (10.0 mL) was added. Benzaldehyde (0.61 mmol, 62.7 µL) was added and reagents were allowed to mix for 16 h. Rxn contents were then filtered and concentrated to leave a white solid. The white solid was dissolved in MeOH (10 mL), and cooled to 0 °C, then $NaBH_4$ (3.0 equiv, 1.83 mmol, 63.1 mg), and 1 drop of concentrated HCl were added. Rxn warmed to room temperature and allowed to mix 1h, then 5 mL of a saturated solution of $NaHCO_3$ was added to quench the reaction. The resulting mixture was washed with CH_2Cl_2 (3x10mL) and brine (1x10mL) and then dried over $MgSO_4$ and concentrated. Product purified by column chromatography (85:15 hex:EtOAc) and obtained as a white solid (mp: 102.0 °C). Yield: 0.171 g (91% over 4 steps). ^{1}H NMR (600 MHz, $CDCl_3$) 7.28 (d, *J* = 7.2 Hz, 2H), 7.19 (t obscured, *J* = 7.3 Hz, 2H), 7.10 (d, *J* = 7.1 Hz, 1H), 6.11 (d, *J* = 7.8 Hz, 1H), 4.11 (dq, *J* = 16.3, 7.0 Hz, 1H), 3.71 (d, *J* = 13.2 Hz, 1H), 3.44 (d, *J* = 13.1 Hz, 1H), 2.67 (s, 1H), 1.76 (s, 1H), 0.99 (s, 6H), 0.89 (d, *J* = 7.0 Hz, 4H), 0.84 (s, 6H). ^{13}C NMR (100 MHz,C_6D_6) δ 171.1, 140.2, 128.4, 128.1, 127.1, 71.6, 52.8, 52.3, 33.7, 33.5, 27.1, 26.2, 16.0. HRMS (ESI) m/z: calc for $C_{19}H_{33}N_2O$ $[M + H]^+$ 305.2587 found 305.2592.

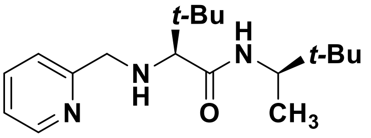

(*S*)-2-(pyrl)-*N*-((*R*)-3,3-dimethylbutan-2-yl)-3,3-dimethylbutanamide (4c).
Compound **4c** was synthesized by adapting the previously reported synthesis of **4a**.[2] A flame-dried round bottom flask with stir bar was charged with *(S)*-2-amino-*N*-*((R)*-3,3-dimethylbutan-2-yl)-3,3-dimethylbutanamide (4.72 mmol, 1.0110 g, synthesized over 2 steps and carried forward without purification), and then $MgSO_4$ (0.83 equiv, 4.72 mmol, 0.4706 g) and CH_2Cl_2 (2.4 mL) was added. 2-Pyridinecarboxaldehyde (1.0 equiv, 4.72 mmol, 0.45 mL) and reagents were stirred at room temperature for 18 h. Rxn contents were filtered and concentrated to yield an orange oil. The oil was then dissolved in MeOH (2 mL), then $NaBH_4$ (3.0 equiv, 14.16 mmol, 0.5357 g), and (0.07 equiv, 0.31 mmol, 9.5 µL) of

concentrated HCl were added. Reaction mixture was stirred at 0 °C for 5 min then at room temperature for 1.5h. Then 5 mL of a saturated solution of $NaHCO_3$ was added to quench the reaction. The resulting mixture was washed with CH_2Cl_2 (3 x 10 mL) and brine (1 x 10 mL) and then dried over $MgSO_4$ and concentrated in vacuo. Product purified by column chromatography (98:2 DCM/MeOH) to 96:4 DCM/MeOH with 0.2% TEA, and obtained as a brown oil. Compound Yield: 0.1350 g (9% over 4 steps). ^{1}H NMR (400 MHz, $CDCl_3$) δ 8.56 (ddd, *J* = 4.9, 1.8, 0.9 Hz, 1H), 7.63 (dd, *J* = 7.7, 1.8 Hz, 1H), 7.19 (m, 2H), 3.89 (m, 1H), 3.85 (d, *J* = 13.7 Hz, 2H), 3.66 (d, *J* = 14.1 Hz, 1H), 2.80 (s, 1H), 1.06 (d, *J* = 6.7 Hz, 4H), 1.01 (s, 10H), 0.91 (s, 9H). ^{13}C NMR (100 MHz, $CDCl_3$) δ 171.9, 136.7, 122.6, 122.4, 72.6, 53.9, 53.2, 34.1, 33.9, 27.5, 26.6, 16.4. HRMS (ESI) m/z: calc for $C_{18}H_{32}N_3O$ $[M + H]^+$ 306.2540, found 306.2547.

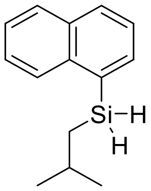

(Naphthalen-1-yl)isobutylsilane (S1): Magnesium turnings (0.350 g, 14.4 mmol, 1.20 equiv) were added to a round-bottom flask. The flask was flame-dried under vacuum and back filled with argon. 20 mL of THF were added to the round-bottom flask followed by the addition of dibromoethane (4.0 mmol, 0.34 mL,0.2 equiv) which was allowed to react with the magnesium turnings until the solution turned a dark grey color (about 30 minutes). Then 1-bromonapthalene (1.68 mL, 12.0 mmol, 1.00 equiv) was added and allowed to stir at room temperature for 5 h. trichloroisobutylsilane (1.83 mL, 12.0 mmol, 1.00 equiv) was then added at once quickly. The reaction was warmed to room temperature and stirred for 16 h. The reaction was then cooled down to –78 °C and LAH (4.00 M in Et_2O, 6.00 mL, 24.0 mmol, 2.0 equiv) was added dropwise. Then the reaction was warmed to room temperature, stirred for an additional 3 h, at which time the reaction was quenched by the addition of a solution of saturated aq. Rochelle's salt (15 mL) and filtered over celite. The organic layer was separated, and the aqueous layer was washed with Et_2O (3 x 10 mL), then the organic layers were combined and washed with brine (10 mL), dried over anhydrous magnesium sulfate, filtered, and then concentrated in vacuo. The crude product was purified by bulb-to-bulb distillation to yield **S1** as a clear oil. Yield: 1.80 g (71%); ^{1}H NMR (600 MHz,C_6D_6) δ 8.14 (d, *J* = 8.3 Hz, 1H), 7.74 (d, *J* = 6.6 Hz, 1H), 7.65 (m, 2H), 7.33 (t, *J* = 7.5 Hz, 1H), 7.27 (t, *J* = 7.4 Hz– 1H), 7.25 - 7.20 (m, 1H), 4.83 (s, 1H), 1.76 (m, 1H), 0.94 (m, 3H), 0.91 (d, *J* = 6.6 Hz, 6H). ^{13}C NMR (100 MHz, $CDCl_3$) δ 137.4, 135.8, 133.2, 131.9, 130.5, 129.0, 126.3, 125.9, 125.4, 26.1, 25.6, 20.8. ^{29}Si NMR (80 MHz, $CDCl_3$) δ –36.02.

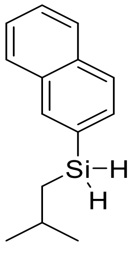

(Naphthalen-2-yl)isobutylsilane (S2): Magnesium turnings (0.268 g, 11.0 mmol, 1.10 equiv) and LiCl (0.475 g, 11.0 mmol, 1.10 equiv) were added to a round-bottom flask. The flask was flame-dried under vacuum and back filled with argon. Then THF (20 mL) was added to the round-bottom flask. Then 2-bromonapthalene (2.07 g, 10.0 mmol, 1.00 equiv) was added and allowed to stir at room temperature for 5 h. trichloroisobutylsilane (1.68 mL, 11.0 mmol, 1.10 equiv) was taken up in 10 mL THF in a separate flame-dried flask, then the Grignard was added slowly via cannula to the solution of chlorosilane. The resulting

mixture was stirred for 16 h. The reaction was then cooled down to –78 °C and LAH (4.00 M in Et_2O 5.00 mL, 20.0 mmol, 2.0 equiv) was added dropwise. Then the reaction was warmed to room temperature, stirred for an additional 3 h, at which time the reaction was quenched by the addition of a solution of saturated aq. Rochelle's salt (15 mL) and filtered over celite. The organic layer was separated, and the aqueous layer was washed with Et_2O (3 x 10 mL), then the organic layers were combined and washed with brine (10 mL), dried over anhydrous magnesium sulfate, filtered, and then concentrated in vacuo. The crude product was purified by bulb-to-bulb distillation, followed by sublimation (80 °C at 1 atm) to yield **S2** as a clear oil. Yield: 1.08 g (50%); ^{1}H NMR (400 MHz, $CDCl_3$) δ 8.11 (s, 1H), 7.84 (dd, *J* = 8.5, 4.9 Hz, 3H), 7.62 (dd, *J* = 8.1, 1.2 Hz, 1H), 7.55 – 7.47 (m, 2H), 1.90 (m, 1H), 1.06 – 1.00 (m, 8H).^{13}C NMR (150 MHz, C_6D_6) δ 135.5, 134.8, 134.6, 133.5, 130.3, 128.7, 128.4, 127.5, 127.0, 126.3, 26.3, 25.9, 24.3. ^{29}Si NMR (80 MHz, C_6D_6, $Cr(acac)_3$) δ –33.52.

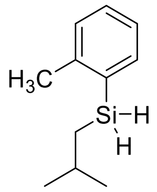

Isobutyl(o-tolyl)silane (S3): Magnesium turnings (0.5846 g, 24.0 mmol, 1.20 equiv) were added to a round-bottom flask. The round bottom flask was flame-dried under vacuum and back filled with argon. 60 mL of THF were added to the round-bottom flask, followed by dibromoethane (0.34 mL, 4.0 mmol, 0.2 equiv) and the flask was left to mix for 1 h, at which time the solution turned dark grey. 1-Bromo-2-methylbenzene (2.40 mL, 20.0 mmol, 1.00 equiv) was added to the reaction and allowed to mix until Mg turnings were consumed (about 10 h). The reaction was cooled to –78 °C and trichloroisobutylsilane (3.33 mL, 20.0 mmol, 1.00 equiv) was then quickly added as one aliquot to avoid double addition of Grignard. The reaction was warmed to room temperature and stirred for 24 h. The reaction was again cooled to –78 °C and LAH (4.00 M in Et_2O, 10.00 mL, 40.0 mmol, 2.0 equiv) was added dropwise. Then the reaction was warmed to room temperature and stirred for an additional 3 h, at which time the reaction was quenched by the addition of a solution of saturated aq. Rochelle's salt (15 mL) and filtered over celite. The organic layer was separated, and the aqueous layer was washed with Et_2O (3 x 10 mL), then the organic layers were combined and washed with brine (10 mL), dried over anhydrous magnesium sulfate, filtered, and then concentrated in vacuo. The crude product was purified by column chromatography (100% hexanes) to yield **S3** as a clear oil. Yield: 1.22 g (34%); ^{1}H NMR (400 MHz, $CDCl_3$) δ 7.57 (d, *J* = 1.3 Hz, 1H), 7.34 (td, *J* = 7.5, 1.5 Hz, 1H), 7.21 (d, *J* = 7.5 Hz, 2H), 4.38 (t, *J* = 4.0 Hz, 2H), 2.48 (s, 3H), 1.88 (m, 1H), 1.03 (d, *J* = 6.6 Hz, 6H), 0.99 (dt, *J* = 7.0, 4.0 Hz, 2H). ^{13}C NMR (100 MHz, $CDCl_3$) δ 144.2, 136.4, 132.5, 130.1, 129.5, 125.3, 26.0, 25.6, 22.7, 20.3. ^{29}Si NMR (80 MHz, $CDCl_3$) δ –37.06.

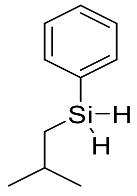

Isobutyl(phenyl)silane (S4): A round-bottom flask was flame-dried under vacuum and back filled with argon. 50 mL of THF were added to the round-bottom flask. PhMgBr (6.66 mL, 20.0 mmol, 1.00 equiv, 3.00 M in Et_2O) was added and the reaction was cooled to –78 °C. Trichloroisobutylsilane (3.33 mL, 20.0 mmol,

1.00 equiv) was then added quickly. The reaction was warmed to room temperature and stirred for 16 h. The reaction was then cooled down to –78 °C and LAH (4.00 M in Et_2O, 10.0 mL, 40.0 mmol, 2.00 equiv) was added dropwise. Then the reaction was warmed to room temperature, stirred for an additional 3 h, at which time the reaction was quenched by the addition of a solution of saturated aq. Rochelle's salt (15 mL) and filtered over celite. The organic layer was separated, and the aqueous layer was washed with Et_2O (3 x 10 mL), then the organic layers were combined and washed with brine (10 mL), dried over anhydrous magnesium sulfate, filtered, and then concentrated in vacuo. The crude product was purified by bulb-to-bulb distillation to yield **S4** as a clear oil. Yield: 1.78 g (55%); ^{1}H NMR (400 MHz,C_6D_6) δ 7.49 (m, 2H), 7.16 (m, 3H, obscured), 4.50 (t, *J* = 3.9 Hz, 2H), 1.75 (m, 1H), 0.91 (d, *J* = 6.6 Hz, 6H), 0.80 (dt, *J* = 7.0, 3.9 Hz, 2H). ^{13}C NMR (100 MHz,C_6D_6) δ 135.6, 133.0, 129.8, 129.1, 128.3, 127.5, 25.9, 25.6, 20.7. ^{29}Si NMR (80 MHz,C_6D_6) δ –34.0.

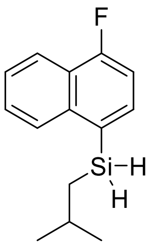

(4-Fluoronaphthalen-1-yl)(isobutyl)silane (S5):): Magnesium turnings (0.1604 g, 6.60 mmol, 1.10 equiv) and LiCl (0.2570 g, 6.00 mmol, 1.0 equiv) were added to a round-bottom flask with stir bar. The flask was flame-dried under vacuum and back filled with argon. 60 mL of THF were added to the round-bottom flask followed by the addition of DIBAL (0.60 mmol, 0.60 mL of 1.0 M solution in THF, 0.10 equiv) which was allowed to react with the magnesium turnings for 10 minutes. Then 1-bromo-4-fluoronaphthalene (1.350 g, 6.00 mmol, 1.00 equiv, purified over neutral alumina) was added and allowed to stir at room temperature for 2 h. The reaction was cooled to –78 °C and trichloro(isobutyl)silane (1.00 mL, 6.00 mmol, 1.00 equiv,) was then added at once quickly. The reaction was warmed to room temperature and stirred for 16 h. The reaction was then cooled down to –78 °C and LAH (4.00 M in Et_2O, 3.00 mL, 12.0 mmol, 2.0 equiv) was added dropwise. Then the reaction was warmed to room temperature, stirred for an additional 3 h, at which time the reaction was quenched by the addition of a solution of saturated aq. Rochelle's salt (15 mL) and filtered over celite. The organic layer was separated, and the aqueous layer was washed with Et_2O (3 x 10 mL), then the organic layers were combined and washed with brine (10 mL), dried over anhydrous magnesium sulfate, filtered, and then concentrated in vacuo. The crude product was passed through a silica plug (100% hexanes) and obtained as a 3:2 mixture of **S5** and 1-fluoronaphthalene, where the silane was carried forward to hydrolysis without further purification. ^{1}H NMR (400 MHz, $CDCl_3$) *δ* 8.20 – 8.16 (m, 1H), 7.74 (dd, *J* = 7.6, 6.1 Hz, 1H), 7.59 (ddd, *J* = 8.0, 5.6, 1.6 Hz, 2H), 7.41 (td, *J* = 8.0, 5.4 Hz, 2H), 7.20 – 7.10 (m, 1H, obscured), 4.62 (t, *J* = 4.0 Hz, 2H), 1.87 (m, 1H), 1.06 (dt, *J* = 7.0, 4.0 Hz, 2H), 1.01 (d, *J* = 6.6 Hz, 6H). ^{13}C NMR (100 MHz, $CDCl_3$) *δ* 160.7 (d, *J* = 254 Hz), 138.9 (d, *J* = 4 Hz), 135.8 (d, *J* = 9 Hz), 127.7 (d, *J* = 3 Hz), 127.5 (d, *J* = 5 Hz), 126.2 (d, *J* = 5 Hz), 125.8, 124.0, 121.4 (d, *J* = 6 Hz), 109.2 (d, *J* = 19 Hz), 26.0, 25.6, 20.8. ^{19}F NMR (376 MHz, $CDCl_3$) –120.3. ^{29}Si NMR (80 MHz, $CDCl_3$) –36.1

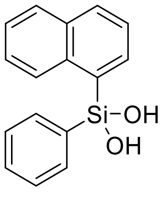

Naphthalen-1-yl(phenyl)silanediol (1a). Silanediol **1a** was synthesized according to previously reported method and matched to spectral data.[3] ^{1}H NMR (400 MHz, d^{6}-DMSO) δ 8.36 (dd, J = 8.0, 1.7 Hz, 1H), 7.99 – 7.85 (m, 3H), 7.65 – 7.62 (m, 2H), 7.53 – 7.42 (m, 3H), 7.38 – 7.30 (m, 3H), 7.18 (s, 2H).

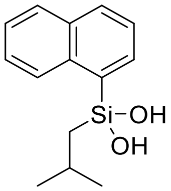

(Naphthalen-1-yl)isobutylsilanediol (1b). Pd/C (0.106 g, 0.10 mmol, 0.050 equiv, 10 wt %) was added to a solution of silane **S1** (0.216 g, 1.0 mmol, 1.0 equiv) in THF (10 mL) in a round-bottom flask. Deionized water (2.0 mmol, 2.0 equiv) was added dropwise. Evolution of H_2 gas was observed initially and the reaction was allowed to stir at room temperature for 22 h until complete consumption of the silane was observed based on TLC analysis. The Pd/C was removed through filtration over celite, and the organic layers were dried over magnesium sulfate, filtered, and then concentrated in vacuo. The crude product was purified using column chromatography (4:1 hexanes/Et_2O) to yield silanediol **1b** as a white solid (mp: 77.8 °C) (Yield: 0.702 g, 41%). ^{1}H NMR (600 MHz,C_6D_6) δ 8.50 (d, J = 8.4 Hz, 1H), 7.97 (d, J = 6.7 Hz, 1H), 7.69 (d, J = 8.1 Hz, 1H), 7.66 (d, J = 8.1 Hz, 1H), 7.36 (t, J = 7.5 Hz, 1H), 7.33 – 7.29 (m, 1H), 7.29 – 7.26 (m, 1H), 2.32 (s, 2H), 1.85 (m, 1H), 0.93 (d, J = 7.0 Hz, 2H), 0.90 (d, J = 6.6 Hz, 6H). ^{13}C NMR (100 MHz, $CDCl_3$) δ 136.7, 134.6, 134.1, 133.5, 131.0, 129.1, 128.3, 126.4, 125.8, 125.2, 26.7, 26.1, 24.2. ^{29}Si NMR (119 MHz, $CDCl_3$, Cr(acac)$_3$) δ –14.5. HRMS (ESI) m/z: calc for $C_{14}H_{18}O_2Si+HCO_2^-$ [M + HCOO]$^-$ 291.1058, found 291.1045.

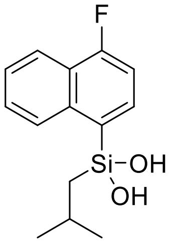

(4-Fluoronaphthalen-1-yl)(isobutyl)silanediol (1c). Pd/C (0.041 g, 0.40 mmol, 0.10 equiv, 10 wt %) was added to a solution of silane **S5** (0.411 g, 1.77 mmol, 1.0 equiv) in THF (20 mL) in a round-bottom flask. Deionized water (17.7 mmol, 10 equiv) was added dropwise. Evolution of H_2 gas was observed initially and the reaction was allowed to stir at room temperature for 1.5 h until complete consumption of the silane was observed based on TLC analysis. The Pd/C was removed through filtration through celite, and the filtrate was dried over magnesium sulfate, filtered, and then concentrated in vacuo. The crude product was purified using column chromatography (4:1 hexanes/EtOAc) to yield silanediol **1c** as a white solid (mp: 93.0 °C) (Yield: 0.152 g, 32%). ^{1}H NMR (400 MHz, $CDCl_3$) δ 8.32 (dt, J = 7.7, 2.0 Hz, 1H), 8.16 (dd, J = 7.9, 1.9 Hz, 1H), 7.87 (dd, J = 7.7, 6.1 Hz, 1H), 7.64 – 7.50 (m, 2H), 7.13 (dd, J = 10.6, 7.6 Hz, 1H), 2.82 (s, 2H), 1.87 (m, 1H), 1.01 (d, J = 7.0 Hz, 2H), 0.93 (d, J = 6.6 Hz, 6H). ^{13}C NMR (100 MHz, $CDCl_3$) δ 160.9 (d, J = 255 Hz), 138.53, 134.9 (d, J = 9 Hz), 130.0, 128.0 (d, J = 3 Hz), 127.42, 126.1 (d, J = 2 Hz), 124.0 (d, J = 15 Hz), 121.4 (d, J = 6 Hz), 109.0 (d, J = 18 Hz), 26.73, 26.13, 24.20. ^{19}F NMR (376 MHz, $CDCl_3$) δ -119.4. ^{29}Si NMR (119 MHz, $CDCl_3$) δ -14.8. HRMS (ESI) m/z: calc for $C_{14}H_{16}FO_2Si^-$ [M – H]$^-$ 263.0909, found 263.0904.

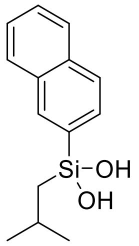

(Naphthalen-2-yl)methylsilanediol (1d). Pd/C (0.2448 g, 0.23 mmol, 0.10 equiv, 10 wt %) was added to a solution of silane **S5** (517.7 mg, 2.3 mmol, 1.0 equiv) in THF (10 mL) in a round-bottom flask. Deionized water (23 mmol, 10 equiv) was added dropwise. Evolution of H_2 gas was observed initially and the reaction was allowed to stir at room temperature for 19 h until complete consumption of the silane was observed based on TLC analysis. The Pd/C was removed through filtration over celite and the organics were dried over magnesium sulfate filtered, and then concentrated in vacuo*.* The crude product was purified using column chromatography (4:1 hexanes/Et_2O) to yield silanediol **1d** as a white solid (mp: 93.6 °C) (Yield: 0.390 g, 67%). ^{1}H NMR (400 MHz,C_6D_6) δ 8.22 (s, 1H), 7.71 (dd, *J* = 6.1, 3.1 Hz, 3H), 7.67 – 7.61 (m, 1H), 7.32 – 7.23 (m, 2H), 2.11 (d, *J* = 10.5 Hz, 2H), 1.89 (m, 1H), 0.97 (d, *J* = 6.6 Hz, 6H), 0.85 (d, *J* = 7.0 Hz, 2H). ^{13}C NMR (100 MHz,C_6D_6) δ 135.1, 134.5, 134.4, 133.1, 129.9, 128.3, 127.1, 126.6, 125.9, 25.9, 25.5, 24.0. Si NMR (119 MHz, $CDCl_3$, Cr(acac)$_3$) δ –17.03. HRMS (ESI) m/z: calc for $C_{14}H_{18}O_2Si$+$HCOO^-$ [M + HCOO]$^-$ 291.1058, found 291.1051.

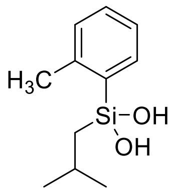

isobutyl(o-tolyl)silanediol (1e). Pd/C (72.4 mg, 0.68 mmol, 0.10 equiv, 10 wt %) was added to a solution of silane **S7** (1.423 g, 6.76 mmol, 1.0 equiv) in THF (45 mL) in a round-bottom flask. Deionized water (1.2 mL, 67.6 mmol, 10 equiv) was added dropwise. Evolution of H_2 gas was observed initially and the reaction was allowed to stir at room temperature for 19 h until complete consumption of the silane was observed based on TLC analysis. The Pd/C was removed through filtration and the filtrate was dried over magnesium sulfate, filtered, and then concentrated in vacuo*.* The crude product was purified using column chromatography (4:1 hexanes/Et_2O) to yield silanediol **1e** as a white solid (mp: 80.6 °C) (1.01 g, 71%). ^{1}H NMR (400 MHz,C_6D_6) 7.77 (dd, *J* = 7.3, 1.7 Hz, 1H), 7.19 (dd, obscured, 2H), 7.14 (t, *J* = 7.3 1H), 7.04 (d, *J* = 7.5 Hz, 1H), 2.44 (s, 3H), 1.83 (m, 1H), 0.93 (d, *J* = 6.6 Hz, 6H), 0.78 (d, *J* = 7.1 Hz, 2H). ^{13}C NMR (100 MHz,C_6D_6) δ 143.7, 135.7, 135.5, 130.5, 130.2, 125.2, 26.6, 26.2, 24.3, 22.9. ^{29}Si NMR (119 MHz,C_6D_6) δ –15.57. HRMS (ESI) m/z: calc for $C_{11}H_{18}O_2Si$+$HCOO^-$ [M + HCOO]$^-$ 255.1058 found 255.1052.

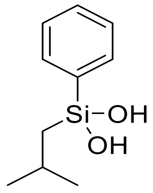

Phenylmethylsilanediol (1f). Pd/C (0.1019 g, 0.95 mmol, 0.10 equiv, 10 wt %) was added to a solution of silane **S6** (1.5610 g, 9.50 mmol, 1.0 equiv) in THF (60 mL) in a round-bottom flask. Deionized water (95.0 mmol, 10 equiv) was added dropwise. Evolution of H_2 gas was observed initially and the reaction was allowed to stir at room temperature for 19 h until complete consumption of the silane was observed based on TLC analysis. The Pd/C was removed through filtration and the filtrate was dried over magnesium sulfate, filtered, and then concentrated in vacuo*.* The crude product was purified using column chromatography

(4:1 hexanes/Et_2O) to yield silanediol **1f** as a white solid (mp: 81.6 °C) (Yield: 0.141 g, 8%). ^{1}H NMR (400 MHz,C_6D_6) δ 7.64 (q, 2H), 7.20 (t, 4H), 2.40 (s, 2H), 1.86 (m, 1H), 0.94 (d, *J* = 6.6 Hz, 6H), 0.77 (d, *J* = 7.0 Hz, 2H). ^{13}C NMR (150 MHz,C_6D_6) δ 134.3, 134.2, 130.20, 130.17, 26.2, 25.8, 24.3. ^{29}Si NMR (119 MHz, $CDCl_3$, Cr(acac)$_3$) δ –16.80.

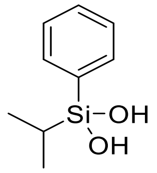

isopropyl(phenyl)silanediol (1g). Silanediol **1g** was synthesized according to previously reported method and matched to spectral data.[2] ^{1}H NMR (400 MHz, $CDCl_3$) δ 7.70 – 7.63 (m, 2H), 7.47 – 7.42 (m, 1H), 7.42 – 7.37 (m, 2H), 2.49 (s, 2H), 1.18 – 1.02 (m, 1H, overlapping), 1.08 (m, 6H, overlapping).

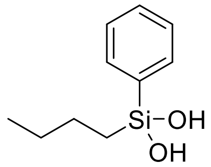

butyl(phenyl)silanediol (1h). Trichlorophenylsilane (0.480 mL, 3.0 mmol, 1.0 equiv) was added via syringe to a flame dried argon purged round bottom flask. 50 mL dry Et_2O was added. Then n-BuLi (1.2 mL, 3.0 mmol, 1 equiv., 2.5 M in hexanes) was added dropwise, a white precipitate formed immediately on addition. The reaction was left to mix for 1 hour, then LAH (1.50 mL, 6.0 mmol, 2.0 equiv., 4.0 M in Et_2O) was added dropwise. Reaction was left to mix an additional 2.5 hours. Then the reaction was quenched by the addition of a solution of saturated aq. Rochelle's salt (15 mL) and filtered over celite. The organic layer was separated, and the aqueous layer was washed with Et_2O (3 x 10 mL), then the organic layers were combined and washed with brine (10 mL), dried over anhydrous magnesium sulfate, filtered, and then concentrated at 250 torr and 34 °C to avoid loss of the low boiling silane. The reaction contents were then taken up in 40.0 mL wet THF, and Pd/C (49.3 mg, 10wt %) was added, followed by the addition of deionized water (0.100 mL, 6.0 mmol, 2.0 equiv). The flask was left to mix for 2 hours, then filtered through celite, dried over magnesium sulfate, and concentrated. The crude product was purified by column chromatography (7:3 hexanes/EtOAc) to yield silanediol **1h** as a white powder matched to previous spectra (63.0 mg, 16% yield).[10] ^{1}H NMR (400 MHz, $CDCl_3$) δ 7.68 – 7.63 (m, 2H), 7.47 – 7.35 (m, 3H), 2.53 (s, 2H), 1.48 – 1.30 (m, 4H), 0.92 – 0.85 (m, 5H).

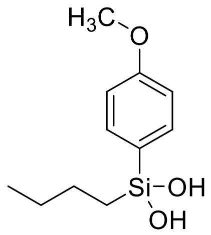

butyl(4-methoxyphenyl)silanediol (1i). Triethoxy(4-methoxyphenyl)silane (0.530 mL, 2.0 mmol, 1.0 equiv) was added via syringe to a flame dried argon purged round bottom flask. 50 mL dry Et_2O was added. Then n-BuLi (0.80 mL, 2.0 mmol, 1 equiv., 2.5 M in hexanes) was added dropwise, a white precipitate formed immediately on addition. The reaction was left to mix for 1 hour, then LAH (1.00 mL, 4.0 mmol, 2.0 equiv., 4.0 M in Et_2O) was added dropwise. Reaction was left to mix an additional 2.0 hours. Then the reaction was quenched by the addition of a solution of saturated aq. Rochelle's salt (15 mL) and filtered over celite. The organic layer was separated, and the aqueous layer was washed with Et_2O (3 x 10 mL), then the organic layers were combined and washed with brine (10 mL), dried over anhydrous magnesium sulfate, filtered, and then concentrated at 250 torr and 34 °C to avoid loss of the low boiling silane. The reaction contents were then taken up in 40.0 mL wet THF, and Pd/C (49.3 mg, 10wt %) was added, followed

by the addition of deionized water (0.072 mL, 4.0 mmol, 2.0 equiv). The flask was left to mix for 1 hour, then filtered through celite, dried over magnesium sulfate, and concentrated. The crude product was purified by column chromatography (7:3 hexanes/EtOAc) to yield silanediol **1i** as a white powder (219.2 mg 48% yield). ^{1}H NMR (400 MHz, $CDCl_3$) δ 7.58 (d, J = 8.7 Hz, 2H), 6.93 (d, J = 8.6 Hz, 2H), 3.82 (s, 3H), 2.61 (s, 2H), 1.46 – 1.27 (m, 4H), 0.87 (t, J = 7.1 Hz, 5H). ^{13}C NMR (100 MHz,C_6D_6) δ 161.5, 135.5, 126.7, 113.8, 55.2, 26.3, 25.1, 14.8, 13.9. HRMS (ESI) m/z: calc for $C_{11}H_{18}O_3Si$+$HCOO^-$ [M + HCOO]$^-$ 271.1007 found 271.0998.

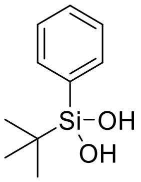

tert-butyl(phenyl)silanediol (1j). Silanediol **1j** was synthesized from tert-butyldichloro(phenyl)silane according to previously reported method and matched spectral data.[2,11] ^{1}H NMR (400 MHz, $CDCl_3$) δ 7.68 (m, 2H), 7.47 – 7.35 (m, 3H), 2.44 (s, 2H), 1.00 (s, 9H).

9.1, Characterization data for siloxanols (3aa-mb)

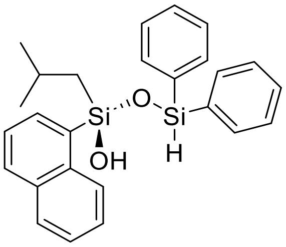

(*S*)-1-isobutyl-1-(naphthalen-1-yl)-3,3-diphenyldisiloxan-1-ol (**3ba**). Synthesized according to the general procedure for desymmetrization of silanediols using silanediol **1b** (1.00 equiv, 0.20 mmol, 0.0492 g), and chlorosilane **2a** (1.00 equiv, 0.20 mmol, 39.1 µL). For this product a modified work-up was employed where the reaction was first diluted with 5.0 mL Et_2O, then 1.0 mL 10% citric acid solution was used to quench the reaction. The organic and aqueous layers were separated immediately and then the organic layer was dried over magnesium sulfate. The product was purified using column chromatography (9:1 hexanes/Et_2O) and isolated as a clear oil (62.7 mg, 73%). Enantiomeric ratios were determined by HPLC with Diacel CHIRALPAK ® OD-H column (1% IPA/hexanes) 1 mL/min, **3ba1 =** 19.1min, **3ba2** = 21.6 min 88:12 er.^{1}H NMR (400 MHz,C_6D_6) δ 8.48 (d, *J* = 8.1 Hz, 1H), 8.02 (d, 1H), 7.77 – 7.58 (m, 6H), 7.34 – 7.22 (m, 3H), 7.16 (s, obscured, 6H), 5.95 (s, 1H), 2.14 (s, 1H), 1.91 (m, 1H), 1.04 (d, *J* = 7.0 Hz, 2H), 0.88 (dd, *J* = 10.8, 6.6 Hz, 6H). ^{13}C NMR (100 MHz,C_6D_6) δ 137.3, 135.79, 135.76, 135.1, 134.82, 134.76, 134.0, 131.1, 130.56, 130.53, 129.3, 128.9, 126.4, 125.8, 125.4, 27.6, 26.2, 24.4. ^{29}Si NMR (80 MHz,C_6D_6) δ –21.31, –21.79. UV/Vis (hexanes/IPA 99:1) λ_{max} (*ε)* 200, 282 nm.

Racemic standard for **3ba**

<Chromatogram>

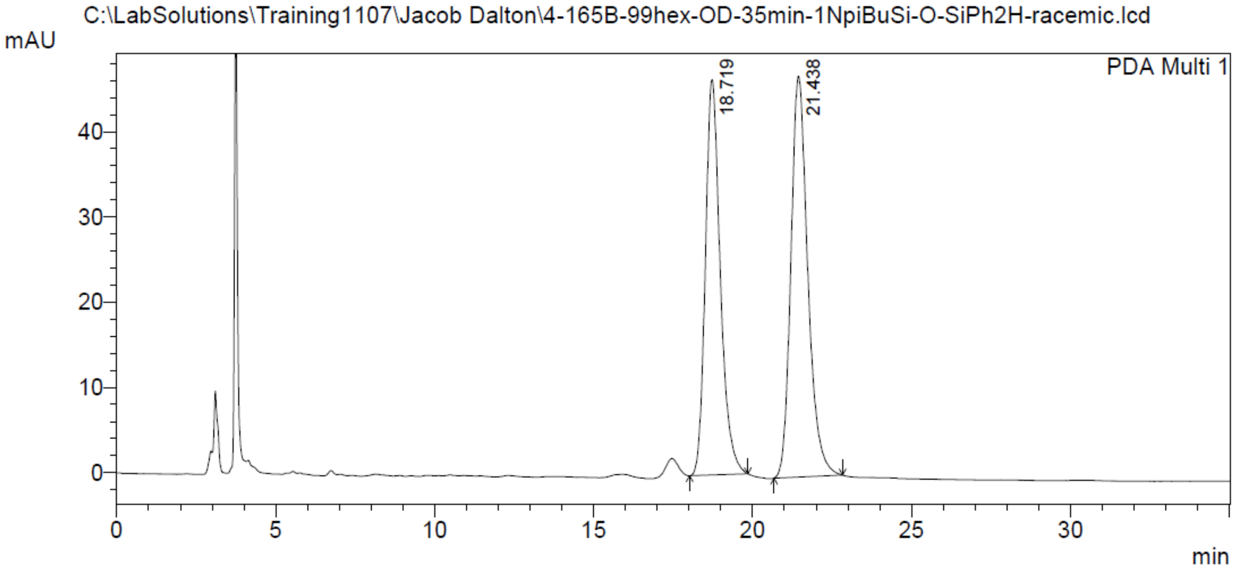

1 PDA Multi 1/283nm 4nm

< Peak Table >

PeakTable C:\LabSolutions\Training1107\Jacob Dalton\4-165B-99hex-OD-35min-1NpiBuSi-O-SiPh2H-racemic.lcd
PDA Ch1 283nm 4nm

Peak#	Ret. Time	Area	Height	Area %	Height %
1	18.719	1515473	46356	46.559	49.650
2	21.438	1739509	47009	53.441	50.350
Total		3254982	93365	100.000	100.000

Enantiomerically enriched **3ba** (88:12 er),

<Chromatogram>

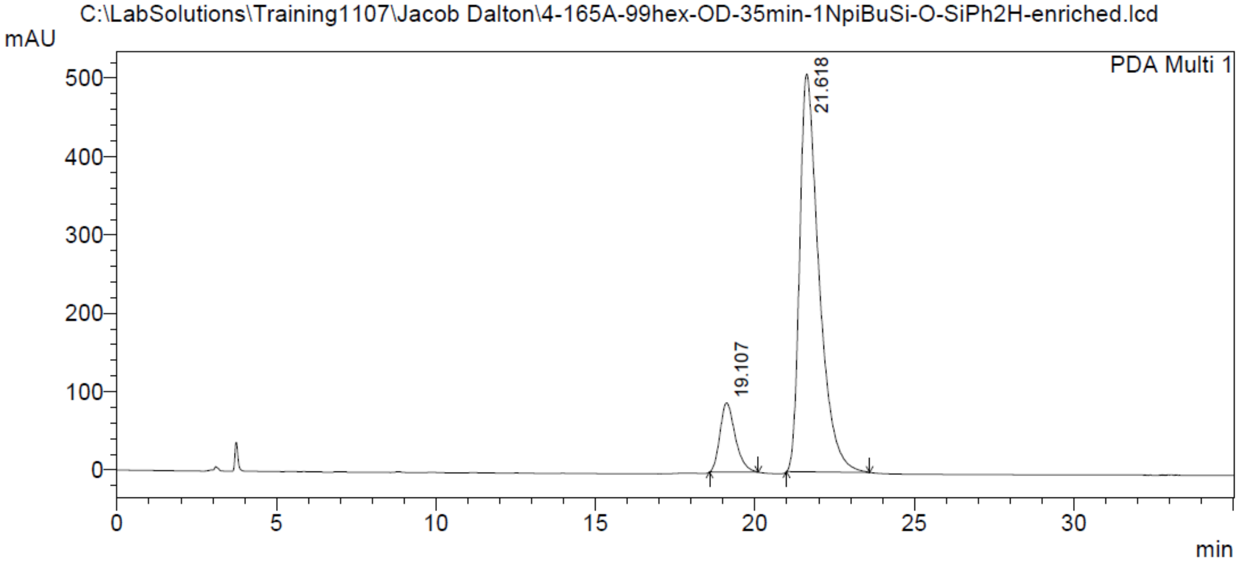

1 PDA Multi 1/282nm 4nm

< Peak Table >

PeakTable C:\LabSolutions\Training1107\Jacob Dalton\4-165A-99hex-OD-35min-1NpiBuSi-O-SiPh2H-enriched.lcd
PDA Ch1 282nm 4nm

Peak#	Ret. Time	Area	Height	Area %	Height %
1	19.107	2941575	88222	12.110	14.810
2	21.618	21348836	507464	87.890	85.190
Total		24290410	595686	100.000	100.000

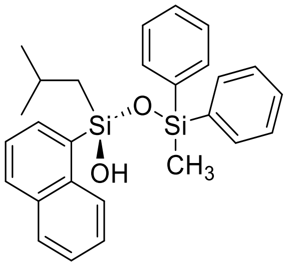

(*S*)-1-isobutyl-3-methyl-1-(naphthalen-1-yl)-3,3-diphenyldisiloxan-1-ol (**3bb**). Synthesized according to the general procedure for desymmetrization of silanediols using silanediol **1b** (1.00 equiv, 0.10 mmol, 0.0246 g), and chlorosilane **2b** (1.00 equiv, 0.10 mmol, 20.8 µL). The product was purified using column chromatography (9:1 hexanes/EtOAc) and isolated as a clear oil (40.1 mg, 91%). Enantiomeric ratios were determined by HPLC with Diacel CHIRALPAK ® AD-H column (2% IPA/hexanes) 1.0 mL/min, **3bb1 =** 6.5 min, **3bb2** = 7.2 min, 98:2 er. ^{1}H NMR (600 MHz,C_6D_6) δ 8.50 (d, *J* = 8.3 Hz, 1H), 8.02 (dd, *J* = 6.7, 1.4 Hz, 1H), 7.71-7.67 (m, 5H), 7.65 (dd, *J* = 8.0, 1.6 Hz, 1H), 7.59 – 7.56 (m, 1H), 7.28 (dddd, *J* = 14.7, 9.2, 6.8, 3.5 Hz, 3H), 7.21 – 7.17 (m, 5H), 2.23 (s, 1H), 1.87 (m, 1H), 1.03 (d, *J* = 7.0 Hz, 2H), 0.86 (dd, *J* = 12.6, 6.5 Hz, 6H), 0.64 (s, 3H). ^{13}C NMR (100 MHz,C_6D_6) δ 138.0, 137.8, 137.3, 135.5, 134.7, 134.54, 134.51, 134.0, 131.0, 130.04, 130.02, 129.2, 129.0, 128.19, 128.18, 126.3, 125.8, 125.4, 27.9, 26.2, 24.4, –0.42. HRMS (ESI) m/z: calc for $C_{27}H_{29}O_2Si_2$ $[M]^-$ 441.1711, found 441.1695 UV/Vis (hexanes/IPA 95:5) λ_{max} (*ε)* 207, 282 nm.

Racemic standard for **3bb**

<Chromatogram>

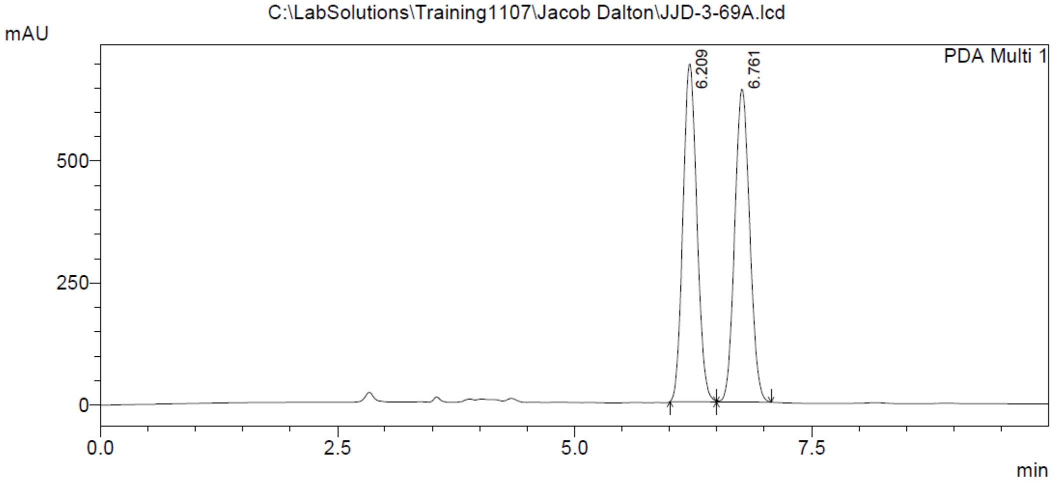

1 PDA Multi 1/283nm 4nm

< Peak Table >

PeakTable C:\LabSolutions\Training1107\Jacob Dalton\JJD-3-69A.lcd

PDA Ch1 283nm 4nm

Peak#	Ret. Time	Area	Height	Area %	Height %
1	6.209	7069279	692527	50.017	51.922
2	6.761	7064471	641263	49.983	48.078
Total		14133750	1333790	100.000	100.000

Enantiomerically enriched **3bb** (97:3 er),

<Chromatogram>

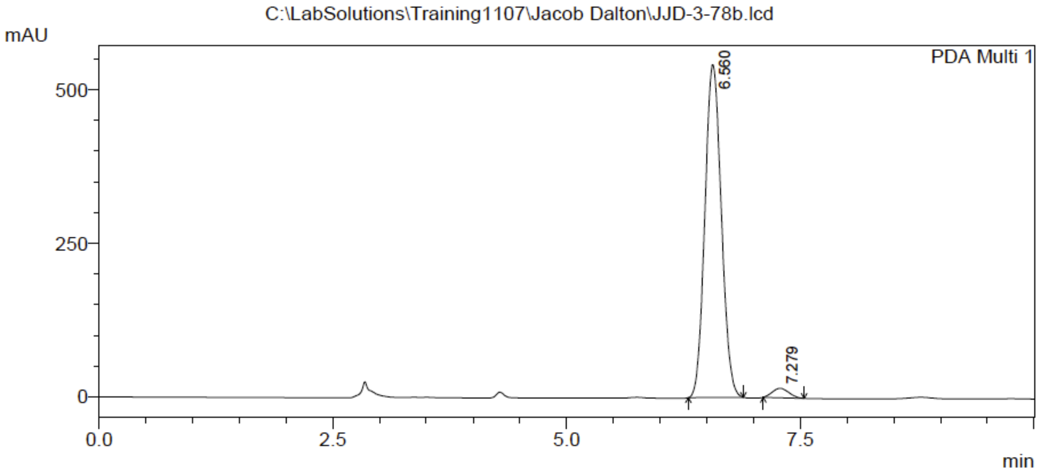

1 PDA Multi 1/282nm 4nm

< Peak Table >

PeakTable C:\LabSolutions\Training1107\Jacob Dalton\JJD-3-78b.lcd

PDA Ch1 282nm 4nm

Peak#	Ret. Time	Area	Height	Area %	Height %
1	6.560	6629441	541743	97.255	97.269
2	7.279	187121	15213	2.745	2.731
Total		6816562	556956	100.000	100.000

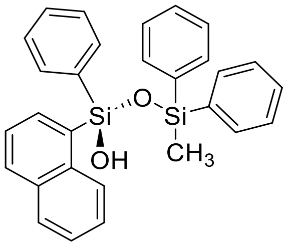

(*S*)-3-methyl-1-(naphthalen-1-yl)-1,3,3-triphenyldisiloxan-1-ol (**3ab**). Synthesized according to the general procedure for desymmetrization of silanediols using silanediol **1a** (1.00 equiv, 0.13 mmol, 0.0351 g), and chlorosilane **2b** (1.00 equiv, 0.13 mmol, 27.7 μL). The product was purified using column chromatography (9:1 hexanes/EtOAc) and isolated as a clear oil (50.9 mg, 83%). Enantiomeric ratios were determined by HPLC with Diacel CHIRALPAK ® AD-H column (2% IPA/hexanes) 1.0 mL/min, **3ab1 =** 22.1 min, **3ab2** = 24.1 min 70:30 er. ^{1}H NMR (400 MHz CDCl$_3$) 8.19 (d, *J* = 8.4 Hz, 1H), 7.92 (d, *J* = 7.3 Hz, 2H), 7.86 – 7.81 (m, 1H), 7.65 – 7.59 (m, 2H), 7.58 – 7.51 (m, 4H), 7.47 – 7.34 (m, 5H), 7.33 – 7.27 (m, 7H), 2.79 (s, 1H), 0.60 (s, 3H). ^{13}C (100 MHz, CDCl$_3$) *δ* 137.1, 136.7, 135.7, 135.4, 134.2, 134.1, 133.3, 132.7, 131.1, 130.2, 129.7, 128.8, 128.7, 127.9, 127.8, 126.1, 125.5, 125.0, –0.80. ^{29}Si (80 MHz, CDCl$_3$) *δ* –8.86, –34.89. HRMS (ESI) m/z: calc for $C_{29}H_{25}O_2Si_2$ $[M]^-$ 461.1399, found 461.1396. UV/Vis (hexanes/IPA 98:2) λ_{max} (*ε)* 223, 200, 656, 284, 294 nm.

Racemic standard for **3ab**

<Chromatogram>

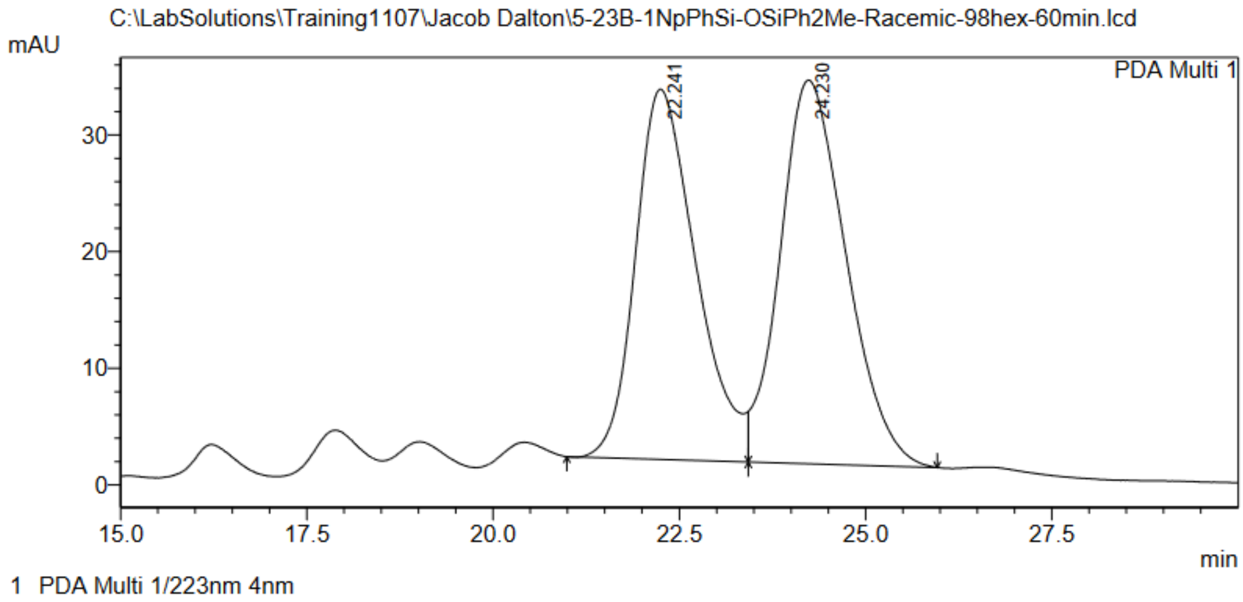

< Peak Table >

PeakTable C:\LabSolutions\Training1107\Jacob Dalton\5-23B-1NpPhSi-OSiPh2Me-Racemic-98hex-60min.lcd

PDA Ch1 223nm 4nm

Peak#	Ret. Time	Area	Height	Area %	Height %
1	22.241	1800296	31744	47.024	49.113
2	24.230	2028168	32891	52.976	50.887
Total		3828465	64635	100.000	100.000

Enantiomerically enriched **3ab** (70:30 er),

<Chromatogram>

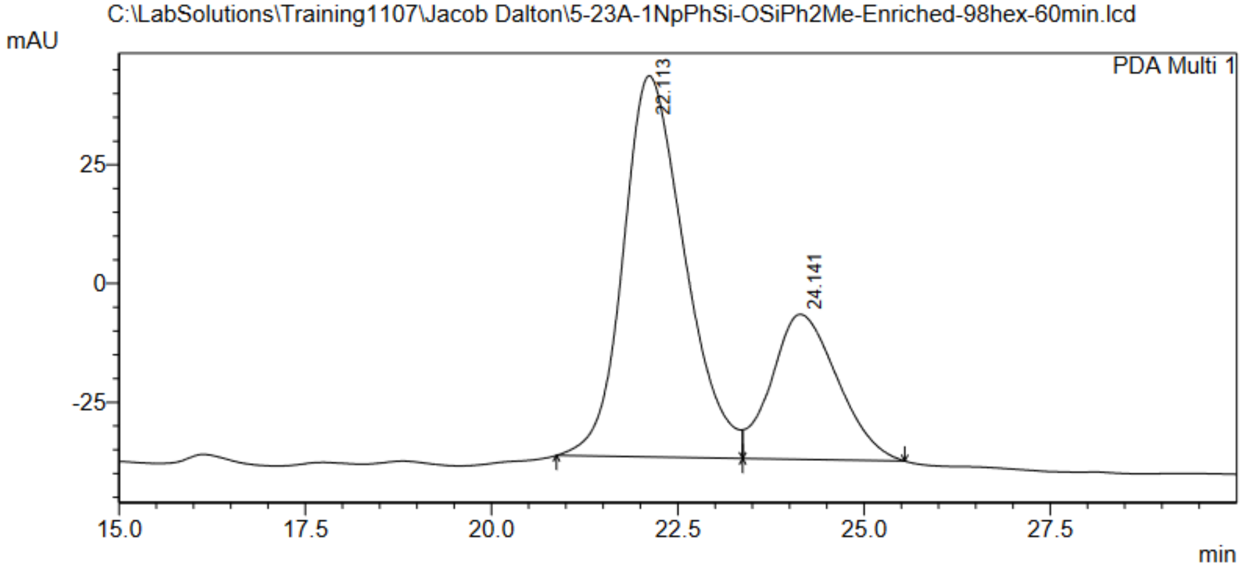

1 PDA Multi 1/223nm 4nm

< Peak Table >

PeakTable C:\LabSolutions\Training1107\Jacob Dalton\5-23A-1NpPhSi-OSiPh2Me-Enriched-98hex-60min.lcd
PDA Ch1 223nm 4nm

Peak#	Ret. Time	Area	Height	Area %	Height %
1	22.113	4612671	80232	70.975	72.437
2	24.141	1886312	30530	29.025	27.563
Total		6498983	110762	100.000	100.000

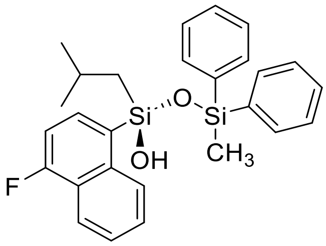

(*S*)-1-(4-fluoronaphthalen-1-yl)-1-isobutyl-3-methyl-3,3-diphenyldisiloxan-1-ol (**3cb**). Synthesized according to the general procedure for desymmetrization of silanediols using silanediol **1c** (1.00 equiv, 0.15 mmol, 0.0273 g), and chlorosilane **2b** (1.00 equiv, 0.15 mmol, 31.5 µL). The product was purified using column chromatography (9:1 hexanes/EtOAc) and isolated as a clear oil (44.4 mg, 78%) Enantiomeric ratios were determined by HPLC with Diacel CHIRALPAK ® AD-H column (1% IPA/hexanes) 0.5 mL/min,33 °C **3cb1 =** 35.3 min, **3cb2** = 38.0 min 92:8 er. ^{1}H NMR (400 MHz, $CDCl_3$) δ 8.28 (d, *J* = 8.4 1H), 8.19 – 8.12 (m, 1H), 7.82 (dd, *J* = 7.7, 6.2 Hz, 1H), 7.65 – 7.29 (m, 12H), 7.09 (dd, *J* = 10.7, 7.7 Hz, 1H), 2.51 (s, 1H), 1.81 (m, 1H), 0.98 (dd, *J* = 7.0, 1.1 Hz, 2H), 0.87 (dd, *J* = 8.6, 6.6 Hz, 6H), 0.66 (s, 3H). ^{13}C NMR (100 MHz, $CDCl_3$) δ 160.7 (d, *J* = 254 Hz), 138.4 (d, *J* = 4 Hz), 137.4, 137.3, 134.7 (d, *J* = 9 Hz), 134.1 (d, *J* = 4 Hz), 130.7 (d, *J* = 5 Hz), 129.9 (d, *J* = 3 Hz), 129.2, 128.3 (d, *J* = 3 Hz), 128.0 (d, *J* = 2 Hz), 127.1, 125.9 (d, *J* = 2 Hz), 125.5, 123.9, 123.8, 121.2 (d, *J* = 6 Hz), 108.9, 108.7, 29.9, 27.6, 26.1, 24.2f. ^{19}F NMR (376 MHz, $CDCl_3$) δ -119.99. ^{29}Si NMR (80 MHz, $CDCl_3$) δ -9.78, -22.94. HRMS (ESI) m/z: calc for $C_{27}H_{28}FO_2Si_2^-$ [M-H]$^-$ 459.1617, found 459.1613. UV/Vis (hexanes/IPA 99:1) λ_{max} (*ε)* 225, 198, 285, 656, 440 nm.

Racemic standard for **3cb**

<Chromatogram>

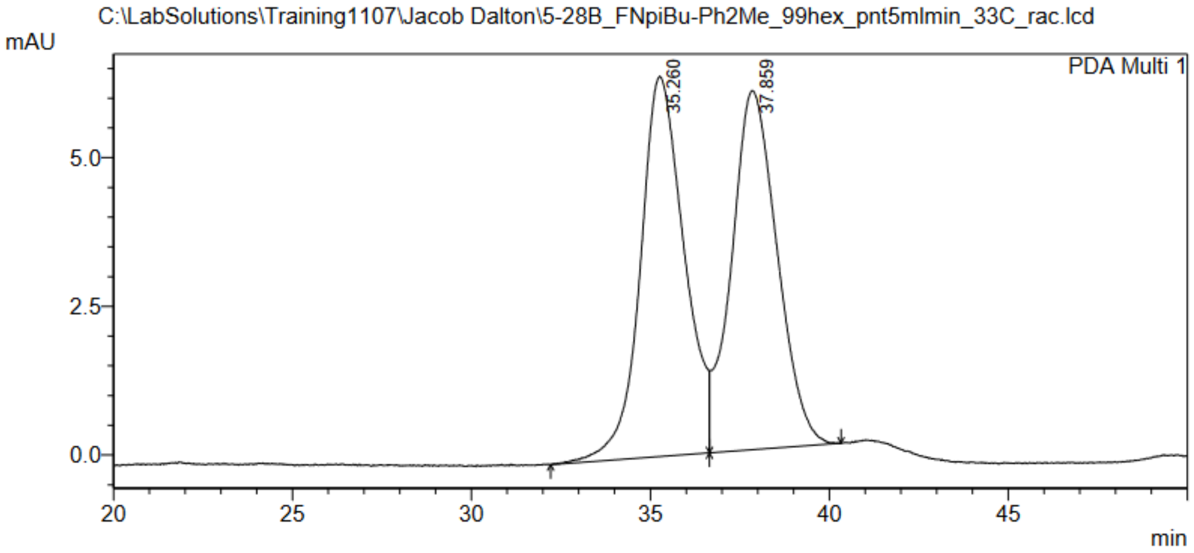

1 PDA Multi 1/285nm 4nm

< Peak Table >

PeakTable C:\LabSolutions\Training1107\Jacob Dalton\5-28B_FNpiBu-Ph2Me_99hex_pnt5mlmin_33C_rac.lcd
PDA Ch1 285nm 4nm

Peak#	Ret. Time	Area	Height	Area %	Height %
1	35.260	545393	6401	50.342	51.451
2	37.859	537986	6040	49.658	48.549
Total		1083379	12441	100.000	100.000

Enantiomerically enriched **3cb** (92:8 er)

<Chromatogram>

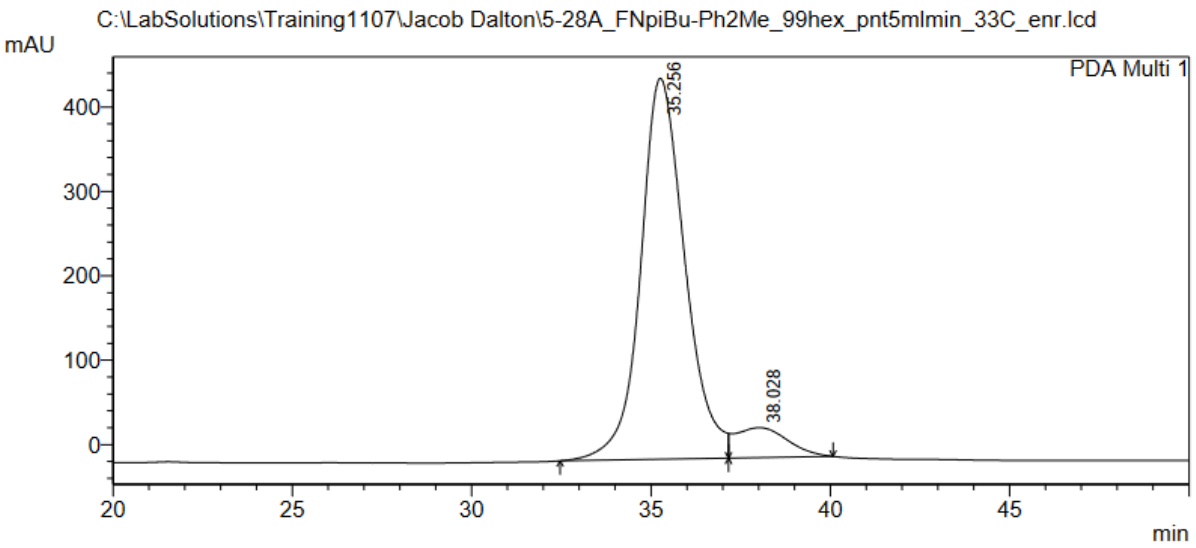

1 PDA Multi 1/225nm 4nm

< Peak Table >

PeakTable C:\LabSolutions\Training1107\Jacob Dalton\5-28A_FNpiBu-Ph2Me_99hex_pnt5mlmin_33C_enr.lcd
PDA Ch1 225nm 4nm

Peak#	Ret. Time	Area	Height	Area %	Height %
1	35.256	38732421	451284	91.513	92.713
2	38.028	3591985	35470	8.487	7.287
Total		42324406	486754	100.000	100.000

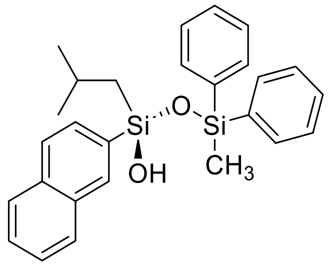

(*S*)-1-isobutyl-3-methyl-1-(naphthalen-2-yl)-3,3-diphenyldisiloxan-1-ol (**3db**). Synthesized according to the general procedure for desymmetrization of silanediols at 2.5x scale using silanediol **1d** (1.00 equiv, 0.25 mmol, 0.0620 g), and chlorosilane **2b** (1.00 equiv, 0.25 mmol, 52.0 µL). The product was purified using column chromatography (9:1 hexanes/EtOAc) and isolated as a clear oil (82.2 mg, 74%). Enantiomeric ratios were determined by HPLC with Diacel CHIRALPAK ® AD-H column (5% IPA/hexanes) 1.0 mL/min, **3db1 =** 12.0 min, **3db2** = 13.1 min, 85:15 er. ^{1}H NMR (400 MHz,C_6D_6) δ 8.19 (s, 1H), 8.14 (s, 1H), 7.69 (m, 9H), 7.26 (m, 2H), 2.03 (s, 1H), 1.93 (m, 1H), 0.94 (ddd, J = 7.4, 5.4, 1.8 Hz, 10H), 0.67 (s, 3H).^{13}C NMR (150 MHz,C_6D_6) δ 137.9, 137.8, 135.3 134.7, 134.6, 134.4, 134.3, 134.3, 133.3, 130.0, 129.9, 129.9, 129.8, 128.6, 128.2, 128.0, 127.95, 127.8, 127.4, 126.7, 126.0, 65.8, 26.5, 26.2, 24.2. ^{29}Si NMR (80 MHz,C_6D_6) δ –3.64, –21.30. UV/Vis (hexanes/IPA 95:5) λ_{max} (ε) 201, 212, 277 nm.

Racemic Standard for **3db**

<Chromatogram>

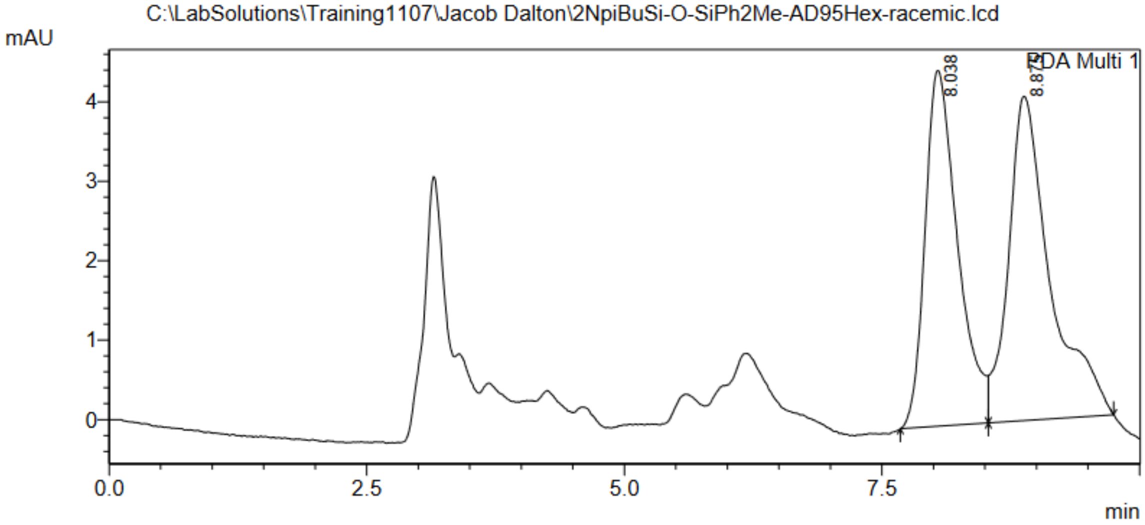

1 PDA Multi 1/282nm 4nm

< Peak Table >

PeakTable C:\LabSolutions\Training1107\Jacob Dalton\2NpiBuSi-O-SiPh2Me-AD95Hex-racemic.lcd

PDA Ch1 282nm 4nm

Peak#	Ret. Time	Area	Height	Area %	Height %
1	8.038	98981	4481	46.385	52.322
2	8.875	114408	4083	53.615	47.678
Total		213389	8564	100.000	100.000

Enantiomerically enriched **3db** (85:15 er)

<Chromatogram>

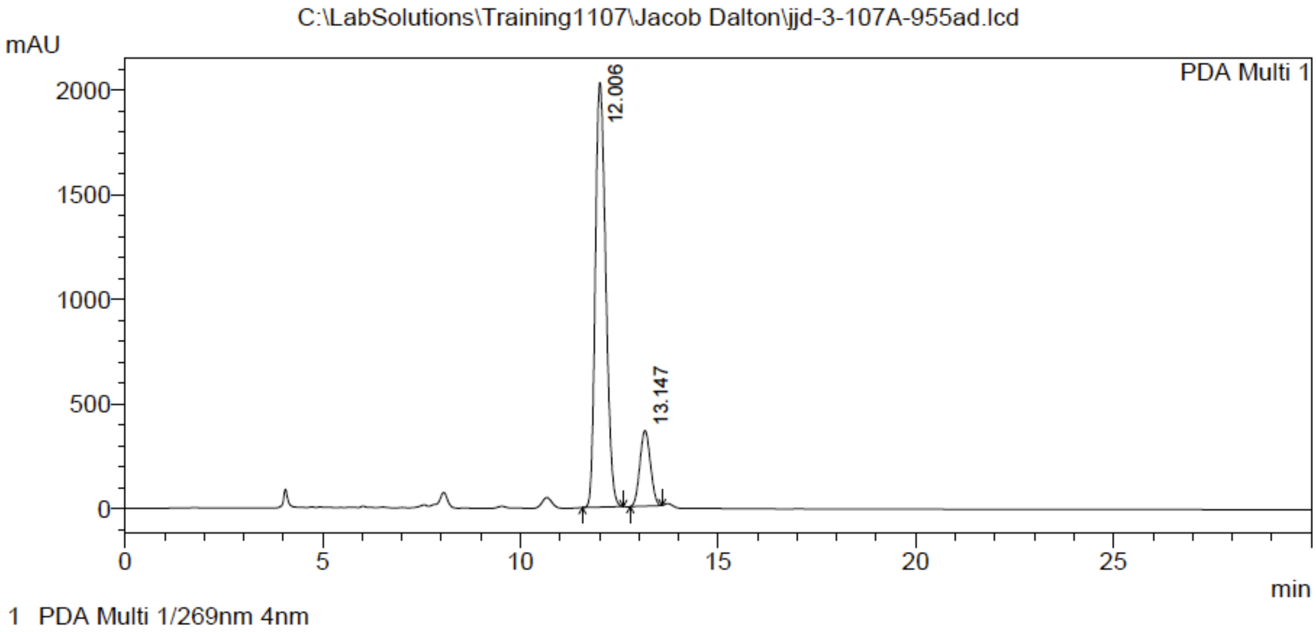

< Peak Table >

PeakTable C:\LabSolutions\Training1107\Jacob Dalton\jjd-3-107A-955ad.lcd

PDA Ch1 269nm 4nm

Peak#	Ret. Time	Area	Height	Area %	Height %
1	12.006	37172957	2027983	84.763	84.881
2	13.147	6682089	361219	15.237	15.119
Total		43855046	2389203	100.000	100.000

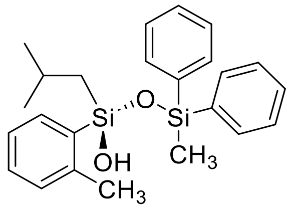

(*S*)-1-isobutyl-3-methyl-3,3-diphenyl-1-(o-tolyl)disiloxan-1-ol (**3eb**). Synthesized according to the general procedure for desymmetrization of silanediols using silanediol **1e** (1.00 equiv, 0.25 mmol, 0.0453 g), and chlorosilane **2b** (1.00 equiv, 0.25 mmol, 52.0 μL). The product was purified using column chromatography (9:1 hexanes/EtOAc) and isolated as a clear oil (74.5 mg, 82%). Enantiomeric ratios were determined by HPLC with Diacel CHIRALPAK ® AD-H column (1% IPA/hexanes) 1.0 mL/min, **3eb1 =** 13.6 min, **3eb2** = 14.8 min, 87:13 er. ^{1}H NMR (400 MHz,C_6D_6) δ 7.83 (d, *J* = 1.6 Hz, 1H), 7.71 – 7.63 (m, 4H), 7.22 – 7.16 (m, 5H), 7.09 (t, *J* = 7.3 Hz, 1H), 7.01 (d, *J* = 7.5 Hz, 1H), 2.42 (s, 3H), 1.91 (s, 1H), 1.83 (m, 1H), 0.88 (dd, *J* = 7.3, 5.3 Hz, 8H), 0.66 (s, 2H). ^{13}C NMR (100 MHz,C_6D_6) δ 143.6, 138.0, 137.9, 135.9, 135.4, 134.5, 134.5, 130.4, 130.2, 130.03, 130.01, 128.3, 128.2, 128.1, 127.8, 125.3, 32.0, 27.5, 26.24, 26.17, 24.4, 23.1, 23.0, 14.4, 1.42, –0.4. ^{29}Si NMR (80 MHz,C_6D_6) δ –10.43, –23.56. HRMS (ESI) m/z: calc for $C_{24}H_{29}O_2Si_2^-$ $[M]^-$ 405.1712 found 405.1712, UV/Vis (hexanes/IPA 99:1) λ_{max} (*ε)* 217, 198, 264 nm.

Racemic standard for **3eb**

<Chromatogram>

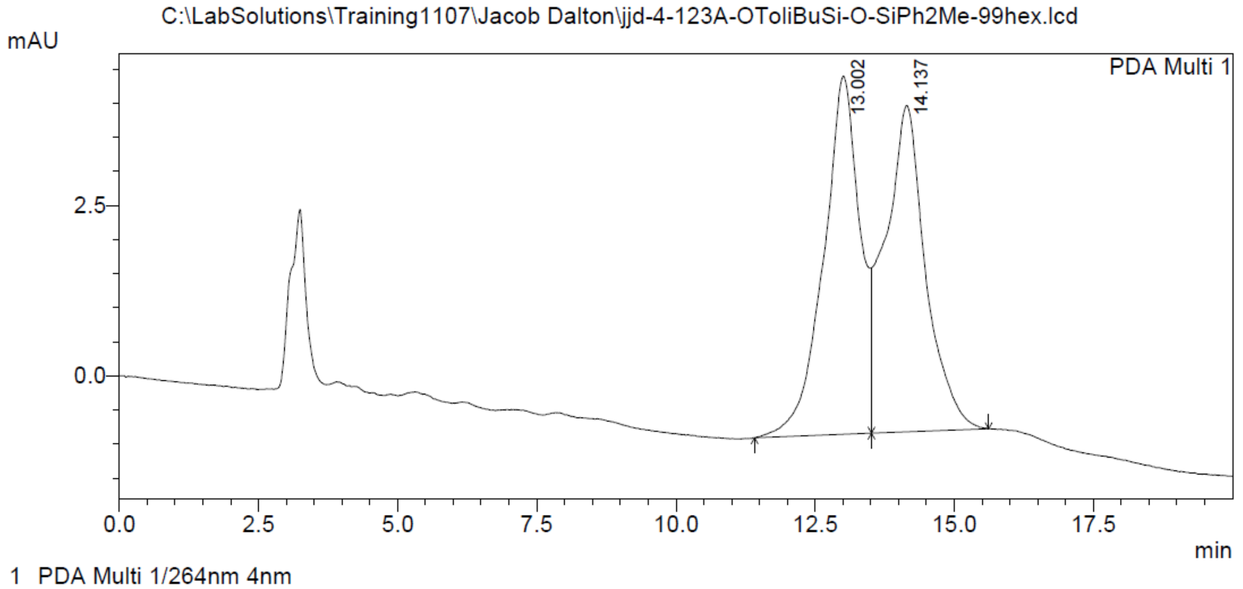

1 PDA Multi 1/264nm 4nm

< Peak Table >

PeakTable C:\LabSolutions\Training1107\Jacob Dalton\jjd-4-123A-OToliBuSi-O-SiPh2Me-99hex.lcd

PDA Ch1 264nm 4nm

Peak#	Ret. Time	Area	Height	Area %	Height %
1	13.002	240280	5255	49.761	52.314
2	14.137	242584	4790	50.239	47.686
Total		482864	10045	100.000	100.000

Enantiomerically enriched **3eb** (87:13 er)

<Chromatogram>

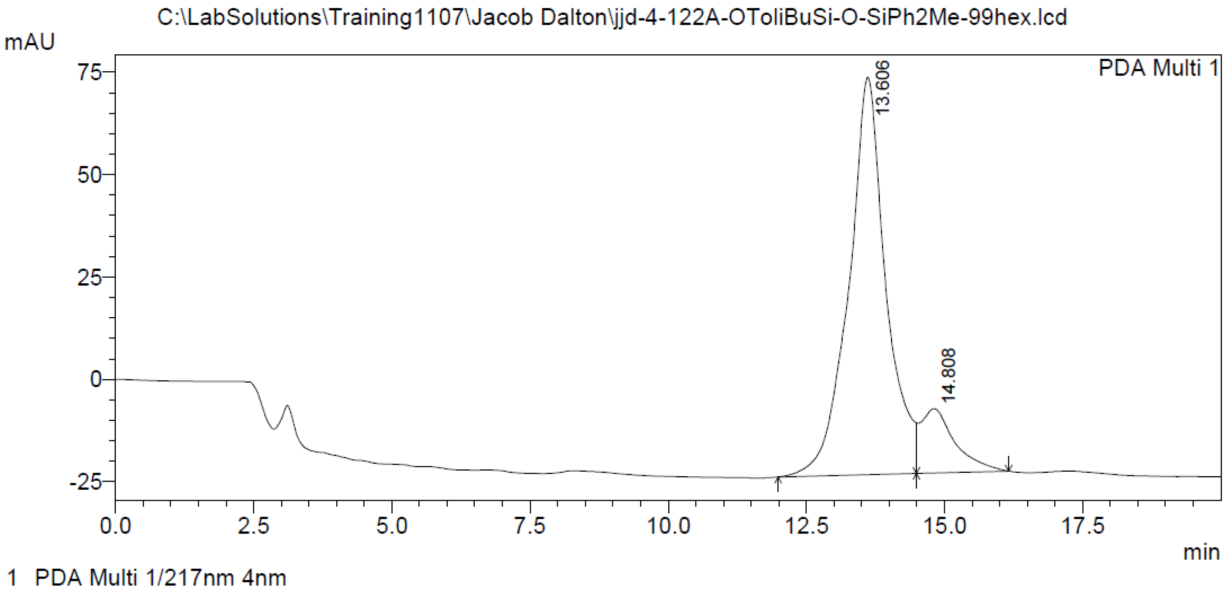

< Peak Table >

PeakTable C:\LabSolutions\Training1107\Jacob Dalton\jjd-4-122A-OToliBuSi-O-SiPh2Me-99hex.lcd

PDA Ch1 217nm 4nm

Peak#	Ret. Time	Area	Height	Area %	Height %
1	13.606	4537138	97127	86.950	86.073
2	14.808	680938	15715	13.050	13.927
Total		5218076	112842	100.000	100.000

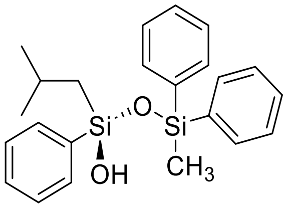

(*S*)-1-isobutyl-3-methyl-1,3,3-triphenyldisiloxan-1-ol (**3fb**). Synthesized according to the general procedure for desymmetrization of silanediols using silanediol **1f** (1.00 equiv, 0.25 mmol, 0.0453 g), and chlorosilane **2b** (1.00 equiv, 0.25 mmol, 52.0 µL). The product was purified using column chromatography (9:1 hexanes/EtOAc) and isolated as a clear oil (74.5 mg, 82%) Enantiomeric ratios were determined by HPLC with Diacel CHIRALPAK ® AD-H column (2% IPA/hexanes) 1.0 mL/min, **3fb1 =** 9.8 min, **3fb2** = 10.7 min, 87:13 er. ^{1}H NMR (400 MHz,C_6D_6) δ 7.71 – 7.63 (m, 6H), 7.19 (m, 9H), 1.99 (dd, *J* = 11.0, 4.3 Hz, 1H), 1.87 (m, 1H), 0.91 (dd, *J* = 6.6, 1.5 Hz, 7H), 0.84 (d, *J* = 6.9 Hz, 2H), 0.65 (s, 3H).^{13}C NMR (100 MHz, C_6D_6) δ 140.0, 137.9, 134.4, 133.7, 130.3, 130.0, 26.8, 26.5, 24.6, 1.7, 1.2, 1.2. ^{29}Si NMR (80 MHz,C_6D_6) δ –0.97, –25.26. HRMS (ESI) m/z: calc for $C_{23}H_{27}O_2Si_2^-$ [M - H]$^-$ 391.1555 found 391.1541 UV/Vis (hexanes/IPA 98:2) λ_{max} (*ε)* 208, 259 nm.

Racemic standard for **3fb**

<Chromatogram>

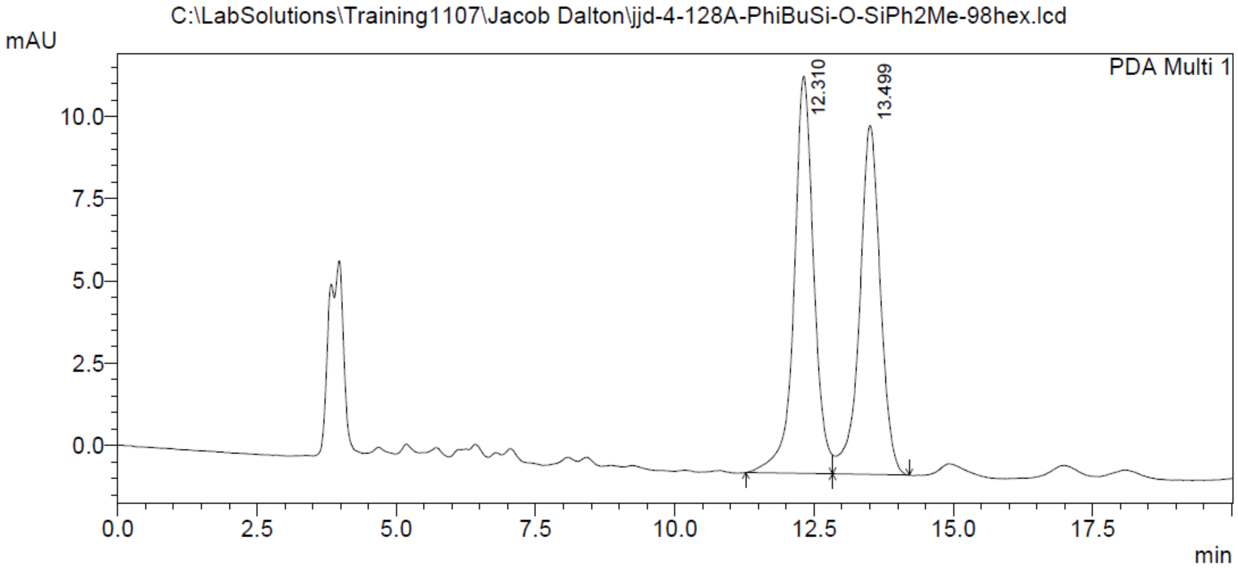

1 PDA Multi 1/259nm 4nm

< Peak Table >

PeakTable C:\LabSolutions\Training1107\Jacob Dalton\jjd-4-128A-PhiBuSi-O-SiPh2Me-98hex.lcd

PDA Ch1 259nm 4nm

Peak#	Ret. Time	Area	Height	Area %	Height %
1	12.310	296527	12076	51.740	53.212
2	13.499	276579	10618	48.260	46.788
Total		573106	22694	100.000	100.000

Enantiomerically enriched **3fb** (83:17 er)

<Chromatogram>

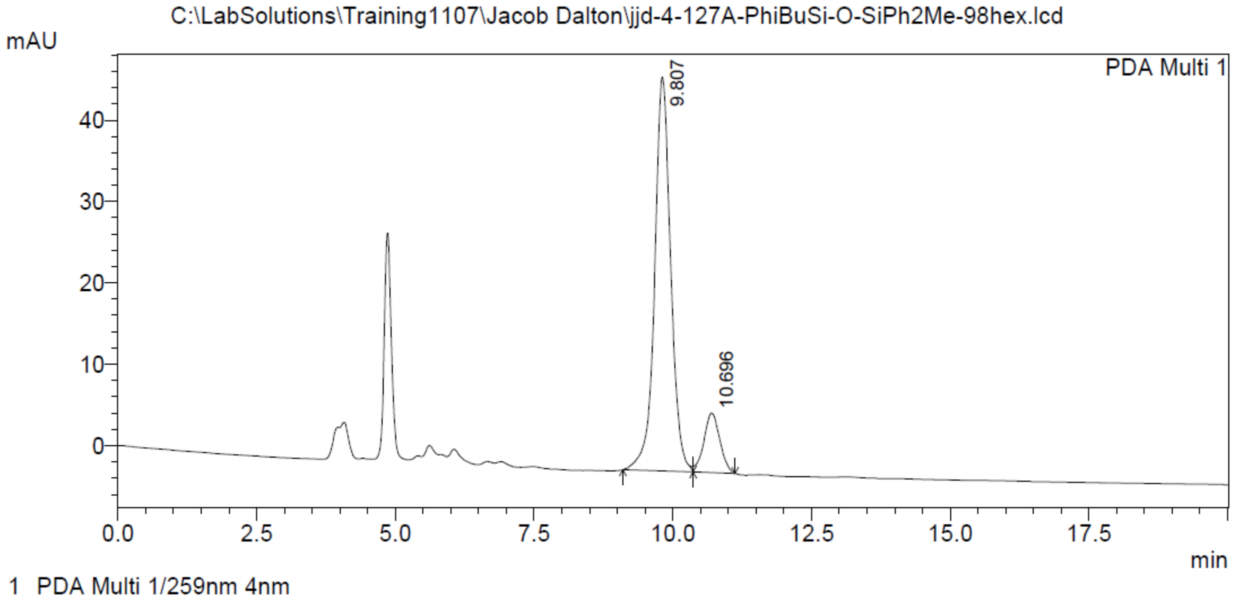

1 PDA Multi 1/259nm 4nm

< Peak Table >

PeakTable C:\LabSolutions\Training1107\Jacob Dalton\jjd-4-127A-PhiBuSi-O-SiPh2Me-98hex.lcd

PDA Ch1 259nm 4nm

Peak#	Ret. Time	Area	Height	Area %	Height %
1	9.807	976572	48416	86.955	86.843
2	10.696	146510	7335	13.045	13.157
Total		1123082	55751	100.000	100.000

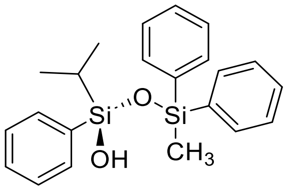

(*S*)-1-isopropyl-3-methyl-1,3,3-triphenyldisiloxan-1-ol (**3gb**). Synthesized according to the general procedure for desymmetrization of silanediols using silanediol **1g** (1.00 equiv, 0.15 mmol, 0.0273 g), and chlorosilane **2b** (1.00 equiv, 0.15 mmol, 31.5 µL). The product was purified using column chromatography (9:1 hexanes/EtOAc) and isolated as a clear oil (44.4 mg, 78%) Enantiomeric ratios were determined by HPLC with Diacel CHIRALPAK ® AD-H column (2% IPA/hexanes) 1.0 mL/min, **3gb1 =** 10.6 min, **3gb2** = 12.4 min 65:35 er. ^{1}H NMR (400 MHz, $CDCl_3$) δ 7.64 – 7.55 (m, 5H), 7.46 – 7.30 (m, 10H), 2.28 (s, 1H), 1.11-1.03 (m, 1H), 1.00 (d, *J* = 9.2 Hz, 6H), 0.98 (d, *J* = 9.4 Hz, 3H), 0.68 (s, 3H). ^{13}C NMR (100 MHz, $CDCl_3$) δ 137.64, 137.58, 135.02, 134.28, 134.10, 130.05, 129.85, 129.84, 127.98, 127.97, 127.85, 16.89, 16.82, 14.28. ^{29}Si NMR (80 MHz, $CDCl_3$) δ –10.39, –23.80. HRMS (ESI) m/z: calc for $C_{21}H_{22}O_2Si_2^-$ [M – CH_3]$^-$ 363.1164 found 363.1086. UV/Vis (hexanes/IPA 98:2) λ_{max} (*ε)* 205, 259, 656, 485, 462 nm.

Racemic standard for **3gb**

<Chromatogram>

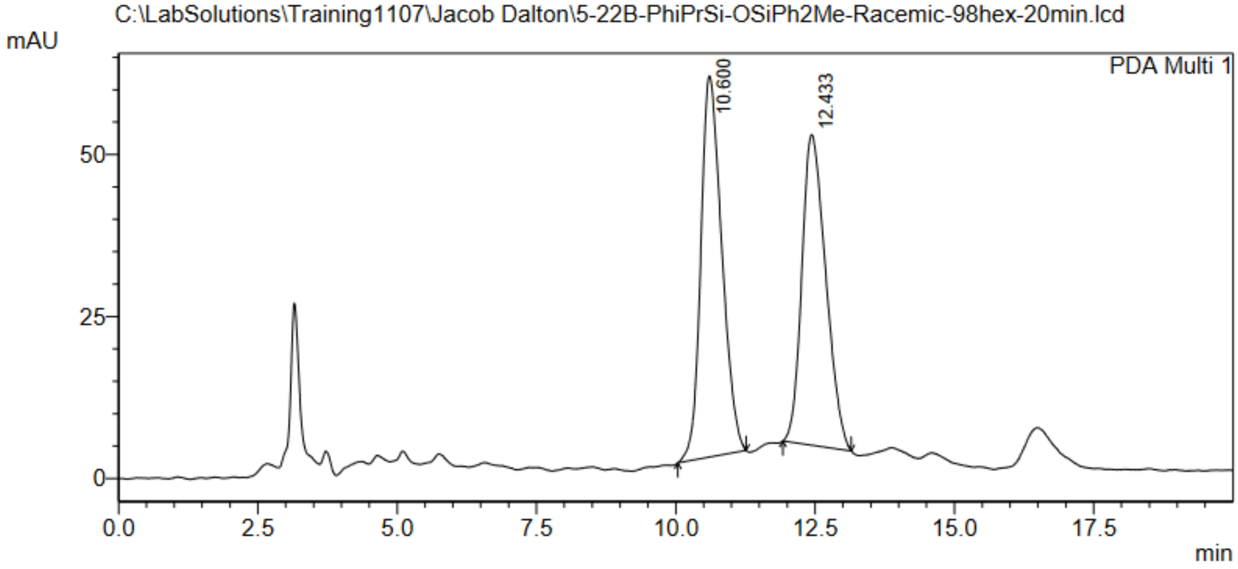

1 PDA Multi 1/204nm 4nm

< Peak Table >

PeakTable C:\LabSolutions\Training1107\Jacob Dalton\5-22B-PhiPrSi-OSiPh2Me-Racemic-98hex-20min.lcd

PDA Ch1 204nm 4nm

Peak#	Ret. Time	Area	Height	Area %	Height %
1	10.600	1541020	58830	51.880	55.113
2	12.433	1429331	47913	48.120	44.887
Total		2970351	106743	100.000	100.000

Enantiomerically enriched **3gb** (65:35 er)

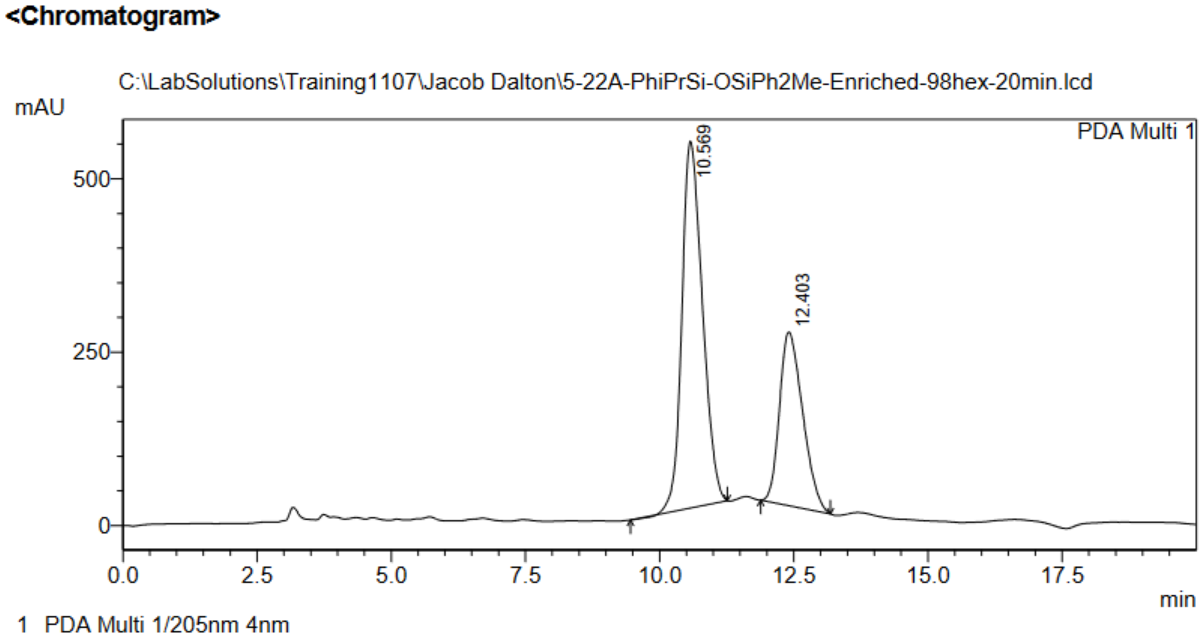

< Peak Table >

PeakTable C:\LabSolutions\Training1107\Jacob Dalton\5-22A-PhiPrSi-OSiPh2Me-Enriched-98hex-20min.lcd

PDA Ch1 205nm 4nm

Peak#	Ret. Time	Area	Height	Area %	Height %
1	10.569	14102926	529220	65.187	67.885
2	12.403	7531477	250368	34.813	32.115
Total		21634403	779587	100.000	100.000

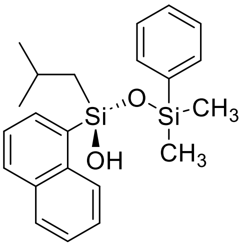

1-isobutyl-3,3-dimethyl-1-(naphthalen-1-yl)-3-phenyldisiloxan-1-ol (**3bc**). Synthesized according to the general procedure for desymmetrization of silanediols scaled up by 2.5 using silanediol **1b** (1.00 equiv, 0.25 mmol, 0.0490 g), and chlorosilane **2c** (1.00 equiv, 0.25 mmol, 33.6 µL). The product was purified using column chromatography (90:10 hexanes/EtOAc) and isolated as a clear oil (44.2 mg, 58%). Enantiomeric ratios were determined by HPLC with Diacel CHIRALPAK ® AD-H column (5% IPA/hexanes) 1 mL/min, **3bc1 =** 8.1min, **3bc2** = 8.6 min 93:7 er. ^{1}H NMR (600 MHz,C_6D_6) δ 8.46 (d, J = 8.4 Hz, 1H), 7.96 (d, 1H), 7.64 (t, 2H), 7.62 – 7.58 (m, 4H), 7.30 (t, 1H), 7.26 (t, J = 7.5 Hz, 1H), 7.23 (t, 1H), 7.12 (s, 1H), 1.85 (m, 1H), 0.97 (d, J = 1.7 Hz, 2H), 0.86 (dd, J = 12.7, 1.6 Hz, 7H), 0.36 (s, 4H), 0.34 (s, 3H). ^{13}C NMR (100 MHz,C_6D_6) δ 139.6, 137.4, 135.7, 134.6, 134.0, 133.5, 130.9, 129.8, 129.3, 129.0, 128.2, 127.9, 126.2, 125.8, 125.4, 27.9, 26.2, 24.5. ^{29}Si NMR (80 MHz,C_6D_6) δ -21.31, -24.55. UV/Vis (hexanes/IPA 95:5) λ_{max} (ε) 208, 281 nm.

Racemic standard for **3bc**

<Chromatogram>

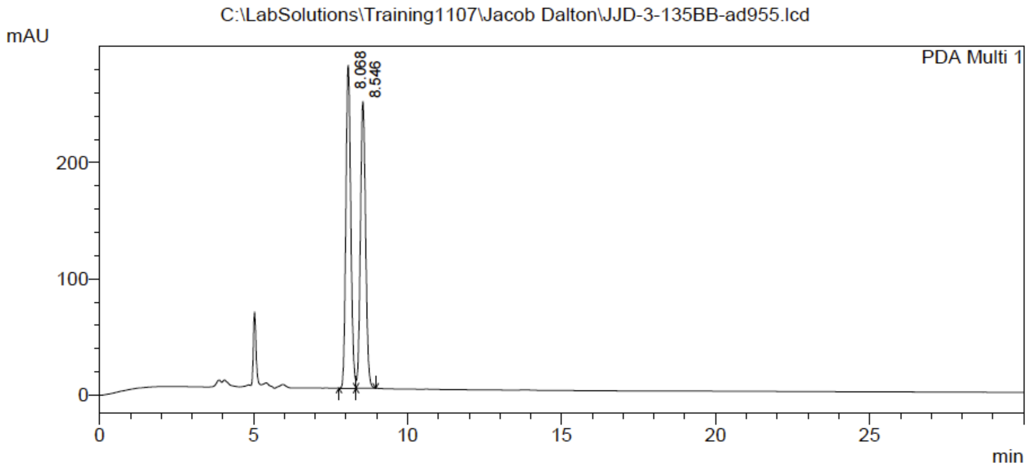

1 PDA Multi 1/254nm 4nm

< Peak Table >

PeakTable C:\LabSolutions\Training1107\Jacob Dalton\JJD-3-135BB-ad955.lcd

PDA Ch1 254nm 4nm

Peak#	Ret. Time	Area	Height	Area %	Height %
1	8.068	3091678	277781	51.767	52.974
2	8.546	2880579	246595	48.233	47.026
Total		5972258	524377	100.000	100.000

Enantiomerically enriched **3bc** (94:6 er)

<Chromatogram>

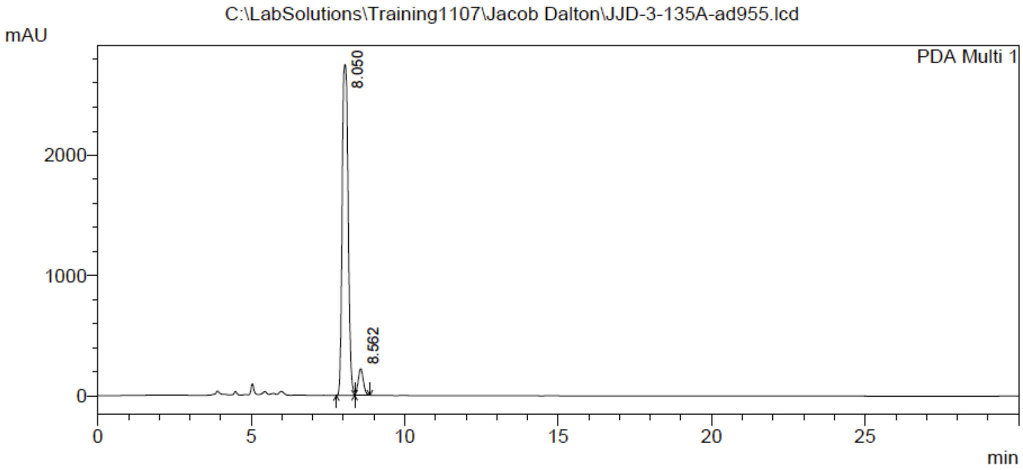

1 PDA Multi 1/263nm 4nm

< Peak Table >

PeakTable C:\LabSolutions\Training1107\Jacob Dalton\JJD-3-135A-ad955.lcd

PDA Ch1 263nm 4nm

Peak#	Ret. Time	Area	Height	Area %	Height %
1	8.050	37085326	2749790	93.415	92.560
2	8.562	2614222	221026	6.585	7.440
Total		39699548	2970817	100.000	100.000

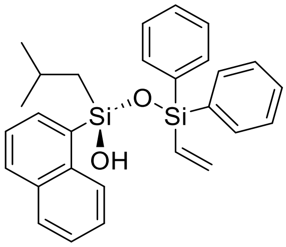

(S)-1-isobutyl-1-(naphthalen-1-yl)-3,3-diphenyl-3-vinyldisiloxan-1-ol (**3bd**). Synthesized according to the general procedure for desymmetrization of silanediols using silanediol **1b** (1.00 equiv, 0.20 mmol, 0.0492 g), and chlorosilane **2d** (1.00 equiv, 0.20 mmol, 44.2 μL). The product was purified using column chromatography (9:1 hexanes/EtOAc) and isolated as a clear oil (63.0 mg, 69%). Enantiomeric ratios were determined by HPLC with Diacel CHIRALPAK ® AS-H column (1% IPA/hexanes) 1 mL/min, **3bd1 =** 6.0 min, **3bd2** 6.3 min 97:3 er. ^{1}H NMR (400 MHz,C_6D_6) δ 8.54 – 8.48 (m, 1H), 8.05 (dd, J = 6.8, 1.3 Hz, 1H), 7.80 – 7.71 (m, 4H), 7.70 – 7.60 (m, 2H), 7.33 – 7.22 (m, 3H), 7.18 (dd, J = 5.2, 2.6 Hz, 6H), 6.51 (dd, J = 20.3, 14.9 Hz, 1H), 6.06 (dd, J = 14.9, 3.7 Hz, 1H), 5.93 (dd, J = 20.3, 3.7 Hz, 1H), 2.17 (s, 1H), 1.89 (m, 1H), 1.06 (d, J = 6.9 Hz, 2H), 0.86 (dd, J = 10.8, 6.6 Hz, 7H). ^{13}C NMR (100 MHz,C_6D_6) δ 137.3, 136.4, 135.99, 135.97, 135.7, 135.36, 135.27, 134.8, 134.0, 131.0, 130.26, 130.24, 129.2, 129.1 128.20, 128.18, 128.0, 126.3, 125.8, 125.4, 28.0, 26.24, 26.19, 24.4. ^{29}Si NMR (80 MHz,C_6D_6) δ –23.05. UV/Vis (hexanes/IPA 99:1) λ_{max} (ε) 203, 283 nm.

Racemic standard for **3bd**

<Chromatogram>

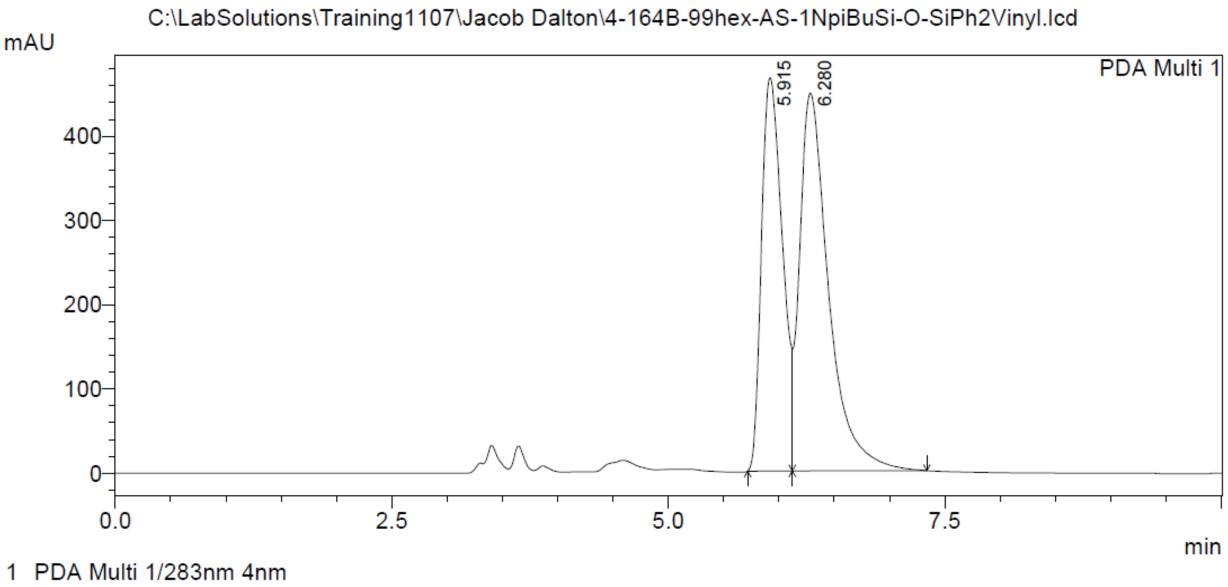

< Peak Table >

PeakTable C:\LabSolutions\Training1107\Jacob Dalton\4-164B-99hex-AS-1NpiBuSi-O-SiPh2Vinyl.lcd
PDA Ch1 283nm 4nm

Peak#	Ret. Time	Area	Height	Area %	Height %
1	5.915	6158464	467089	43.025	51.009
2	6.280	8155177	448608	56.975	48.991
Total		14313641	915697	100.000	100.000

Enantiomerically enriched **3bd** (97:3 er),

<Chromatogram>

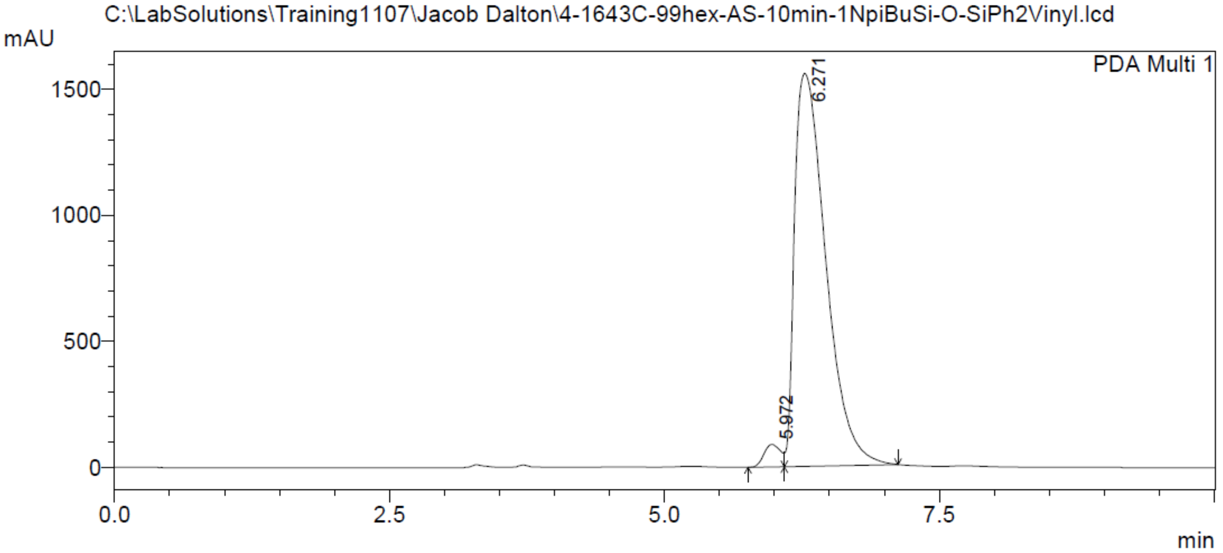

1 PDA Multi 1/282nm 4nm

< Peak Table >

PeakTable C:\LabSolutions\Training1107\Jacob Dalton\4-1643C-99hex-AS-10min-1NpiBuSi-O-SiPh2Vinyl.lcd
PDA Ch1 282nm 4nm

Peak#	Ret. Time	Area	Height	Area %	Height %
1	5.972	954816	89321	3.013	5.422
2	6.271	30738480	1558039	96.987	94.578
Total		31693297	1647360	100.000	100.000

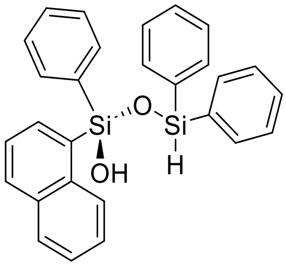

(*S*)-1-(naphthalen-1-yl)-1,3,3-triphenyldisiloxan-1-ol (**3aa**). Synthesized according to the general procedure for desymmetrization of silanediols using silanediol **1b** (1.00 equiv, 0.15 mmol, 0.0246 g), and chlorosilane **2a** (1.00 equiv, 0.15 mmol, 20.8 μL). The product was purified using column chromatography (9:1 hexanes/EtOAc) and isolated as a clear oil (24.2 mg, 36%). Enantiomeric ratios were determined by HPLC with Diacel CHIRALPAK ® AD-H column (2% IPA/hexanes) 1.0 mL/min, **3aa =** 28.1 min, **3aa** = 32.1 min, 70:30 er. Product matched previously reported NMR.[2] ^{1}H NMR (400 MHz, $CDCl_3$) δ 8.20 (dd, J = 8.44, 1.0 Hz, 1H), 7.94 – 7.89 (m, 2H), 7.84 (d, J = 0.9 Hz, 1H), 7.67 – 7.62 (m, 3H), 7.57 (ddd, J = 8.0, 2.8, 1.5 Hz, 4H), 7.47 – 7.36 (m, 6H), 7.34 – 7.29 (m, 7H), 5.64 (s, 1H), 2.90 (s, 1H).

Racemic **3aa**

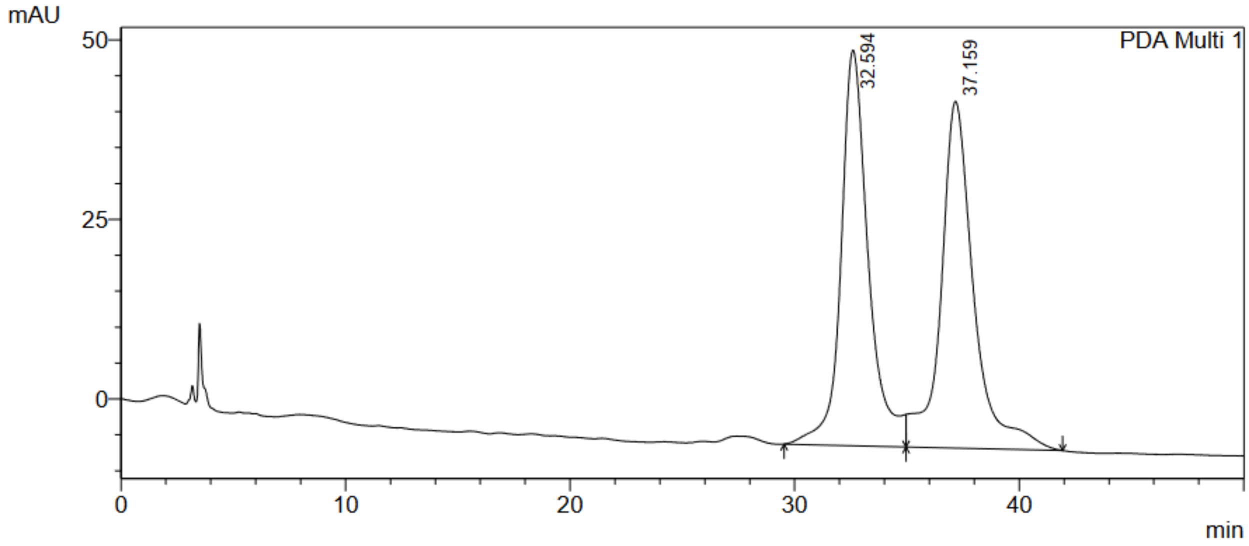

1 PDA Multi 1/283nm 4nm

< Peak Table >

PeakTable C:\LabSolutions\Training1107\Jacob Dalton\5-41A-1NpPhSi-OSiPh2H-98hex-50min.lcd

PDA Ch1 283nm 4nm

Peak#	Ret. Time	Area	Height	Area %	Height %
1	32.594	4680246	55084	48.820	53.284
2	37.159	4906563	48294	51.180	46.716
Total		9586810	103378	100.000	100.000

Enantiomerically enriched **3aa** (70:30)

C:\LabSolutions\Training1107\Jacob Dalton\5-12-1NpPhSi-O-SiPh2H-98hex-40min.lcd

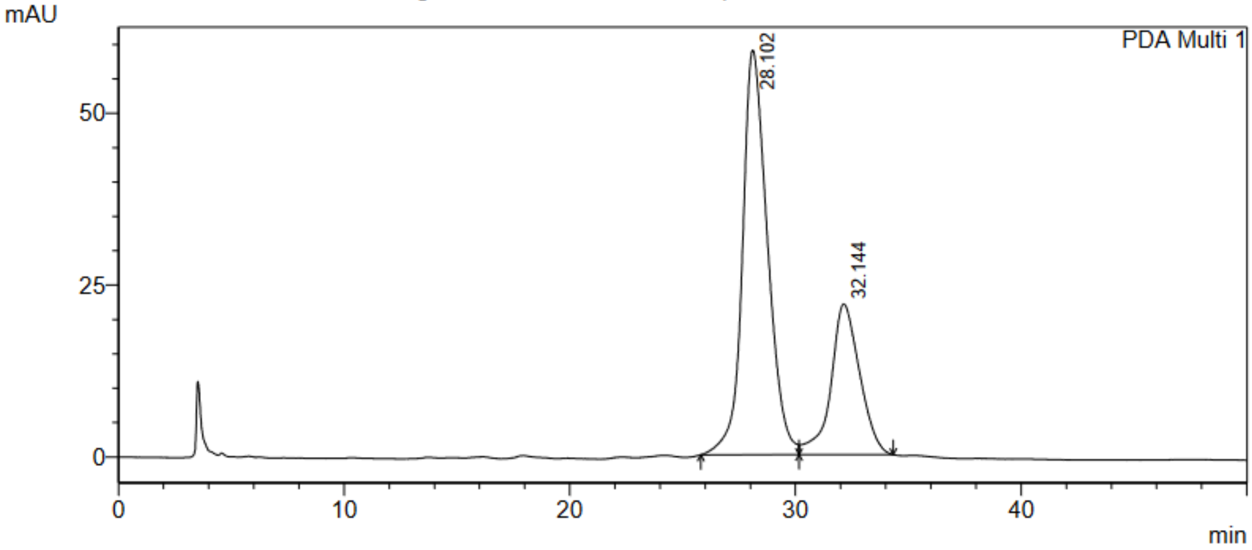

1 PDA Multi 1/282nm 4nm

< Peak Table >

PeakTable C:\LabSolutions\Training1107\Jacob Dalton\5-12-1NpPhSi-O-SiPh2H-98hex-40min.lcd

PDA Ch1 282nm 4nm

Peak#	Ret. Time	Area	Height	Area %	Height %
1	28.102	4543771	58871	69.942	72.851
2	32.144	1952693	21939	30.058	27.149
Total		6496464	80810	100.000	100.000

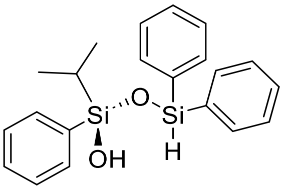

(*S*)-1-isopropyl-1,3,3-triphenyldisiloxan-1-ol (**3ga**). Synthesized according to the general procedure for desymmetrization of silanediols using silanediol **1g** (1.00 equiv, 0.15 mmol, 0.0246 g), and chlorosilane **2a** (1.00 equiv, 0.15 mmol, 20.8 µL). The product was purified using column chromatography (9:1 hexanes/EtOAc) and isolated as a clear oil (26.8 mg, 49%). Enantiomeric ratios were determined by HPLC with Diacel CHIRALPAK ® AD-H column (2% IPA/hexanes) 1.0 mL/min, **3ga1 =** 14.4 min, **3ga2** = 18.2 min 98:2 er. Product matched previously reported NMR.[2] ^{1}H NMR (400 MHz, $CDCl_3$) δ 7.62 (dtd, *J* = 8.0, 6.5, 1.5 Hz, 6H), 7.45 – 7.32 (m, 9H), 5.63 (s, 1H), 2.34 (s, 1H), 1.05 – 0.97 (m, 7H).

Scalemic 3ga

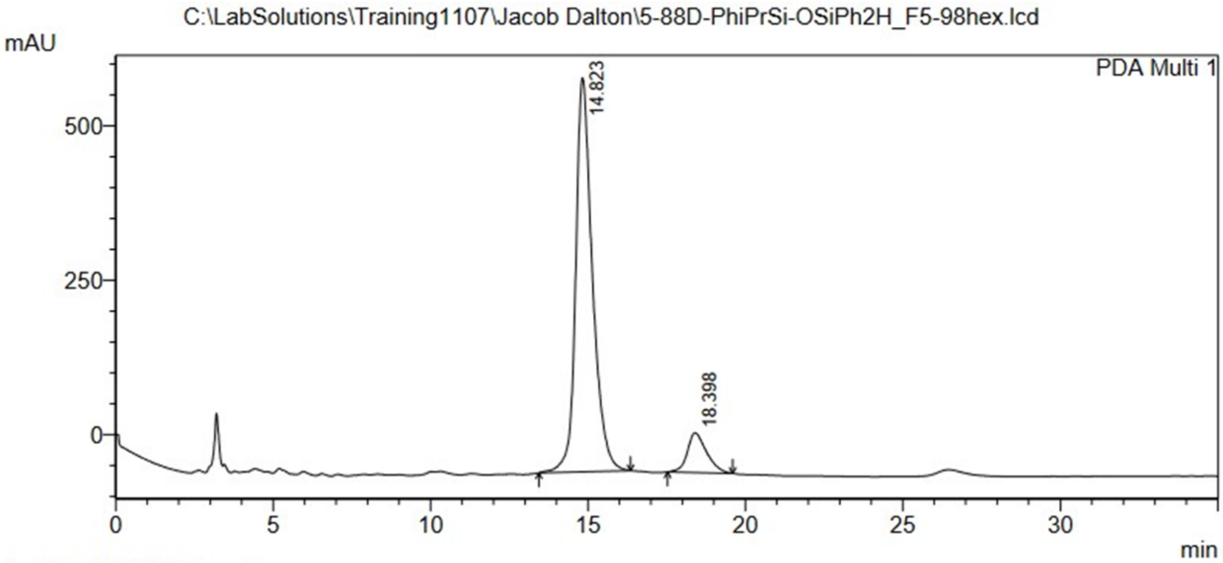

1 PDA Multi 1/205nm 4nm

< Peak Table >

PeakTable C:\LabSolutions\Training1107\Jacob Dalton\5-88D-PhiPrSi-OSiPh2H_F5-98hex.lcd
PDA Ch1 205nm 4nm

Peak#	Ret. Time	Area	Height	Area %	Height %
1	14.823	23514952	637753	89.584	90.839
2	18.398	2734086	64316	10.416	9.161
Total		26249038	702069	100.000	100.000

Enantiomerically enriched **3ga** (98:2 er)

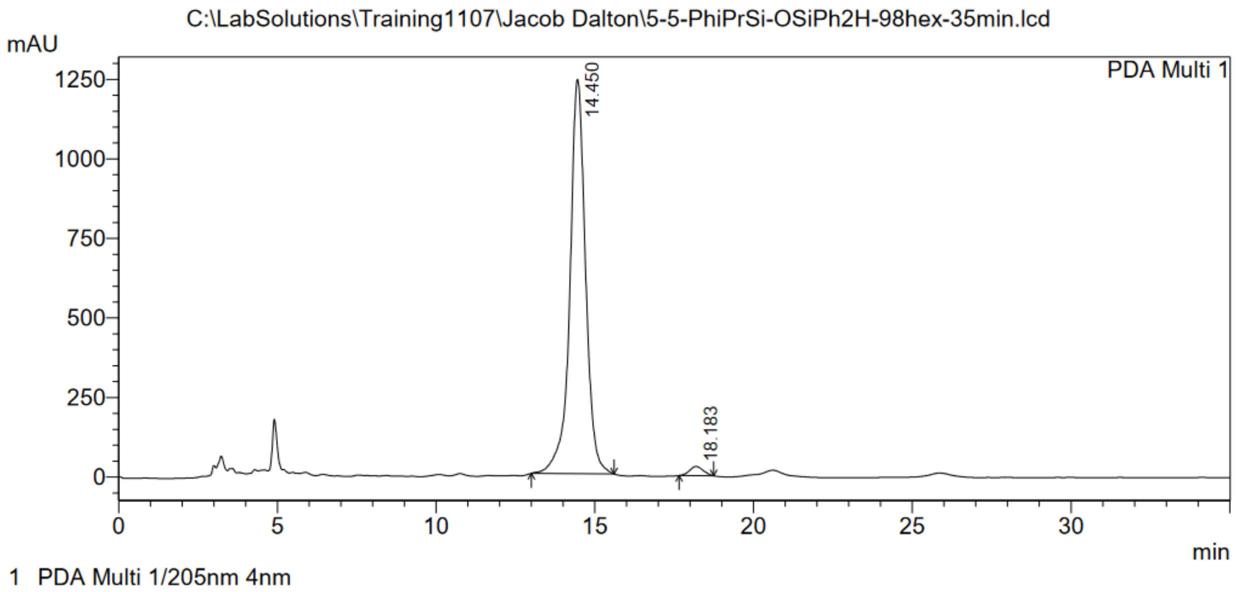

< Peak Table >

PeakTable C:\LabSolutions\Training1107\Jacob Dalton\5-5-PhiPrSi-OSiPh2H-98hex-35min.lcd
PDA Ch1 205nm 4nm

Peak#	Ret. Time	Area	Height	Area %	Height %
1	14.450	42639071	1239594	97.991	97.704
2	18.183	874043	29134	2.009	2.296
Total		43513114	1268729	100.000	100.000

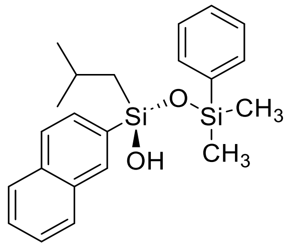

(*S*)-1-isobutyl-3,3-dimethyl-1-(naphthalen-2-yl)-3-phenyldisiloxan-1-ol (**3dc**). Synthesized according to the general procedure for desymmetrization of silanediols scaled up by 2.5 using silanediol **1d** (1.00 equiv, 0.25 mmol, 0.0490 g), and chlorosilane **2c** (1.00 equiv, 0.25 mmol, 33.6 μL). The product was purified using column chromatography (90:10 hexanes/EtOAc) and isolated as a clear oil (66.4 mg, 70%). Enantiomeric ratios were determined by HPLC with Diacel CHIRALPAK ® AD-H column (2% IPA/hexanes) 0.5 mL/min, **3dc1 =** 44.0 min, **3dc2** = 47.0 min, 62:38 er. ^{1}H NMR (400 MHz,C_6D_6) δ 8.22 (s, 1H), 7.74 (dd, *J* = 8.2, 1.1 Hz, 1H), 7.71 – 7.61 (m, 5H), 7.29 – 7.25 (m, 2H), 7.22 (dd, *J* = 5.4, 1.8 Hz, 3H), 2.00 (d, *J* = 7.6 Hz, 1H), 1.93 (m, 1H), 0.99 (s, 3H), 0.97 (s, 3H), 0.91 (d, *J* = 7.0 Hz, 2H), 0.42 (d, *J* = 3.5 Hz, 6H). ^{13}C NMR (150 MHz,C_6D_6) δ 139.4, 134.9, 134.8, 134.4, 133.2, 133.1, 129.8, 129.5, 128.4, 127.8, 127.6, 127.1, 126.5, 125.9, 26.3, 26.0, 24.1, 0.6, 0.6. ^{29}Si NMR (80 MHz,C_6D_6) *δ* -0.50, -0.80. UV/Vis (hexanes/IPA 98:2) λ_{max} (*ε)* 227, 199, 269 nm.

Racemic standard for **3dc**

<Chromatogram>

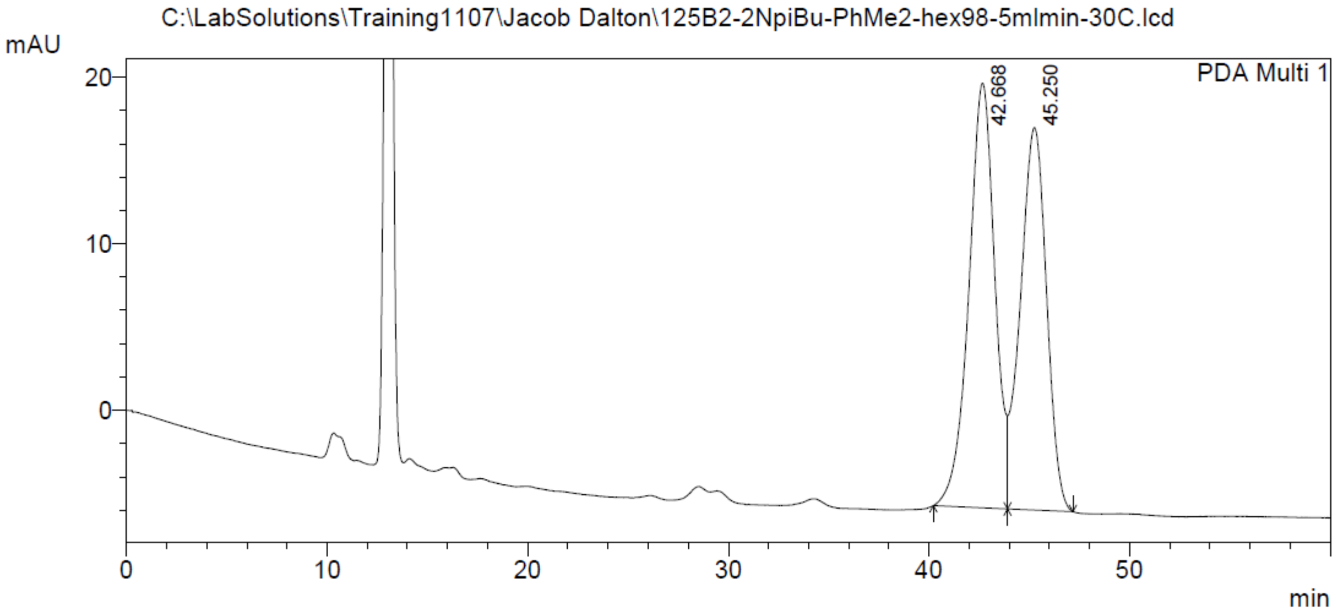

1 PDA Multi 1/254nm 4nm

< Peak Table >

PeakTable C:\LabSolutions\Training1107\Jacob Dalton\125B2-2NpiBu-PhMe2-hex98-5mlmin-30C.lcd
PDA Ch1 254nm 4nm

Peak#	Ret. Time	Area	Height	Area %	Height %
1	42.668	2297643	25509	52.391	52.622
2	45.250	2087926	22966	47.609	47.378
Total		4385569	48475	100.000	100.000

Enantiomerically enriched **3dc** (62:38 er)

<Chromatogram>

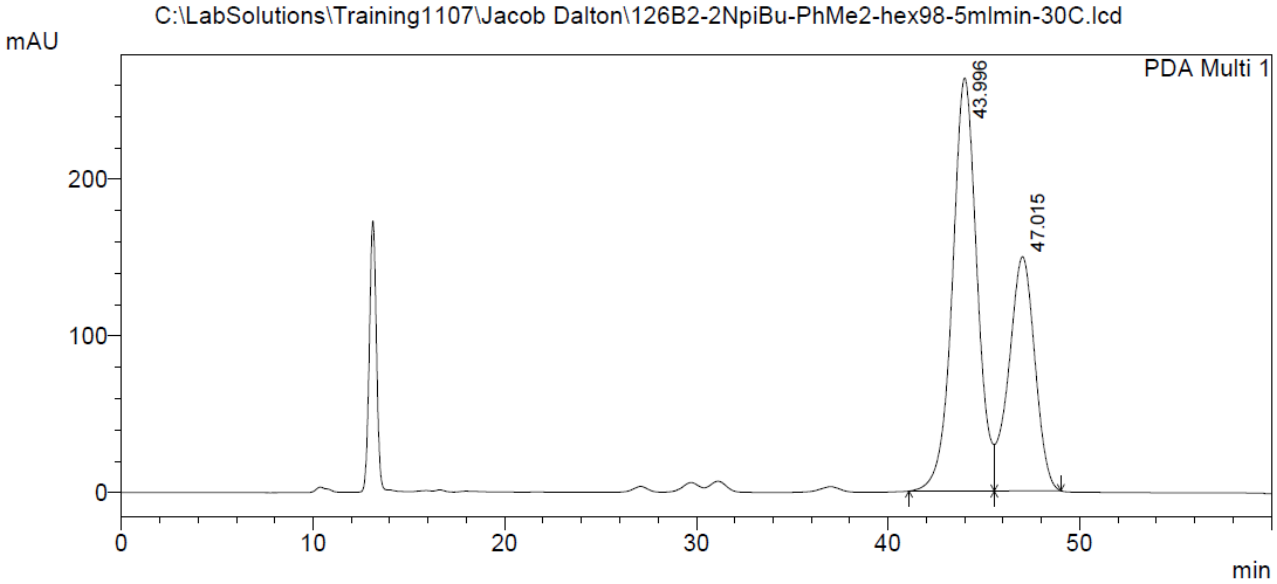

1 PDA Multi 1/254nm 4nm

< Peak Table >

PeakTable C:\LabSolutions\Training1107\Jacob Dalton\126B2-2NpiBu-PhMe2-hex98-5mlmin-30C.lcd

PDA Ch1 254nm 4nm

Peak#	Ret. Time	Area	Height	Area %	Height %
1	43.996	23443315	263509	62.405	63.811
2	47.015	14122982	149446	37.595	36.189
Total		37566296	412954	100.000	100.000

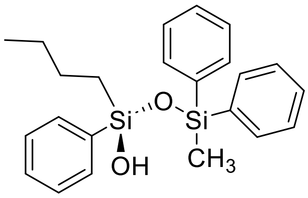

(*S*)-1-butyl-3-methyl-1,3,3-triphenyldisiloxan-1-ol (**3hb**). Synthesized according to the general procedure for desymmetrization of silanediols using silanediol **1h** (1.00 equiv, 0.15 mmol, 0.0294 g), and chlorosilane **2b** (1.00 equiv, 0.15 mmol, 31.5 μL). The product was purified using column chromatography (9:1 hexanes/EtOAc) and isolated as a clear oil (46.5 mg, 79%). Enantiomeric ratios were determined by HPLC with Diacel CHIRALPAK ® AD-H column (2% IPA/hexanes) 1.0 mL/min, **3hb1 =** 11.6 min, **3hb2** = 12.6 min, 82:18 er. ^{1}H NMR (400 MHz, C_6D_6) δ 7.69 (dtd, J = 5.5, 3.8, 2.5 Hz, 6H), 7.22 – 7.17 (m, 9H), 1.95 (s, 1H), 1.44 – 1.32 (m, 2H), 1.31 – 1.15 (m, 2H), 0.85 – 0.80 (m, 2H), 0.77 (t, J = 7.3 Hz, 3H), 0.66 (s, 3H).^{13}C NMR (100 MHz, C_6D_6) δ 138.0, 137.99, 137.0, 134.5, 134.2, 130.16, 130.0, 26.5, 25.4, 15.9, 13.9, 1.4, -0.3. ^{29}Si NMR (80 MHz, C_6D_6) δ -10.49, -21.81. HRMS (ESI) m/z: calc for $[M]^-$, found UV/Vis (hexanes/IPA 98:2) λ_{max} (*ε)* 206, 259, 656, 485, 291 nm.

Racemic standard for **3hb**

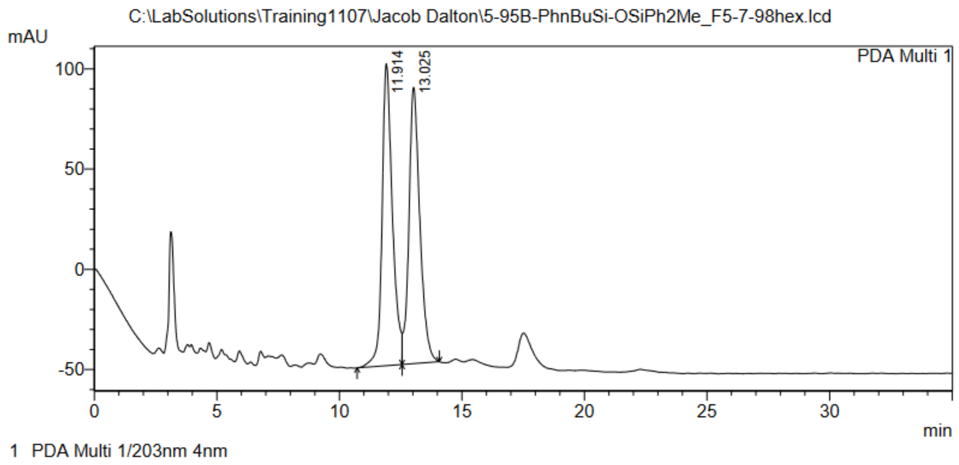

< Peak Table >

PeakTable C:\LabSolutions\Training1107\Jacob Dalton\5-95B-PhnBuSi-OSiPh2Me_F5-7-98hex.lcd
PDA Ch1 203nm 4nm

Peak#	Ret. Time	Area	Height	Area %	Height %
1	11.914	4421400	150612	50.717	52.216
2	13.025	4296358	137826	49.283	47.784
Total		8717758	288438	100.000	100.000

Enantiomerically enriched **3hb** (82:18)

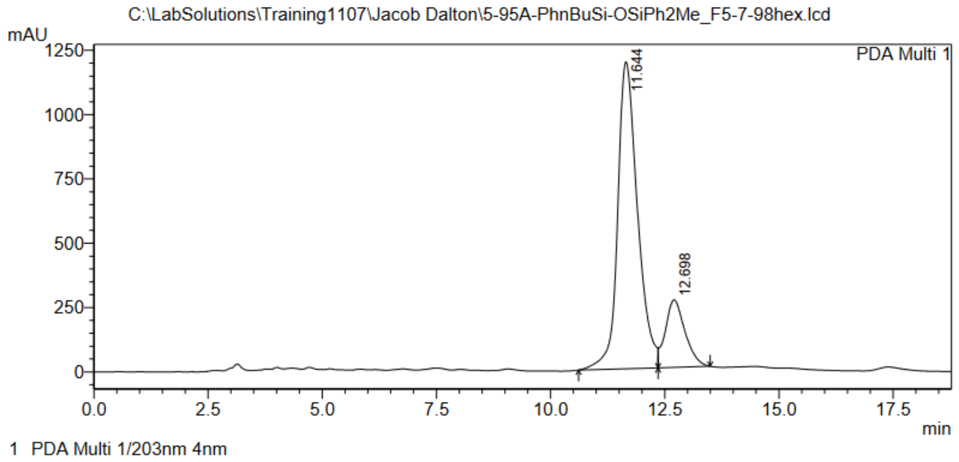

< Peak Table >

PeakTable C:\LabSolutions\Training1107\Jacob Dalton\5-95A-PhnBuSi-OSiPh2Me_F5-7-98hex.lcd
PDA Ch1 203nm 4nm

Peak#	Ret. Time	Area	Height	Area %	Height %
1	11.644	36061698	1192869	82.125	81.967
2	12.698	7849304	262431	17.875	18.033
Total		43911002	1455300	100.000	100.000

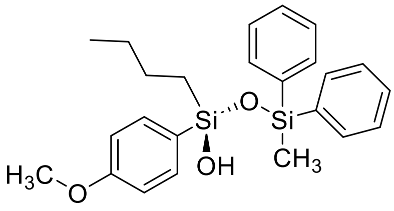

(*S*)-1-butyl-1-(4-methoxyphenyl)-3-methyl-3,3-diphenyldisiloxan-1-ol (3ib). Synthesized according to the general procedure for desymmetrization of silanediols using silanediol **1i** (1.00 equiv, 0.15 mmol, 0.0340 g), and chlorosilane **2b** (1.00 equiv, 0.15 mmol, 31.5 μL). The product was purified using column

chromatography (9:1 hexanes/EtOAc) and isolated as a clear oil (31.8 mg, 50%). Enantiomeric ratios were determined by HPLC with Diacel CHIRALPAK ® AD-H column (2% IPA/hexanes) 1.0 mL/min, **3ib1 =** 17.3 min, **3ib2** = 19.1 min, 77:23 er. ^{1}H NMR (400 MHz, C_6D_6) δ 7.72 (m 4H), 7.66 – 7.61 (m, 2H), 7.60 – 7.54 (m, 6H), 7.19 (m, 4H, obscured), 6.86 – 6.78 (m, 2H), 3.28 (s, 3H), 1.65 (s, 1H), 1.48 – 1.35 (m, 2H), 1.26 (q, J = 7.3 Hz, 2H), 0.89 – 0.77 (m, 5H), 0.69 (s, 3H).^{13}C NMR (100 MHz, C_6D_6) δ 161.7, 138.2, 138.1, 137.9, 135.8, 134.5, 134.4, 130.0, 129.96, 114.0, 54.5, 26.6, 25.6, 16.1, 14.0, 1.4, -0.2. δ ^{29}Si NMR (80 MHz, C_6D_6) δ -10.76, -21.80. HRMS (ESI) run in positive and negative mode, no particles detected. UV/Vis (hexanes/IPA 98:2) λ_{max} (*ε)* 205, 259, 485, 292, 421 nm.

Racemic standard for **3ib**

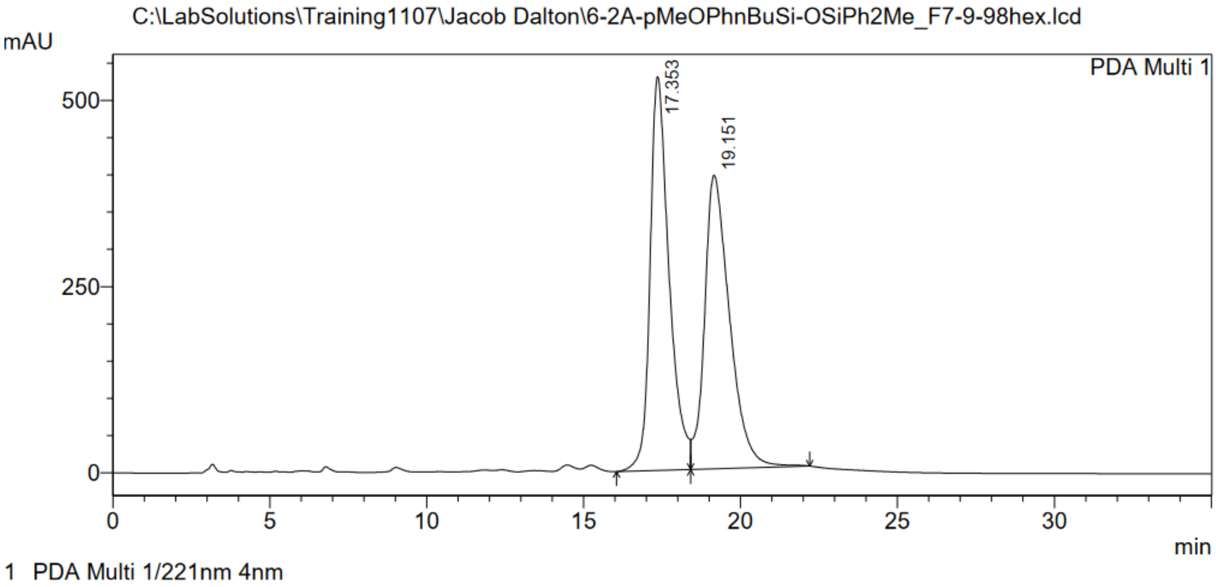

< Peak Table >

PeakTable C:\LabSolutions\Training1107\Jacob Dalton\6-2A-pMeOPhnBuSi-OSiPh2Me_F7-9-98hex.lcd

PDA Ch1 221nm 4nm

Peak#	Ret. Time	Area	Height	Area %	Height %
1	17.353	22270800	528695	50.388	57.277
2	19.151	21928219	394347	49.612	42.723
Total		44199019	923041	100.000	100.000

Enantiomerically enriched **3ib** (77:23)

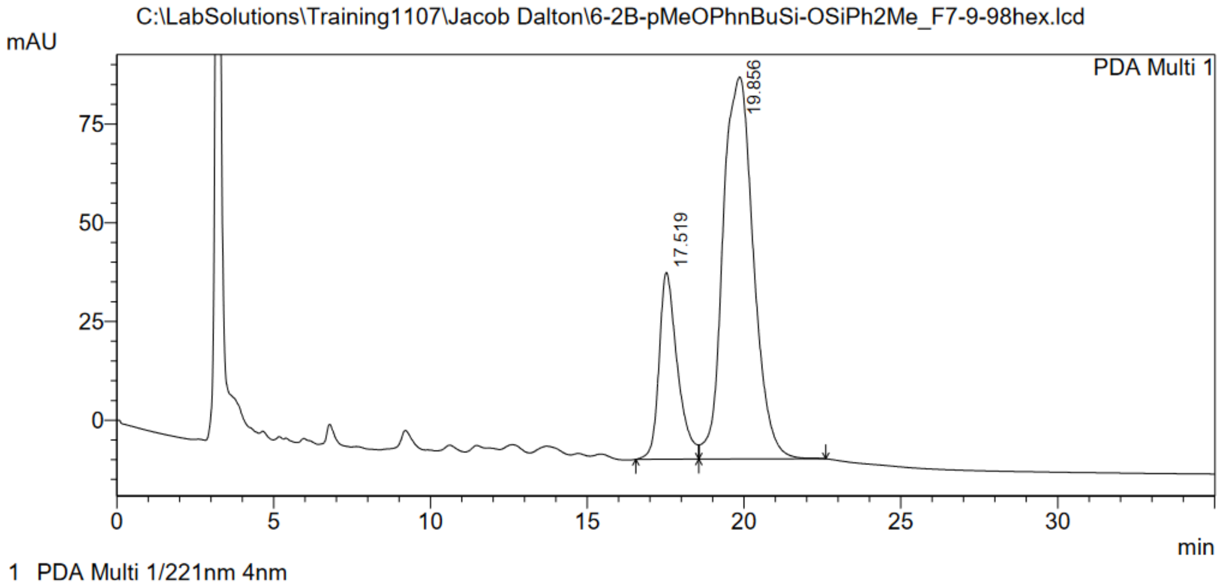

1 PDA Multi 1/221nm 4nm

< Peak Table >

PeakTable C:\LabSolutions\Training1107\Jacob Dalton\6-2B-pMeOPhnBuSi-OSiPh2Me_F7-9-98hex.lcd

PDA Ch1 221nm 4nm

Peak#	Ret. Time	Area	Height	Area %	Height %
1	17.519	1930509	47308	22.785	32.828
2	19.856	6542040	96798	77.215	67.172
Total		8472550	144106	100.000	100.000

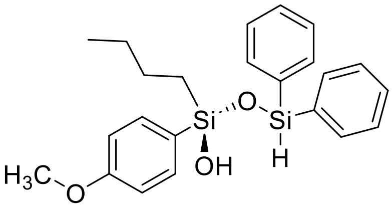

(*S*)-1-butyl-1-(4-methoxyphenyl)-3,3-diphenyldisiloxan-1-ol (**3ia**). Synthesized according to the general procedure for desymmetrization of silanediols using silanediol **1x** (1.00 equiv, 0.15 mmol, 0.0340 g), and chlorosilane **2a** (1.00 equiv, 0.15 mmol, 27.8 µL). The product was purified using column chromatography (9:1 hexanes/EtOAc) and isolated as a clear oil (16.1 mg, 26%). Enantiomeric ratios were determined by HPLC with Diacel CHIRALPAK ® AD-H column (2% IPA/hexanes) 1.0 mL/min, **3ia1 =** 22.7 min, **3ia2** = 24.4, 66:34 er. ^{1}H NMR (400 MHz, C_6D_6) δ δ 7.77 – 7.72 (m, 4H), 7.63 (d, J = 8.6 Hz, 2H), 7.18 (m,, 6H), 6.82 (d, J = 8.6 Hz, 2H), 5.95 (s, 1H), 3.27 (s, 3H), 1.49 – 1.33 (m, 3H), 1.31 – 1.20 (m, 2H), 1.11 – 1.05 (m, 3H), 0.89 – 0.83 (m, 2H), 0.79 (t, J = 7.3 Hz, 3H). ^{13}C NMR (100 MHz, C_6D_6) δ 161.8, 135.8, 135.7, 135.4, 134.8, 134.8, 130.6, 130.5, 127.2, 114.1, 54.5, 26.5, 25.5, 16.4, 13.8. HRMS (ESI) m/z: calc for $[M]^-$, found UV/Vis (hexanes/IPA 98:2) λ_{max} (*ε*) 203, 223, 486, 272, 428 nm.

Racemic standard for **3ia**

C:\LabSolutions\Training1107\Jacob Dalton\6-3B-pMeOPhnBuSi-OSiPh2H_F11-98hex.lcd

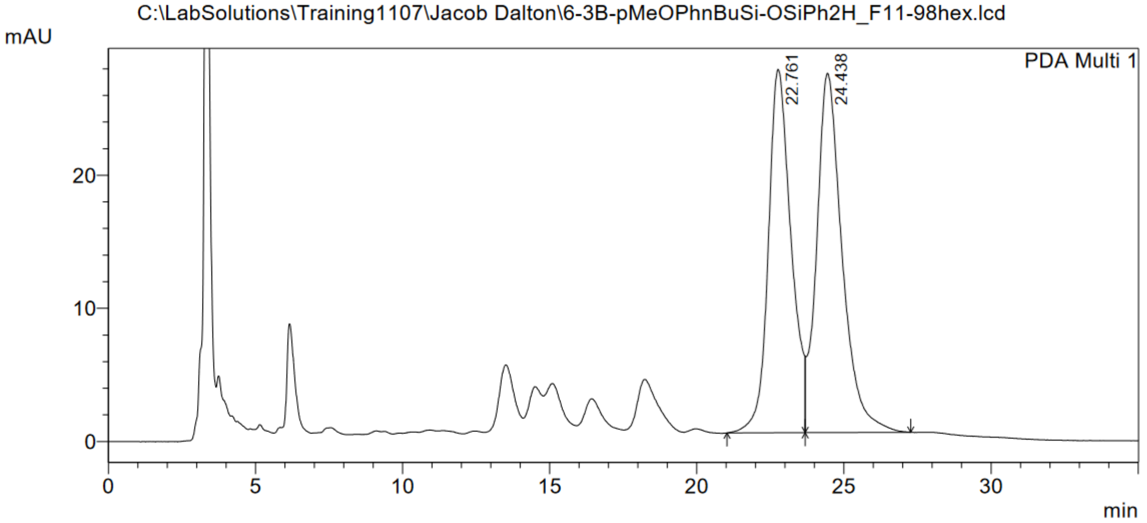

1 PDA Multi 1/224nm 4nm

< Peak Table >

PeakTable C:\LabSolutions\Training1107\Jacob Dalton\6-3B-pMeOPhnBuSi-OSiPh2H_F11-98hex.lcd

PDA Ch1 224nm 4nm

Peak#	Ret. Time	Area	Height	Area %	Height %
1	22.761	1470021	27317	47.354	50.283
2	24.438	1634320	27009	52.646	49.717
Total		3104341	54325	100.000	100.000

Enantiomerically enriched **3ia** (66:34)

C:\LabSolutions\Training1107\Jacob Dalton\6-3A-pMeOPhnBuSi-OSiPh2H_F11-98hex.lcd

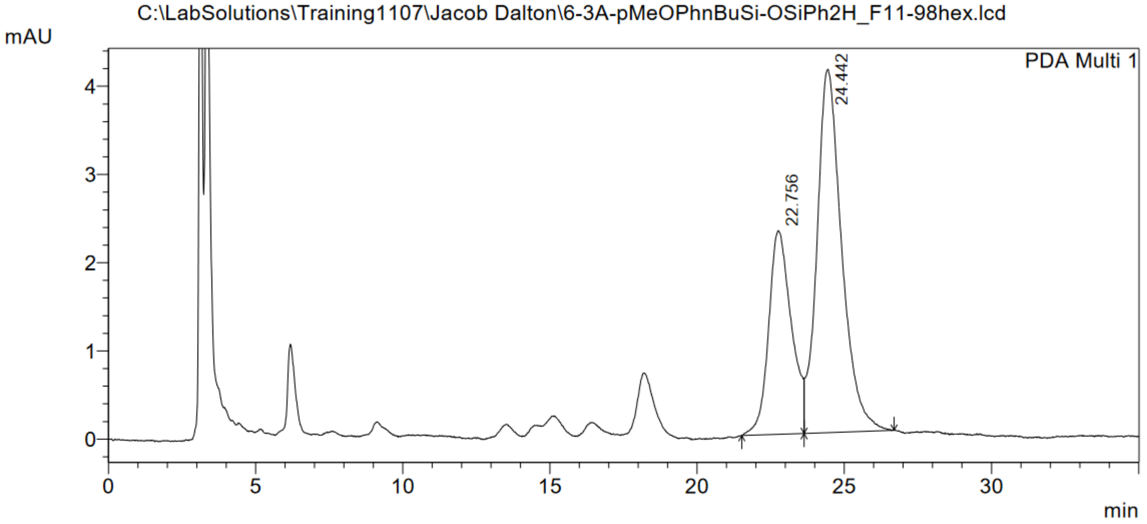

1 PDA Multi 1/223nm 4nm

< Peak Table >

PeakTable C:\LabSolutions\Training1107\Jacob Dalton\6-3A-pMeOPhnBuSi-OSiPh2H_F11-98hex.lcd

PDA Ch1 223nm 4nm

Peak#	Ret. Time	Area	Height	Area %	Height %
1	22.756	124398	2312	33.994	35.946
2	24.442	241541	4120	66.006	64.054
Total		365939	6432	100.000	100.000

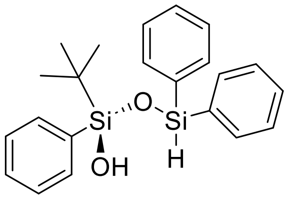

(*S*)-1-(tert-butyl)-1,3,3-triphenyldisiloxan-1-ol (**3ja**). Synthesized according to the general procedure for desymmetrization of silanediols using silanediol **1j** (1.00 equiv, 0.15 mmol, 0.0290 g), and chlorosilane **2a** (1.00 equiv, 0.25 mmol, 33.6 µL). The product was purified using column chromatography and matched previously reported spectra[2] (90:10 hexanes/EtOAc) and isolated as a clear oil (66.4 mg, 32%). Enantiomeric ratios were determined by HPLC with Diacel CHIRALPAK ® AD-H column (2% IPA/hexanes) 0.5 mL/min, **3ja1 =** 25.7 min, **3ja2** = 26.0 min, 51:49 er. ^{1}H NMR (400 MHz, $CDCl_3$) δ 7.67 – 7.57 (m, 5H), 7.47 – 7.30 (m, 10H), 5.66 (s, 1H), 2.32 (s, 1H), 0.95 (s, 9H).^{13}C NMR (100 MHz,$CDCl_3$) δ 135.5 (d, *J* = 2 Hz), 134.8 (d, *J* = 1 Hz), 134.4, 133.9, 130.4 (d, *J* = 3 Hz), 130.1, 128.2 (d, *J* = 1 Hz), 127.78, 31.74, 25.86, 22.81, 18.71, 14.27.

Racemic standard for **3ja**

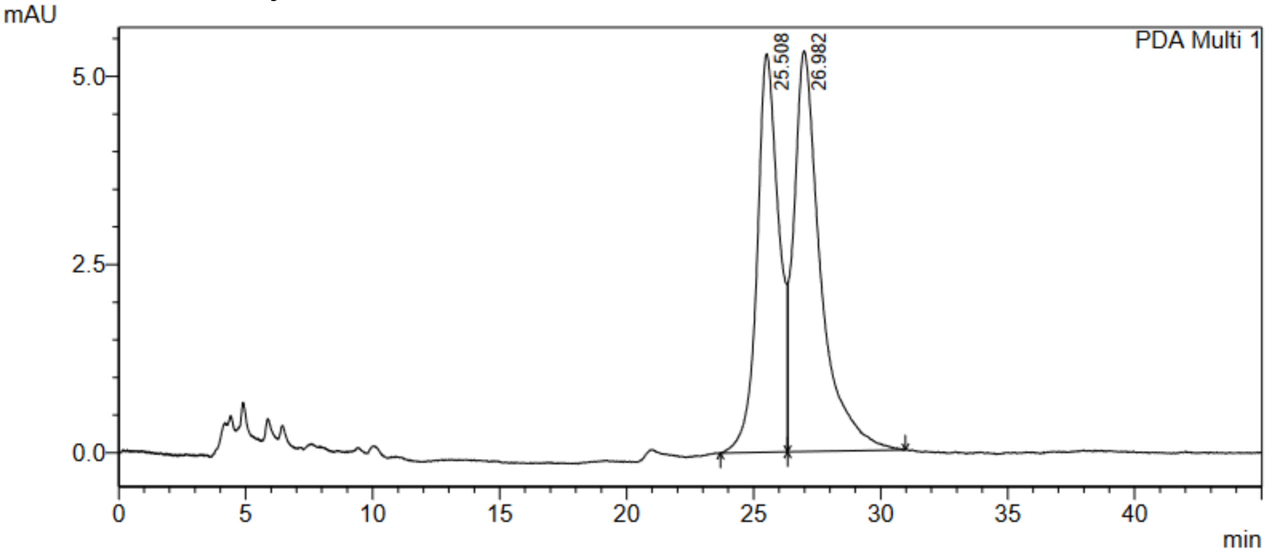

1 PDA Multi 1/254nm 4nm

< Peak Table >

PeakTable C:\LabSolutions\Training1107\Jacob Dalton\4-189B-PhtBuSi-O-SiPh2H-AD99Hex-08mlmin-45-racemic.lcd
PDA Ch1 254nm 4nm

Peak#	Ret. Time	Area	Height	Area %	Height %
1	25.508	315987	5294	44.628	49.839
2	26.982	392053	5329	55.372	50.161
Total		708040	10623	100.000	100.000

Enantiomerically enriched **3ja** (51:49 er)

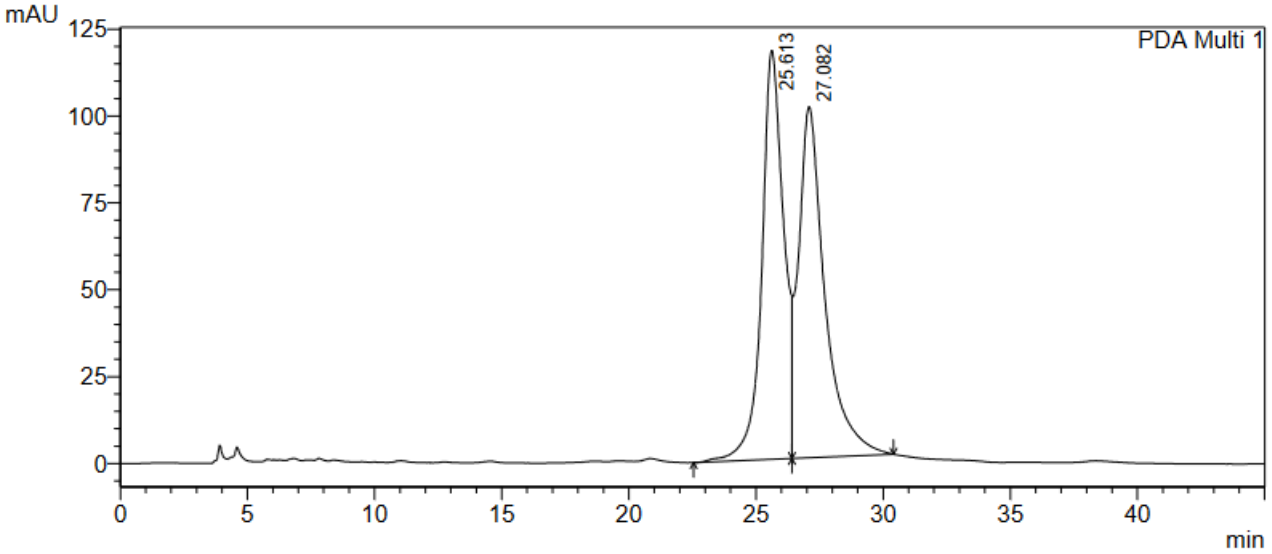

1 PDA Multi 1/220nm 4nm

< Peak Table >

PeakTable C:\LabSolutions\Training1107\Jacob Dalton\4-189A-PhtBuSi-O-SiPh2H-AD99Hex-08mlmin-45-enriched.lcd

PDA Ch1 220nm 4nm

Peak#	Ret. Time	Area	Height	Area %	Height %
1	25.613	7014579	117654	48.953	53.814
2	27.082	7314766	100976	51.047	46.186
Total		14329345	218629	100.000	100.000

11. X-Ray crystallographic structure and information

All crystallographic calculations were performed on a Surface Pro7 with Intel i7-1065G7 at 1.30 GHz with four cores, eight processors and 16 GB of extended memory. Data collected were corrected for Lorentz and polarization effects with Saint[12] and absorption using Blessing's method and merged as incorporated with the program Sadabs.[10,11] The SHELXTL[12] program package was implemented to determine the probable space group and set up the initial files. System symmetry, systematic absences and intensity statistics indicated the non-standard centrosymmetric monoclinic space group P21/n (no. 14). The structure was determined by direct methods with nearly all non-hydrogen atoms being located directly for the four unique, but extremely similar molecules using the program XT.[13] The structure was refined with XL.[14] The 157736 data collected were merged, based upon identical indices to 47049 data, and then merged for least squares refinement to 12121 unique data [R(int)=0.0178]. One of the molecules displayed disorder that was modeled and found to be 0.83:0.17 so its partial occupancy carbon atoms were refined anisotropically with similar U (SIMU/ISOR) instructions. All non-hydrogen atoms were refined anisotropically. All hydrogen atoms were initially idealized and then the full occupancy hydrogen atoms had their thermal parameters refined. The final difference-Fourier map was featureless indicating that the structure is both correct and complete. An empirical correction for extinction was found to be 0.00070(11) and applied.

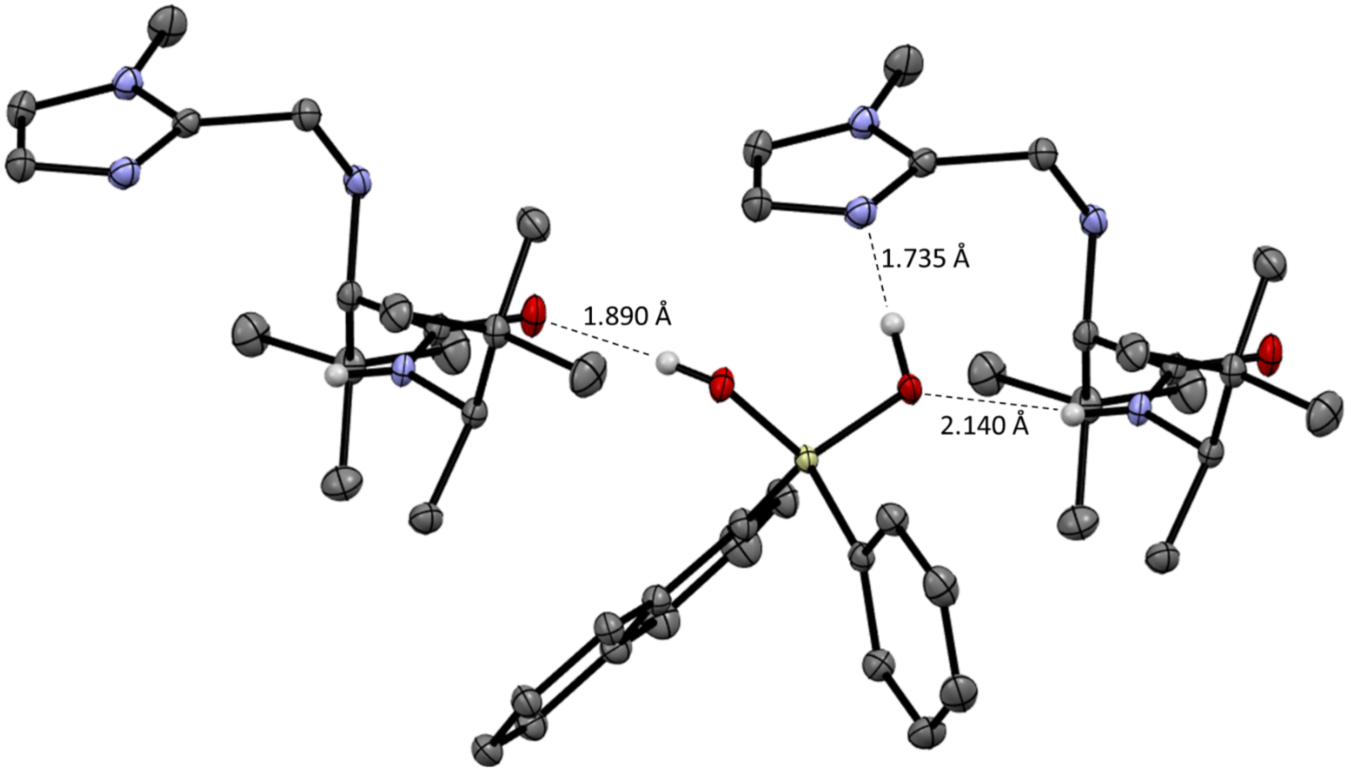

11.1 X-Ray co-crystal analysis of 1a•4

Crystals were grown using slow diffusion in DCM/pentane using crystallization tubes. Co-crystals of **1a** with **4a** were observed after 30 days.

Table S6. Crystal data and structure refinement for [$C_{33}H_{46}N_4O_3Si$]

Identification code	JF2663FMI (ATK-1)	
Empirical formula	C33 H46 N4 O3 Si	
Formula weight	574.83	
Temperature	90(2) K	
Wavelength	0.71073 Å	
Crystal system	Monoclinic	
Space group	P2₁	
Unit cell dimensions	a = 9.546(3) Å	a= 90°.
	b = 10.830(3) Å	b= 96.184(4)°.
	c = 15.606(4) Å	g = 90°.
Volume	1604.1(8) Å^3	

Z	2
Density (calculated)	1.190 Mg/m^3
Absorption coefficient	0.111 mm^{-1}
F(000)	620
Crystal size	0.528 x 0.499 x 0.414 mm^3
Crystal color and habit	Colorless Block
Diffractometer	Bruker APEX-II CCD
Theta range for data collection	2.392 to 27.919°.
Index ranges	–12<=h<=12, –14<=k<=14, –20<=l<=20
Reflections collected	14588
Independent reflections	7619 [R(int) = 0.0181]
Observed reflections (I > 2sigma(I))	7528
Completeness to theta = 25.242°	99.7 %
Absorption correction	Semi-empirical from equivalents
Max. and min. transmission	0.9609 and 0.8501
Solution method	SHELXT (Sheldrick, 2014)
Refinement method	SHELXL-2017/1 (Sheldrick, 2017) Full-matrix least-squares on F^2
Data / restraints / parameters	7619 / 1 / 546
Goodness-of-fit on F^2	1.037
Final R indices [I>2sigma(I)]	R1 = 0.0257, wR2 = 0.0701
R indices (all data)	R1 = 0.0260, wR2 = 0.0703
Absolute structure parameter	0.03(2)
Largest diff. peak and hole	0.349 and –0.152 e.Å^{-3}

Table S7. Atomic coordinates (x 10^4) and equivalent isotropic displacement parameters (Å^{2}x 10^3) for JF2663FMI. U(eq) is defined as one third of the trace of the orthogonalized U^{ij} tensor.

	x	y	z	U(eq)
Si(1)	3392(1)	4486(1)	2158(1)	12(1)
C(1)	3053(2)	6188(1)	2052(1)	16(1)
O(1)	4892(1)	4276(1)	2761(1)	16(1)
C(2)	1894(2)	6711(1)	1502(1)	16(1)
O(2)	2121(1)	3757(1)	2559(1)	17(1)
C(3)	877(2)	5962(1)	1009(1)	17(1)
C(4)	-216(2)	6490(2)	483(1)	20(1)

C(5)	-356(2)	7787(2)	415(1)	22(1)
C(6)	601(2)	8531(2)	884(1)	22(1)
C(7)	1738(2)	8026(1)	1442(1)	19(1)
C(8)	2715(2)	8797(2)	1943(1)	25(1)
C(9)	3797(2)	8297(2)	2481(1)	27(1)
C(10)	3965(2)	6996(2)	2528(1)	21(1)
C(11)	3622(1)	3722(1)	1108(1)	15(1)
C(12)	3618(2)	2419(2)	1073(1)	19(1)
C(13)	3874(2)	1787(2)	325(1)	24(1)
C(14)	4132(2)	2448(2)	-407(1)	26(1)
C(15)	4112(2)	3735(2)	-397(1)	25(1)
C(16)	3858(2)	4361(2)	356(1)	19(1)
C(21)	3714(2)	5493(2)	4660(1)	22(1)
N(21)	4842(1)	4807(1)	4436(1)	19(1)
C(22)	4142(2)	6220(2)	5353(1)	21(1)
N(22)	5557(1)	5976(1)	5564(1)	17(1)
C(23)	5936(2)	5124(1)	4995(1)	15(1)
C(24)	7413(2)	4651(1)	4988(1)	19(1)
N(24)	8340(1)	5629(1)	4708(1)	18(1)
C(25)	8070(1)	5895(1)	3779(1)	15(1)
C(26)	8819(2)	4946(1)	3255(1)	16(1)
N(26)	7989(1)	4264(1)	2688(1)	15(1)
O(26)	10122(1)	4833(1)	3386(1)	21(1)
C(27)	8515(1)	3370(1)	2085(1)	15(1)
C(28)	8472(2)	2012(1)	2418(1)	17(1)
C(29)	6473(2)	6572(2)	6254(1)	25(1)
C(30)	8448(2)	7248(1)	3564(1)	19(1)
C(31)	7502(2)	8106(2)	4038(1)	26(1)
C(32)	9997(2)	7572(2)	3852(1)	31(1)
C(33)	8140(2)	7444(2)	2586(1)	25(1)
C(34)	7684(2)	3580(2)	1199(1)	20(1)
C(35)	9305(2)	1199(2)	1846(1)	28(1)
C(36)	6950(2)	1533(2)	2391(1)	22(1)
C(37)	9198(2)	1933(2)	3347(1)	23(1)

Table S8. Bond lengths [Å] and angles [°] for JF2663FMI.

Si(1)-O(2)	1.6277(11)	C(15)-C(16)	1.399(2)
Si(1)-O(1)	1.6425(11)	C(15)-H(15)	0.99(3)
Si(1)-C(11)	1.8694(15)	C(16)-H(16)	0.94(2)
Si(1)-C(1)	1.8760(16)	C(21)-C(22)	1.365(2)
C(1)-C(10)	1.392(2)	C(21)-N(21)	1.384(2)
C(1)-C(2)	1.441(2)	C(21)-H(21)	0.96(2)
O(1)-H(1)	0.95(3)	N(21)-C(23)	1.3321(19)
C(2)-C(3)	1.425(2)	C(22)-N(22)	1.381(2)
C(2)-C(7)	1.433(2)	C(22)-H(22)	0.93(2)
O(2)-H(2)	0.77(3)	N(22)-C(23)	1.3571(18)
C(3)-C(4)	1.381(2)	N(22)-C(29)	1.462(2)
C(3)-H(3)	0.96(2)	C(23)-C(24)	1.501(2)
C(4)-C(5)	1.413(2)	C(24)-N(24)	1.475(2)
C(4)-H(4)	0.97(2)	C(24)-H(24A)	0.96(2)
C(5)-C(6)	1.370(2)	C(24)-H(24B)	0.96(2)
C(5)-H(5)	0.96(3)	N(24)-C(25)	1.4741(19)
C(6)-C(7)	1.425(2)	N(24)-H(24C)	0.83(3)
C(6)-H(6)	0.98(2)	C(25)-C(26)	1.538(2)
C(7)-C(8)	1.422(2)	C(25)-C(30)	1.554(2)
C(8)-C(9)	1.370(2)	C(25)-H(25)	0.95(2)
C(8)-H(8)	1.01(3)	C(26)-O(26)	1.2448(18)
C(9)-C(10)	1.418(2)	C(26)-N(26)	1.3433(18)
C(9)-H(9)	0.92(2)	N(26)-C(27)	1.4760(18)
C(10)-H(10)	1.00(3)	N(26)-H(26)	0.85(2)
C(11)-C(16)	1.403(2)	C(27)-C(34)	1.534(2)
C(11)-C(12)	1.411(2)	C(27)-C(28)	1.562(2)
C(12)-C(13)	1.397(2)	C(27)-H(27)	0.988(19)
C(12)-H(12)	0.90(2)	C(28)-C(35)	1.535(2)
C(13)-C(14)	1.392(3)	C(28)-C(36)	1.539(2)
C(13)-H(13)	1.05(2)	C(28)-C(37)	1.540(2)
C(14)-C(15)	1.394(3)	C(29)-H(29A)	0.9800
C(14)-H(14)	0.98(3)	C(29)-H(29B)	0.9800

C(29)-H(29C)	0.9800	C(3)-C(2)-C(7)	118.07(14)
C(30)-C(33)	1.537(2)	C(3)-C(2)-C(1)	122.12(14)
C(30)-C(32)	1.539(2)	C(7)-C(2)-C(1)	119.81(13)
C(30)-C(31)	1.539(2)	Si(1)-O(2)-H(2)	125.4(19)
C(31)-H(31A)	0.96(3)	C(4)-C(3)-C(2)	120.78(14)
C(31)-H(31B)	0.98(3)	C(4)-C(3)-H(3)	120.7(11)
C(31)-H(31C)	0.92(3)	C(2)-C(3)-H(3)	118.5(11)
C(32)-H(32A)	0.97(3)	C(3)-C(4)-C(5)	121.08(15)
C(32)-H(32B)	0.97(3)	C(3)-C(4)-H(4)	122.3(14)
C(32)-H(32C)	0.94(3)	C(5)-C(4)-H(4)	116.7(14)
C(33)-H(33A)	1.00(2)	C(6)-C(5)-C(4)	119.48(15)
C(33)-H(33B)	0.97(3)	C(6)-C(5)-H(5)	120.6(16)
C(33)-H(33C)	0.92(3)	C(4)-C(5)-H(5)	119.9(16)
C(34)-H(34A)	0.98(2)	C(5)-C(6)-C(7)	121.36(15)
C(34)-H(34B)	1.00(3)	C(5)-C(6)-H(6)	121.0(12)
C(34)-H(34C)	0.96(3)	C(7)-C(6)-H(6)	117.7(12)
C(35)-H(35A)	0.96(3)	C(8)-C(7)-C(6)	121.46(15)
C(35)-H(35B)	0.97(3)	C(8)-C(7)-C(2)	119.33(14)
C(35)-H(35C)	0.95(3)	C(6)-C(7)-C(2)	119.22(14)
C(36)-H(36A)	0.96(3)	C(9)-C(8)-C(7)	120.76(15)
C(36)-H(36B)	0.98(2)	C(9)-C(8)-H(8)	122.7(13)
C(36)-H(36C)	0.92(2)	C(7)-C(8)-H(8)	116.6(13)
C(37)-H(37A)	1.01(2)	C(8)-C(9)-C(10)	119.84(15)
C(37)-H(37B)	0.97(2)	C(8)-C(9)-H(9)	119.4(14)
C(37)-H(37C)	0.97(3)	C(10)-C(9)-H(9)	120.8(14)
		C(1)-C(10)-C(9)	122.40(15)
O(2)-Si(1)-O(1)	110.63(6)	C(1)-C(10)-H(10)	119.4(15)
O(2)-Si(1)-C(11)	106.55(6)	C(9)-C(10)-H(10)	118.2(15)
O(1)-Si(1)-C(11)	105.26(6)	C(16)-C(11)-C(12)	117.44(14)
O(2)-Si(1)-C(1)	112.44(6)	C(16)-C(11)-Si(1)	124.06(12)
O(1)-Si(1)-C(1)	108.57(6)	C(12)-C(11)-Si(1)	118.45(11)
C(11)-Si(1)-C(1)	113.16(7)	C(13)-C(12)-C(11)	121.47(15)
C(10)-C(1)-C(2)	117.82(14)	C(13)-C(12)-H(12)	119.6(13)
C(10)-C(1)-Si(1)	118.59(11)	C(11)-C(12)-H(12)	118.9(13)
C(2)-C(1)-Si(1)	123.59(11)	C(14)-C(13)-C(12)	119.69(16)
Si(1)-O(1)-H(1)	114.9(17)	C(14)-C(13)-H(13)	120.2(12)

C(12)-C(13)-H(13)	120.0(12)	C(26)-C(25)-H(25)	108.4(12)
C(13)-C(14)-C(15)	120.10(15)	C(30)-C(25)-H(25)	108.8(12)
C(13)-C(14)-H(14)	122.1(16)	O(26)-C(26)-N(26)	124.30(13)
C(15)-C(14)-H(14)	117.7(16)	O(26)-C(26)-C(25)	119.46(13)
C(14)-C(15)-C(16)	119.80(16)	N(26)-C(26)-C(25)	116.22(12)
C(14)-C(15)-H(15)	120.3(16)	C(26)-N(26)-C(27)	124.25(12)
C(16)-C(15)-H(15)	119.9(16)	C(26)-N(26)-H(26)	118.0(15)
C(15)-C(16)-C(11)	121.46(16)	C(27)-N(26)-H(26)	117.6(15)
C(15)-C(16)-H(16)	122.4(13)	N(26)-C(27)-C(34)	107.42(12)
C(11)-C(16)-H(16)	116.2(13)	N(26)-C(27)-C(28)	112.44(11)
C(22)-C(21)-N(21)	109.59(14)	C(34)-C(27)-C(28)	114.21(12)
C(22)-C(21)-H(21)	130.8(14)	N(26)-C(27)-H(27)	106.3(11)
N(21)-C(21)-H(21)	119.6(14)	C(34)-C(27)-H(27)	110.3(11)
C(23)-N(21)-C(21)	105.84(13)	C(28)-C(27)-H(27)	105.9(12)
C(21)-C(22)-N(22)	106.28(14)	C(35)-C(28)-C(36)	109.68(14)
C(21)-C(22)-H(22)	131.3(14)	C(35)-C(28)-C(37)	107.85(14)
N(22)-C(22)-H(22)	122.3(13)	C(36)-C(28)-C(37)	109.55(13)
C(23)-N(22)-C(22)	107.31(13)	C(35)-C(28)-C(27)	108.21(13)
C(23)-N(22)-C(29)	126.96(13)	C(36)-C(28)-C(27)	111.55(12)
C(22)-N(22)-C(29)	125.69(13)	C(37)-C(28)-C(27)	109.92(12)
N(21)-C(23)-N(22)	110.99(13)	N(22)-C(29)-H(29A)	109.5
N(21)-C(23)-C(24)	125.31(13)	N(22)-C(29)-H(29B)	109.5
N(22)-C(23)-C(24)	123.66(13)	H(29A)-C(29)-H(29B)	109.5
N(24)-C(24)-C(23)	110.54(12)	N(22)-C(29)-H(29C)	109.5
N(24)-C(24)-H(24A)	108.1(13)	H(29A)-C(29)-H(29C)	109.5
C(23)-C(24)-H(24A)	110.5(13)	H(29B)-C(29)-H(29C)	109.5
N(24)-C(24)-H(24B)	113.9(13)	C(33)-C(30)-C(32)	109.49(15)
C(23)-C(24)-H(24B)	108.6(13)	C(33)-C(30)-C(31)	109.48(14)
H(24A)-C(24)-H(24B)	105.1(19)	C(32)-C(30)-C(31)	108.50(14)
C(25)-N(24)-C(24)	112.59(12)	C(33)-C(30)-C(25)	108.70(12)
C(25)-N(24)-H(24C)	107.8(17)	C(32)-C(30)-C(25)	112.96(13)
C(24)-N(24)-H(24C)	109.1(18)	C(31)-C(30)-C(25)	107.66(13)
N(24)-C(25)-C(26)	110.51(12)	C(30)-C(31)-H(31A)	110.7(17)
N(24)-C(25)-C(30)	112.13(12)	C(30)-C(31)-H(31B)	108.1(16)
C(26)-C(25)-C(30)	112.53(12)	H(31A)-C(31)-H(31B)	111(2)
N(24)-C(25)-H(25)	104.1(12)	C(30)-C(31)-H(31C)	110.7(16)

H(31A)-C(31)-H(31C)	108(2)	H(34B)-C(34)-H(34C)	105.8(19)
H(31B)-C(31)-H(31C)	109(2)	C(28)-C(35)-H(35A)	109.9(16)
C(30)-C(32)-H(32A)	112.6(15)	C(28)-C(35)-H(35B)	110.7(16)
C(30)-C(32)-H(32B)	105.9(16)	H(35A)-C(35)-H(35B)	104(2)
H(32A)-C(32)-H(32B)	109(2)	C(28)-C(35)-H(35C)	115.6(17)
C(30)-C(32)-H(32C)	111.6(18)	H(35A)-C(35)-H(35C)	109(2)
H(32A)-C(32)-H(32C)	108(2)	H(35B)-C(35)-H(35C)	107(2)
H(32B)-C(32)-H(32C)	110(2)	C(28)-C(36)-H(36A)	110.4(16)
C(30)-C(33)-H(33A)	112.7(14)	C(28)-C(36)-H(36B)	111.3(13)
C(30)-C(33)-H(33B)	111.8(15)	H(36A)-C(36)-H(36B)	103(2)
H(33A)-C(33)-H(33B)	109(2)	C(28)-C(36)-H(36C)	111.7(14)
C(30)-C(33)-H(33C)	113.7(15)	H(36A)-C(36)-H(36C)	110(2)
H(33A)-C(33)-H(33C)	104(2)	H(36B)-C(36)-H(36C)	110.3(19)
H(33B)-C(33)-H(33C)	105(2)	C(28)-C(37)-H(37A)	112.1(13)
C(27)-C(34)-H(34A)	113.8(13)	C(28)-C(37)-H(37B)	110.4(13)
C(27)-C(34)-H(34B)	112.6(15)	H(37A)-C(37)-H(37B)	110.2(18)
H(34A)-C(34)-H(34B)	107(2)	C(28)-C(37)-H(37C)	112.3(15)
C(27)-C(34)-H(34C)	109.1(13)	H(37A)-C(37)-H(37C)	106(2)
H(34A)-C(34)-H(34C)	108.1(19)	H(37B)-C(37)-H(37C)	106(2)

Symmetry transformations used to generate equivalent atoms:

Table S9. Anisotropic displacement parameters ($Å^2 x 10^3$) for JF2663FMI. The anisotropic displacement factor exponent takes the form: $-2p^2[h^2 a^{*2}U^{11} + ... + 2 h k a^* b^* U^{12}]$

	U^{11}	U^{22}	U^{33}	U^{23}	U^{13}	U^{12}
Si(1)	10(1)	14(1)	13(1)	-1(1)	1(1)	1(1)
C(1)	15(1)	15(1)	18(1)	0(1)	2(1)	1(1)
O(1)	11(1)	22(1)	14(1)	-2(1)	0(1)	2(1)
C(2)	16(1)	17(1)	16(1)	1(1)	4(1)	1(1)
O(2)	12(1)	18(1)	23(1)	1(1)	6(1)	1(1)
C(3)	16(1)	18(1)	19(1)	0(1)	2(1)	2(1)
C(4)	17(1)	24(1)	18(1)	0(1)	0(1)	3(1)
C(5)	21(1)	26(1)	18(1)	4(1)	2(1)	7(1)
C(6)	25(1)	18(1)	23(1)	3(1)	5(1)	6(1)

C(7)	21(1)	17(1)	20(1)	1(1)	5(1)	2(1)
C(8)	28(1)	14(1)	32(1)	0(1)	2(1)	-1(1)
C(9)	27(1)	19(1)	33(1)	-4(1)	-3(1)	-5(1)
C(10)	17(1)	20(1)	24(1)	-1(1)	-1(1)	-1(1)
C(11)	11(1)	19(1)	16(1)	-2(1)	-1(1)	1(1)
C(12)	18(1)	19(1)	19(1)	-1(1)	-1(1)	2(1)
C(13)	25(1)	21(1)	25(1)	-6(1)	-3(1)	5(1)
C(14)	26(1)	35(1)	18(1)	-8(1)	0(1)	6(1)
C(15)	25(1)	33(1)	17(1)	1(1)	4(1)	1(1)
C(16)	18(1)	22(1)	19(1)	1(1)	2(1)	0(1)
C(21)	16(1)	30(1)	20(1)	2(1)	3(1)	1(1)
N(21)	18(1)	24(1)	15(1)	-1(1)	3(1)	-2(1)
C(22)	18(1)	24(1)	23(1)	0(1)	7(1)	4(1)
N(22)	18(1)	18(1)	16(1)	-3(1)	3(1)	1(1)
C(23)	18(1)	16(1)	13(1)	0(1)	3(1)	0(1)
C(24)	19(1)	20(1)	17(1)	1(1)	3(1)	5(1)
N(24)	12(1)	26(1)	17(1)	-2(1)	-1(1)	2(1)
C(25)	11(1)	18(1)	16(1)	-3(1)	1(1)	0(1)
C(26)	13(1)	18(1)	17(1)	-1(1)	4(1)	-1(1)
N(26)	10(1)	17(1)	17(1)	-3(1)	2(1)	1(1)
O(26)	11(1)	27(1)	25(1)	-8(1)	2(1)	1(1)
C(27)	12(1)	17(1)	16(1)	-2(1)	3(1)	1(1)
C(28)	14(1)	17(1)	19(1)	-2(1)	3(1)	2(1)
C(29)	25(1)	30(1)	20(1)	-10(1)	1(1)	-2(1)
C(30)	18(1)	17(1)	23(1)	-3(1)	1(1)	-3(1)
C(31)	30(1)	20(1)	29(1)	-6(1)	2(1)	4(1)
C(32)	21(1)	26(1)	43(1)	-3(1)	-3(1)	-9(1)
C(33)	28(1)	23(1)	25(1)	3(1)	5(1)	-3(1)
C(34)	20(1)	24(1)	16(1)	-1(1)	2(1)	0(1)
C(35)	32(1)	21(1)	34(1)	-4(1)	12(1)	6(1)
C(36)	18(1)	20(1)	27(1)	2(1)	2(1)	-4(1)
C(37)	21(1)	23(1)	24(1)	3(1)	-2(1)	3(1)

Table S10. Hydrogen coordinates (x 10^4) and isotropic displacement parameters ($Å^2$x 10^3) for JF2663FMI.

	x	y	z	U(eq)
H(1)	4860(30)	4420(30)	3358(18)	49(7)
H(2)	1570(30)	4060(20)	2817(15)	34(7)
H(3)	950(20)	5077(19)	1063(12)	11(4)
H(4)	-920(20)	6000(20)	141(14)	27(5)
H(5)	-1110(30)	8140(30)	36(16)	36(6)
H(6)	530(20)	9440(20)	840(13)	24(5)
H(8)	2560(20)	9720(20)	1886(14)	28(5)
H(9)	4400(20)	8810(20)	2814(14)	24(5)
H(10)	4760(30)	6660(20)	2925(16)	33(6)
H(12)	3470(20)	1990(19)	1551(13)	13(4)
H(13)	3800(20)	820(20)	303(14)	26(5)
H(14)	4300(30)	2040(30)	-943(16)	36(6)
H(15)	4270(30)	4210(30)	-921(17)	42(7)
H(16)	3810(20)	5230(20)	383(14)	22(5)
H(21)	2800(20)	5400(20)	4334(14)	26(5)
H(22)	3640(20)	6750(20)	5677(14)	25(5)
H(24A)	7790(20)	4390(20)	5557(14)	26(5)
H(24B)	7390(20)	3920(20)	4641(14)	23(5)
H(24C)	9170(30)	5400(20)	4809(16)	33(6)
H(25)	7080(20)	5785(18)	3653(12)	13(4)
H(26)	7110(20)	4390(20)	2650(13)	20(5)
H(27)	9520(20)	3565(19)	2063(12)	14(4)
H(29A)	7314	6064	6400	75(11)
H(29B)	5965	6664	6763	75(11)
H(29C)	6754	7388	6061	110(15)
H(31A)	6530(30)	7880(30)	3917(17)	40(7)
H(31B)	7660(30)	8960(30)	3851(17)	43(7)
H(31C)	7740(30)	8060(20)	4627(17)	33(6)
H(32A)	10250(30)	7440(20)	4464(17)	36(6)
H(32B)	10090(30)	8440(30)	3728(17)	40(7)
H(32C)	10620(30)	7100(30)	3553(19)	48(8)
H(33A)	8760(30)	6930(20)	2247(15)	28(5)

H(33B)	8240(30)	8310(30)	2428(17)	39(6)
H(33C)	7240(30)	7230(20)	2370(16)	32(6)
H(34A)	6670(20)	3470(20)	1196(14)	29(6)
H(34B)	8000(30)	3030(30)	747(16)	36(6)
H(34C)	7850(20)	4410(20)	1011(14)	27(5)
H(35A)	9420(30)	380(30)	2089(17)	38(6)
H(35B)	10260(30)	1500(30)	1848(17)	34(6)
H(35C)	8920(30)	1130(30)	1259(18)	44(7)
H(36A)	6940(30)	720(30)	2623(17)	37(6)
H(36B)	6520(20)	1420(20)	1799(14)	22(5)
H(36C)	6400(20)	2040(20)	2686(14)	24(5)
H(37A)	8670(20)	2410(20)	3766(14)	26(5)
H(37B)	10160(20)	2220(20)	3372(14)	24(5)
H(37C)	9250(30)	1090(30)	3562(16)	40(7)

Table S11. Torsion angles [°] for JF2663FMI.

O(2)-Si(1)-C(1)-C(10)	116.85(12)	C(1)-C(2)-C(7)-C(8)	-1.4(2)
O(1)-Si(1)-C(1)-C(10)	-5.90(14)	C(3)-C(2)-C(7)-C(6)	-1.3(2)
C(11)-Si(1)-C(1)-C(10)	-122.37(12)	C(1)-C(2)-C(7)-C(6)	178.83(13)
O(2)-Si(1)-C(1)-C(2)	-63.12(13)	C(6)-C(7)-C(8)-C(9)	179.60(16)
O(1)-Si(1)-C(1)-C(2)	174.14(11)	C(2)-C(7)-C(8)-C(9)	-0.2(2)
C(11)-Si(1)-C(1)-C(2)	57.66(13)	C(7)-C(8)-C(9)-C(10)	1.3(3)
C(10)-C(1)-C(2)-C(3)	-178.06(14)	C(2)-C(1)-C(10)-C(9)	-0.7(2)
Si(1)-C(1)-C(2)-C(3)	1.9(2)	Si(1)-C(1)-C(10)-C(9)	179.35(14)
C(10)-C(1)-C(2)-C(7)	1.8(2)	C(8)-C(9)-C(10)-C(1)	-0.9(3)
Si(1)-C(1)-C(2)-C(7)	-178.23(11)	O(2)-Si(1)-C(11)-C(16)	138.56(12)
C(7)-C(2)-C(3)-C(4)	0.6(2)	O(1)-Si(1)-C(11)-C(16)	-103.92(12)
C(1)-C(2)-C(3)-C(4)	-179.49(14)	C(1)-Si(1)-C(11)-C(16)	14.49(14)
C(2)-C(3)-C(4)-C(5)	0.3(2)	O(2)-Si(1)-C(11)-C(12)	-44.19(13)
C(3)-C(4)-C(5)-C(6)	-0.5(3)	O(1)-Si(1)-C(11)-C(12)	73.33(12)
C(4)-C(5)-C(6)-C(7)	-0.2(2)	C(1)-Si(1)-C(11)-C(12)	-168.25(11)
C(5)-C(6)-C(7)-C(8)	-178.66(15)	C(16)-C(11)-C(12)-C(13)	1.5(2)
C(5)-C(6)-C(7)-C(2)	1.1(2)	Si(1)-C(11)-C(12)-C(13)	-175.89(12)
C(3)-C(2)-C(7)-C(8)	178.45(14)	C(11)-C(12)-C(13)-C(14)	-0.3(2)

C(12)-C(13)-C(14)-C(15)	-1.1(3)
C(13)-C(14)-C(15)-C(16)	1.3(3)
C(14)-C(15)-C(16)-C(11)	0.0(2)
C(12)-C(11)-C(16)-C(15)	-1.4(2)
Si(1)-C(11)-C(16)-C(15)	175.89(12)
C(22)-C(21)-N(21)-C(23)	0.06(18)
N(21)-C(21)-C(22)-N(22)	-0.23(18)
C(21)-C(22)-N(22)-C(23)	0.30(18)
C(21)-C(22)-N(22)-C(29)	178.10(15)
C(21)-N(21)-C(23)-N(22)	0.13(17)
C(21)-N(21)-C(23)-C(24)	-177.65(14)
C(22)-N(22)-C(23)-N(21)	-0.28(17)
C(29)-N(22)-C(23)-N(21)	-178.03(15)
C(22)-N(22)-C(23)-C(24)	177.55(14)
C(29)-N(22)-C(23)-C(24)	-0.2(2)
N(21)-C(23)-C(24)-N(24)	108.44(16)
N(22)-C(23)-C(24)-N(24)	-69.07(18)
C(23)-C(24)-N(24)-C(25)	-72.55(15)
C(24)-N(24)-C(25)-C(26)	-81.42(15)
C(24)-N(24)-C(25)-C(30)	152.15(12)
N(24)-C(25)-C(26)-O(26)	-59.01(18)
C(30)-C(25)-C(26)-O(26)	67.19(18)
N(24)-C(25)-C(26)-N(26)	119.46(14)
C(30)-C(25)-C(26)-N(26)	-114.33(14)
O(26)-C(26)-N(26)-C(27)	-6.1(2)
C(25)-C(26)-N(26)-C(27)	175.48(12)
C(26)-N(26)-C(27)-C(34)	-134.61(14)
C(26)-N(26)-C(27)-C(28)	98.92(16)
N(26)-C(27)-C(28)-C(35)	-168.74(13)
C(34)-C(27)-C(28)-C(35)	68.53(16)
N(26)-C(27)-C(28)-C(36)	70.53(15)
C(34)-C(27)-C(28)-C(36)	-52.21(16)
N(26)-C(27)-C(28)-C(37)	-51.19(16)
C(34)-C(27)-C(28)-C(37)	-173.93(12)
N(24)-C(25)-C(30)-C(33)	179.85(13)
C(26)-C(25)-C(30)-C(33)	54.52(16)
N(24)-C(25)-C(30)-C(32)	58.12(17)
C(26)-C(25)-C(30)-C(32)	-67.21(17)
N(24)-C(25)-C(30)-C(31)	-61.65(15)
C(26)-C(25)-C(30)-C(31)	173.03(12)

Symmetry transformations used to generate equivalent atoms:

Table S12. Hydrogen bonds for JF2663FMI [Å and °].

D-H...A	d(D-H)	d(H...A)	d(D...A)	<(DHA)
O(1)-H(1)...N(21)	0.95(3)	1.73(3)	2.6813(18)	175(3)
O(2)-H(2)...O(26)#1	0.77(3)	1.91(3)	2.6829(16)	176(3)
N(26)-H(26)...O(1)	0.85(2)	2.14(2)	2.9703(18)	165(2)
C(27)-H(27)...O(2)#2	0.988(19)	2.53(2)	3.4680(19)	159.0(15)

Symmetry transformations used to generate equivalent atoms:

#1 x-1,y,z #2 x+1,y,z

12. References

(1) Galya, L. G.; Mccord, E. F.; Adamsons, K. Use of Paramagnetic Relaxation Agents in the Characterization of Acrylic Polymers: Application of Chromium (III) Acetylacetonate and Iron (III) Acetylacetonate. *Int. J. Polym. Anal. Charact.* **1996**, *2* (3), 293–303. https://doi.org/10.1080/10236669608233916.

(2) Gao, J.; Mai, P. L.; Ge, Y.; Yuan, W.; Li, Y.; He, C. Copper-Catalyzed Desymmetrization of Prochiral Silanediols to Silicon-Stereogenic Silanols. *ACS Catal.* **2022**, *12* (14), 8476–8483. https://doi.org/10.1021/acscatal.2c02482.

(3) Thordarson, P. Determining Association Constants from Titration Experiments in Supramolecular Chemistry. *Chem Soc Rev* **2011**, *40* (3), 1305–1323. https://doi.org/10.1039/C0CS00062K.

(4) Tran, N. T.; Wilson, S. O.; Franz, A. K. Cooperative Hydrogen-Bonding Effects in Silanediol Catalysis. *Org. Lett.* **2012**, *14* (1), 186–189. https://doi.org/10.1021/ol202971m.

(5) Kondo, S.-I.; Harada, T.; Tanaka, R.; Unno, M. Anion Recognition by a Silanediol-Based Receptor. *Org Lett* **2006**, *8* (20), 4621–4624.

(6) Walsh, S.; Diamond, D. Non-Linear Curve Fitting Using Microsoft Excel Solver. *Talanta* **1995**, *42* (4), 561–572. https://doi.org/10.1016/0039-9140(95)01446-I.

(7) Schneider, H.-J.; 1935-; Dürr, Heinz. Frontiers in Supramolecular Organic Chemistry and Photochemistry. **1991**, 485. https://doi.org/10.3/JQUERY-UI.JS.

(8) Thordarson, P. Determining Association Constants from Titration Experiments in Supramolecular Chemistry. *Chem Soc Rev* **2011**, *40* (3), 1305–1323. https://doi.org/10.1039/C0CS00062K.

(9) Zhao, Y.; Rodrigo, J.; Hoveyda, A. H.; Snapper, M. L. Enantioselective Silyl Protection of Alcohols Catalysed by an Amino-Acid-Based Small Molecule. *Nature* **2006**, *443* (7107), 67–70. https://doi.org/10.1038/nature05102.

(10) Wang, K.; Zhou, J.; Jiang, Y.; Zhang, M.; Wang, C.; Xue, D.; Tang, W.; Sun, H.; Xiao, J.; Li, C. Selective Manganese-Catalyzed Oxidation of Hydrosilanes to

Silanols under Neutral Reaction Conditions. *Angew. Chem. Int. Ed.* **2019**, *58* (19), 6380–6384. https://doi.org/10.1002/ANIE.201900342.
(11) Sommer, L. H.; Tyler, L. J. Steric Effects of the T-Butyl Group in Organosilicon Compounds. *J. Am. Chem. Soc.* **1954**, *76* (4), 1030–1033. https://doi.org/10.1021/ja01633a032.
(12) Bruker (2019) APEX3 (Version 2019.0) and (2016) SAINT (Version 8.37a). Bruker AXS Inc., Madison, Wisconsin, USA. .

12. Author contributions

JJD designed and performed synthesis, optimization, scope, NMR binding studies, acid scavenger study, crystallization experiments, writing and editing the manuscript, and supplemental information. ABS contributed to synthesis, scope, catalyst analog **4c**, NMR binding studies and contributed to the manuscript and supplemental information. ATK formulated the original desymmetrization concept and initial screening and optimization experiments. JAF solved the crystal structure. AKF contributed to original concept, experimental design, analysis, writing and editing the manuscript.

Catalysts

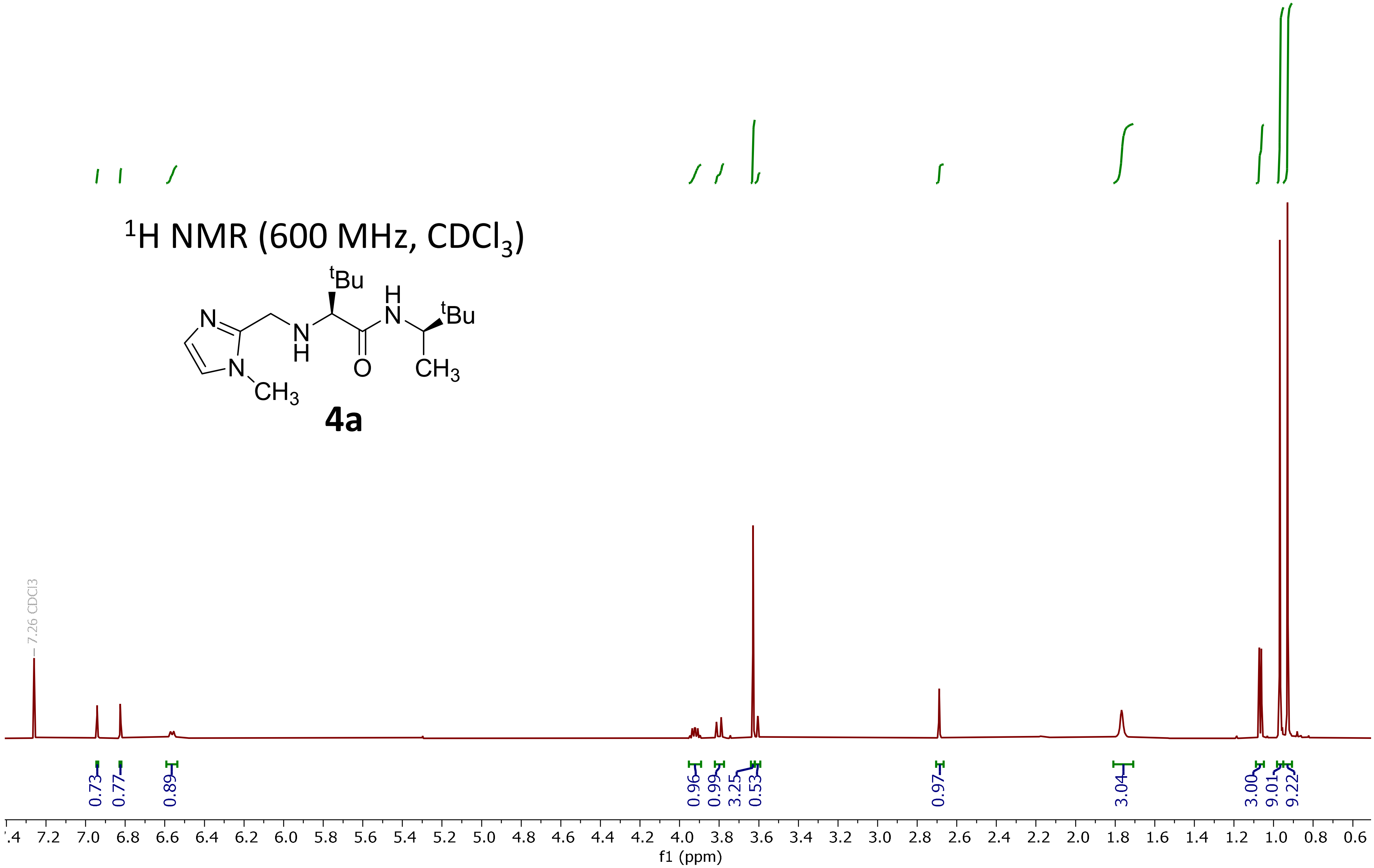
^1H NMR (600 MHz, $CDCl_3$)
tBu
H
N
tBu
N
N
H
O
N
CH3
CH3
4a
7.26 CDCl3
0.73
0.77
0.89
0.96
0.99
3.25
0.53
0.97
3.04
3.00
9.01
9.22
7.2 7.0 6.8 6.6 6.4 6.2 6.0 5.8 5.6 5.4 5.2 5.0 4.8 4.6 4.4 4.2 4.0 3.8 3.6 3.4 3.2 3.0 2.8 2.6 2.4 2.2 2.0 1.8 1.6 1.4 1.2 1.0 0.8 0.6
f1 (ppm)

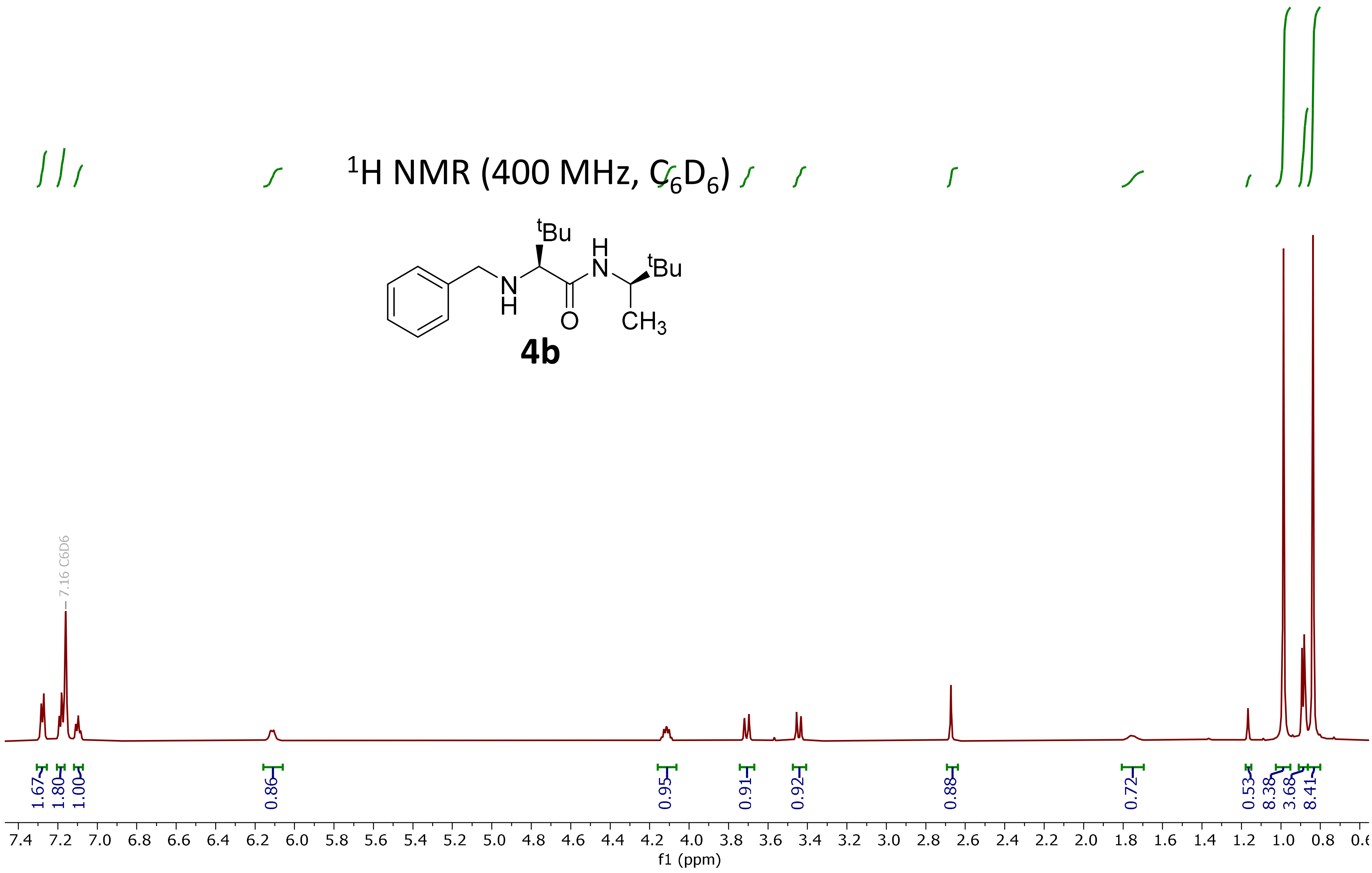

^{1}H NMR (400 MHz, C_6D_6)
^{t}Bu
H
N
^{t}Bu
N
H
O
CH_3
4b
7.16 C6D6
1.67
1.80
1.00
0.86
0.95
0.91
0.92
0.88
0.72
0.53
8.38
3.68
8.41
7.4 7.2 7.0 6.8 6.6 6.4 6.2 6.0 5.8 5.6 5.4 5.2 5.0 4.8 4.6 4.4 4.2 4.0 3.8 3.6 3.4 3.2 3.0 2.8 2.6 2.4 2.2 2.0 1.8 1.6 1.4 1.2 1.0 0.8
f1 (ppm)

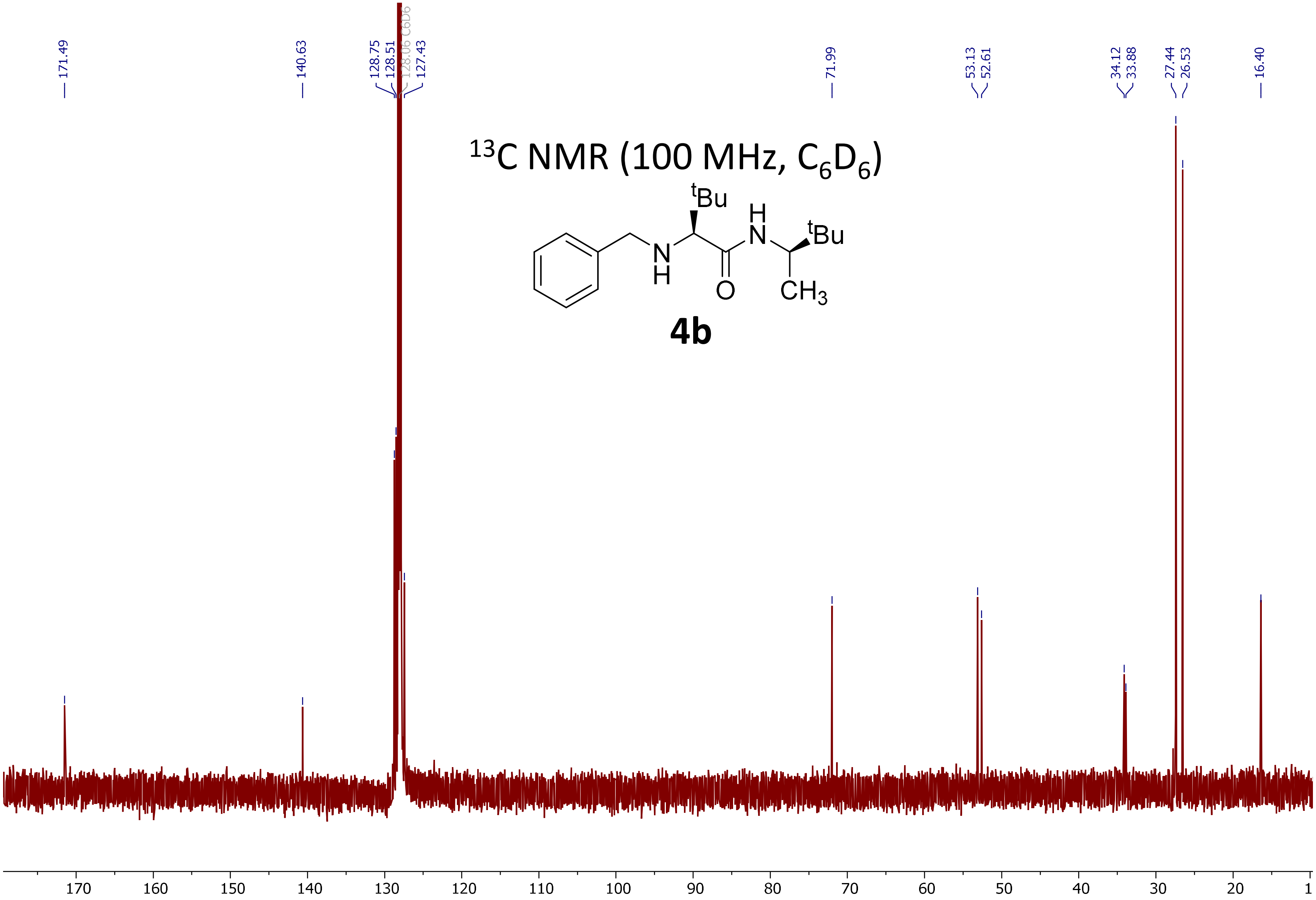
171.49
140.63
128.75
128.51
128.06 C6D6
127.43
71.99
53.13
52.61
34.12
33.88
27.44
26.53
16.40
^{13}C NMR (100 MHz, C_6D_6)
tBu
H
N
tBu
N
H
O
CH3
4b
170
160
150
140
130
120
110
100
90
80
70
60
50
40
30
20
1

^{1}H NMR (400 MHz, $CDCl_3$)

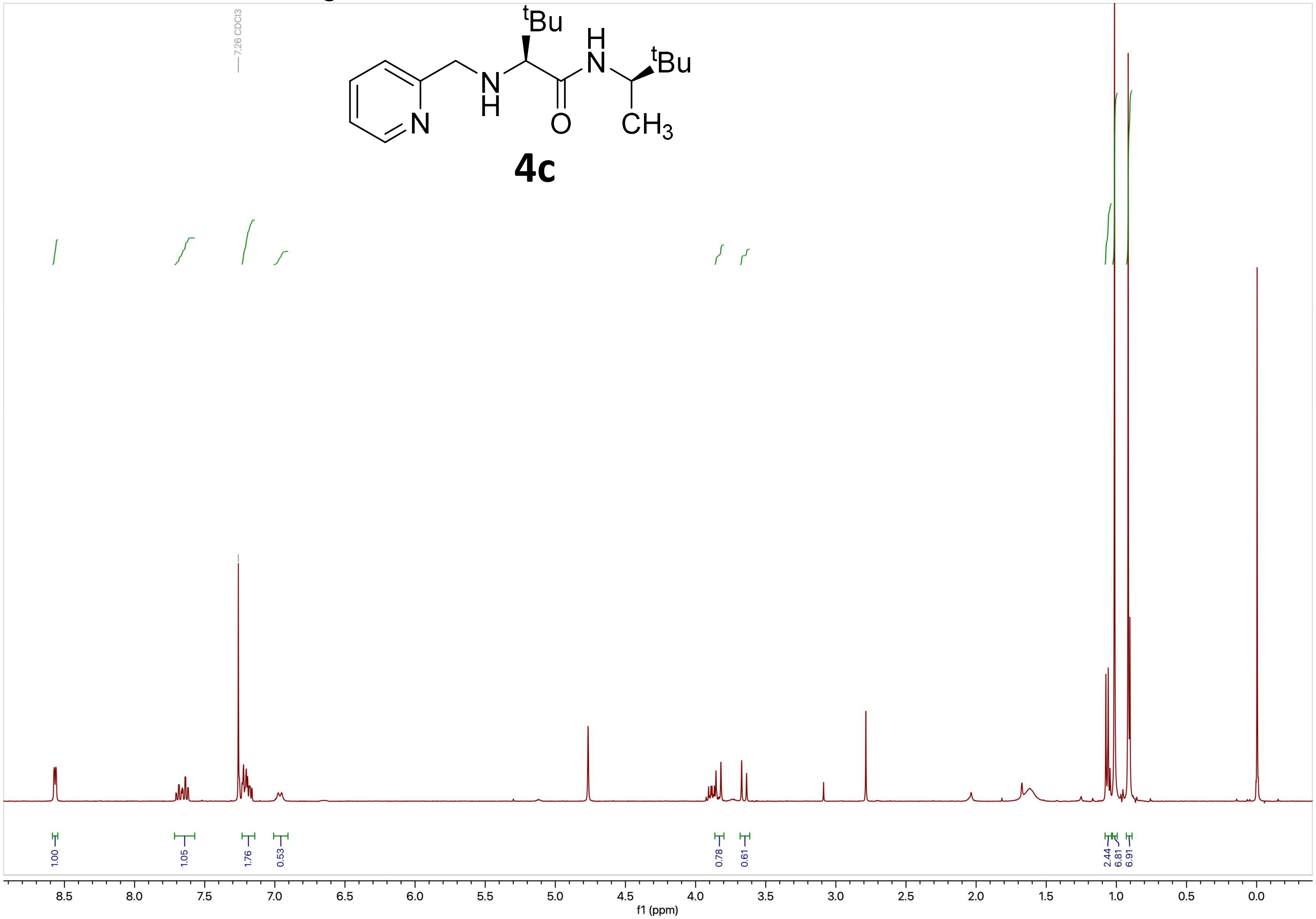

^{13}C NMR (100 MHz, $CDCl_3$)

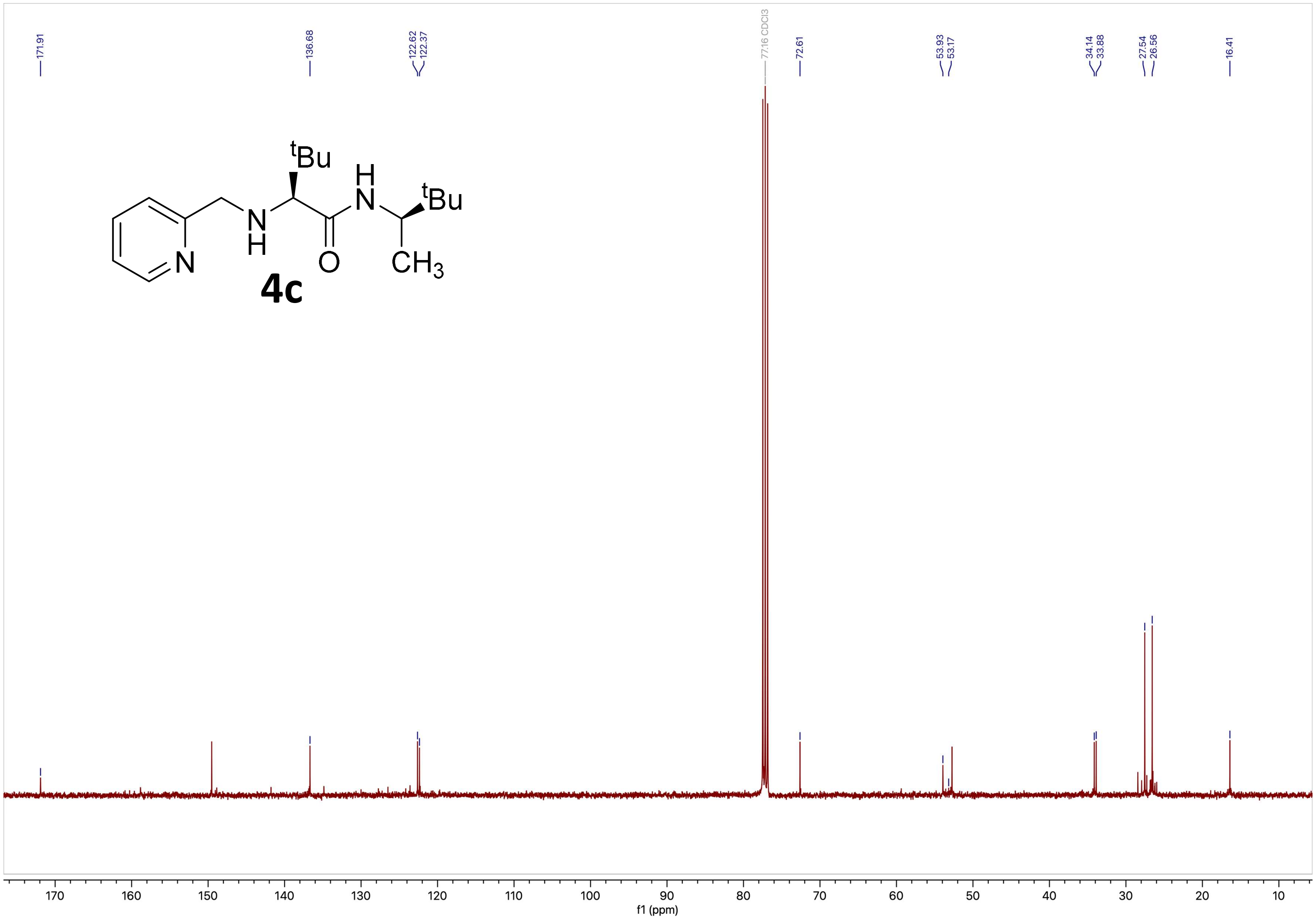

Silanes

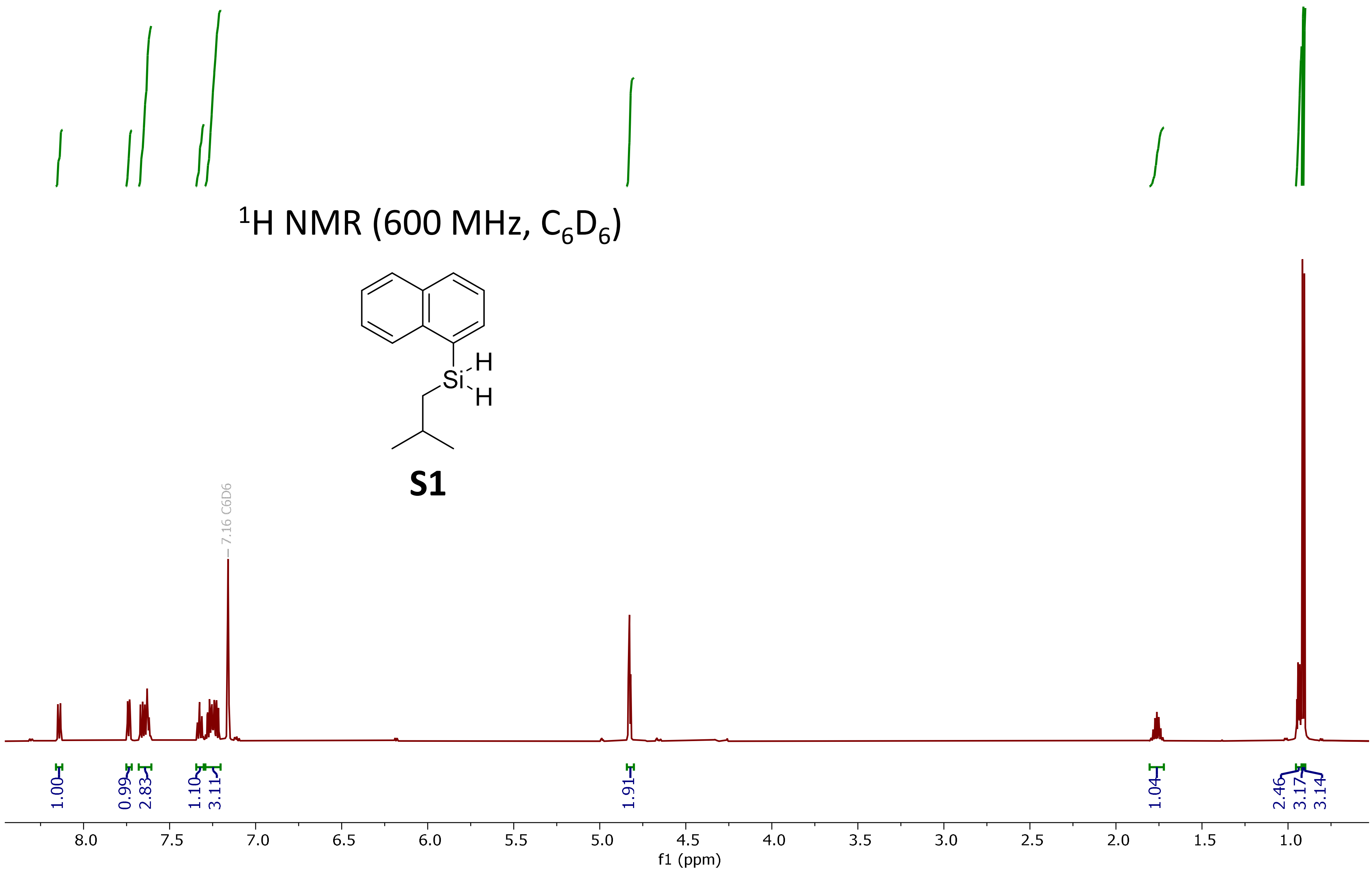

¹H NMR (600 MHz, C6D6)
Si
H
H
S1
7.16 C6D6
1.00
0.99
2.83
1.10
3.11
1.91
1.04
2.46
3.17
3.14
8.0
7.5
7.0
6.5
6.0
5.5
5.0
4.5
4.0
3.5
3.0
2.5
2.0
1.5
1.0
f1 (ppm)

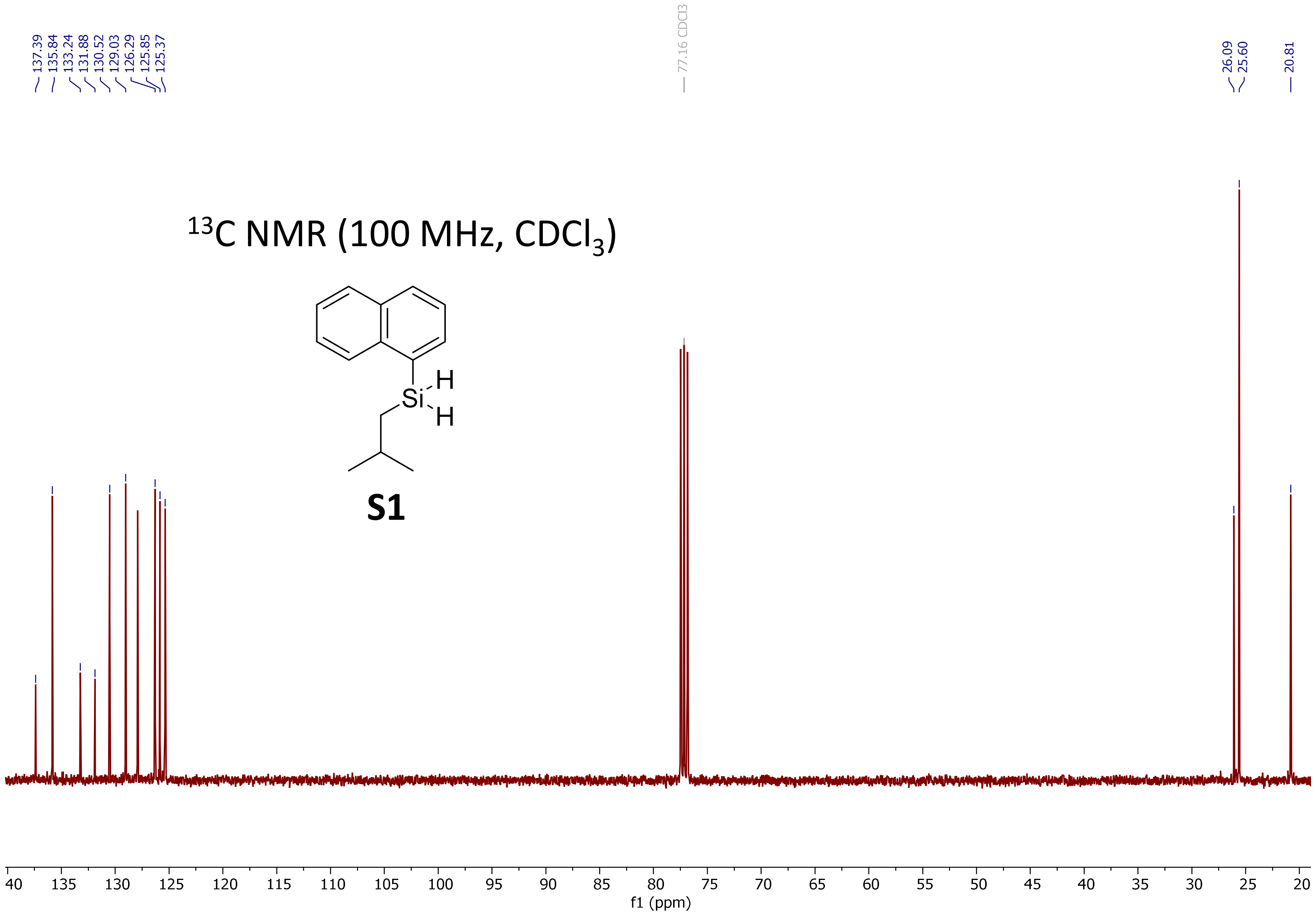

137.39
135.84
133.24
131.88
130.52
129.03
126.29
125.85
125.37
77.16 CDCl3
26.09
25.60
20.81
^{13}C NMR (100 MHz, $CDCl_3$)
Si
H
H
S1
140 135 130 125 120 115 110 105 100 95 90 85 80 75 70 65 60 55 50 45 40 35 30 25 20
f1 (ppm)

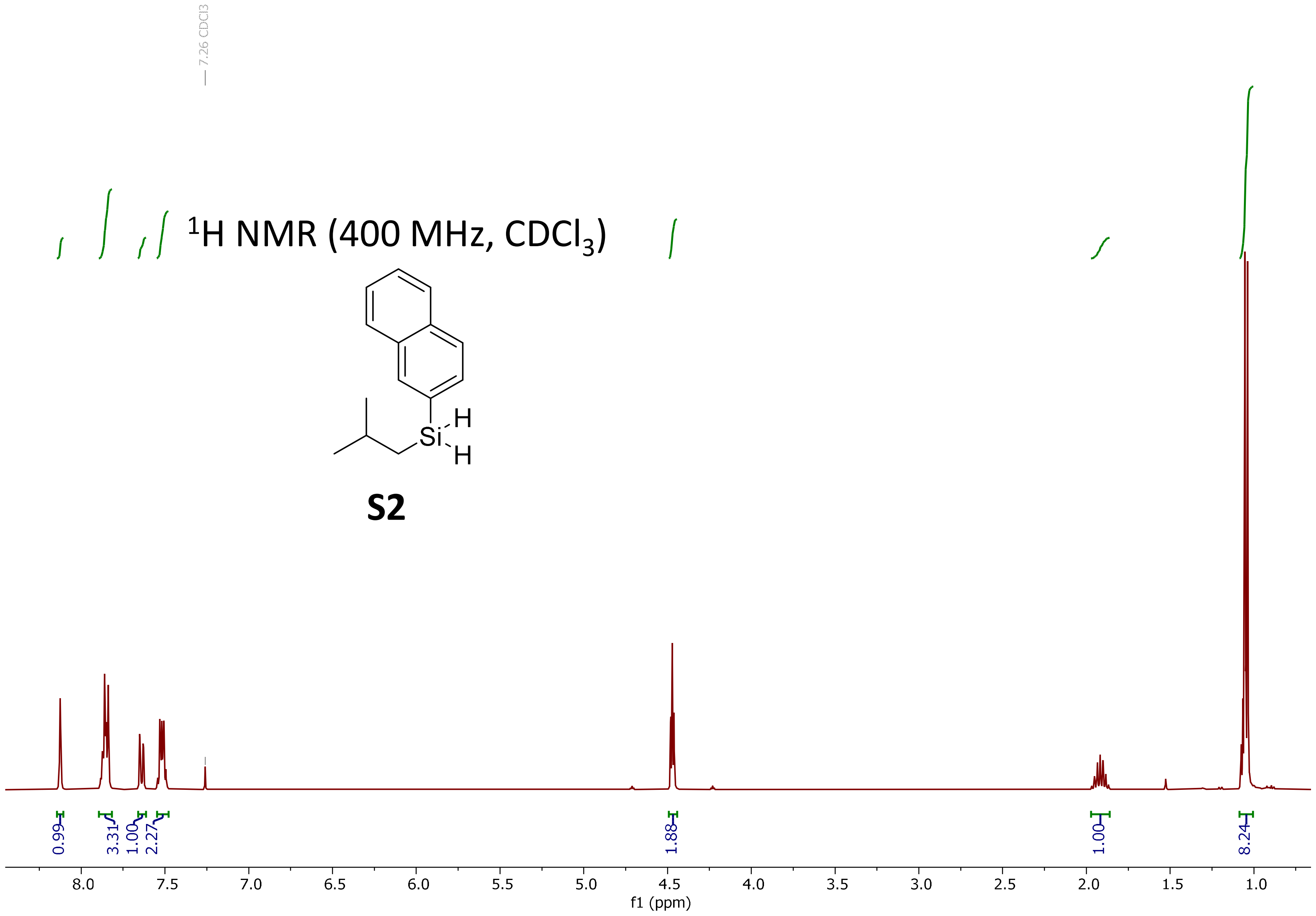

7.26 CDCl3
^{1}H NMR (400 MHz, $CDCl_3$)
Si
H
H
S2
0.99
3.31
1.00
2.27
1.88
1.00
8.24
8.0
7.5
7.0
6.5
6.0
5.5
5.0
4.5
4.0
3.5
3.0
2.5
2.0
1.5
1.0
f1 (ppm)

^{13}C NMR (100 MHz, $CDCl_3$)

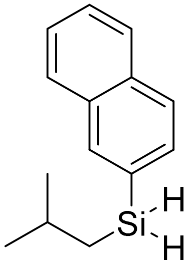

S2

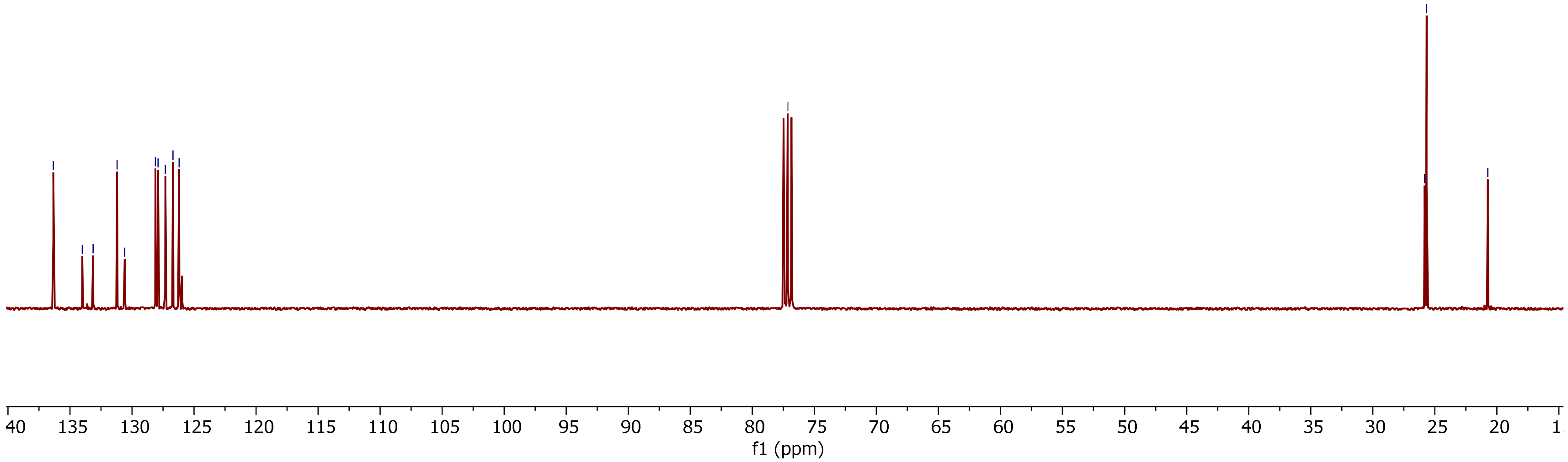

^1H NMR (400 MHz, $CDCl_3$)

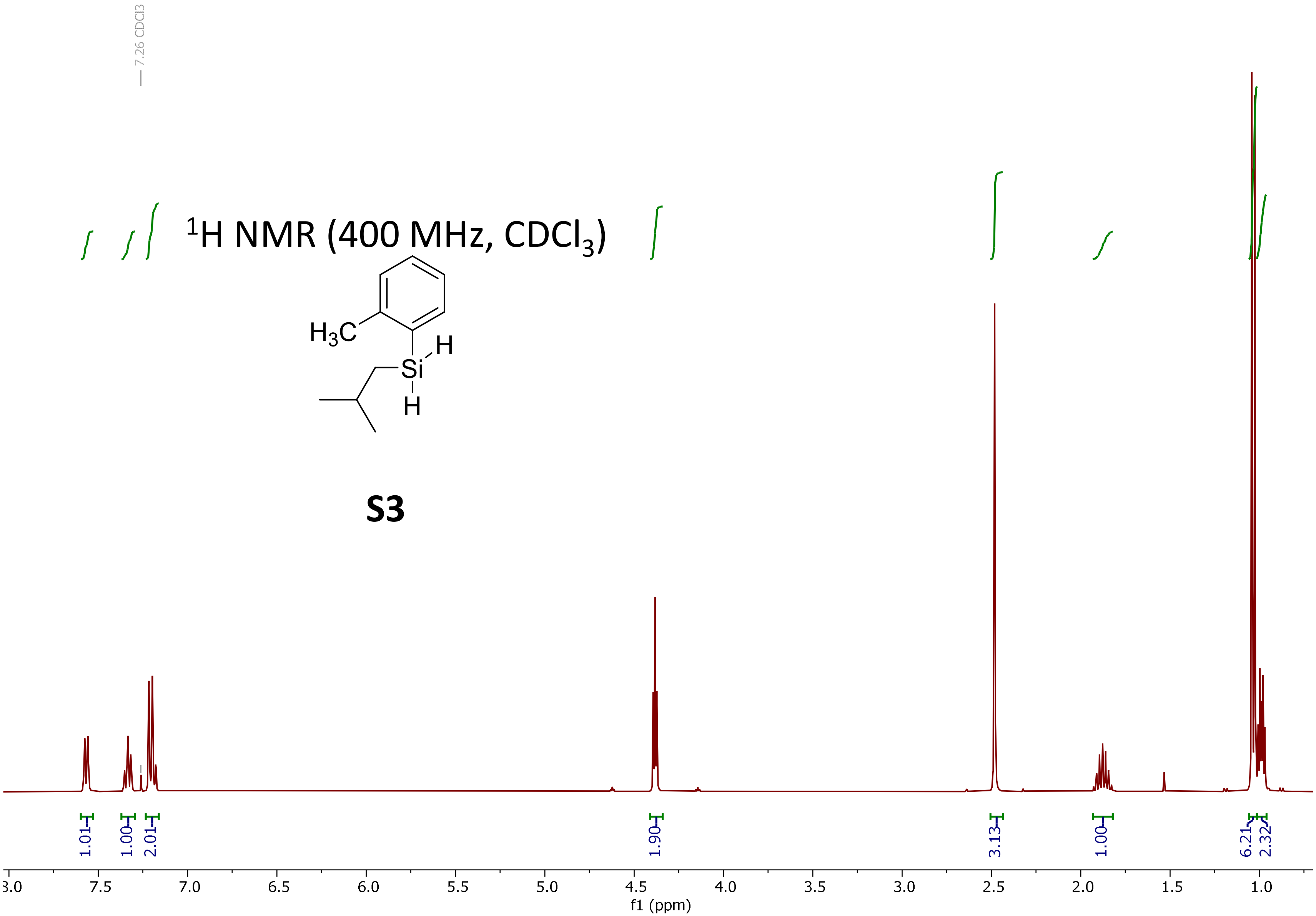

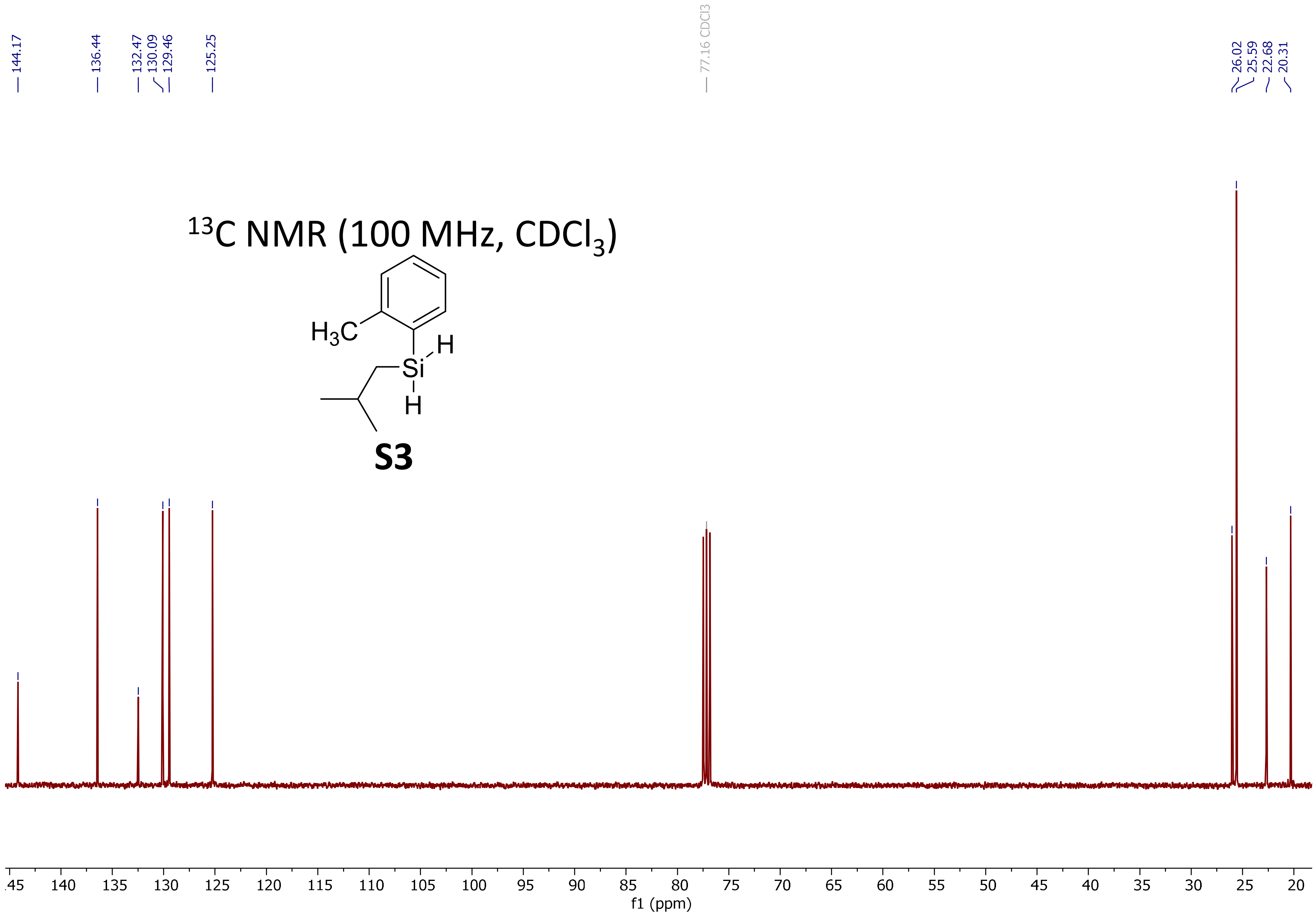

144.17
136.44
132.47
130.09
129.46
125.25
77.16 CDCl3
26.02
25.59
22.68
20.31
13C NMR (100 MHz, CDCl3)
H3C
Si
H
H
S3
f1 (ppm)

^{1}H NMR (400 MHz, C_6D_6)

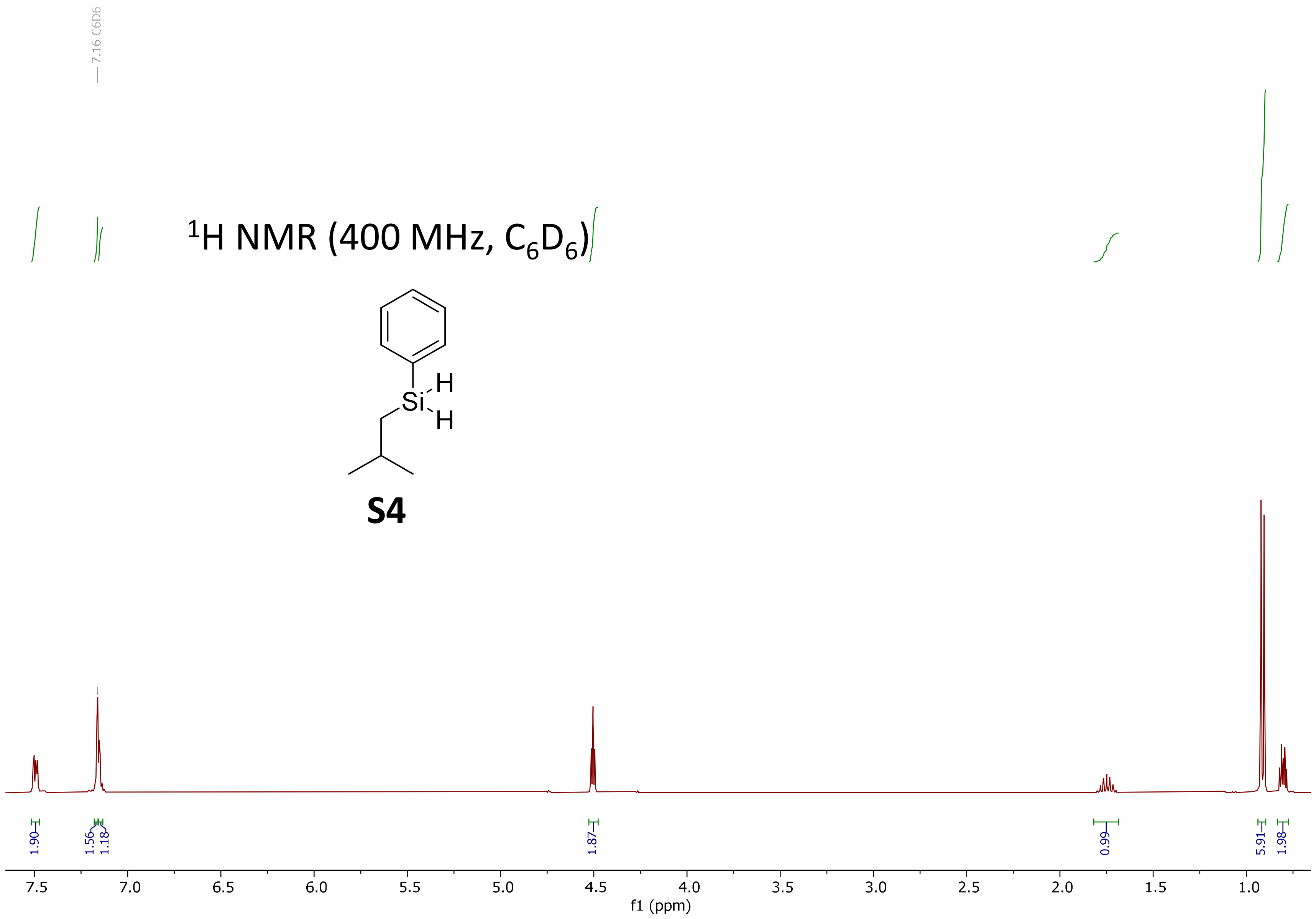

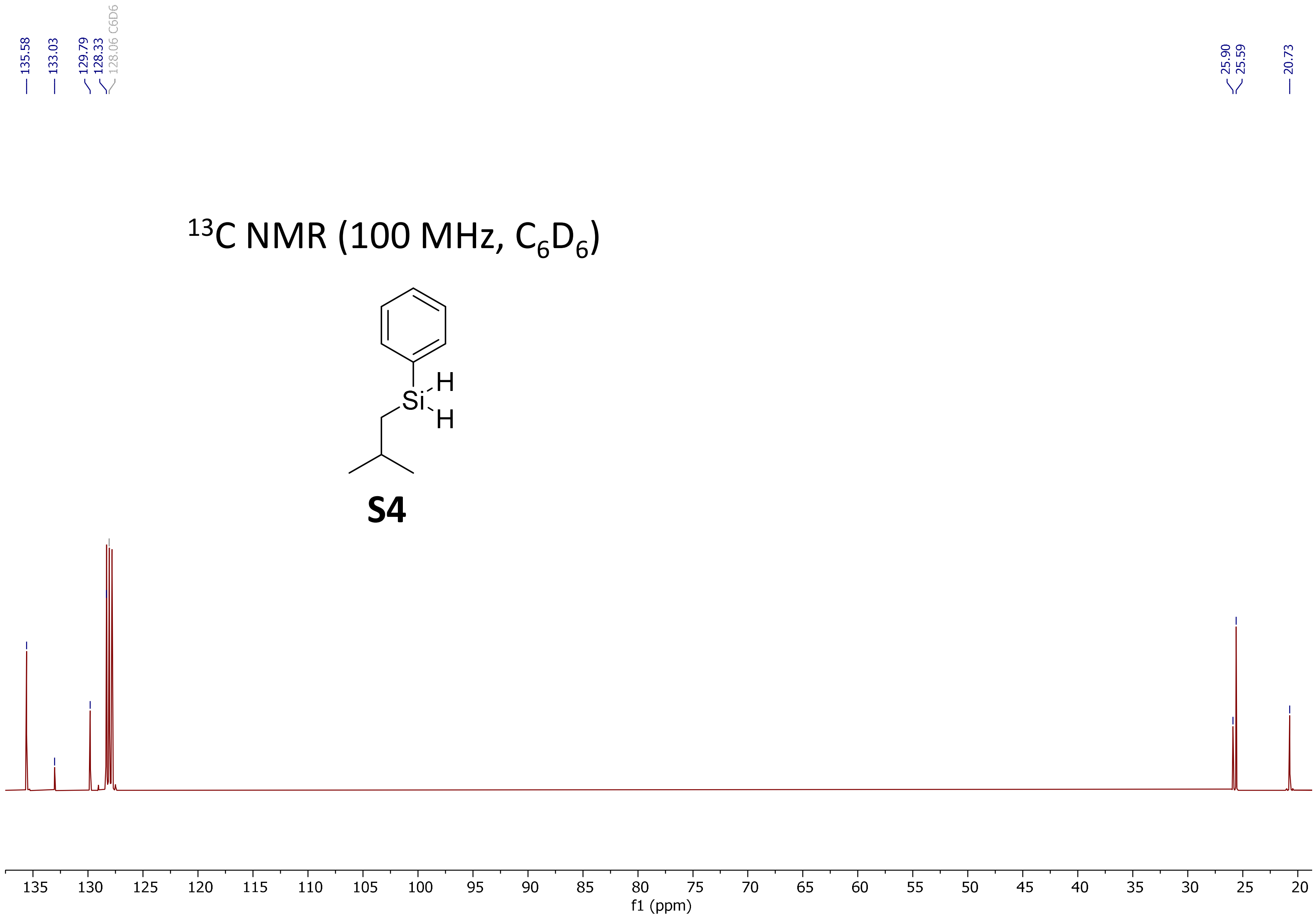
135.58
133.03
129.79
128.33
128.06 C6D6
25.90
25.59
20.73
^{13}C NMR (100 MHz, C_6D_6)
Si
H
H
S4
135
130
125
120
115
110
105
100
95
90
85
80
75
70
65
60
55
50
45
40
35
30
25
20
f1 (ppm)

^{1}H NMR (400 MHz, $CDCl_3$)

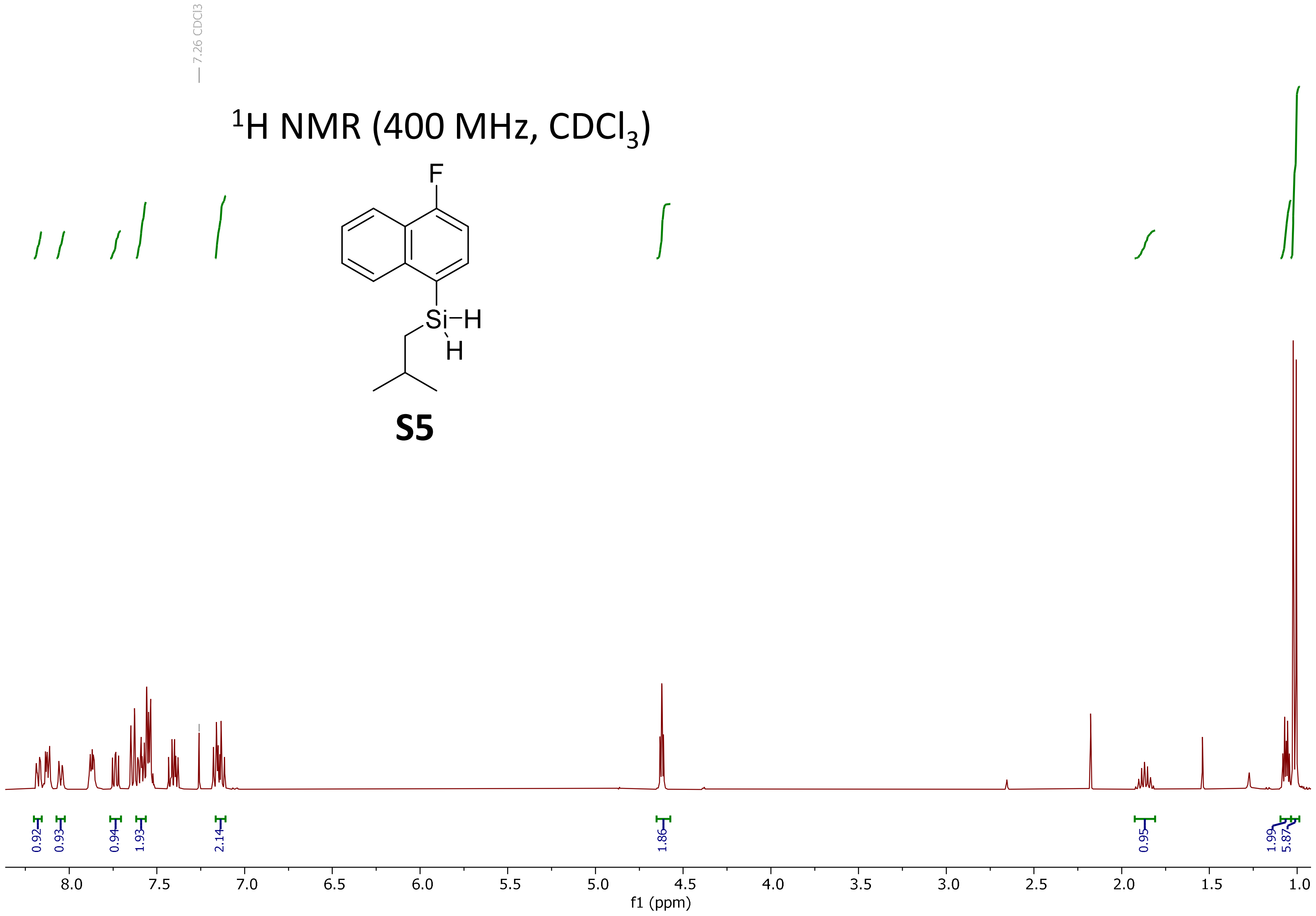

S5

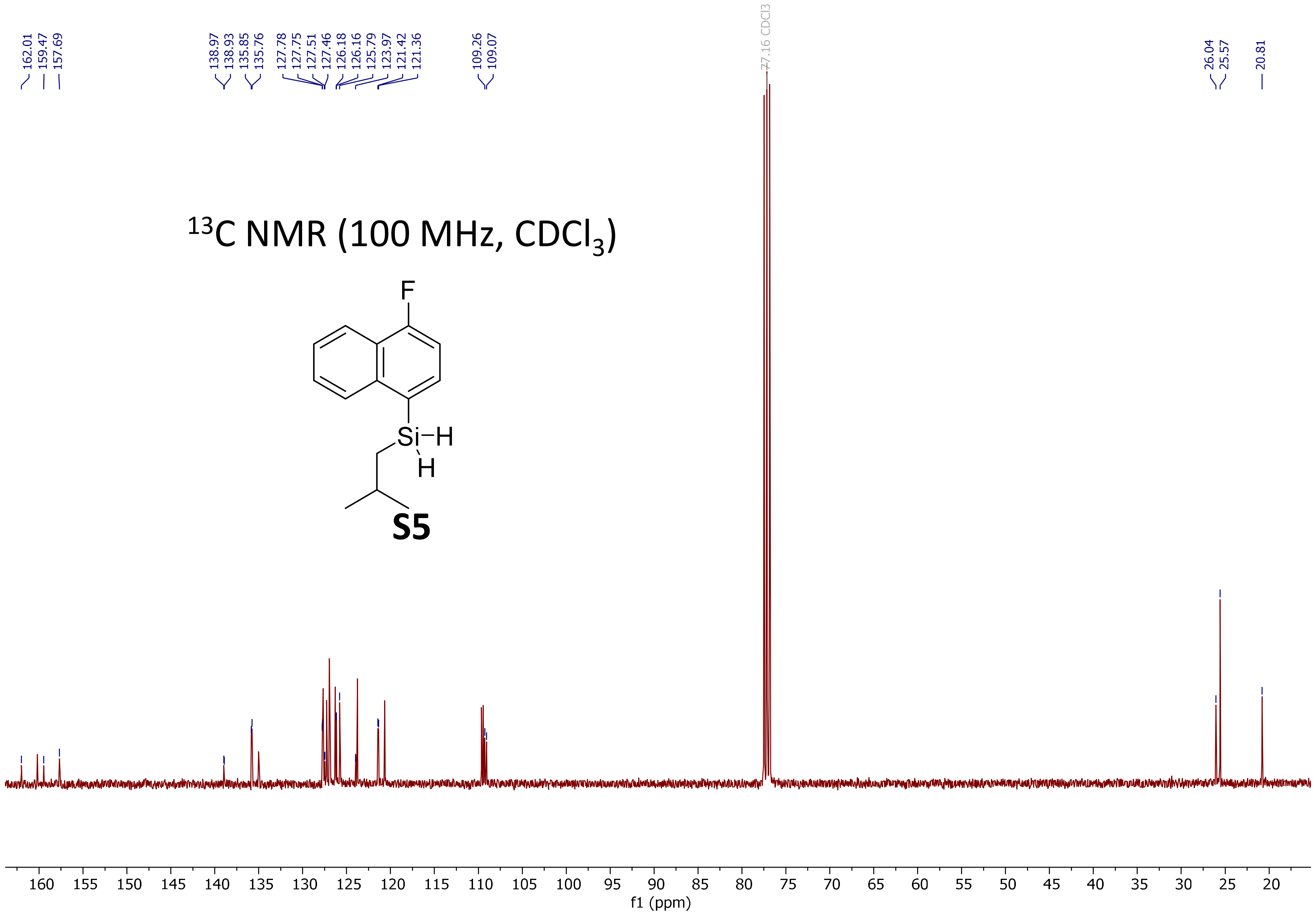

^{13}C NMR (100 MHz, CDCl$_3$)
162.01
159.47
157.69
138.97
138.93
135.85
135.76
127.78
127.75
127.51
127.46
126.18
126.16
125.79
123.97
121.42
121.36
109.26
109.07
77.16 CDCl3
26.04
25.57
20.81
F
Si–H
H
S5
160 155 150 145 140 135 130 125 120 115 110 105 100 95 90 85 80 75 70 65 60 55 50 45 40 35 30 25 20
f1 (ppm)

Silanediols

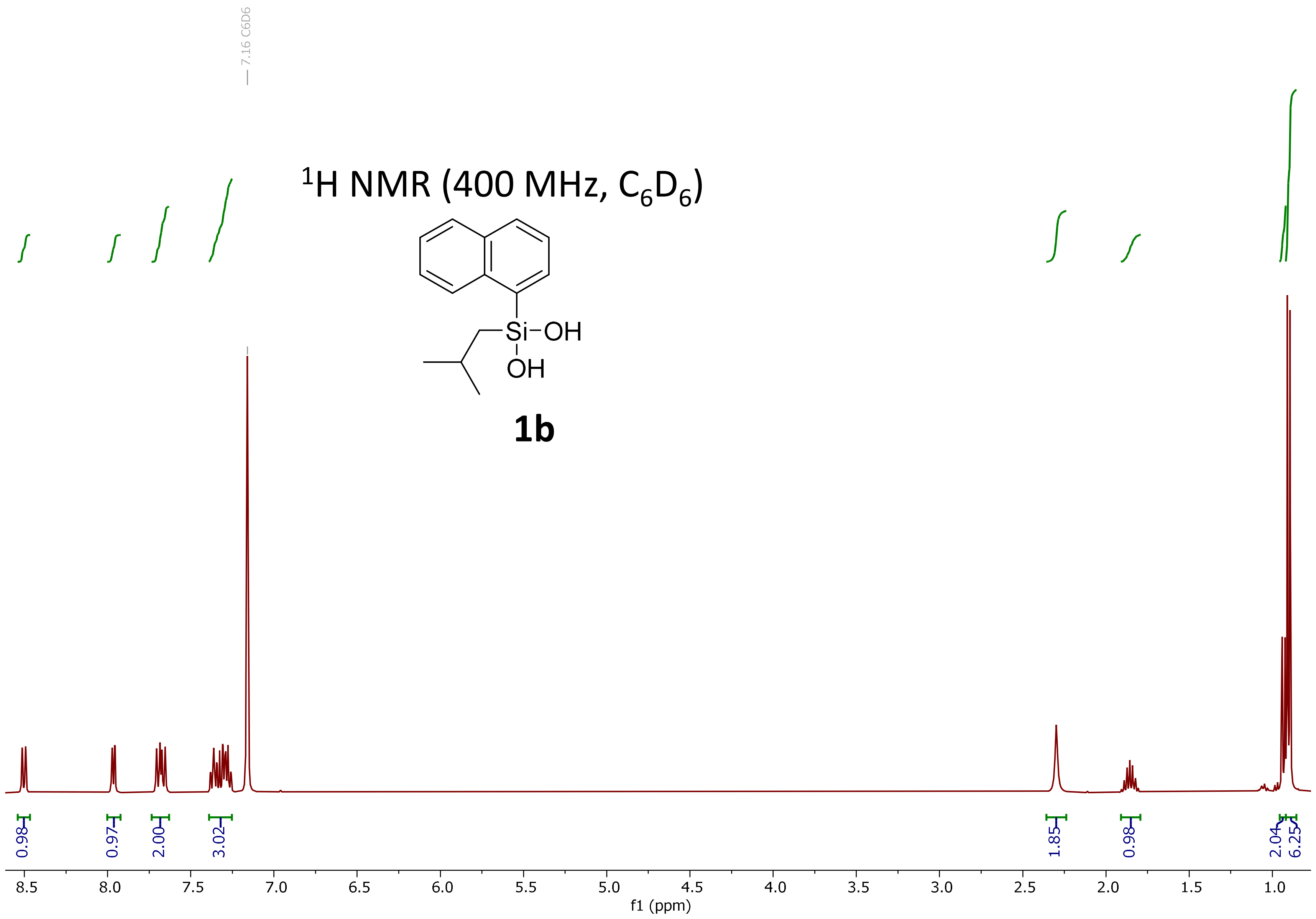
7.16 C6D6
^{1}H NMR (400 MHz, C_6D_6)
Si
OH
OH
1b
0.98
0.97
2.00
3.02
1.85
0.98
2.04
6.25
8.5
8.0
7.5
7.0
6.5
6.0
5.5
5.0
4.5
4.0
3.5
3.0
2.5
2.0
1.5
1.0
f1 (ppm)

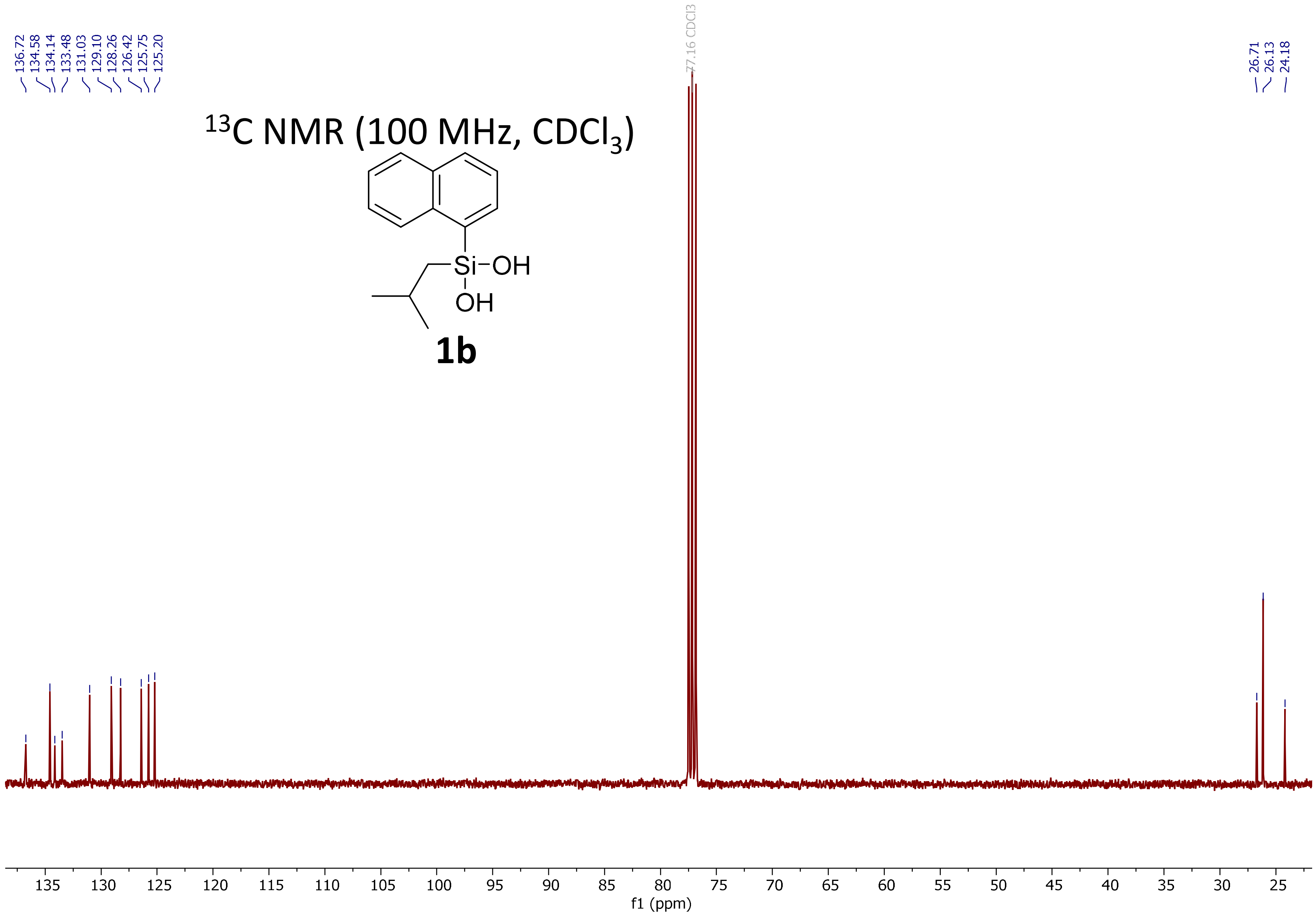

13C NMR (100 MHz, CDCl3)
1b
136.72
134.58
134.14
133.48
131.03
129.10
128.26
126.42
125.75
125.20
77.16 CDCl3
26.71
26.13
24.18
Si
OH
OH
135 130 125 120 115 110 105 100 95 90 85 80 75 70 65 60 55 50 45 40 35 30 25
f1 (ppm)

^{1}H NMR (400 MHz, $CDCl_3$)

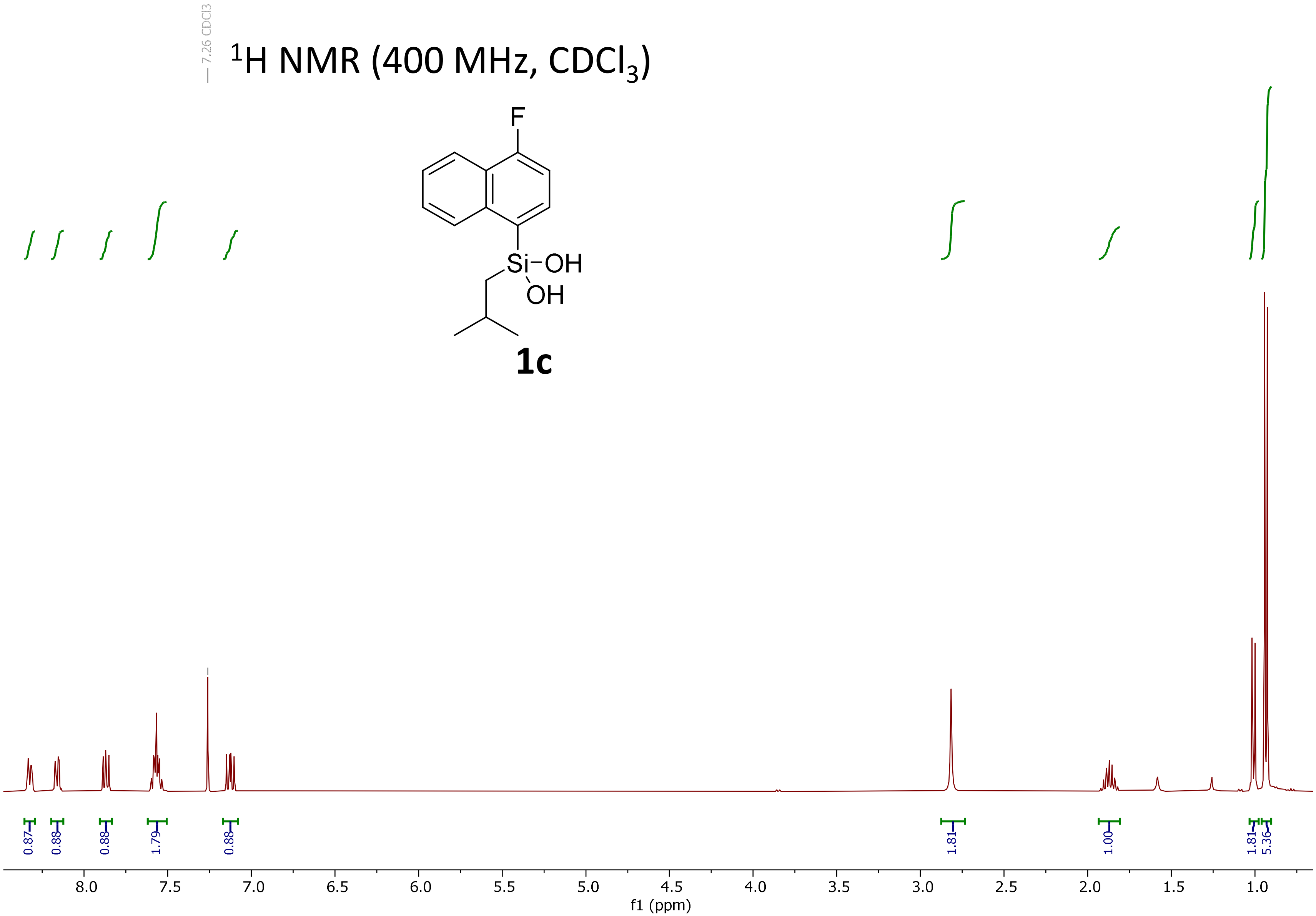

^{13}C NMR (100 MHz, $CDCl_3$)

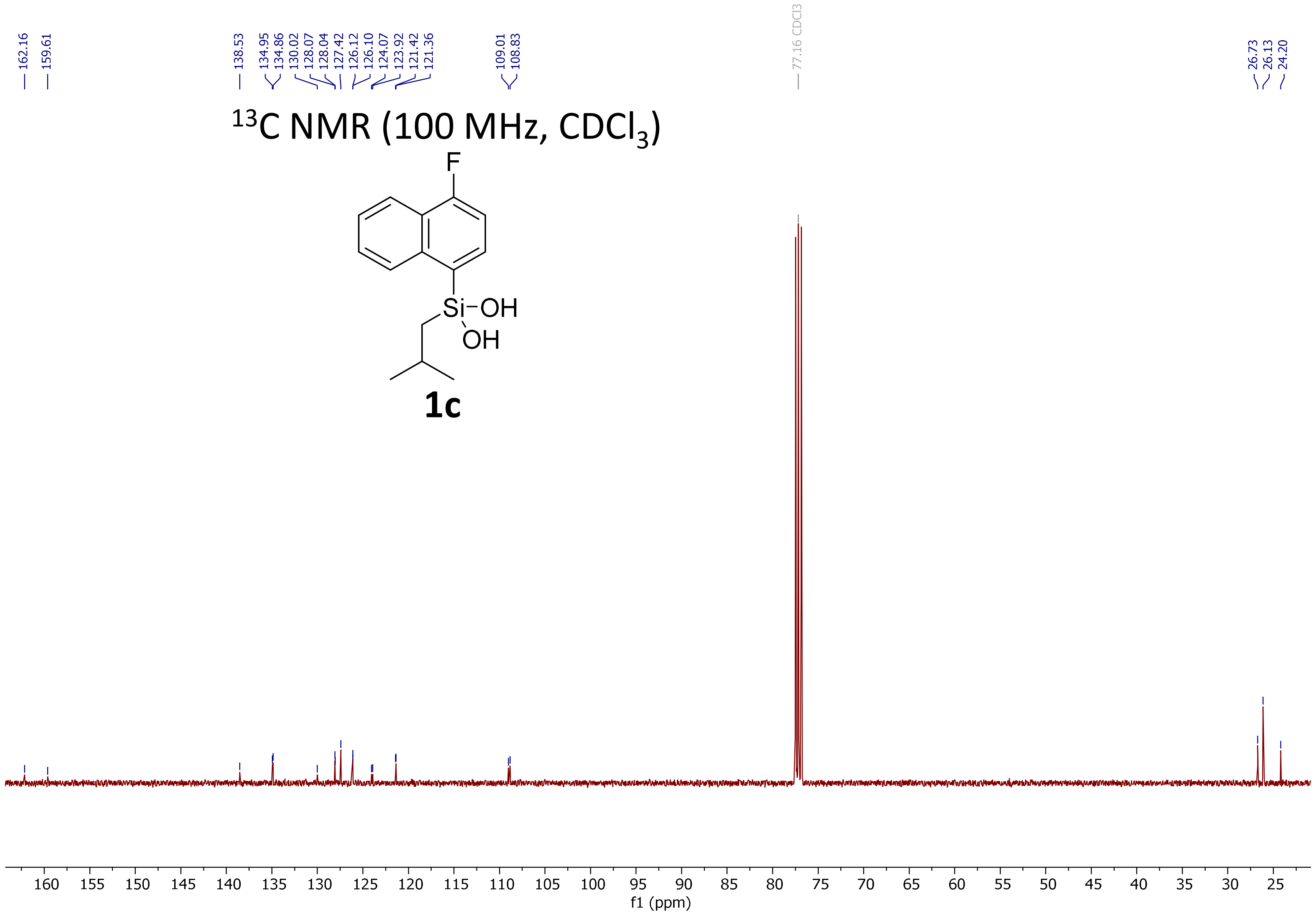

^1H NMR (400 MHz, C_6D_6)

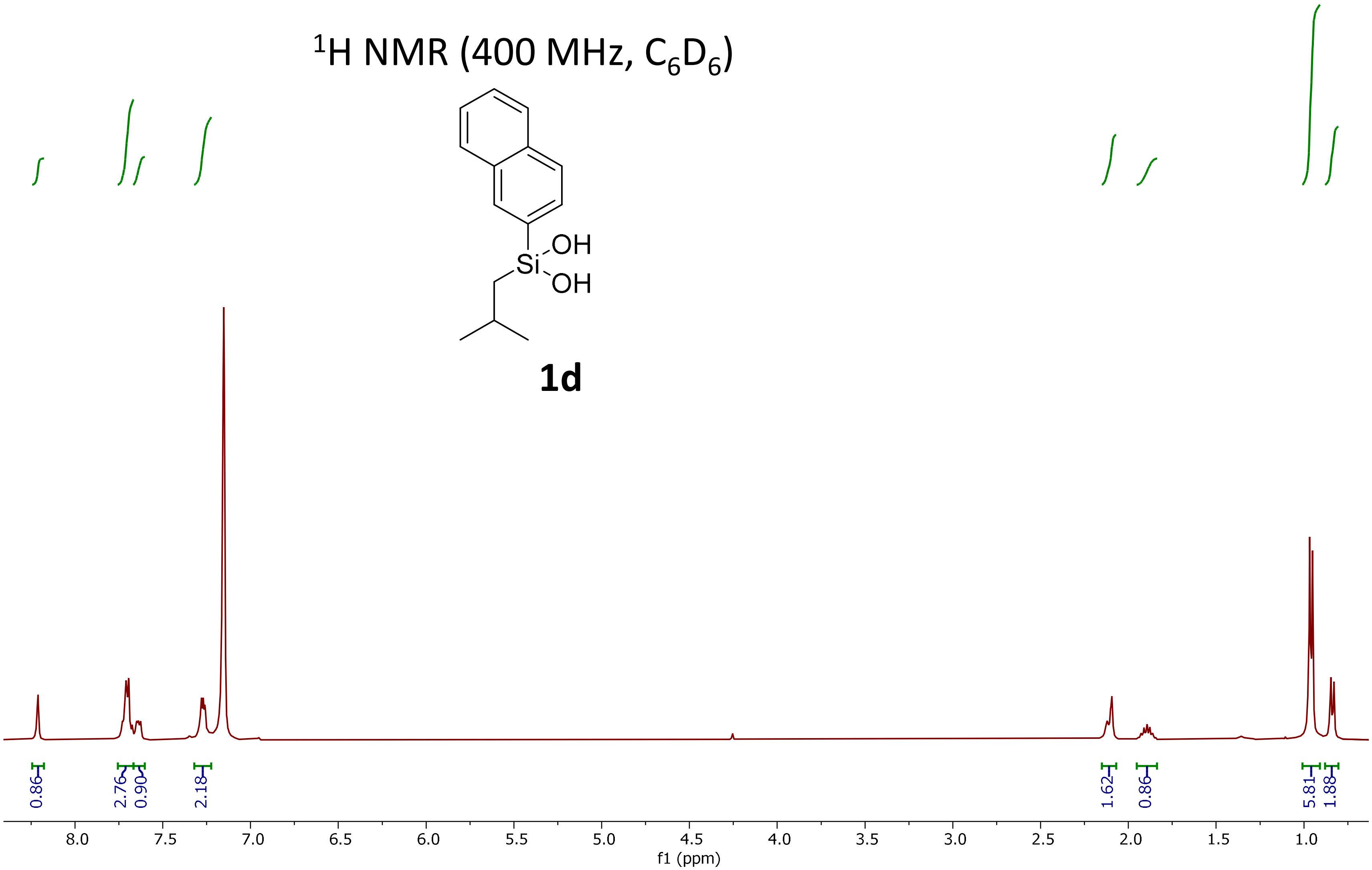

^{13}C NMR (100 MHz, C_6D_6)

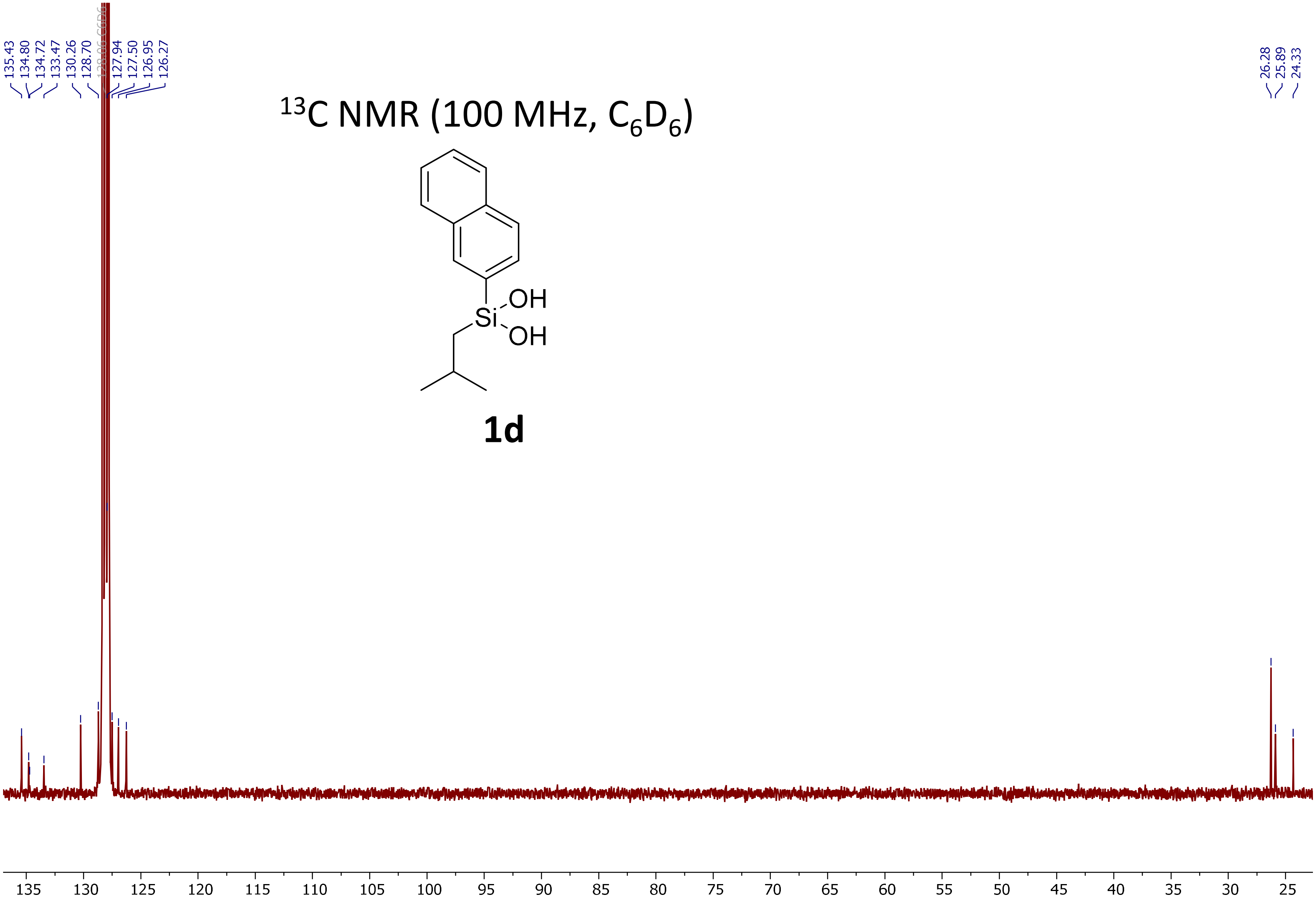

1d

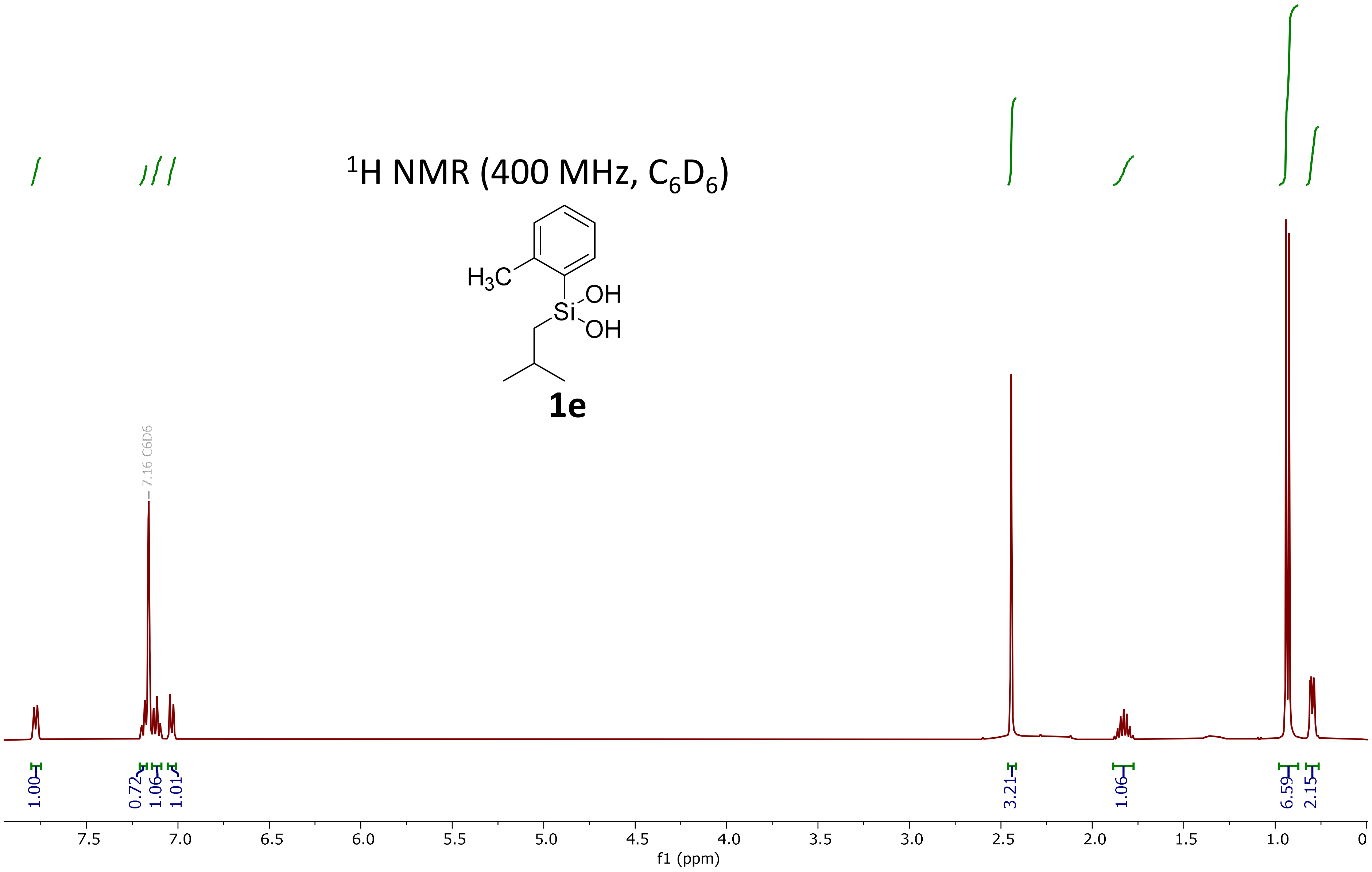

^{1}H NMR (400 MHz, C_6D_6)
H_3C
Si
OH
OH
1e
7.16 C6D6
1.00
0.72
1.06
1.01
3.21
1.06
6.59
2.15
7.5
7.0
6.5
6.0
5.5
5.0
4.5
4.0
3.5
3.0
2.5
2.0
1.5
1.0
0
f1 (ppm)

^{13}C NMR (100 MHz, C_6D_6)

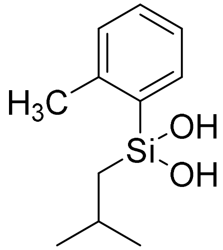

1e

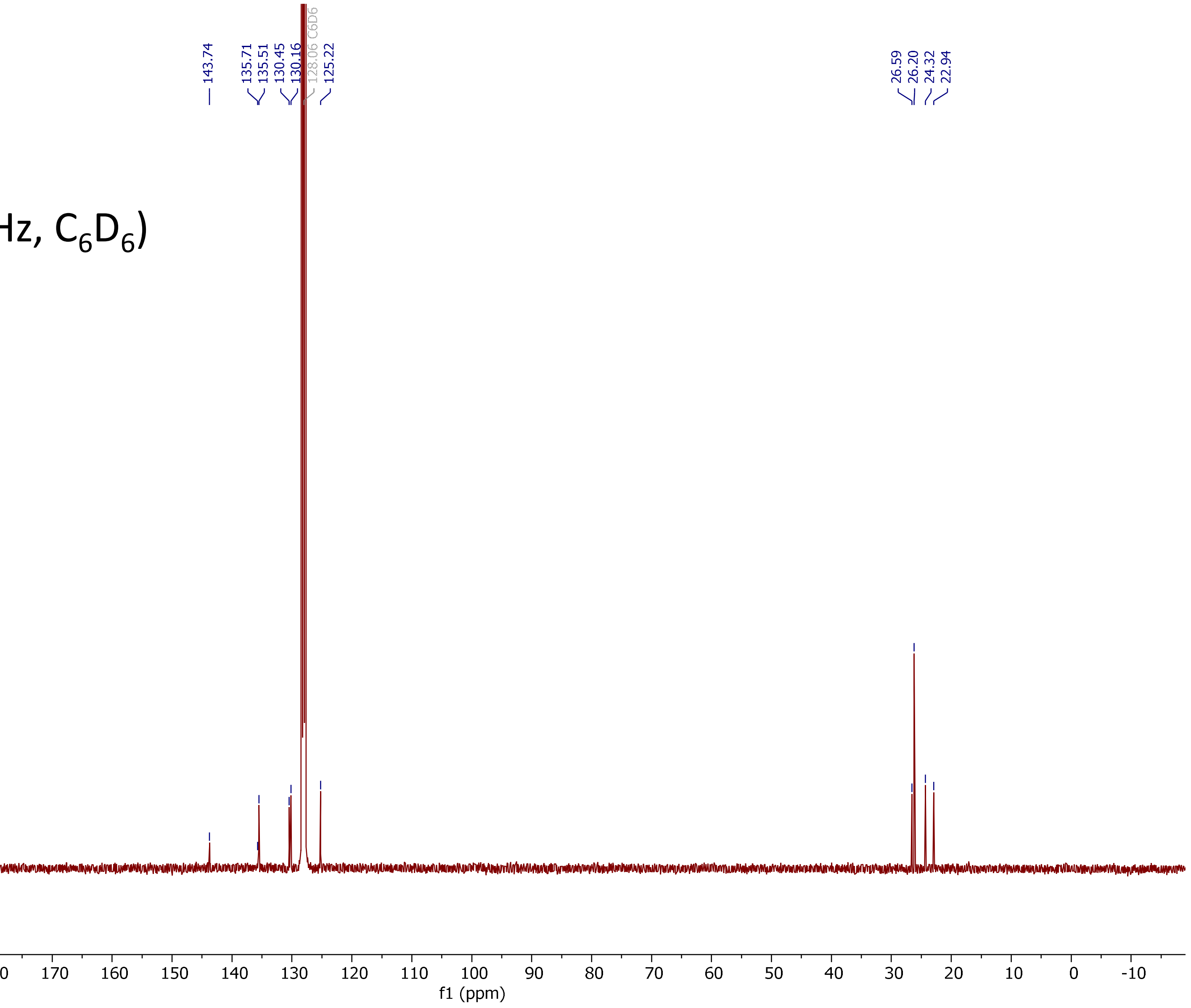

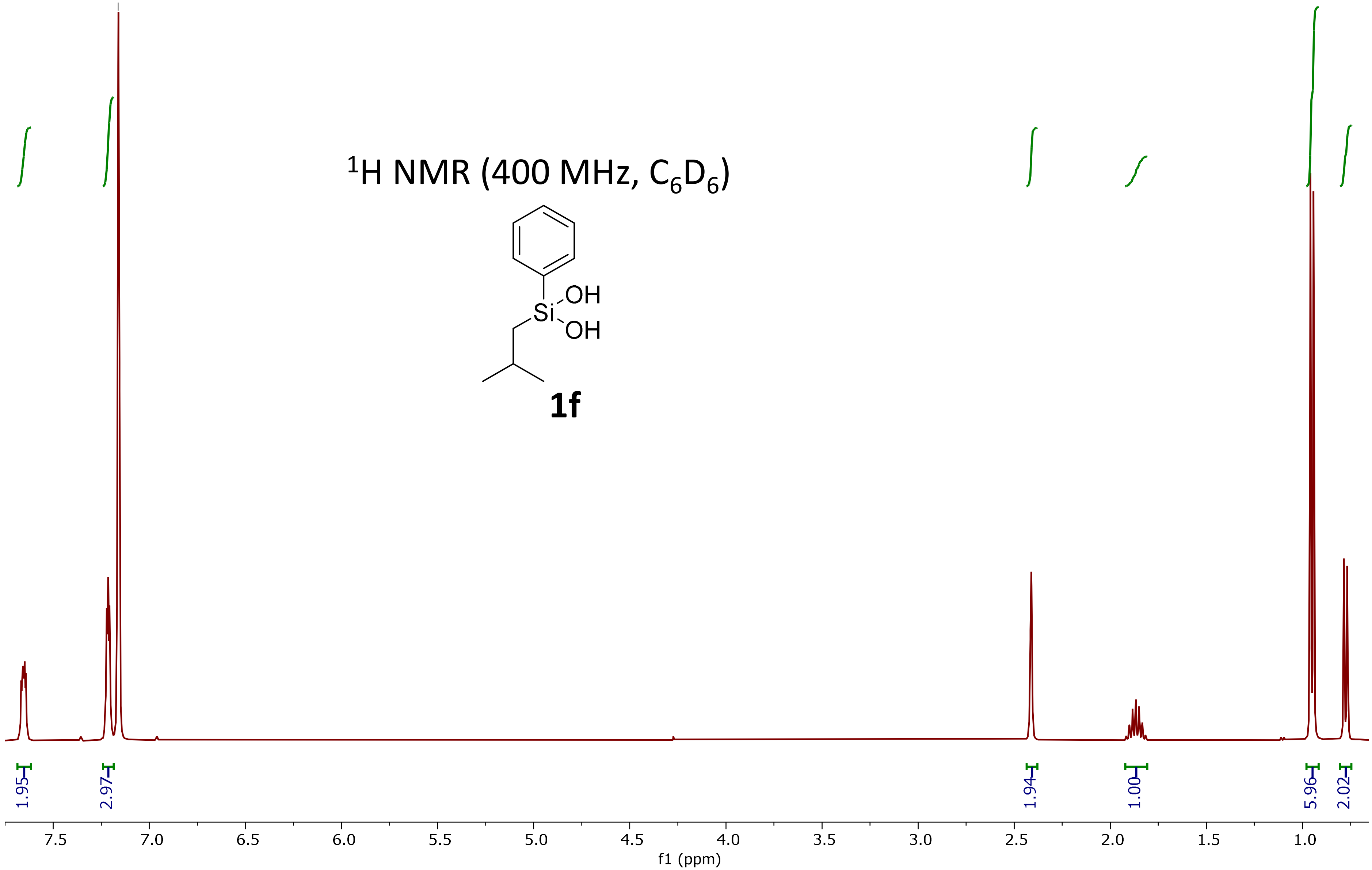

^{1}H NMR (400 MHz, C_6D_6)
OH
Si
OH
1f
1.95
2.97
1.94
1.00
5.96
2.02
7.5
7.0
6.5
6.0
5.5
5.0
4.5
4.0
3.5
3.0
2.5
2.0
1.5
1.0
f1 (ppm)

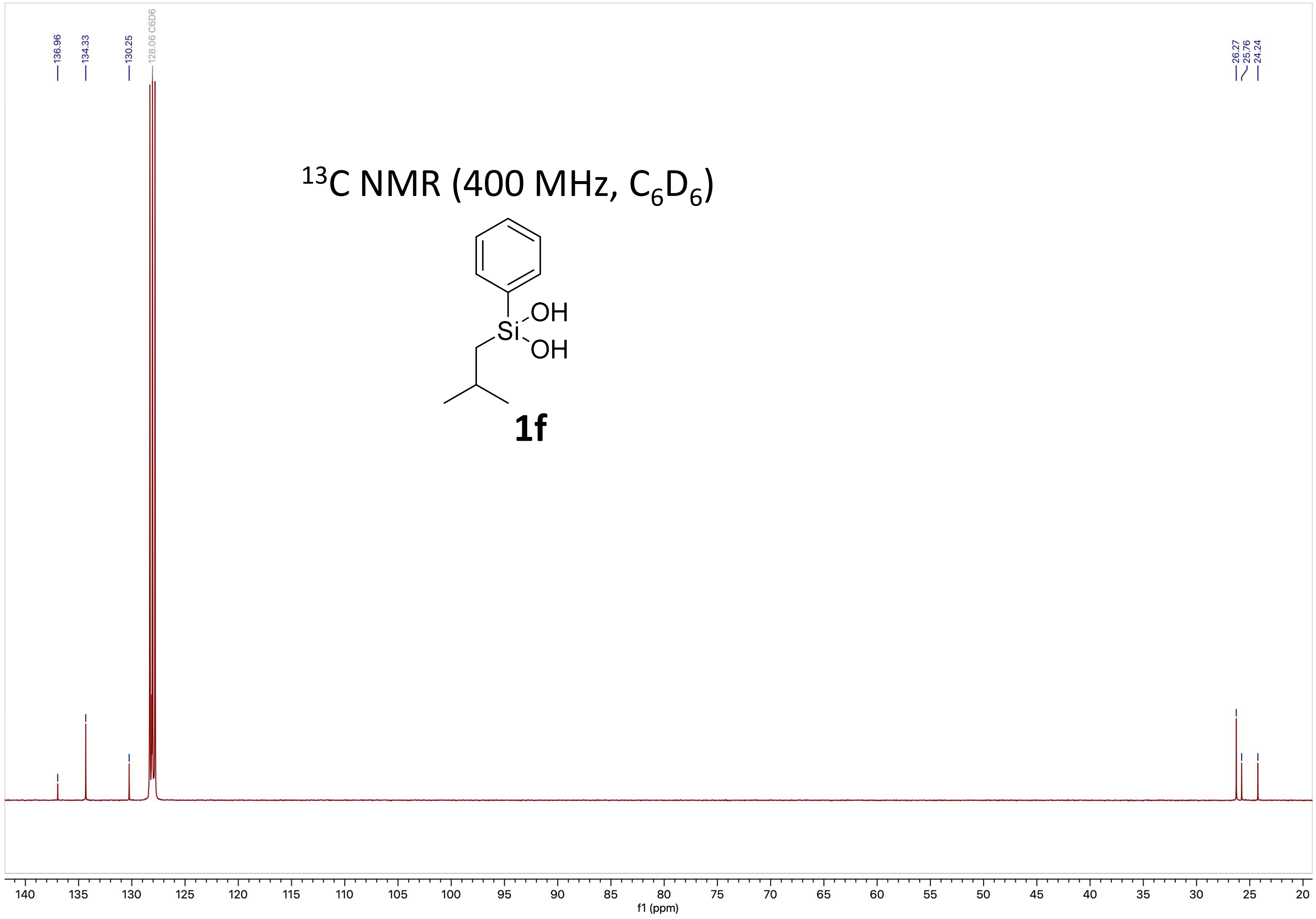

^{13}C NMR (400 MHz, C_6D_6)
1f
OH
Si
OH
136.96
134.33
130.25
128.06 C6D6
26.27
25.76
24.24
140 135 130 125 120 115 110 105 100 95 90 85 80 75 70 65 60 55 50 45 40 35 30 25 20
f1 (ppm)

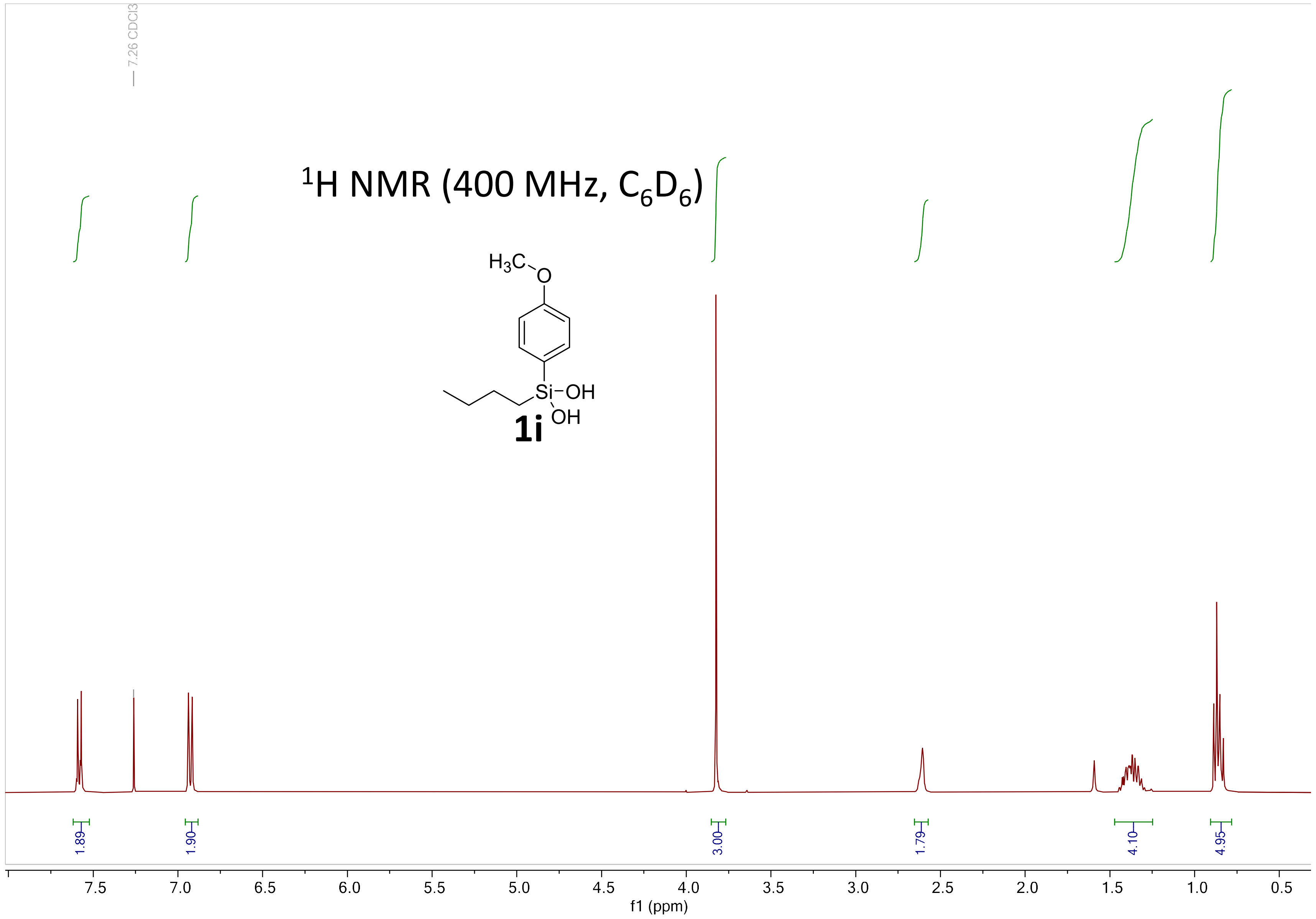

7.26 CDCl3
^{1}H NMR (400 MHz, C_6D_6)
H_3C
O
Si
OH
OH
1i
1.89
1.90
3.00
1.79
4.10
4.95
7.5
7.0
6.5
6.0
5.5
5.0
4.5
4.0
3.5
3.0
2.5
2.0
1.5
1.0
0.5
f1 (ppm)

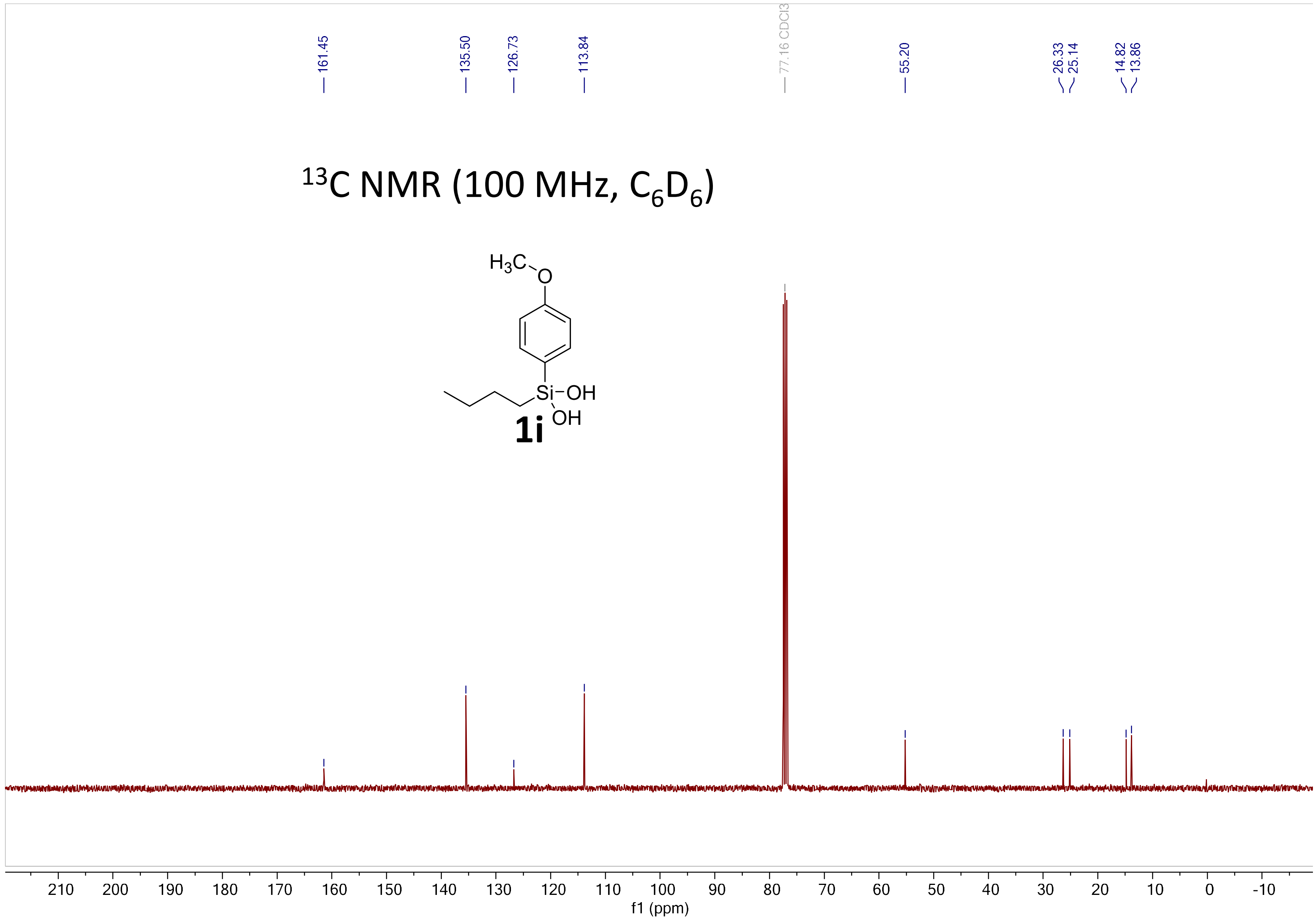

161.45
135.50
126.73
113.84
77.16 CDCl3
55.20
26.33
25.14
14.82
13.86
^{13}C NMR (100 MHz, C_6D_6)
H_3C
O
Si
OH
OH
1i
210
200
190
180
170
160
150
140
130
120
110
100
90
80
70
60
50
40
30
20
10
0
-10
f1 (ppm)

Siloxanols

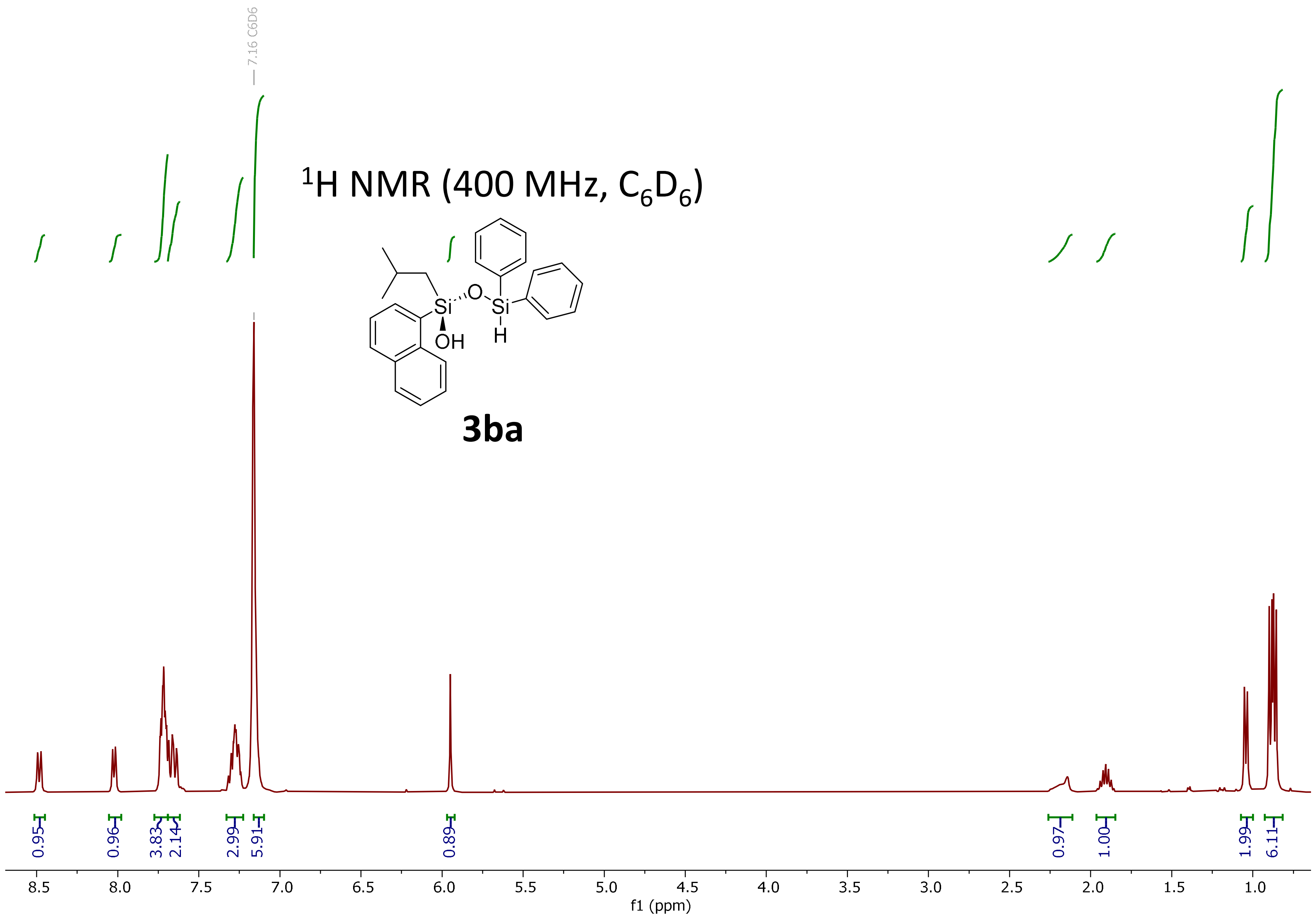

— 7.16 C6D6
¹H NMR (400 MHz, C6D6)
Si
O
Si
OH
H
3ba
0.95
0.96
3.83
2.14
2.99
5.91
0.89
0.97
1.00
1.99
6.11
8.5
8.0
7.5
7.0
6.5
6.0
5.5
5.0
4.5
4.0
3.5
3.0
2.5
2.0
1.5
1.0
f1 (ppm)

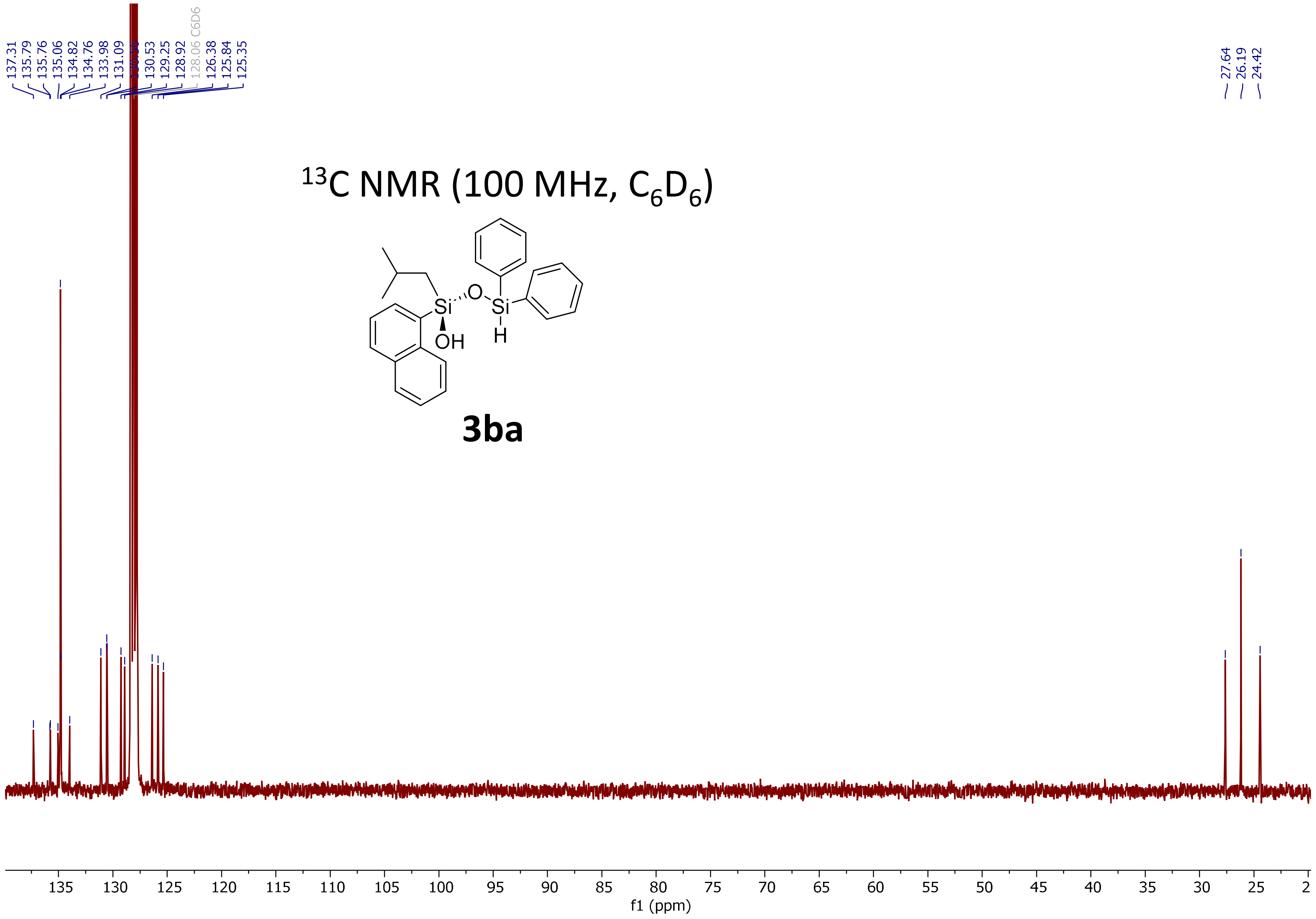

137.31
135.79
135.76
135.06
134.82
134.76
133.98
131.09
130.56
130.53
129.25
128.92
128.06 C6D6
126.38
125.84
125.35
27.64
26.19
24.42
^{13}C NMR (100 MHz, C_6D_6)
Si
O
Si
OH
H
3ba
135
130
125
120
115
110
105
100
95
90
85
80
75
70
65
60
55
50
45
40
35
30
25
2
f1 (ppm)

^{1}H NMR (400 MHz, C_6D_6)

3bb

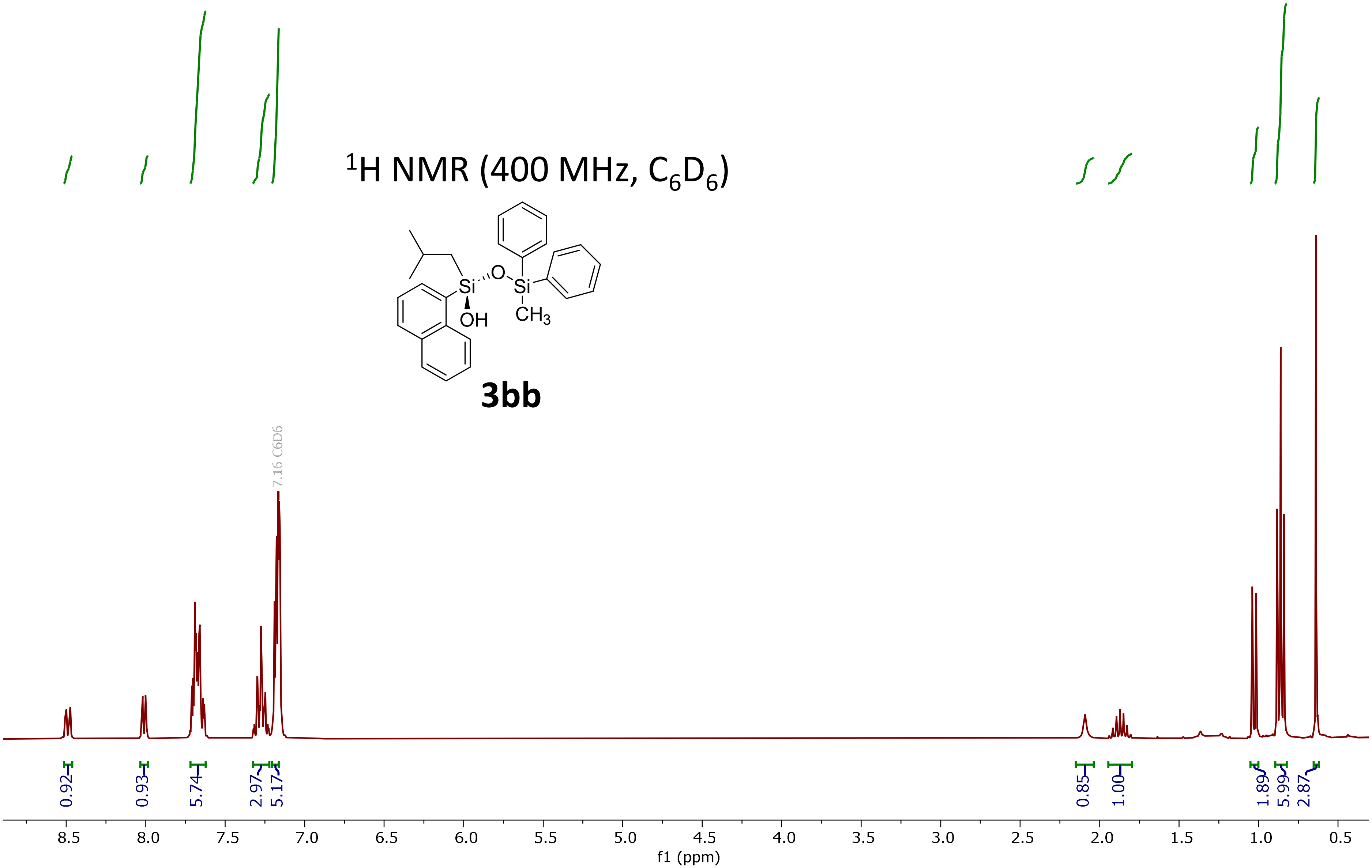

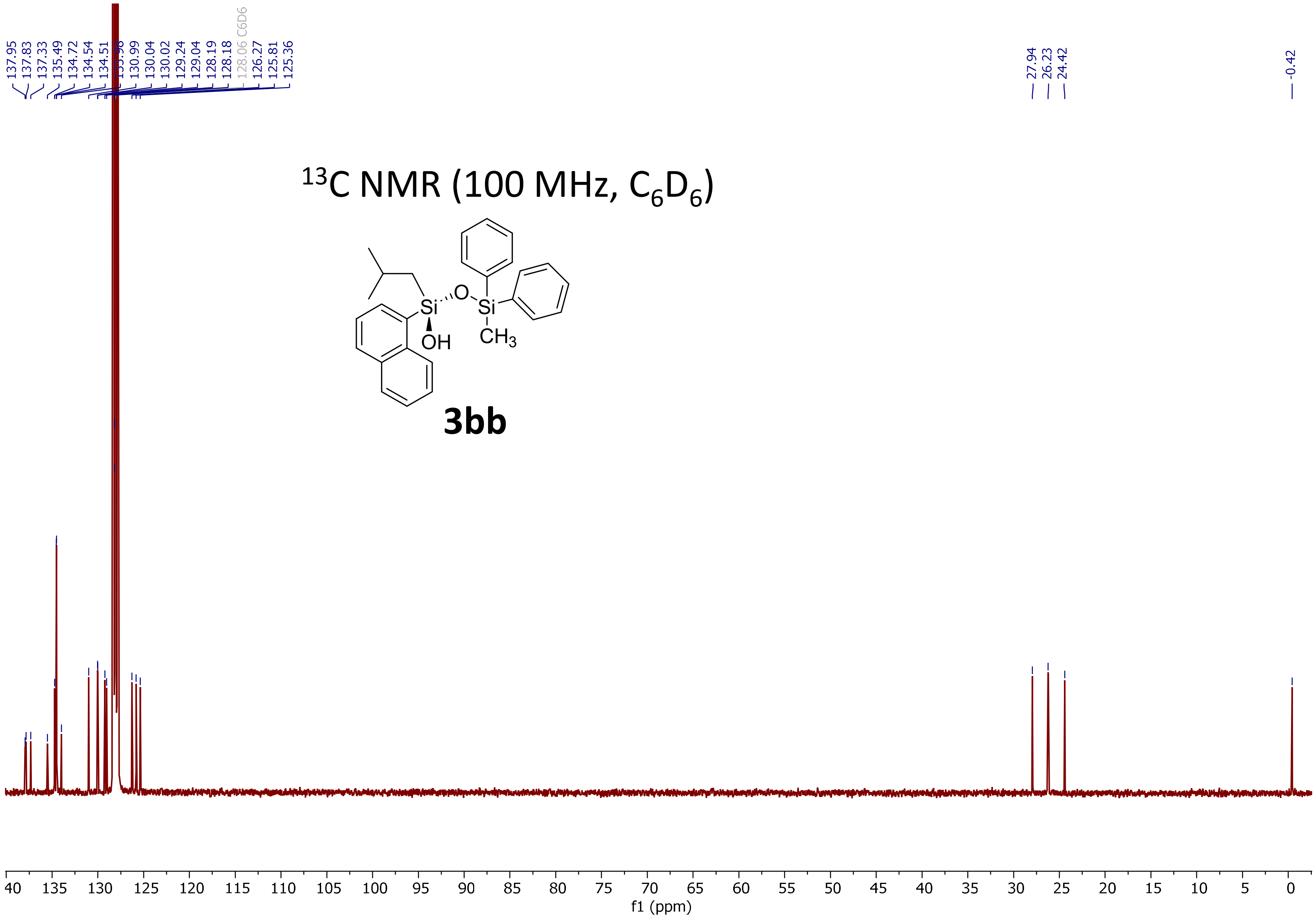

137.95
137.83
137.33
135.49
134.72
134.54
134.51
133.98
130.99
130.04
130.02
129.24
129.04
128.19
128.18
128.06 C6D6
126.27
125.81
125.36
27.94
26.23
24.42
-0.42
^{13}C NMR (100 MHz, C_6D_6)
Si
O
Si
OH
CH_3
3bb
140 135 130 125 120 115 110 105 100 95 90 85 80 75 70 65 60 55 50 45 40 35 30 25 20 15 10 5 0
f1 (ppm)

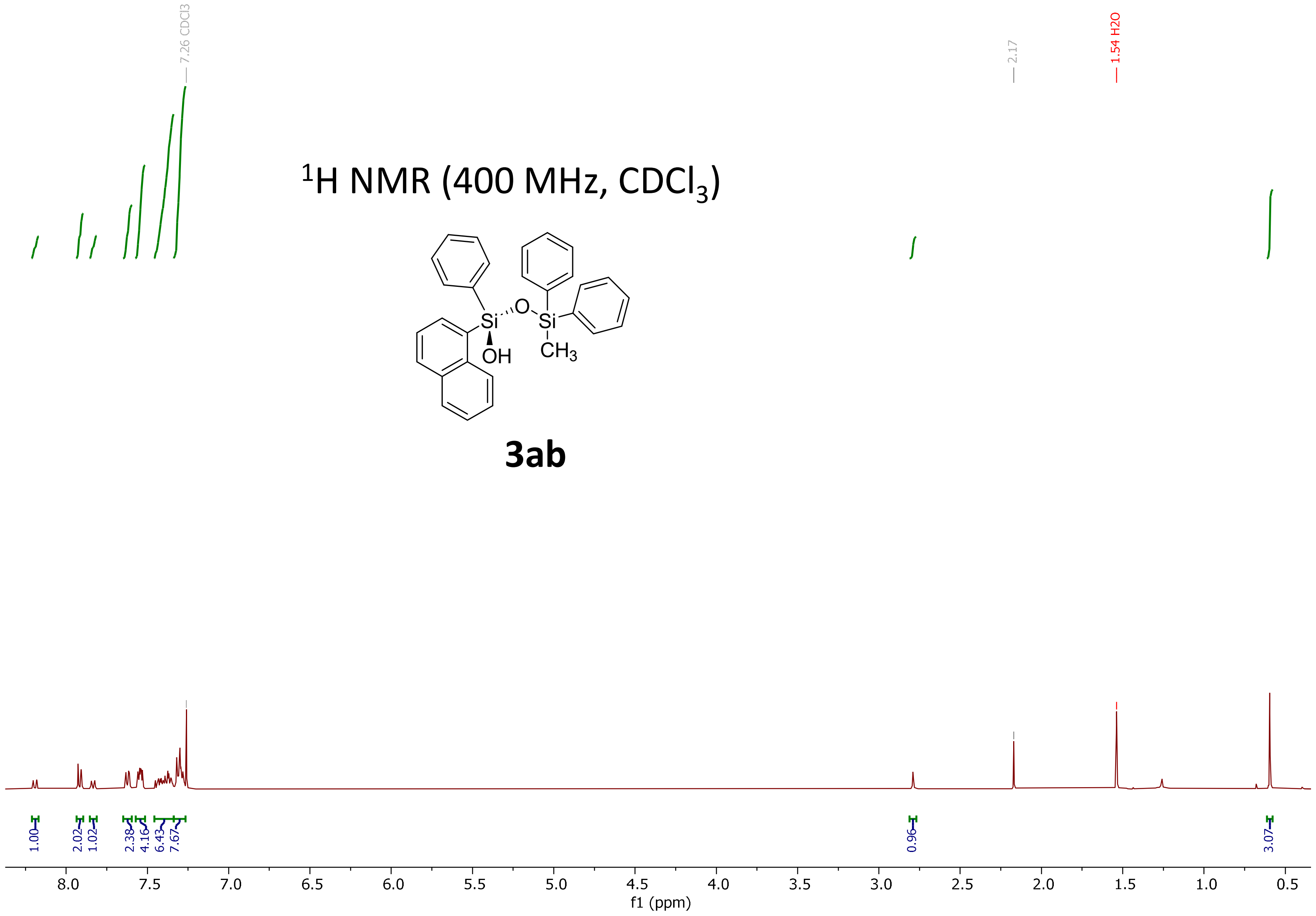

^{1}H NMR (400 MHz, $CDCl_3$)
7.26 CDCl3
2.17
1.54 H2O
Si
O
Si
OH
CH_3
1.00
2.02
1.02
2.38
4.16
6.43
7.67
0.96
3.07
8.0
7.5
7.0
6.5
6.0
5.5
5.0
4.5
4.0
3.5
3.0
2.5
2.0
1.5
1.0
0.5
f1 (ppm)

3ab

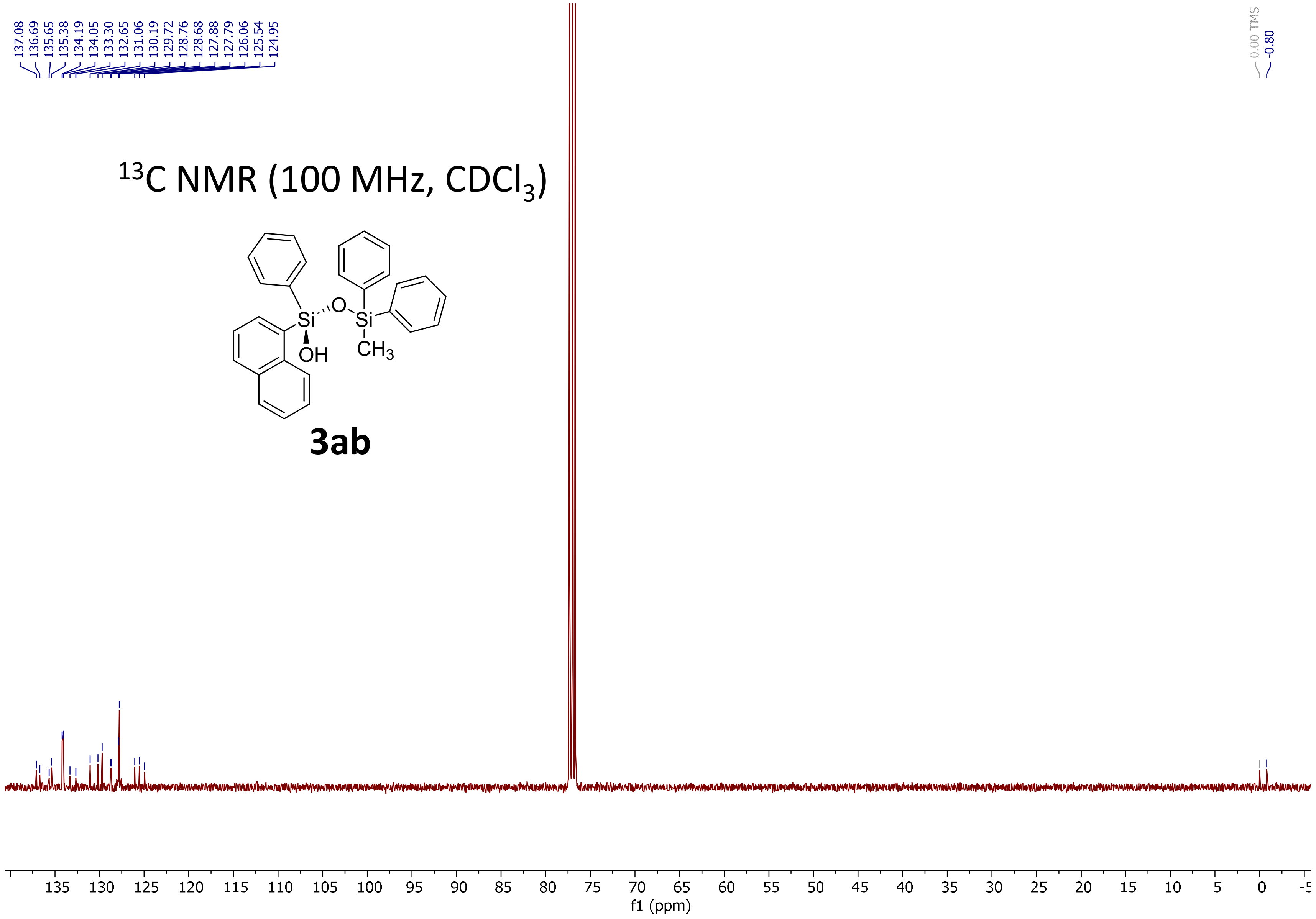

137.08
136.69
135.65
135.38
134.19
134.05
133.30
132.65
131.06
130.19
129.72
128.76
128.68
127.88
127.79
126.06
125.54
124.95
0.00 TMS
-0.80
^{13}C NMR (100 MHz, CDCl$_3$)
Si
O
Si
OH
CH$_3$
3ab
f1 (ppm)

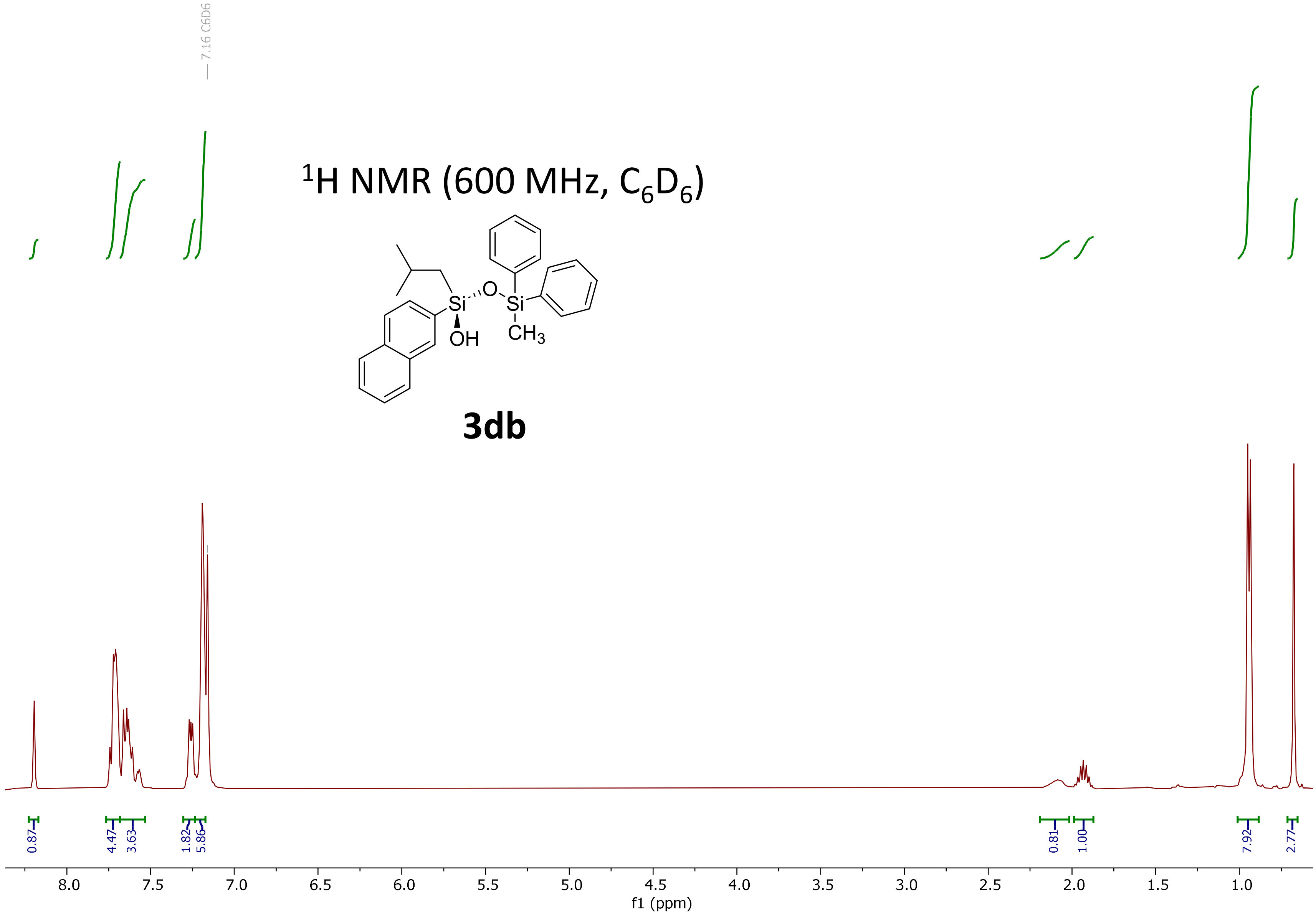

1H NMR (600 MHz, C6D6)
3db
OH
O
Si
Si
CH3
7.16 C6D6
0.87
4.47
3.63
1.82
5.86
0.81
1.00
7.92
2.77
8.0
7.5
7.0
6.5
6.0
5.5
5.0
4.5
4.0
3.5
3.0
2.5
2.0
1.5
1.0
f1 (ppm)

^{13}C NMR (100 MHz, C_6D_6)

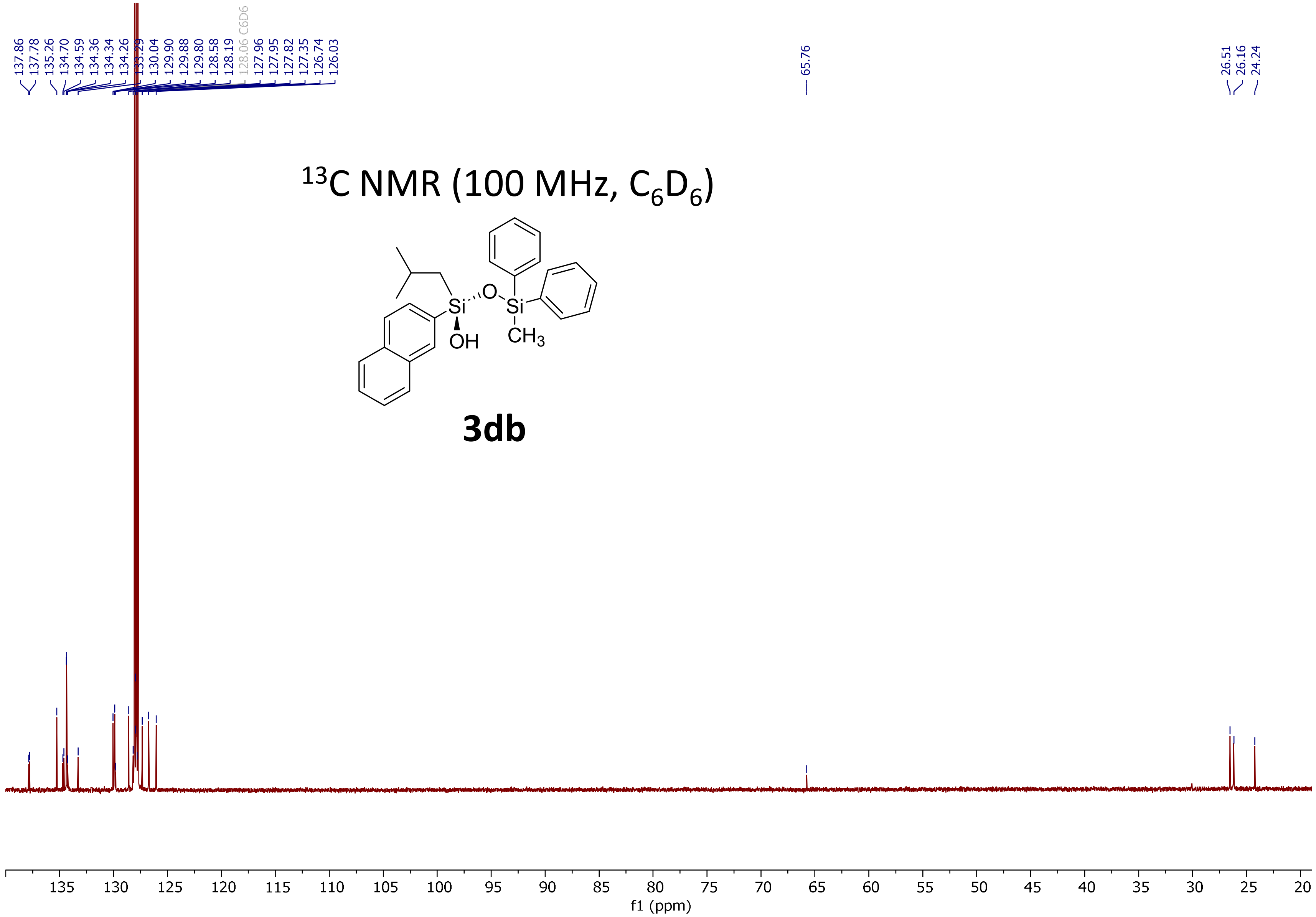

3db

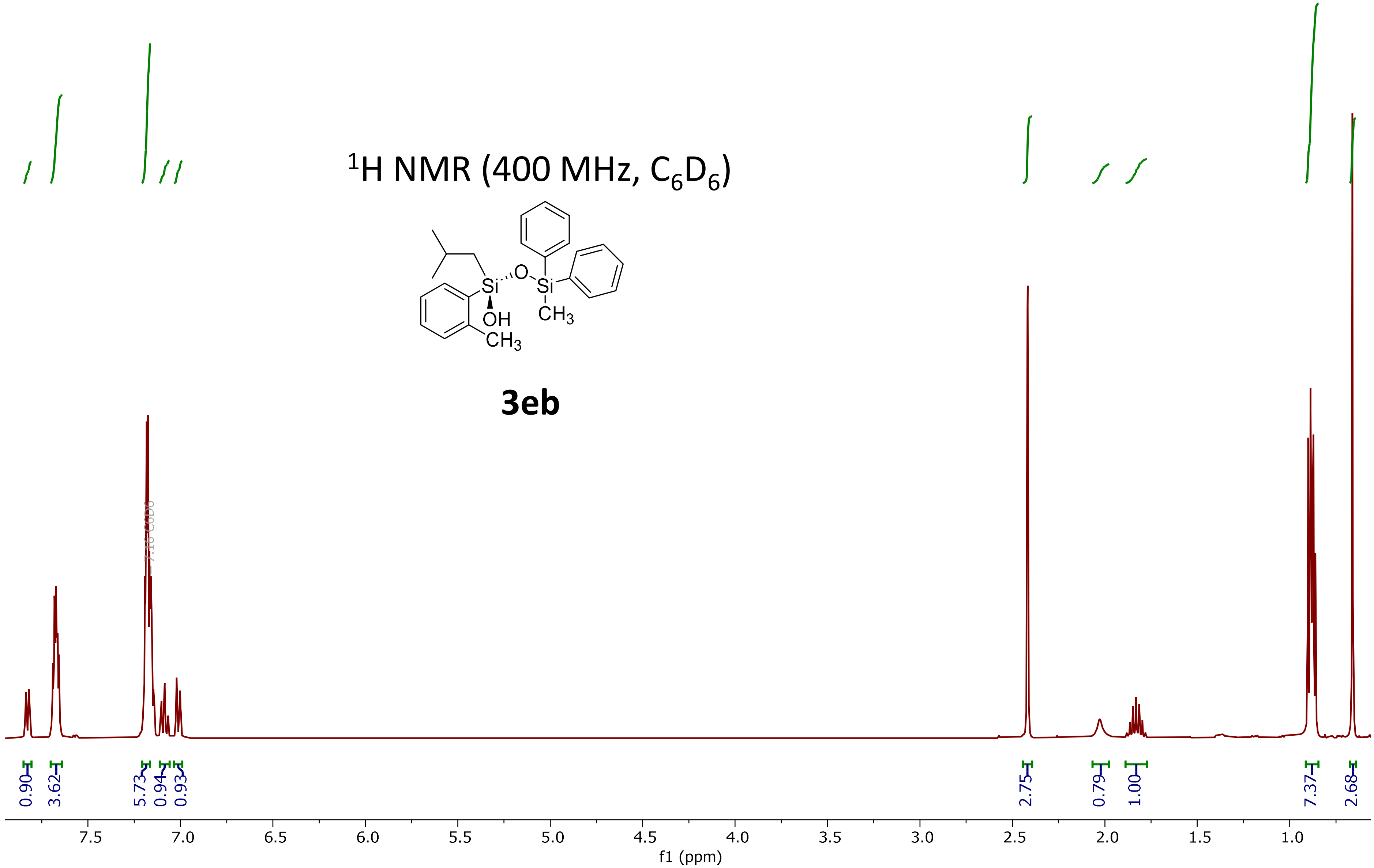

^1H NMR (400 MHz, C_6D_6)
OH
CH3
Si
O
Si
CH3
CH3
3eb
7.16 C6D6
0.90
3.62
5.73
0.94
0.93
2.75
0.79
1.00
7.37
2.68
7.5
7.0
6.5
6.0
5.5
5.0
4.5
4.0
3.5
3.0
2.5
2.0
1.5
1.0
f1 (ppm)

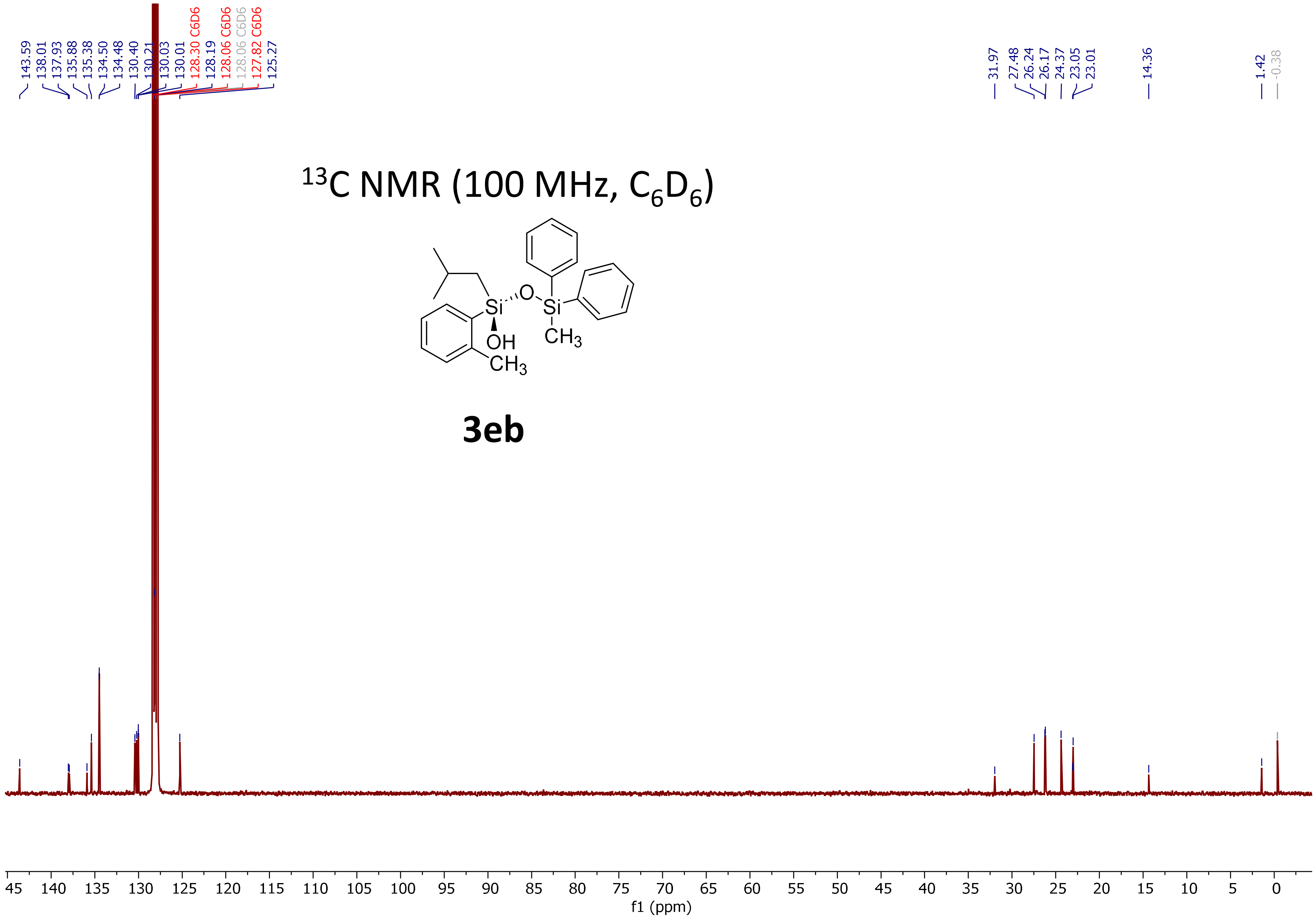

143.59
138.01
137.93
135.88
135.38
134.50
134.48
130.40
130.21
130.03
130.01
128.30 C6D6
128.19
128.06 C6D6
128.06 C6D6
127.82 C6D6
125.27
31.97
27.48
26.24
26.17
24.37
23.05
23.01
14.36
1.42
-0.38
^{13}C NMR (100 MHz, C_6D_6)
Si
O
Si
OH
CH_3
CH_3
3eb
f1 (ppm)

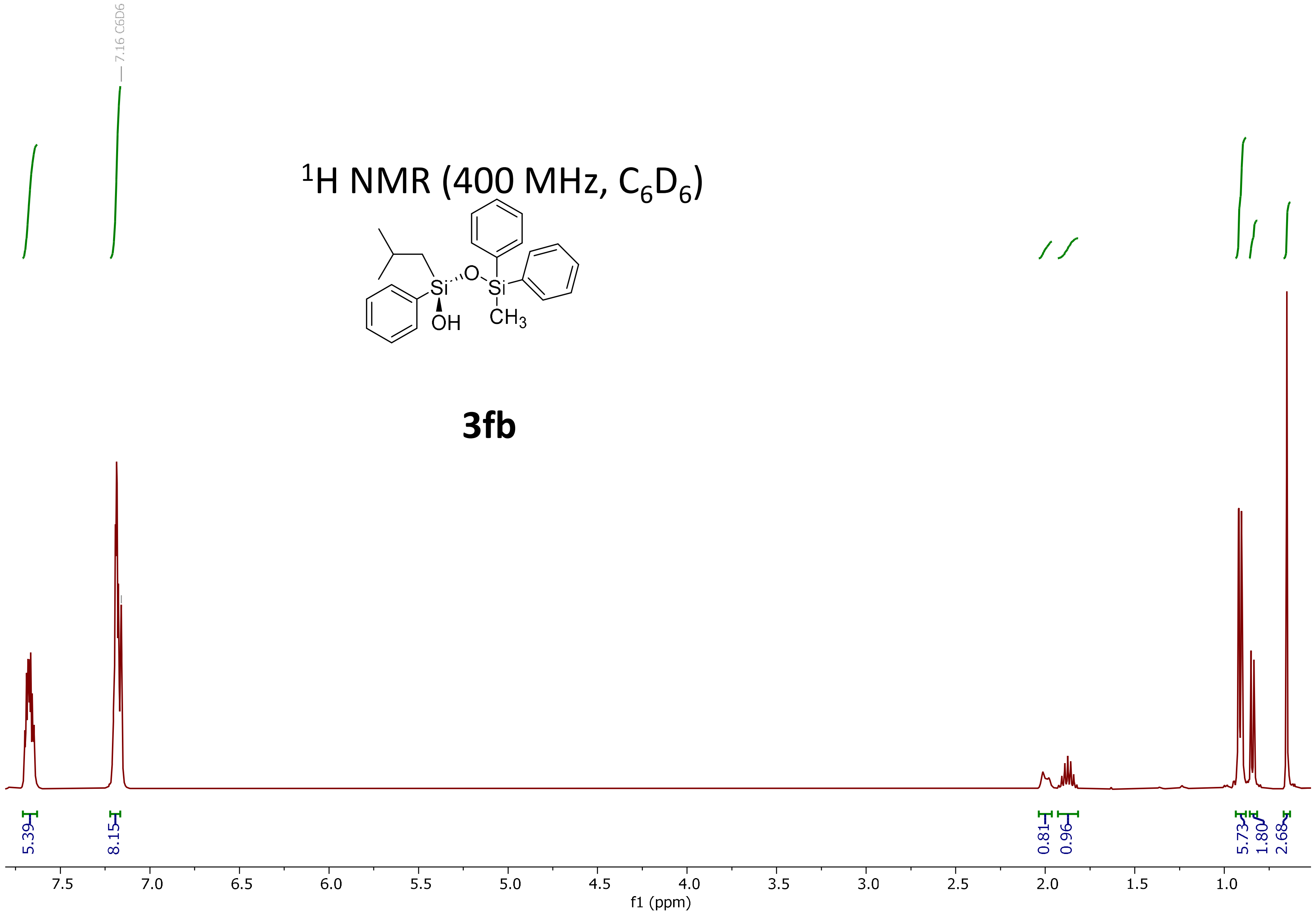

1H NMR (400 MHz, C6D6)
OH
CH3
O
Si
3fb
7.16 C6D6
5.39
8.15
0.81
0.96
5.73
1.80
2.68
7.5
7.0
6.5
6.0
5.5
5.0
4.5
4.0
3.5
3.0
2.5
2.0
1.5
1.0
f1 (ppm)

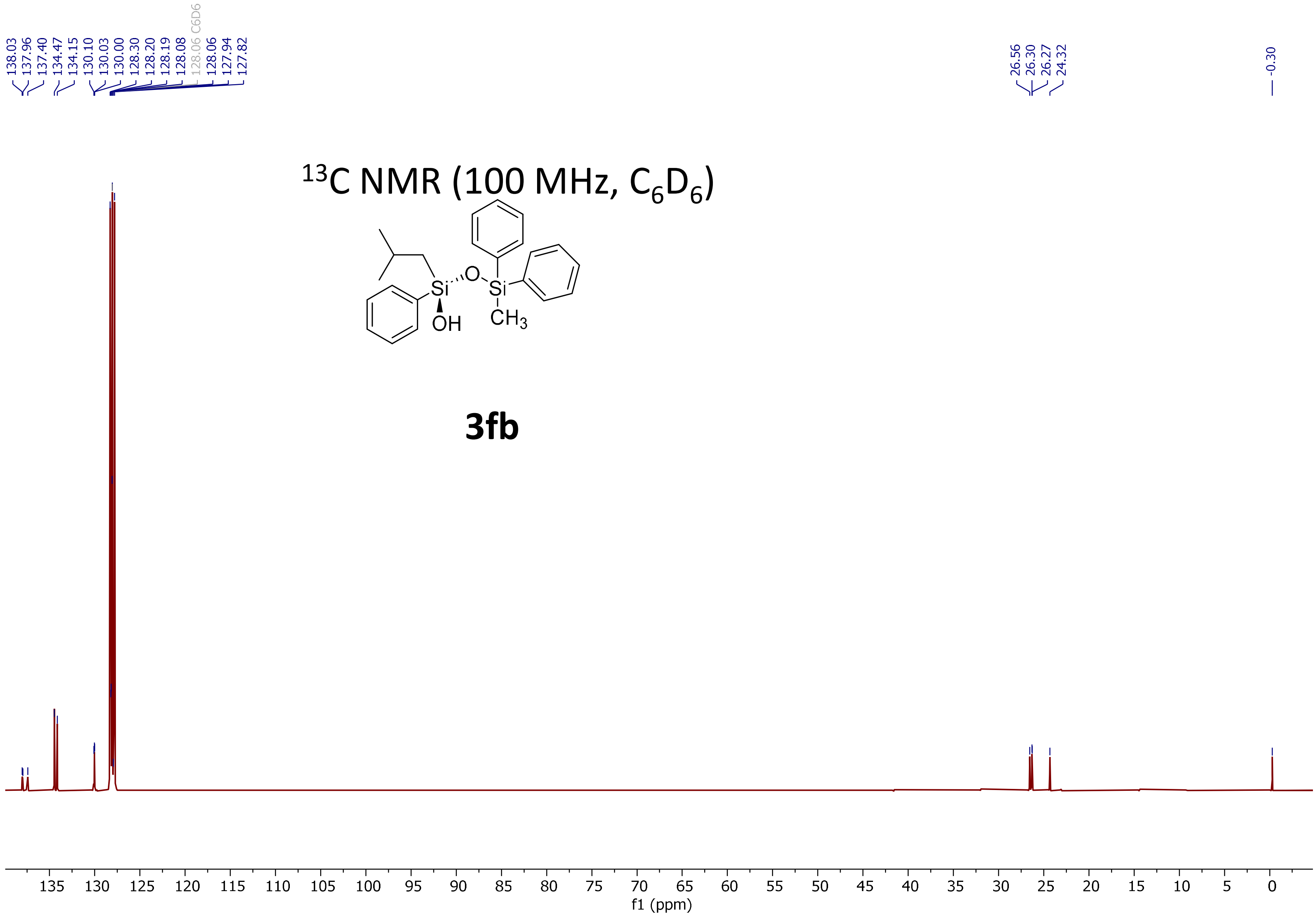
138.03
137.96
137.40
134.47
134.15
130.10
130.03
130.00
128.30
128.20
128.19
128.08
128.06 C6D6
128.06
127.94
127.82
26.56
26.30
26.27
24.32
-0.30
13C NMR (100 MHz, C6D6)
OH
CH3
O
Si
Si
3fb
f1 (ppm)

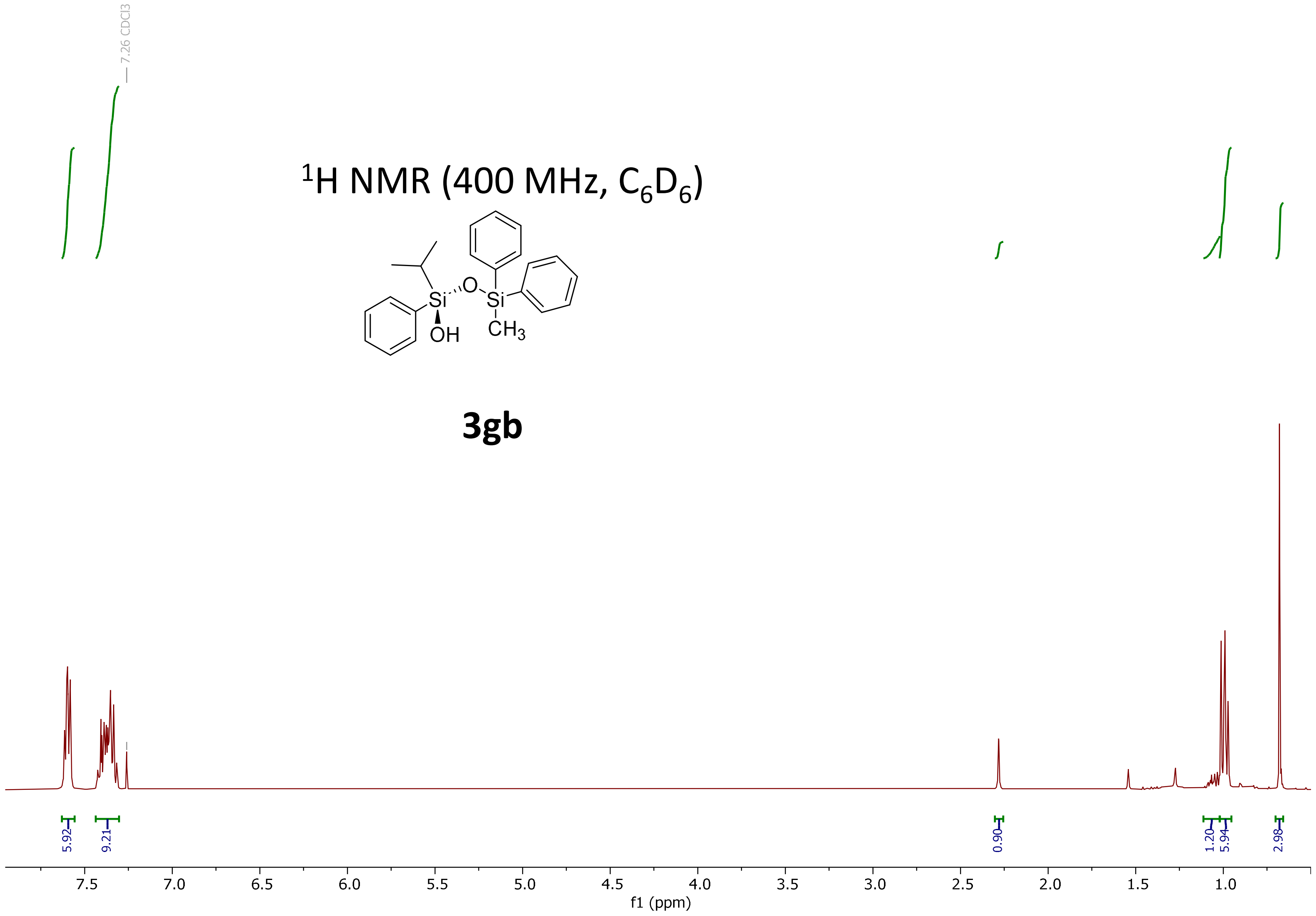

7.26 CDCl3
^{1}H NMR (400 MHz, C_6D_6)
Si
O
Si
OH
CH_3
3gb
5.92
9.21
0.90
1.20
5.94
2.98
7.5
7.0
6.5
6.0
5.5
5.0
4.5
4.0
3.5
3.0
2.5
2.0
1.5
1.0
f1 (ppm)

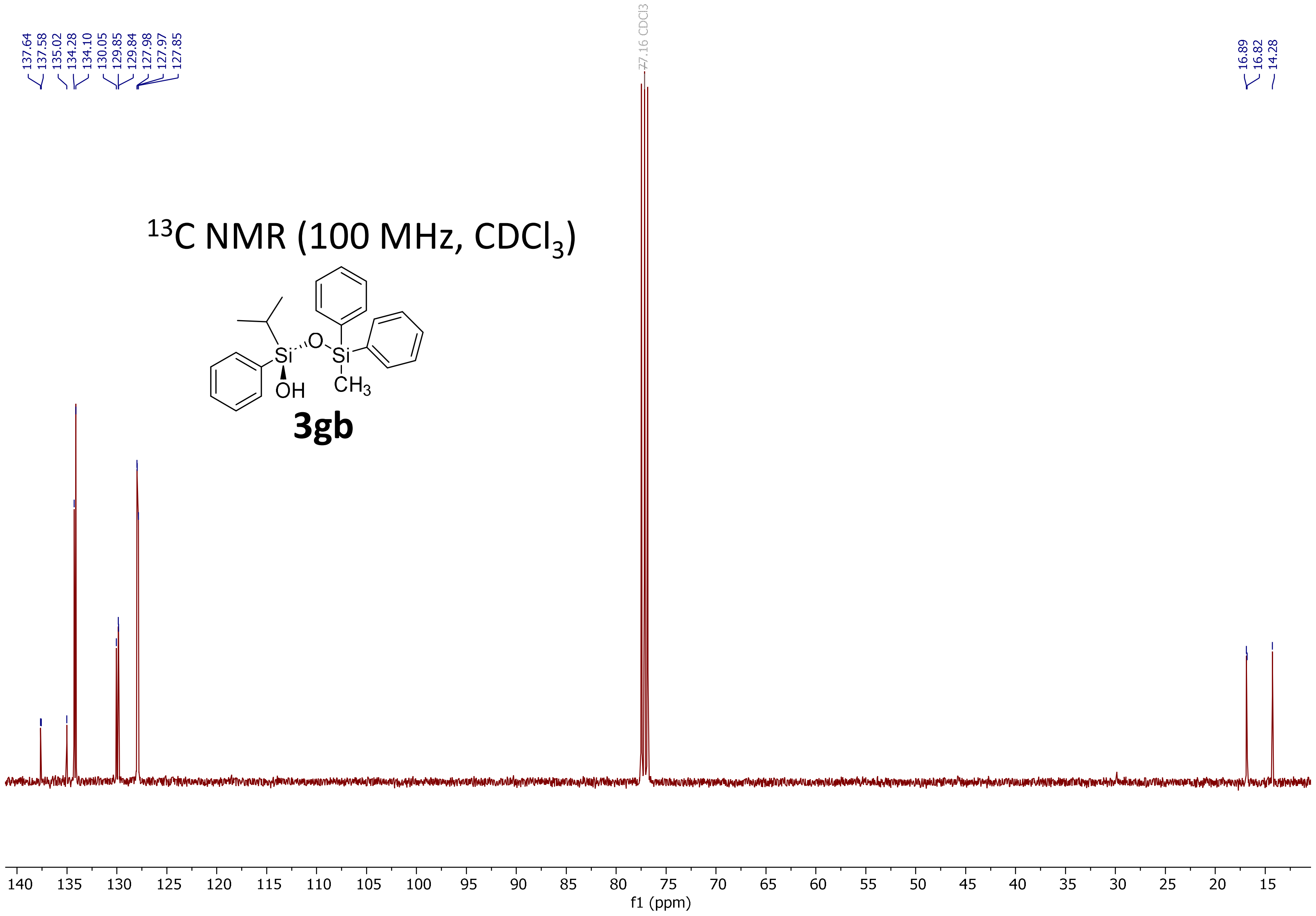
137.64
137.58
135.02
134.28
134.10
130.05
129.85
129.84
127.98
127.97
127.85
77.16 CDCl3
16.89
16.82
14.28
13C NMR (100 MHz, CDCl3)
Si
O
Si
OH
CH3
3gb
140 135 130 125 120 115 110 105 100 95 90 85 80 75 70 65 60 55 50 45 40 35 30 25 20 15
f1 (ppm)

^{1}H NMR (400 MHz, C_6D_6)

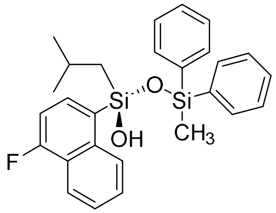

3cb

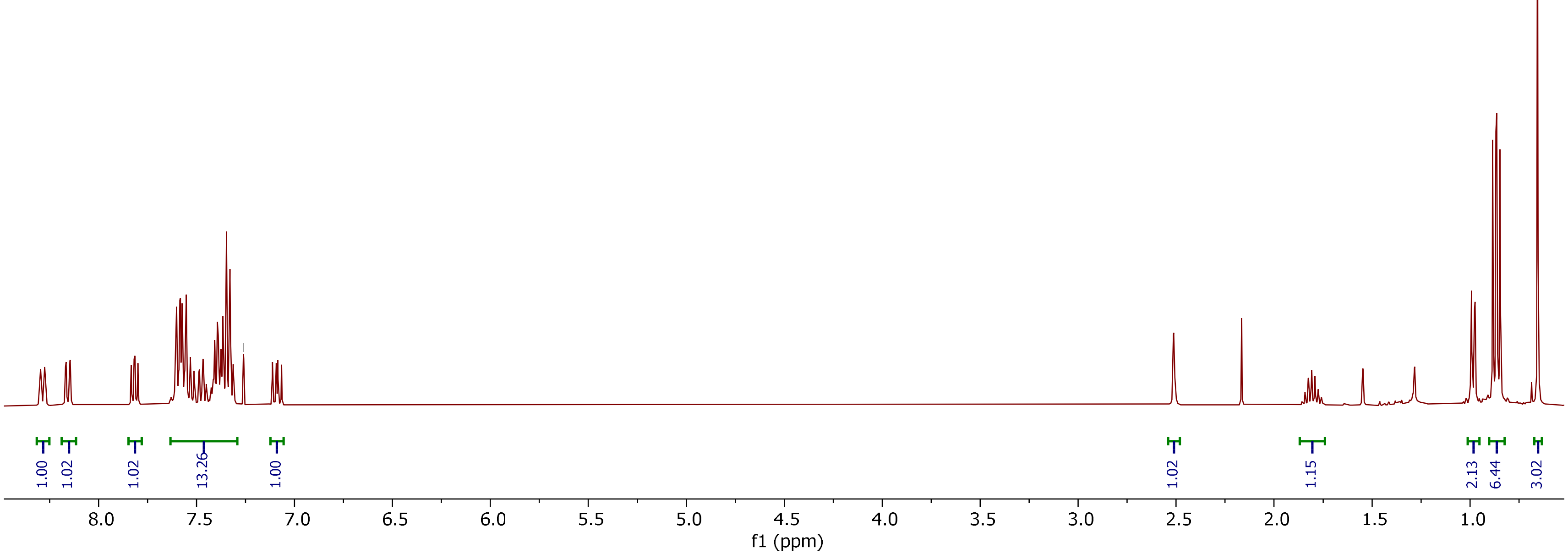

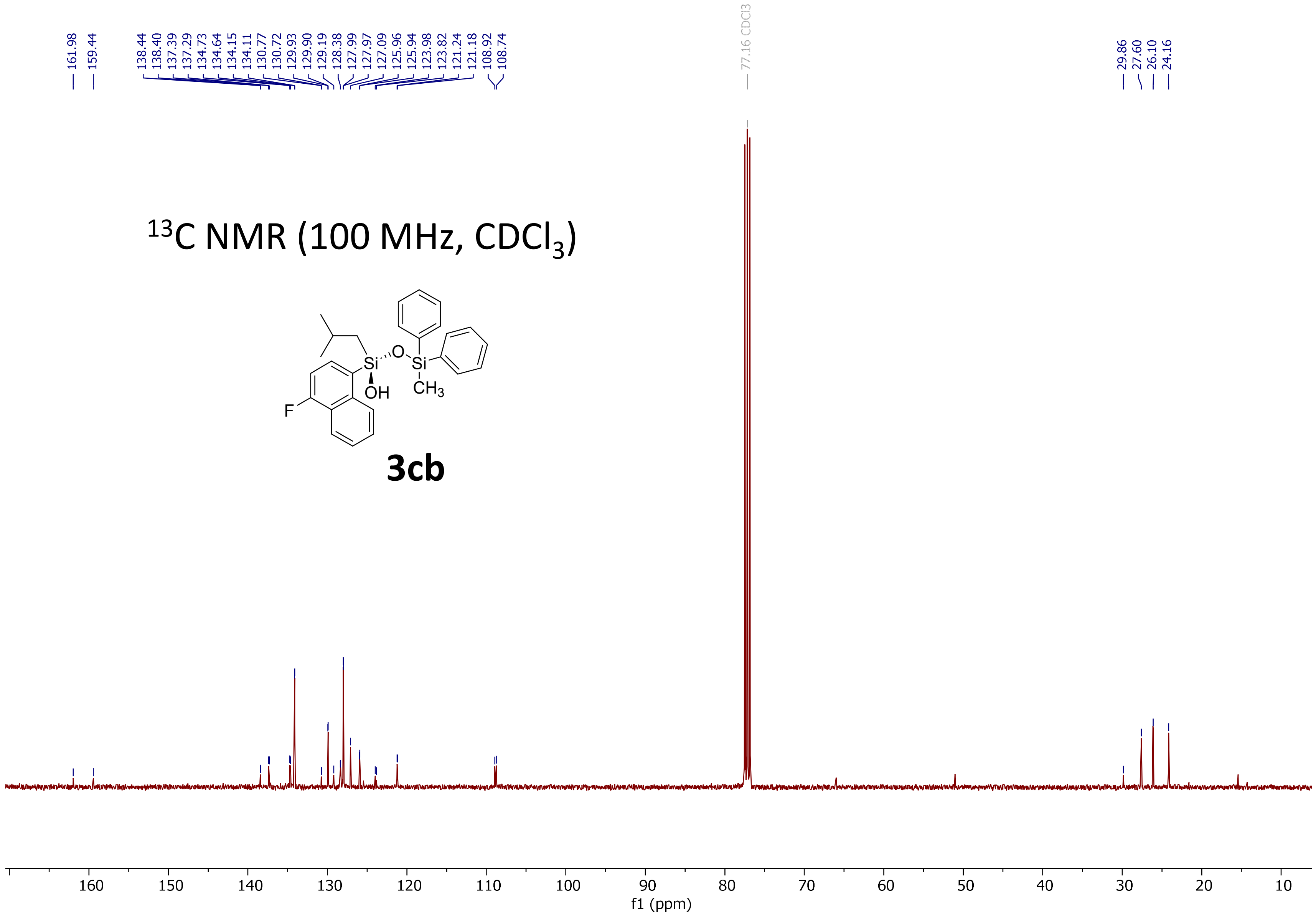

161.98
159.44
138.44
138.40
137.39
137.29
134.73
134.64
134.15
134.11
130.77
130.72
129.93
129.90
129.19
128.38
127.99
127.97
127.09
125.96
125.94
123.98
123.82
121.24
121.18
108.92
108.74
77.16 CDCl3
29.86
27.60
26.10
24.16
^{13}C NMR (100 MHz, $CDCl_3$)
Si
O
Si
OH
CH_3
F
3cb
160
150
140
130
120
110
100
90
80
70
60
50
40
30
20
10
f1 (ppm)

^1H NMR (400 MHz, C_6D_6)

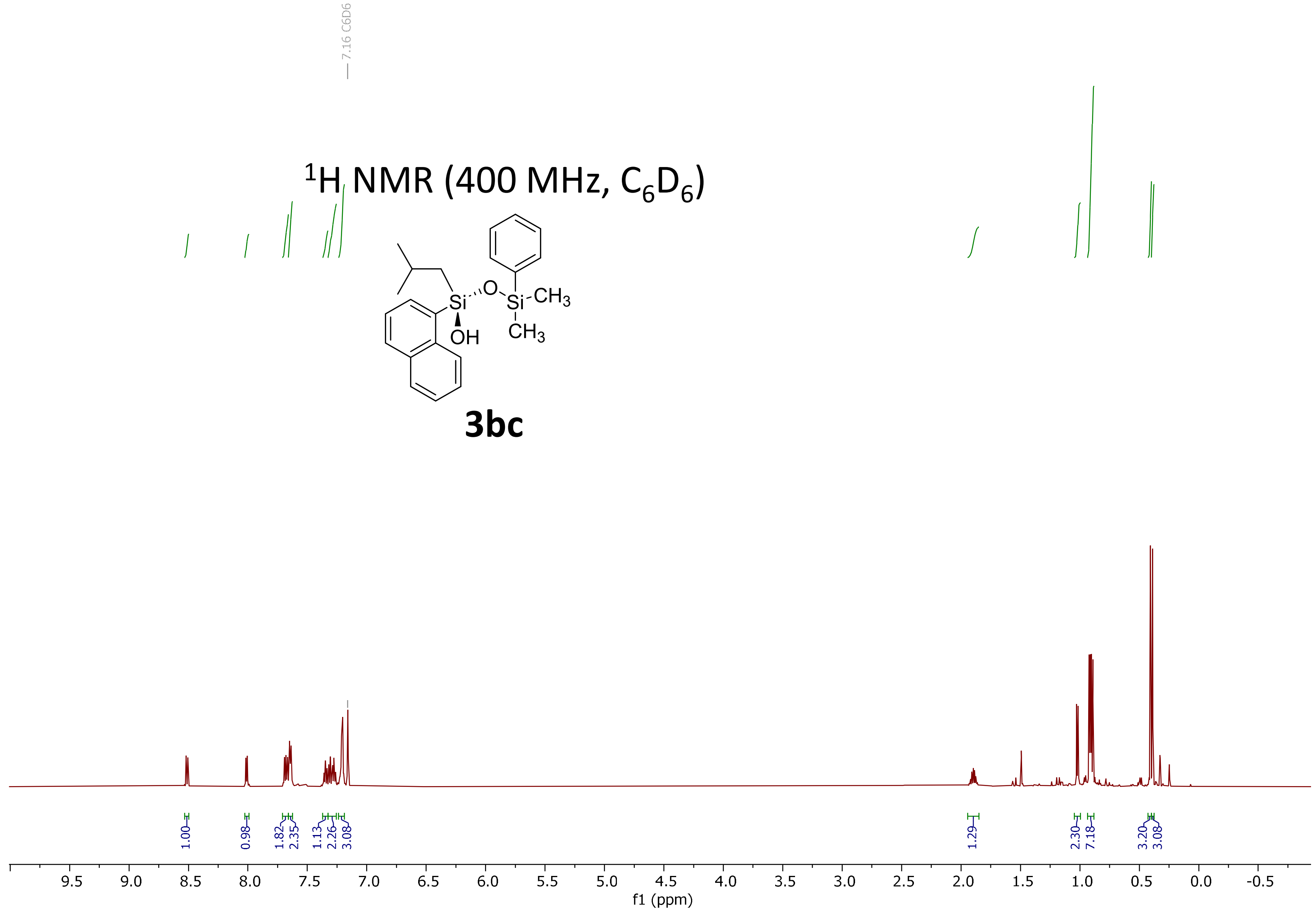

3bc

^{13}C NMR (100 MHz, C_6D_6)

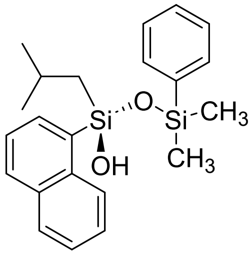

3bc

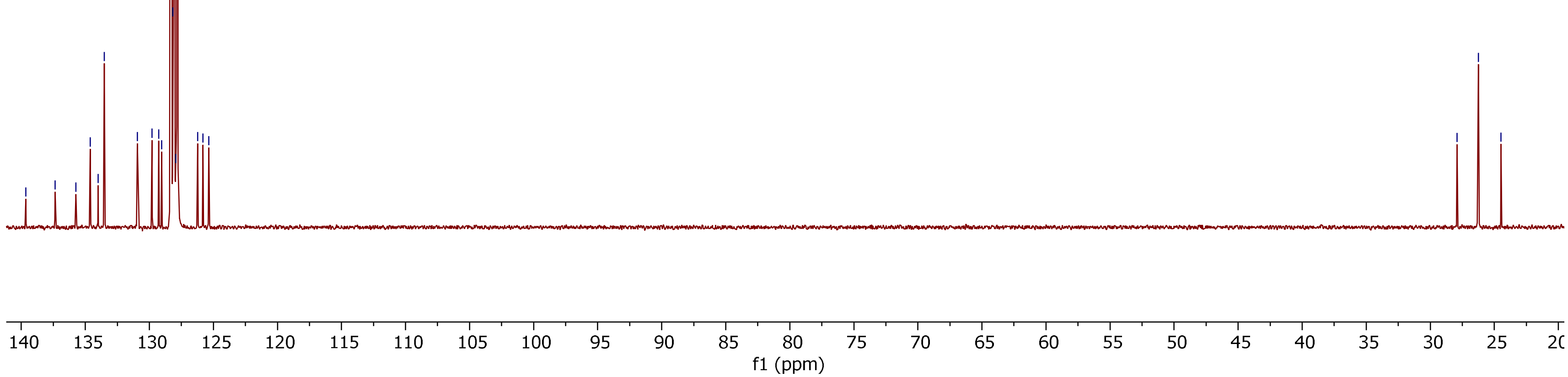

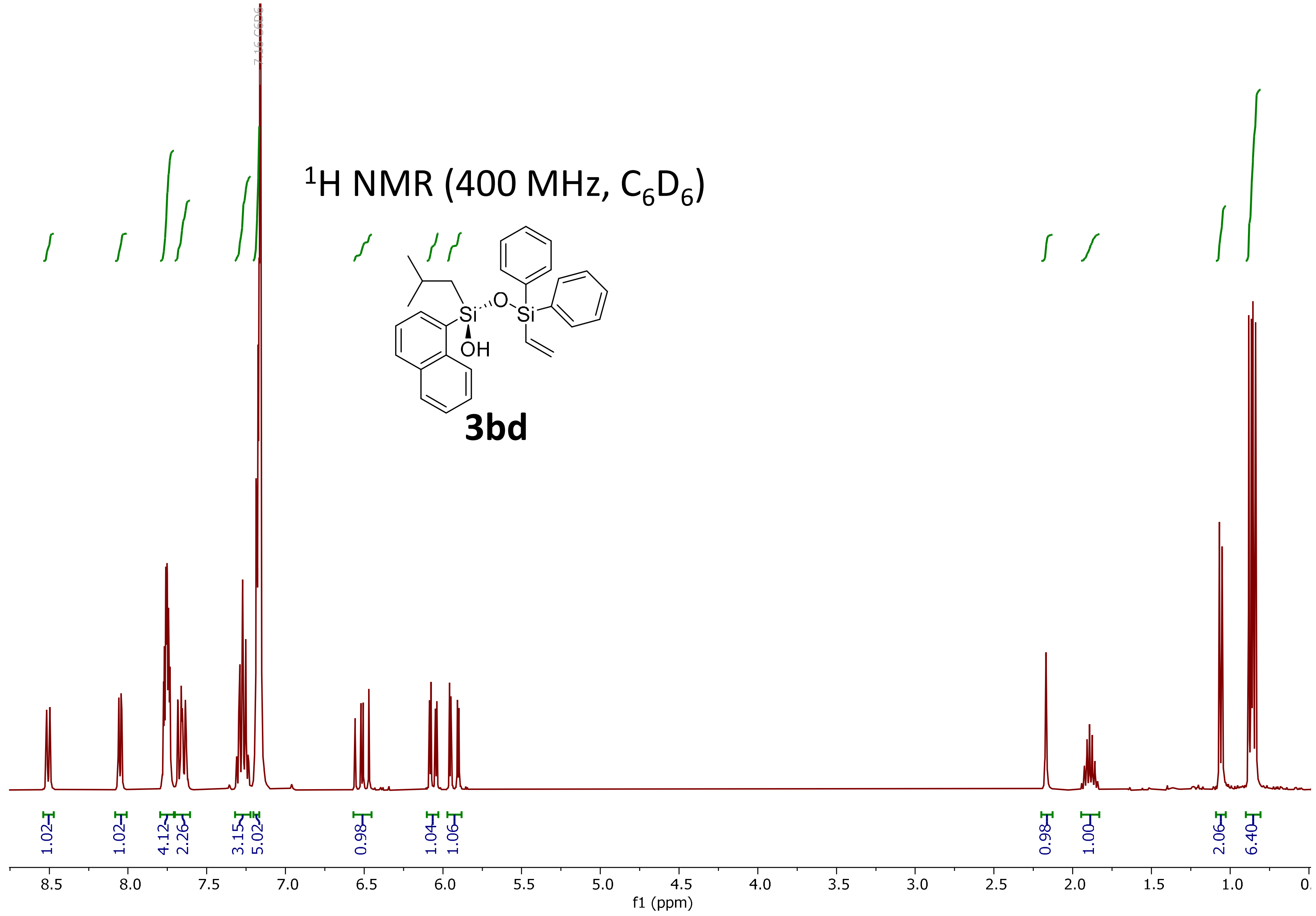

^{1}H NMR (400 MHz, C_6D_6)
7.16 C6D6
Si
O
Si
OH
3bd
1.02
1.02
4.12
2.26
3.15
5.02
0.98
1.04
1.06
0.98
1.00
2.06
6.40
8.5
8.0
7.5
7.0
6.5
6.0
5.5
5.0
4.5
4.0
3.5
3.0
2.5
2.0
1.5
1.0
f1 (ppm)

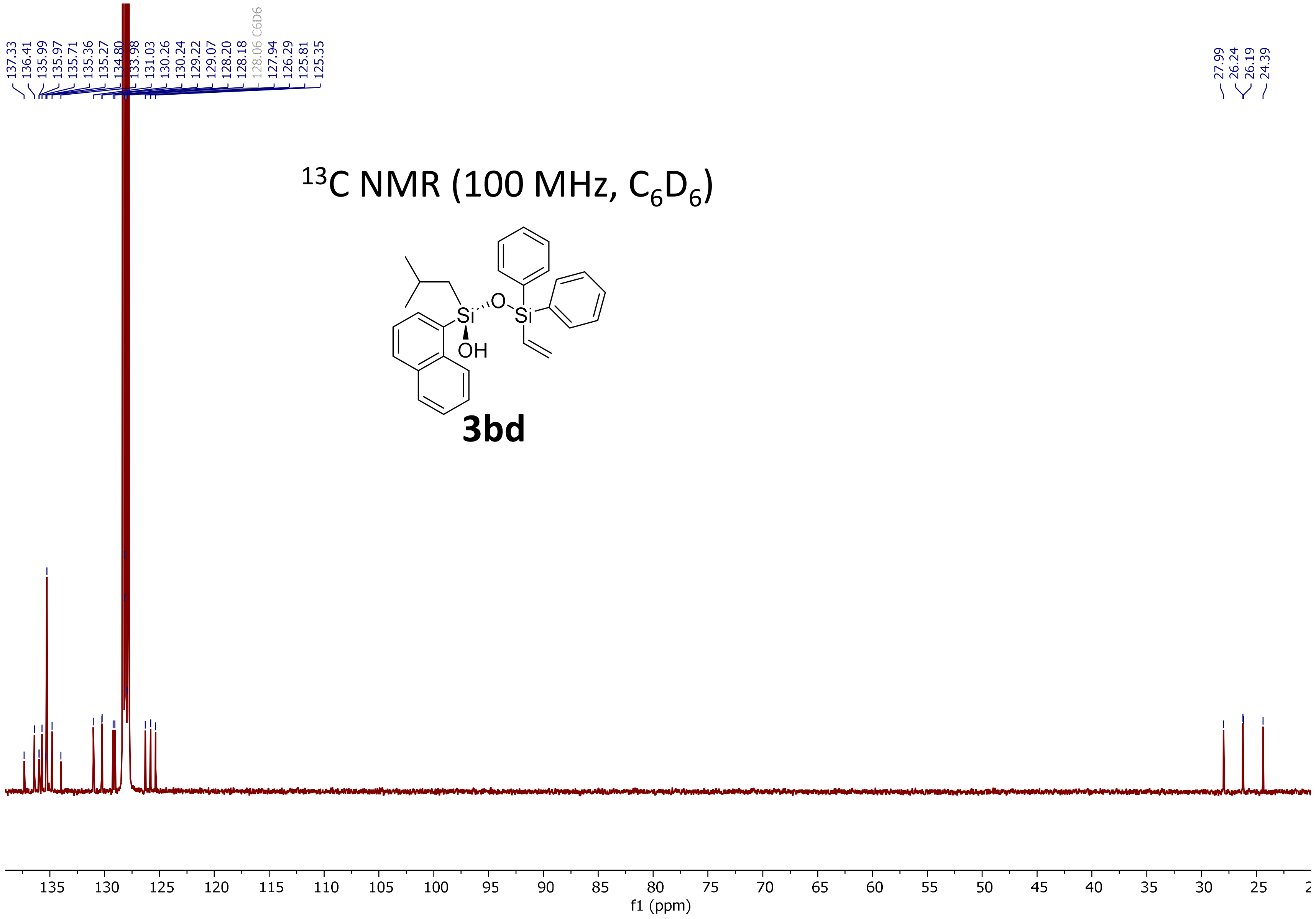
137.33
136.41
135.99
135.97
135.71
135.36
135.27
134.80
133.98
131.03
130.26
130.24
129.22
129.07
128.20
128.18
128.06 C6D6
127.94
126.29
125.81
125.35
27.99
26.24
26.19
24.39
13C NMR (100 MHz, C6D6)
Si
O
Si
OH
f1 (ppm)

3bd

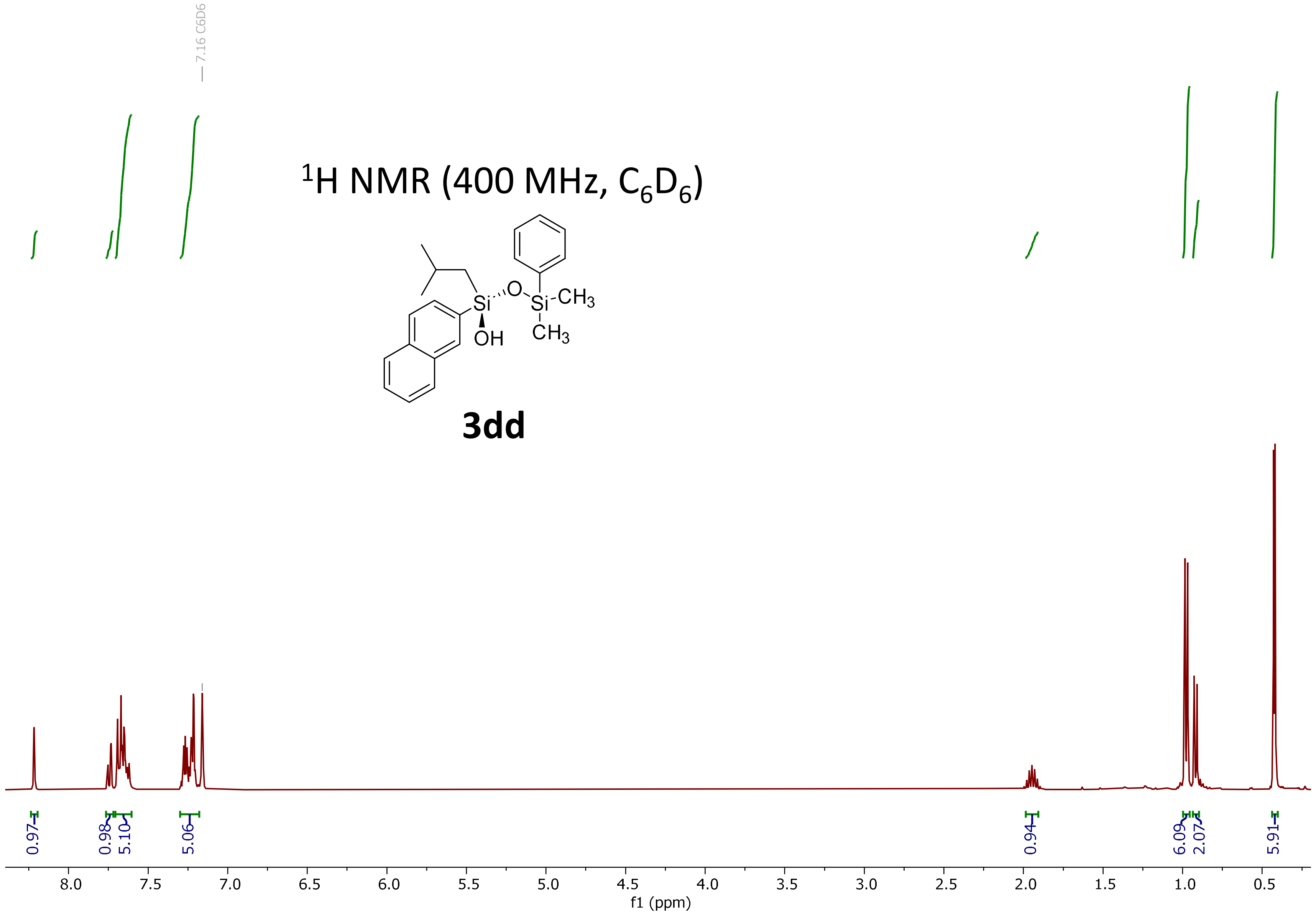
7.16 C6D6
1H NMR (400 MHz, C6D6)
Si
O
Si
CH3
CH3
OH
3dd
0.97
0.98
5.10
5.06
0.94
6.09
2.07
5.91
8.0
7.5
7.0
6.5
6.0
5.5
5.0
4.5
4.0
3.5
3.0
2.5
2.0
1.5
1.0
0.5
f1 (ppm)

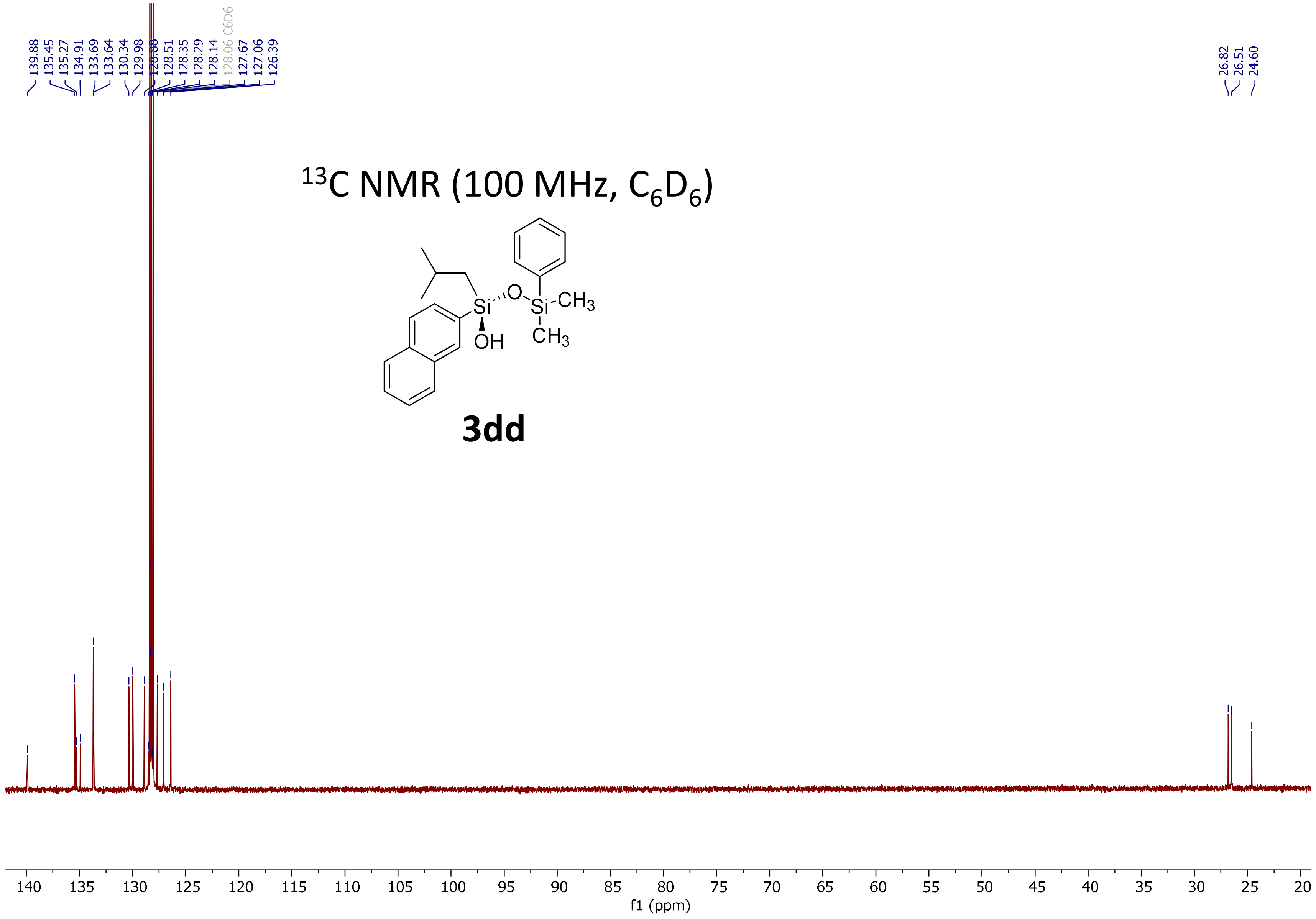
139.88
135.45
135.27
134.91
133.69
133.64
130.34
129.98
128.88
128.51
128.35
128.29
128.14
128.06 C6D6
127.67
127.06
126.39
26.82
26.51
24.60
^{13}C NMR (100 MHz, C_6D_6)
Si
O
Si
CH_3
OH
CH_3
3dd
140
135
130
125
120
115
110
105
100
95
90
85
80
75
70
65
60
55
50
45
40
35
30
25
20
f1 (ppm)

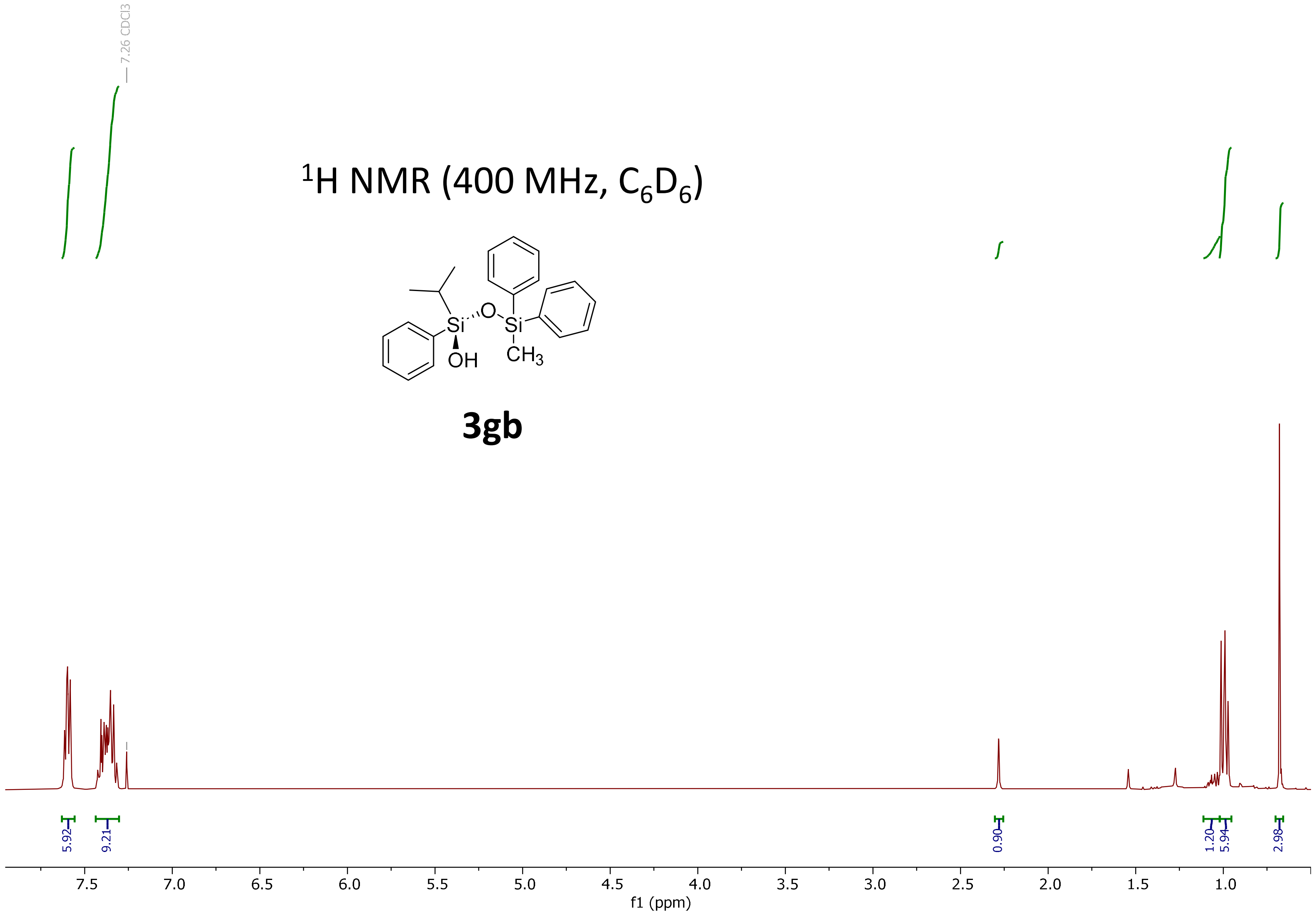

1H NMR (400 MHz, C6D6)
Si
O
Si
OH
CH3
3gb
7.26 CDCl3
5.92
9.21
0.90
1.20
5.94
2.98
7.5
7.0
6.5
6.0
5.5
5.0
4.5
4.0
3.5
3.0
2.5
2.0
1.5
1.0
f1 (ppm)

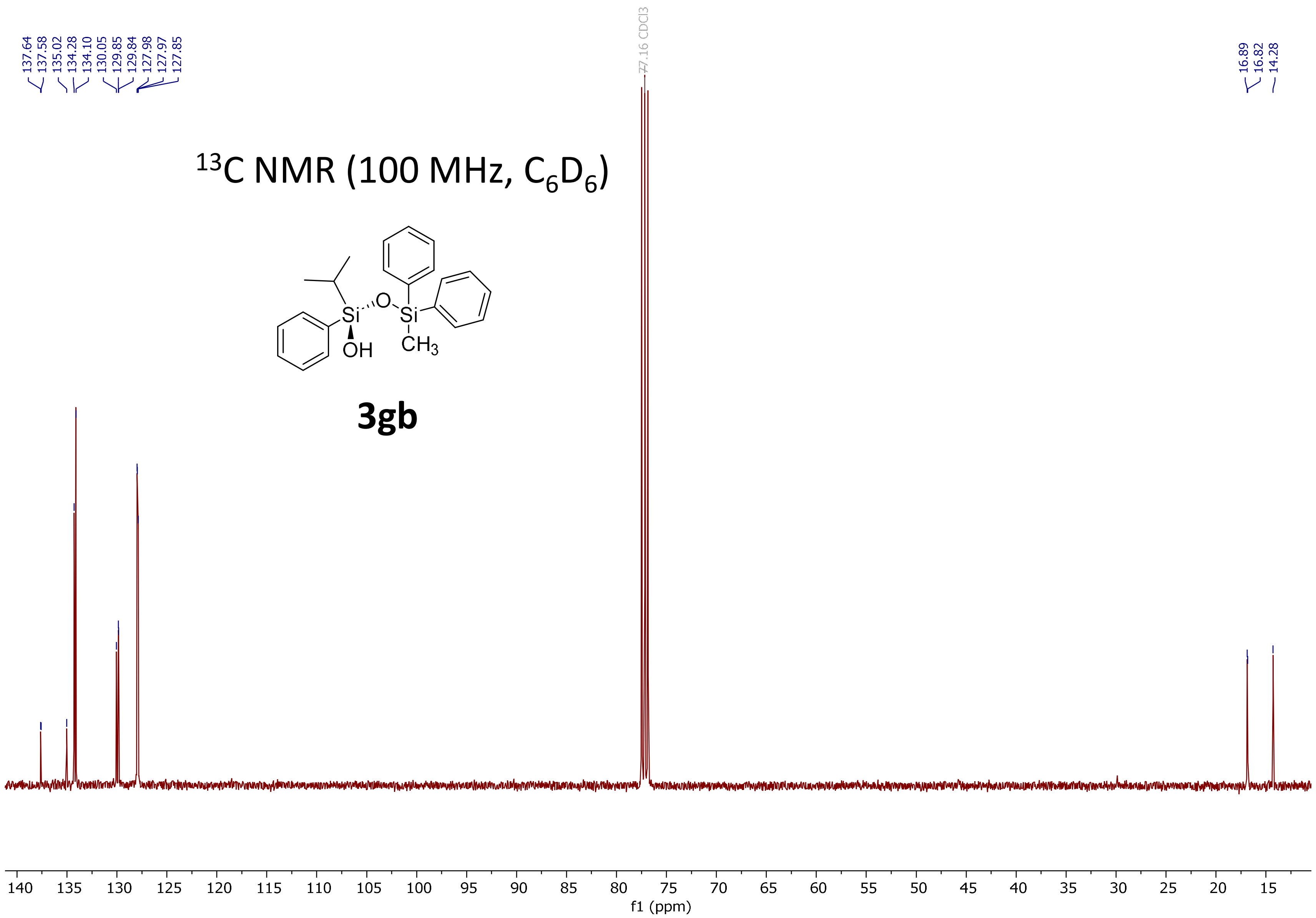

137.64
137.58
135.02
134.28
134.10
130.05
129.85
129.84
127.98
127.97
127.85
77.16 CDCl3
16.89
16.82
14.28
^{13}C NMR (100 MHz, C_6D_6)
Si
O
Si
OH
CH_3
3gb
f1 (ppm)

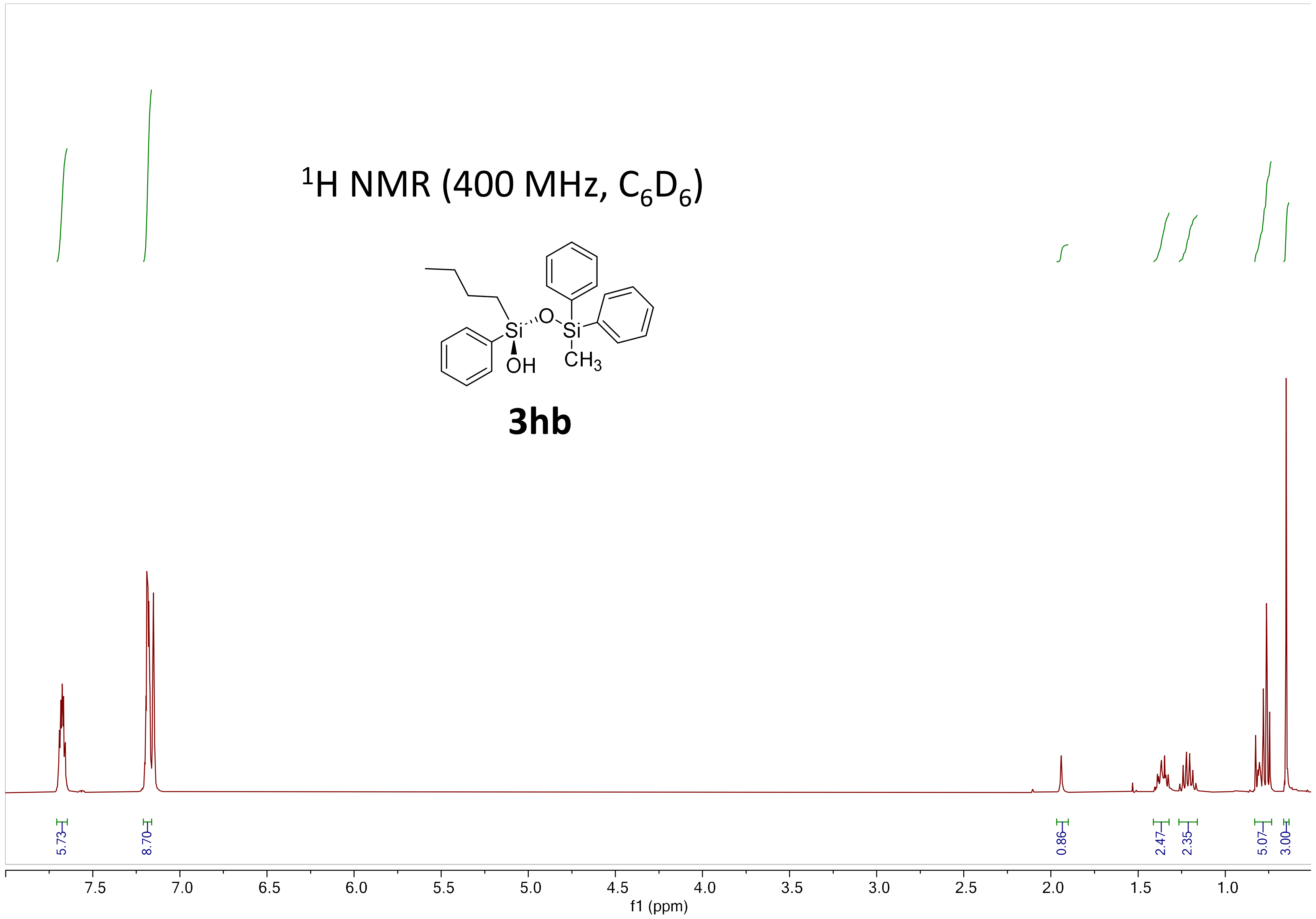

^1H NMR (400 MHz, C_6D_6)
3hb
5.73
8.70
0.86
2.47
2.35
5.07
3.00
7.5
7.0
6.5
6.0
5.5
5.0
4.5
4.0
3.5
3.0
2.5
2.0
1.5
1.0
f1 (ppm)

^{13}C NMR (100 MHz, C_6D_6)

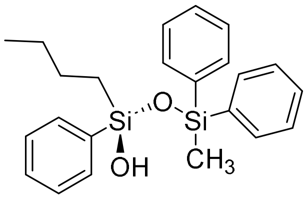

3hb

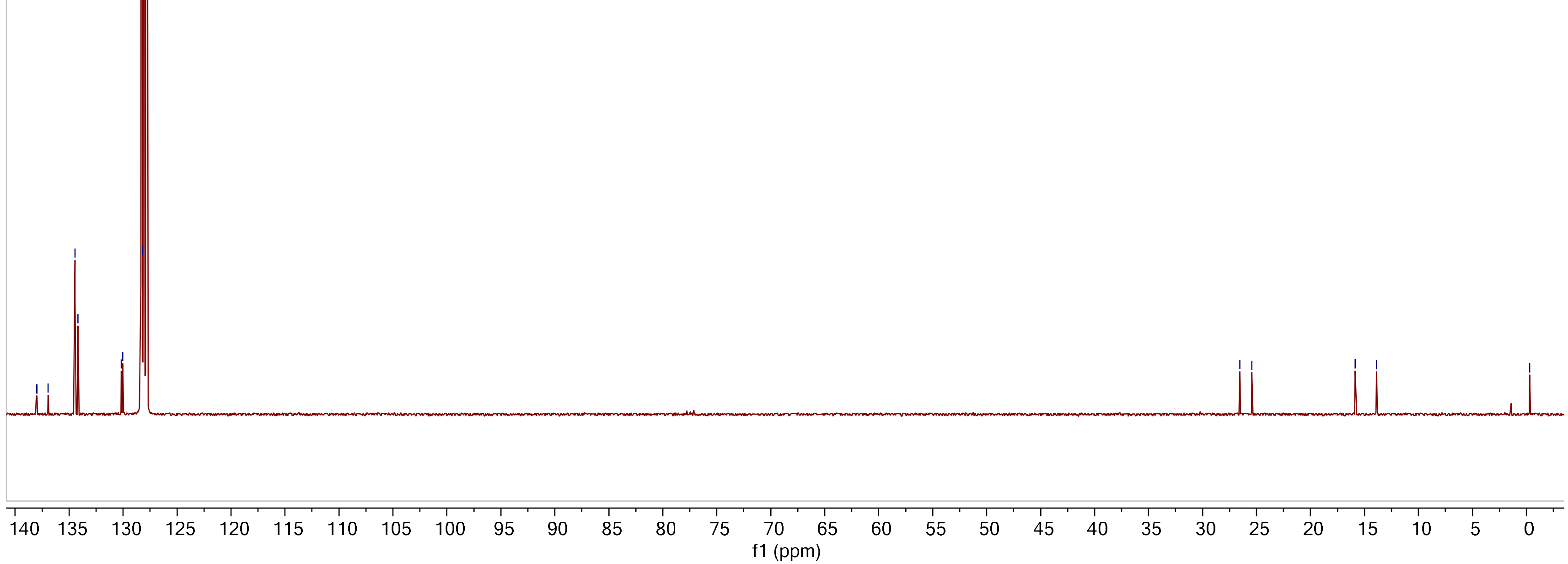

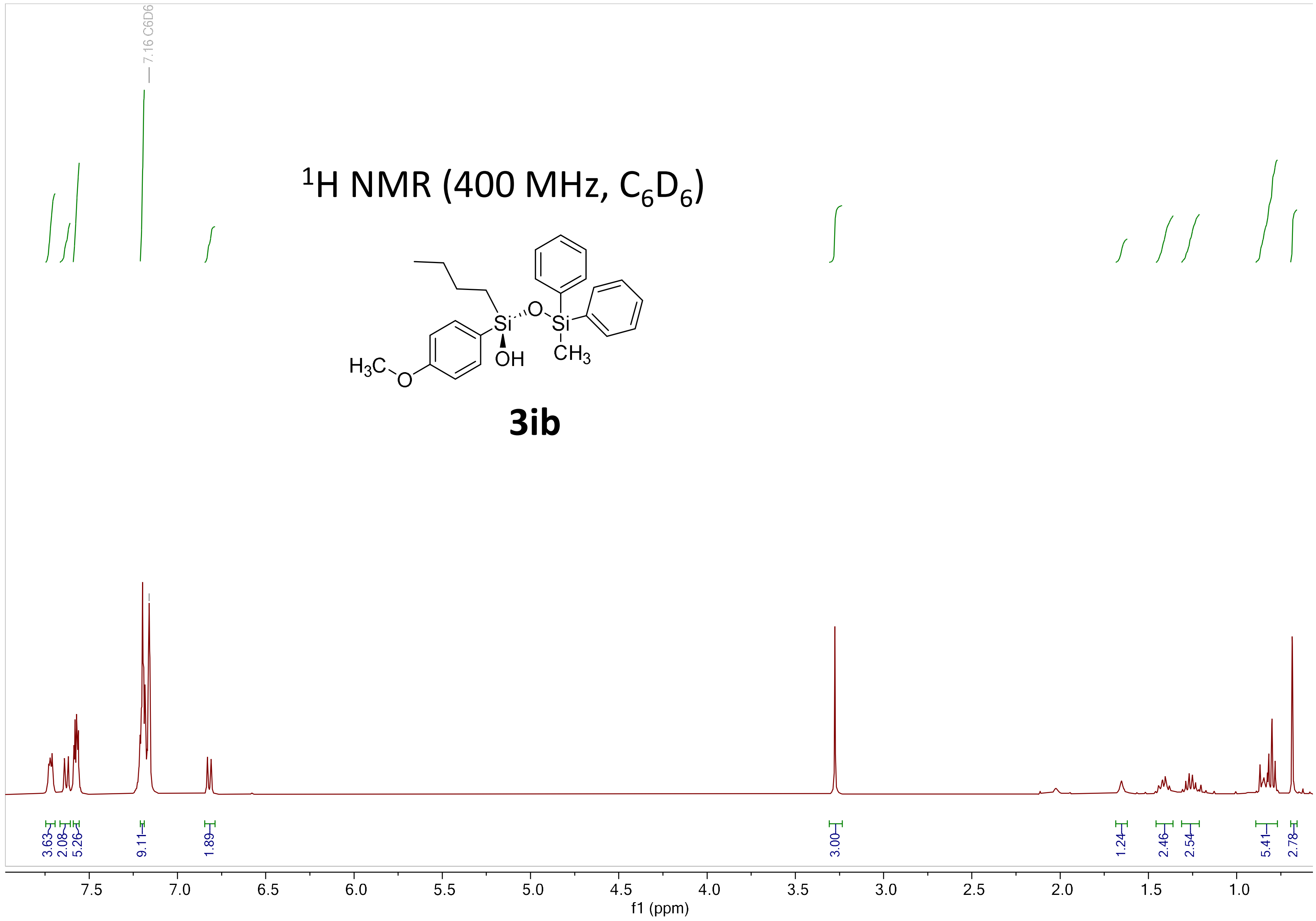

7.16 C6D6
^1H NMR (400 MHz, C_6D_6)
H_3C
O
Si
OH
O
Si
CH_3
3ib
3.63
2.08
5.26
9.11
1.89
3.00
1.24
2.46
2.54
5.41
2.78
7.5
7.0
6.5
6.0
5.5
5.0
4.5
4.0
3.5
3.0
2.5
2.0
1.5
1.0
f1 (ppm)

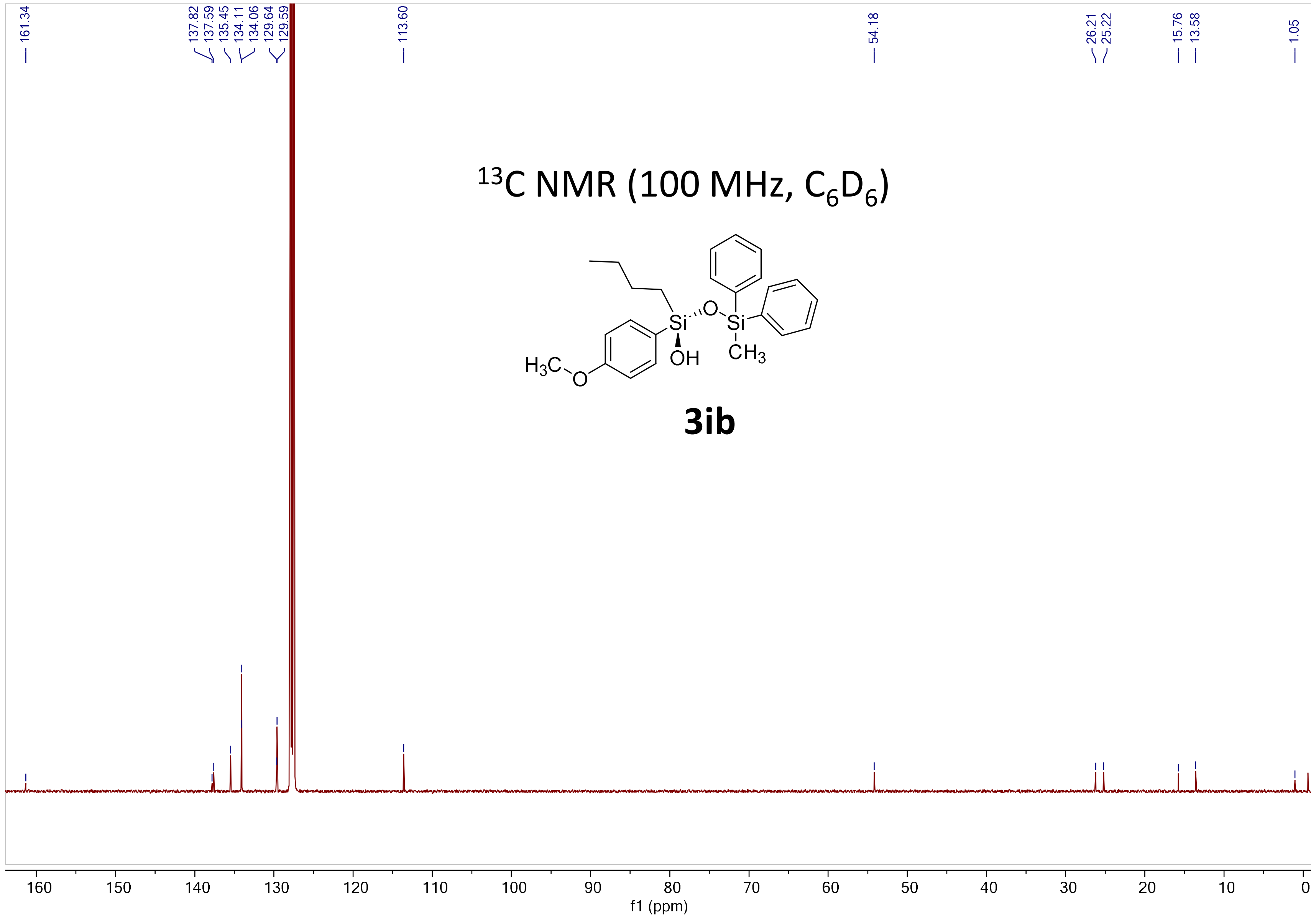

161.34
137.82
137.59
135.45
134.11
134.06
129.64
129.59
113.60
54.18
26.21
25.22
15.76
13.58
1.05
^{13}C NMR (100 MHz, C_6D_6)
H_3C
O
Si
OH
O
Si
CH_3
3ib
160 150 140 130 120 110 100 90 80 70 60 50 40 30 20 10 0
f1 (ppm)

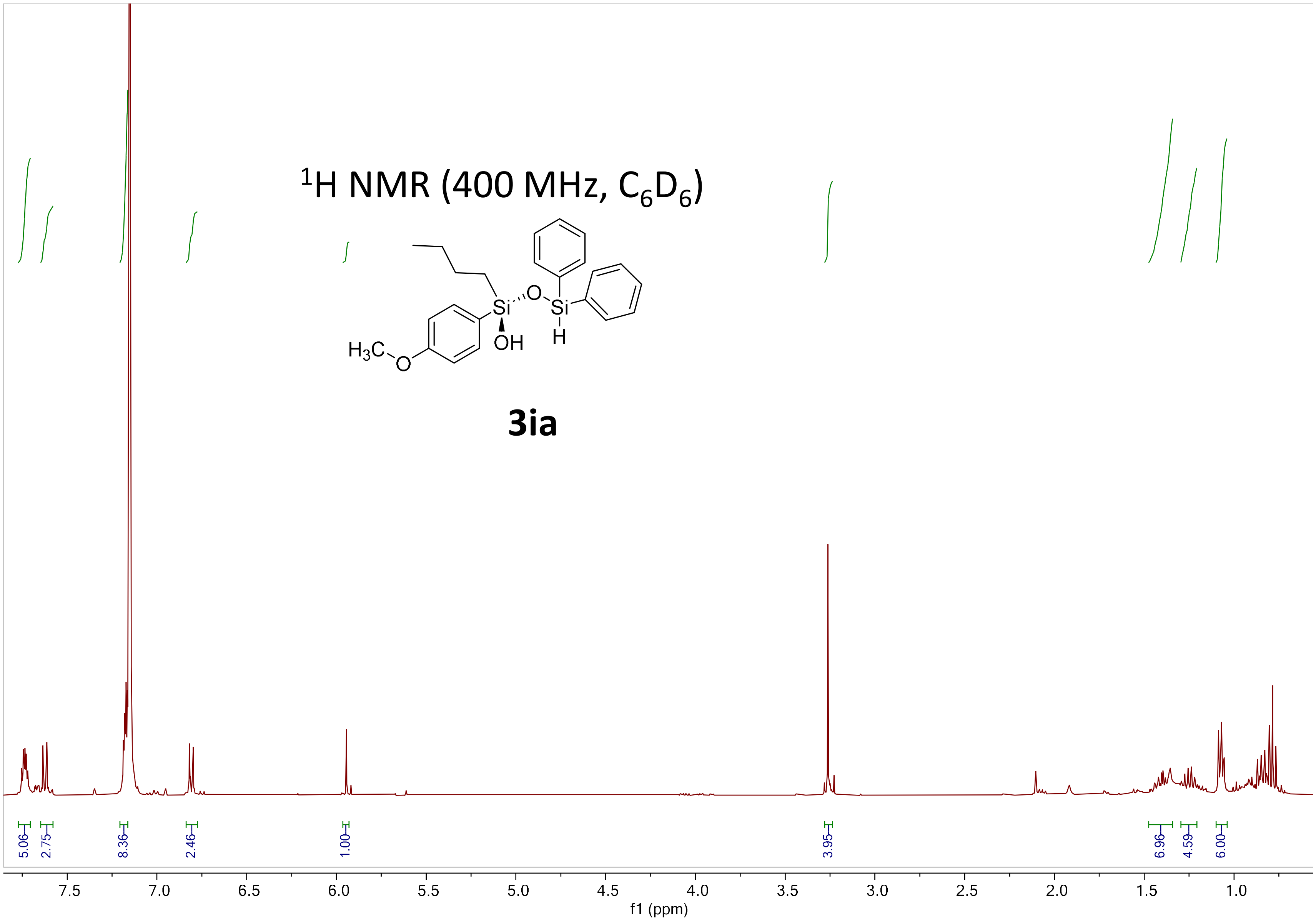

^{1}H NMR (400 MHz, C_6D_6)
H3C
O
Si
OH
O
Si
H
3ia
5.06
2.75
8.36
2.46
1.00
3.95
6.96
4.59
6.00
7.5
7.0
6.5
6.0
5.5
5.0
4.5
4.0
3.5
3.0
2.5
2.0
1.5
1.0
f1 (ppm)

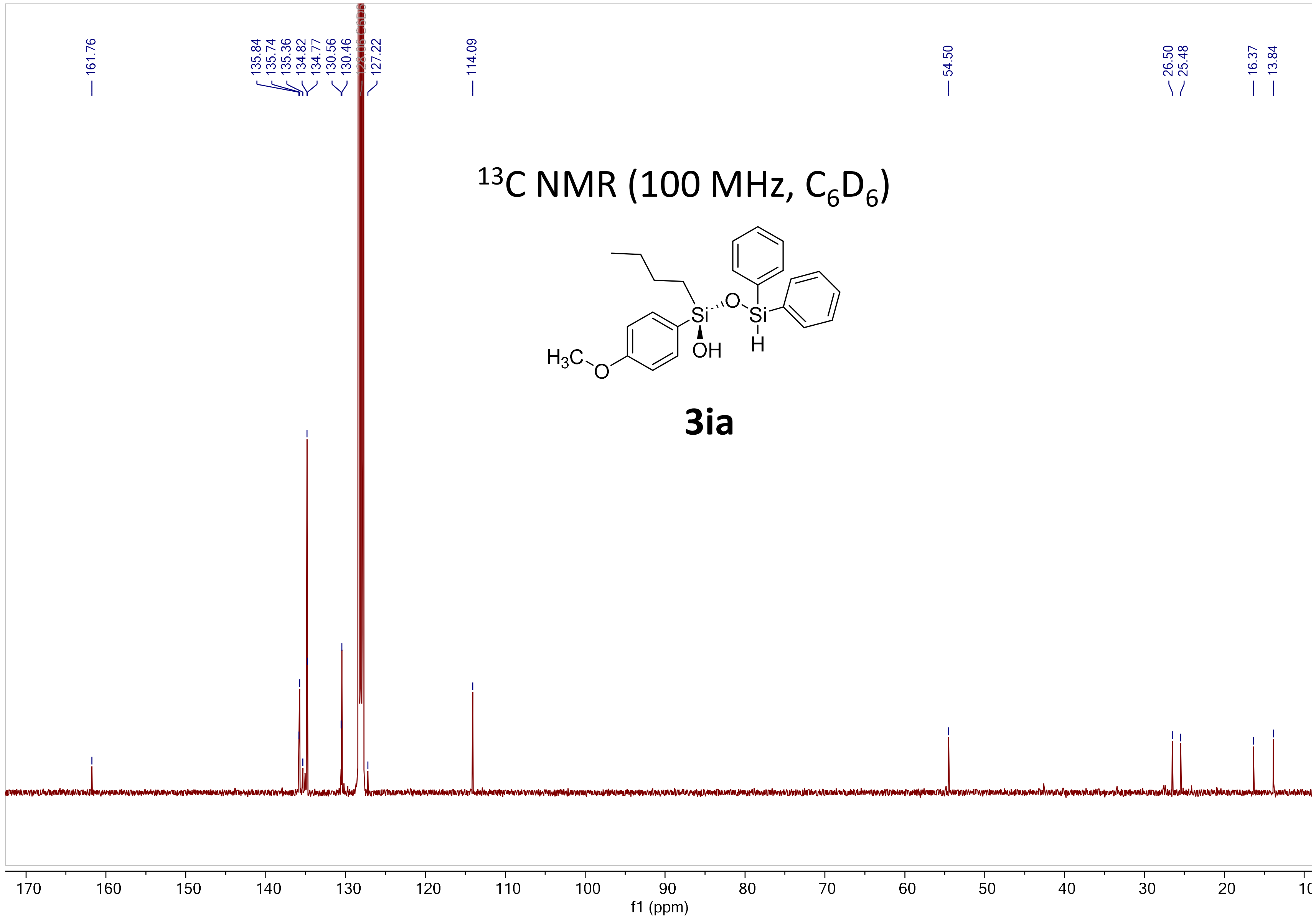

161.76
135.84
135.74
135.36
134.82
134.77
130.56
130.46
127.22
114.09
54.50
26.50
25.48
16.37
13.84
13C NMR (100 MHz, C6D6)
H3C
O
Si
O
Si
OH
H
3ia
170
160
150
140
130
120
110
100
90
80
70
60
50
40
30
20
f1 (ppm)
